# Ligands with 1,10-phenanthroline scaffold for highly regioselective iron-catalyzed alkene hydrosilylation

**DOI:** 10.1038/s41467-017-02472-6

**Published:** 2018-01-15

**Authors:** Meng-Yang Hu, Qiao He, Song-Jie Fan, Zi-Chen Wang, Luo-Yan Liu, Yi-Jiang Mu, Qian Peng, Shou-Fei Zhu

**Affiliations:** 1 State Key Laboratory and Institute of Elemento-Organic Chemistry, Tianjin, 300071 China; 2 Collaborative Innovation Center of Chemical Science and Engineering (Tianjin), College of Chemistry, Nankai University, Tianjin, 300071 China

## Abstract

Transition-metal-catalyzed alkene hydrosilylation is one of the most important homogeneous catalytic reactions, and the development of methods that use base metals, especially iron, as catalysts for this transformation is a growing area of research. However, the limited number of ligand scaffolds applicable for base-metal-catalyzed alkene hydrosilylation has seriously hindered advances in this area. Herein, we report the use of 1,10-phenanthroline ligands in base-metal catalysts for alkene hydrosilylation. In particular, iron catalysts with 2,9-diaryl-1,10-phenanthroline ligands exhibit unexpected reactivity and selectivity for hydrosilylation of alkenes, including unique benzylic selectivity with internal alkenes, Markovnikov selectivity with terminal styrenes and 1,3-dienes, and excellent activity toward aliphatic terminal alkenes. According to the mechanistic studies, the unusual benzylic selectivity of this hydrosilylation initiates from *π*–*π* interaction between the phenyl of the alkene and the phenanthroline of the ligand. This ligand scaffold and its unique catalytic model will open possibilities for base-metal-catalyzed hydrosilylation reactions.

## Abstract (web summary)

Hydrosilylation of alkenes poses substantial challenges in terms of regioselectivity. Here, the authors report iron complexes with 1,10-phenantroline ligand scaffolds which  display benzylic selectivity in the hydrosilylation of internal alkenes and Markovnikov selectivity with terminal styrenes and 1,3-dienes.

## Introduction

Ligand design lies at the heart of transition-metal catalysis because ligands play critical roles in the tuning of catalyst activity and selectivity. Although many ligands have been developed, most are based on the same few scaffolds (core structures)^1–3^. The emergence of new types of ligand scaffolds generally revolutionizes the catalytic reactions for which they are used. Therefore, the history of transition-metal catalysis roughly parallels the history of the development of ligands with privileged scaffolds.

Transition-metal-catalyzed alkene hydrosilylation is one of the most important homogeneous catalytic reactions and has been widely used both for basic research and for the industrial production of organosilicons^4^. Precious metals, especially platinum, are the predominant catalysts employed for this transformation^5^, and large amounts of platinum are consumed every year for industrial hydrosilylation of alkenes^6^. The development of methods that instead employ base metals, especially iron, as catalysts is a growing area of research^7^. Base-metal catalysts provide new opportunities for addressing the challenges presented by precious-metal catalysts. The design of suitable ligands is critical to the development of efficient base-metal catalysts. In an elegant example of ligand design, Chirik and co-workers^8^ introduced tridentate bis(imino)pyridine ligands into highly active iron catalysts for selective hydrosilylation reactions of terminal alkenes. This seminal work triggered the design of additional ligands with a pyridine and/or imine scaffold for use in base-metal-catalyzed hydrosilylation reactions^9–15^. However, the number of ligand scaffolds applicable for base-metal-catalyzed alkene hydrosilylation remains limited. This shortage of scaffolds seriously limits the catalytic utility of base metals: the base-metal catalysts developed to date are suitable for the hydrosilylation of terminal alkenes only and cannot be used with internal alkenes. The development of new ligand scaffolds that could overcome this limitation is highly desirable.

Although 1,10-phenanthroline derivatives coordinate strongly to many metals and have been widely used as ligands in transition-metal catalysis^16^, it is surprising that ligands containing this scaffold have never, to our knowledge, been used in alkene hydrosilylation. As part of our ongoing work on iron-catalyzed reactions^17–19^, we herein report the development of bidentate ligands based on a 1,10-phenanthroline scaffold (Darphen, **1**) and their use for highly efficient iron-catalyzed alkene hydrosilylation. Catalysts bearing these ligands showed unexpected substrate reactivity and selectivity for iron-catalyzed hydrosilylation of alkenes, including unique benzylic selectivity for internal alkenes, Markovnikov selectivity for terminal styrenes and 1,3-dienes, and excellent activity toward aliphatic terminal alkenes. A set of control experiments and density functional theory (DFT) calculations were also performed to understand the mechanism and to rationalize these unusual selectivities. The mechanistic studies indicate that the benzylic selectivity of this hydrosilylation initiates from *π*–*π* interaction between the phenyl of the alkene and the phenanthroline of the ligand.

## Results

### Synthesis of catalysts

**Fig. 1:**
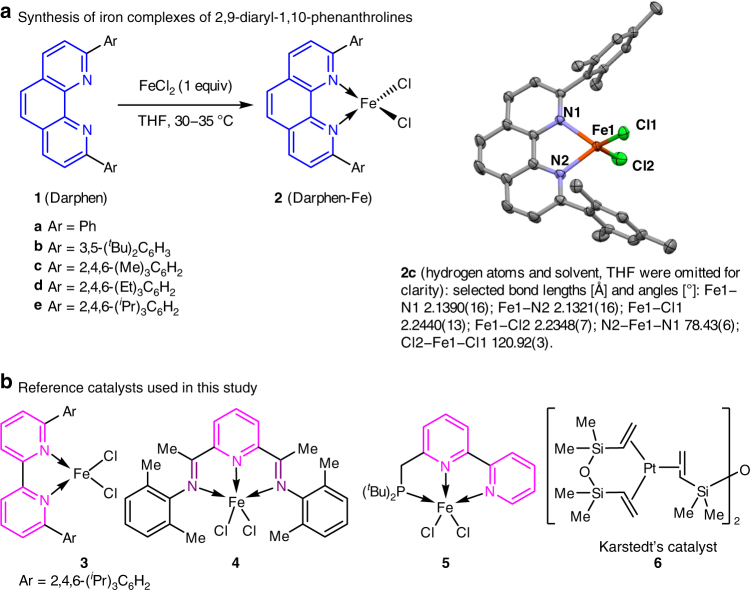
Ligands and catalysts used in this study. **a** Synthesis of iron complexes of 2,9-diaryl-1,10-phenanthrolines. The diaryl groups in ligand **1** may prevent another **1** molecule from coordinating with the iron center. **b** These catalysts are high-effective catalysts for the hydrosilylation of terminal alkenes

We synthesized a series of new iron complexes with 2,9-diaryl-1,10-phenanthroline ligands (Darphen-Fe, **2a–e**, Fig. 1a). The corresponding Darphen ligands (**1a–e**) were easily prepared in high yields from 1,10-phenanthroline by a modified literature procedure^20^. Single-crystal X-ray diffraction analysis (Supplementary Table 1) showed that complex **2c** has a distorted tetrahedral structure and contains only one molecule of **1c**, which acts as a bidentate ligand. The bulky diaryl groups of **1** may prevent coordination of more than one ligand molecule to the iron center.

### Hydrosilylation of alkenes

We began by evaluating the catalytic activity of **2** for hydrosilylation of internal alkenes. We chose internal alkenes because although their hydrosilylation has potential utility for the preparation of new organosilicon reagents, however it poses challenges in terms of regioselectivity and substrate reactivity^4,7^. Unlike the two sides of a terminal alkene, the two sides of a dissymmetric internal alkene cannot be readily discriminated. Moreover, the higher steric bulk of internal alkenes hinders hydrosilylation and often favors competitive side reactions, such as isomerization, polymerization, and reduction. For example, although the α-hydrosilylation of β-alkyl styrenes with trichlorosilane, an inorganic silane, has been achieved with palladium catalysts^21,22^, in the presence of base-metal catalysts, internal alkenes undergo rapid alkenyl isomerization to form terminal alkenes before hydrosilylation^12,23–26^.

We chose β-methyl styrene (**7a**) as the initial model substrate and phenylsilane as the silylation reagent (Table 1). As a reductant, we used the Grignard reagent EtMgBr, which is thought to reduce iron(II) complexes to active low-valent iron species^27^. Although iron complex **2a**, which has a 2,9-phenyl-1,10-phenanthroline ligand, was inactive in the hydrosilylation reaction (Table 1, entry 1), complexes **2b–e**, which have bulkier aryl groups in the ligands, exclusively afforded the product of benzylic hydrosilylation in moderate to high yields (Table 1, entries 2–5). No other hydrosilylation product was detected in all these cases. The catalyst performance was strongly influenced by the steric bulk of the aryl groups in ligands **1**, and catalyst **2e**, which has a 2,4,6-isopropylphenyl ligand, gave the best results (>95% conversion and 91% yield, Table 1, entry 5).

**Table 1:** Transition-metal-catalyzed hydrosilylation of β-methyl styrene **7a** with phenylsilane^a^


Entry	[M]	Reductant	Solvent	Conv. (%)^b^	Yield (%)^c^
1	**2a**	EtMgBr	THF	0	ND
2	**2b**	EtMgBr	THF	41	35
3	**2c**	EtMgBr	THF	70	65
4	**2d**	EtMgBr	THF	88	82
5	**2e**	EtMgBr	THF	>95	91
6	**2e**	EtMgCl	THF	>95	90
7	**2e**	MeMgBr	THF	>95	88
8	**2e**	PhMgBr	THF	>95	90
9	**2e**	(*p*-Tol)MgBr	THF	>95	88
10	**2e**	^*n*^BuLi	THF	>95	90
11	**2e**	LiAlH_4_	THF	>95	89
12	**2e**	NaHBEt_3_	THF	29	20
13	**2e**	^*t*^BuOK	THF	75	68
14	**2e**	EtMgBr	None	>95	95
15^d^	**2e**	EtMgBr	None	>95	95
16^e^	**2e**	EtMgBr	None	>95	95
17	**3**	EtMgBr	None	0	ND
18	**4**	EtMgBr	None	0	ND
19	**5**	EtMgBr	None	0	ND
20	H_2_PtCl_6_·6H_2_O	None	None	0	ND
21	**6**	None	None	0	ND
22^f^	PdCl_2_/MOP	None	None	0	ND

ND, not determined

^a^ Reaction conditions: **7a**/PhSiH_3_/[M]/reductant = 0.25:0.275:0.0125:0.0275 (mmol), in 1 mL solvent, at 30 °C, 24 h

^b^ Conversion of **7a**. Determined by ^1^H NMR using 1,3,5-trimethoxybenzene as internal standard

^c^ Isolated yield

^d^ **7a**/PhSiH_3_/**2e**/EtMgBr = 2:2.2:0.04:0.088 (mmol), at 30 °C, 24 h

^e^ Gram-scale experiment: used 5 mmol **7a**, 5.5 mmol PhSiH_3_, 1 mol% **2e**, and 2.2 mol% EtMgBr, and 1.07 g of **8a** was obtained

^f^ MOP = (2′-methoxy-[1,1′-binaphthalen]-2-yl)diphenylphosphane

The reaction conditions were then systematically studied. Other organometallic reductants, such as EtMgCl, MeMgBr, PhMgBr, (*p*-Tol)MgBr, ^*n*^BuLi, and LiAlH_4_,^19^ gave outcomes similar to those obtained with EtMgBr (Table 1, entries 6–11). Although having similar reactivity, the active catalysts generated from different activators might be different.^28–30^ In contrast, NaHBEt_3_ and ^*t*^BuOK gave lower conversion and yield (Table 1, entries 12 and 13). When the reaction was performed without solvent, the catalyst loading could be reduced to 1 mol%, and the reaction could be scaled up to gram scale without diminishing either the yield or the regioselectivity (Table 1, entries 14‒16). Although the iron complex was decomposed during the work-up, ligand **1e** was recovered in 90% yield, indicating its high stability under the reaction conditions. The 1,10-phenanthroline scaffold of ligands **1** was critical for the unusual substrate reactivities of the corresponding catalysts. A ligand with a 2,2′-bipyridine scaffold (**3**, Fig. 1b) was totally inactive under identical reaction conditions (Table 1, entry 17). Iron catalysts with pyridine- and imine-bearing ligands (**4** and **5**, respectively, Fig. 1b), which efficiently catalyze the hydrosilylation of terminal alkenes^7^, failed to afford the desired hydrosilylation product (Table 1, entries 18 and 19). Note that well-established precious-metal-based hydrosilylation catalysts^4^, such as H_2_PtCl_6_·6H_2_O (Speier’s catalyst), **6** (Karstedt’s catalyst, Fig. 1b), and a palladium catalyst modified with a phosphine ligand, were inactive for the hydrosilylation of **7a** (Table 1, entries 20‒22). These control experiments clearly demonstrate the superiority of our iron catalysts with bidentate phenanthroline ligands for the hydrosilylation of internal alkenes. Trace metal contamination analysis of iron precatalysts (Supplementary Table 2) and a set of background experiments (Supplementary Table 3) indicate that the iron is the real catalyst in this reaction.

Using catalyst **2e** under the optimized conditions, we then carried out hydrosilylation reactions of various β-alkyl styrene derivatives **7** (Table 2). First, the effect of the alkyl group R of the substrate was evaluated (Table 2, entries 1–10). Hydrosilylation reactions of substrates **7** with a wide range of R groups exhibited similar good yields and selectivities (Table 2, entries 1–9). The only exception was **7j**, which has a sterically bulky *tert*-butyl group and gave only a moderate yield of **8j** (Table 2, entry 10). Note that in the reaction of **7h**, the highly reactive cyclopropane moiety, which is directly connected to the alkenyl group, remained intact (Table 2, entry 8). The steric and electronic properties of the substituents on the benzene ring of the substrates had negligible effects on the reaction outcome; all the tested styrene derivatives (**7k**–**7p**) afforded high yields (90–94%) with exclusive benzylic selectivity (Table 2, entries 11–16). Substrates with fused aromatic ring systems, such as 2-vinylnaphthalene (**7q**) and 5-vinylbenzo[*d*][1,3]dioxole (**7r**), also gave satisfactory yields and regioselectivities (Table 2, entries 17 and 18, respectively). We observed the dehydrocoupling of phenylsilane as a side reaction in almost all reactions but with only <5% yield. The robustness screen experiments^31^ indicate that the reaction can tolerate ketone, ester, alkyne, borates, and several heteroaromatics, but sensitive to aldehyde, amide, and nitriles (Supplementary Table 4).

**Table 2:** Iron-catalyzed hydrosilylation of β-alkyl styrene with phenylsilane^a^


Entry	Ar	R	**7**	**8**	Yield (%)
1	Ph	Me	**7a**	**8a**	95
2	Ph	Et	**7b**	**8b**	93
3	Ph	^*n*^Pr	**7c**	**8c**	92
4	Ph	^*n*^Bu	**7d**	**8d**	93
5	Ph	^*i*^Bu	**7e**	**8e**	93
6	Ph	Bn	**7f**	**8f**	92
7	Ph	^*i*^Pr	**7g**	**8g**	89
8	Ph		**7h**	**8h**	92
9	Ph		**7i**	**8i**	92
10	Ph	^*t*^Bu	**7j**	**8j**	68
11	2-MeC_6_H_4_	^*n*^Bu	**7k**	**8k**	90
12	3-MeC_6_H_4_	^*n*^Bu	**7l**	**8l**	92
13	4-MeC_6_H_4_	^*n*^Bu	**7m**	**8m**	94
14	2-FC_6_H_4_	^*n*^Bu	**7n**	**8n**	93
15	4-FC_6_H_4_	^*n*^Bu	**7o**	**8o**	94
16	4-MeOC_6_H_4_	^*n*^Bu	**7p**	**8p**	92
17^b^	2-naphthyl	^*n*^Bu	**7q**	**8q**	92
18		^*n*^Bu	**7r**	**8r**	91

^a^ Reaction conditions: **7**/PhSiH_3_/**2e**/EtMgBr = 2:2.2:0.04:0.088 (mmol), at 30 °C, 24 h. Isolated yields were given. Exclusive benzylic selectivity was observed in all reactions

^b^ Reaction conditions: **7q**/PhSiH_3_/**2e**/EtMgBr = 0.25:0.275:0.0125:0.0275 (mmol), in 1 mL THF, at 30 °C, 24 h

Although Markovnikov selectivity has been achieved in the cobalt- and nickel-catalyzed alkene hydrosilylation,^13,32–37^ and in iron- and cobalt-catalyzed alkene hydroboration,^38–44^ the known iron catalysts always exhibit anti-Markovnikov selectivity in the alkene hydrosilylation^7^. To our delight, catalyst **2b** developed in this study showed excellent Markovnikov selectivity (≥98%) and high yields (88–95%) in the hydrosilylation of terminal styrenes **9** (Table 3). The steric and electronic properties of the substituents on the benzene ring of the substrates (**9a**‒**9l**) had little effect on the reaction outcome. Substrates with fused aromatic ring systems, such as 1-vinylnaphthalene (**9m**), 2-vinylnaphthalene (**9n**), and 5-vinylbenzo[*d*][1,3]dioxole (**9o**), also gave satisfactory yields.

**Table 3:** Iron-catalyzed hydrosilylation of terminal styrenes^a^


Entry	Ar	**9**	**10**	Yield (%)
1	Ph	**9a**	**10a**	92
2	2-MeC_6_H_4_	**9b**	**10b**	90
3	2-MeOC_6_H_4_	**9c**	**10c**	91
4^b^	2-FC_6_H_4_	**9d**	**10d**	90
5	3-MeC_6_H_4_	**9e**	**10e**	90
6	3-MeOC_6_H_4_	**9f**	**10f**	91
7^b^	3-FC_6_H_4_	**9g**	**10g**	90
8^b^	3-ClC_6_H_4_	**9h**	**10h**	90
9	4-MeC_6_H_4_	**9i**	**10i**	91
10	4-MeOC_6_H_4_	**9j**	**10j**	93
11	4-FC_6_H_4_	**9k**	**10k**	90
12^b^	4-ClC_6_H_4_	**9l**	**10l**	88
13	1-naphthyl	**9m**	**10m**	92
14	2-naphthyl	**9n**	**10n**	95
15^b^	Piperonyl	**9o**	**10o**	90

^a^ Reaction conditions: **9**/PhSiH_3_/**2b**/EtMgBr = 0.5:0.55:0.01:0.022 (mmol), in 1 mL THF, at 30–35 °C. The conversion of olefins is >95% and the Markovnikov selectivity is ≥98% in all cases

^b^ Used 5 mol% **2b** and 11 mol% EtMgBr

We then evaluated the use of iron catalyst **2e** for the hydrosilylation of some other internal alkenes (**11**) (Table 4). High benzylic selectivity was observed in the hydrosilylation of 1*H*-indene (**11a**), a cyclic dissymmetric internal alkene. Surprisingly, the reaction of unconjugated internal alkene *E*-**11b**, which has a methylene group between the phenyl and alkenyl moieties, also exhibited good benzylic selectivity (90%), which suggests that phenyl-directed migration of the double bond occurred before the hydrosilylation. To our knowledge, such reactivity and selectivity toward internal alkenes have not previously been reported. The geometry of the double bond of **11b** had little effect on the reaction outcome: a 3:1 *E*/*Z* mixture of **11b** exhibited essentially the same yield and benzylic selectivity as those obtained with pure *E*-**11b**. When an additional methylene group was introduced between the phenyl and alkenyl moieties (substrate **11c**), the hydrosilylation still occurred mainly at the benzyl position, but the selectivity was lower (76%). In contrast, during the reaction of pure alkyl internal alkene **11d**, the double bond migrated to the terminus of the molecule, and **12d**, which has a terminal silyl group, was obtained as the major product, owing to the lack of the phenyl directing group. Different with its isomer (*E*)-**7a** (Table 2, entry 1), the hydrosilylation of (*Z*)-1-phenyl-1-propene, (*Z*)-**7a** was sluggish and afforded a mixture of branched silane (**8a**) and linear silane (**16e**) with moderate yield (38%).

**Table 4:** Iron-catalyzed hydrosilylation of other internal alkenes^a^

^a^ Reaction conditions: **11**/PhSiH_3_/**2e**/EtMgBr = 0.5:0.6:0.025:0.055 (mmol), in 1 mL THF, at 30 °C, 24 h. Isolated yields were given

^b^ Used 0.55 mol PhSiH_3_

^c^ Reaction conditions: (*Z*)-**7a**/PhSiH_3_/**2e**/EtMgBr = 2:2.2:0.04:0.088 (mmol), at 30 °C, 24 h

**Fig. 2:**
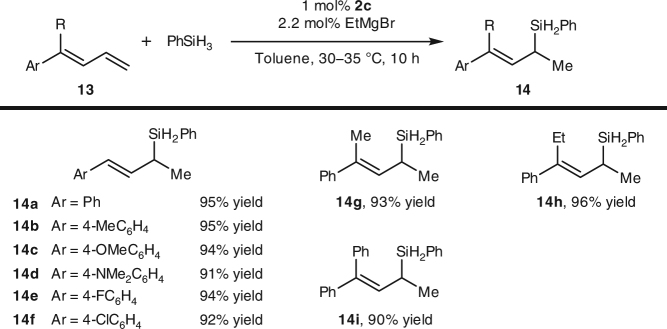
Iron-catalyzed hydrosilylation of 1-substituted and 1,1-disubstituted buta-1,3-dienes. High yields (91–95%) with unique Markovnikov selectivities were observed for all the examined conjugated dienes

We also evaluated iron catalysts **2** in the hydrosilylation of 1-substituted and 1,1-disubstituted buta-1,3-dienes **13** (Fig. 2). Unlike an iron catalyst modified with a tridentate bis(imino)pyridine ligand, which affords a 1:1 mixture of branched and linear products in the hydrosilylation of 1-phenyl-buta-1,3-dienes^45^, catalyst **2c** produced 1,2-addition products with exclusive Markovnikov selectivity (**14**). Again, the substituents on the benzene ring of the 1-aryl-buta-1,3-diene substrates had little effect on the reaction outcome; high yields (91–95%) of Markovnikov products were observed for all the examined substrates (**13a**–**13f**). The hydrosilylation of 1,1-disubstituted buta-1,3-dienes **13g**–**13i** also proceeded smoothly to give 1,2-addition products with exclusive Markovnikov selectivity.

**Fig. 3:**
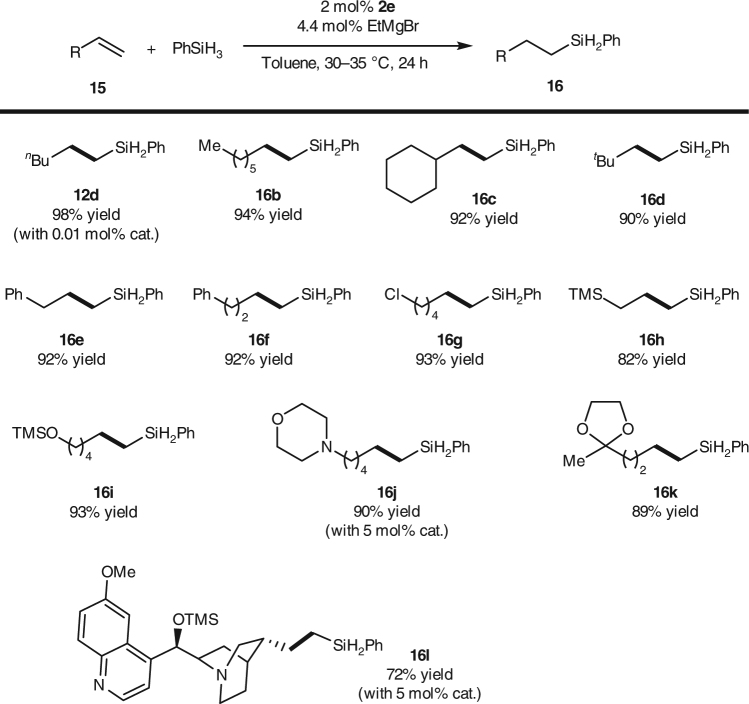
Iron-catalyzed hydrosilylation of 1-alkyl ethylenes. Exclusive linear products were obtained with remarkable high turnover numbers (up to 9800). The reaction exhibits good tolerance to functional groups and to steric hindrance

Catalysts **2** also exhibited high activity for the hydrosilylation of 1-alkyl ethylenes **15**, but with anti-Markovnikov selectivity (Fig. 3). For example, the reaction of 1-hexene (**15a**) with phenylsilane in the presence of 0.01 mol% **2e** afforded linear hydrosilylated product **12d** exclusively in almost quantitative yield. Although the turnover frequency (223 h^−1^ at 1 h) is moderate comparing to the iron-bis(imino)pyridine catalysts developed by Chirik^6^ and Thomas^45^, the high turnover number (9800) for this reaction indicates that the iron catalyst has a relatively long life time. Functional groups, including chloro (**16g**), trimethylsilyl (**16h**), siloxy (**16i**), amino (**16j** and **16l**), ketal (**16k**), and quinoline (**16l**) can be tolerated in this reaction. Moreover, the catalyst also exhibits good tolerances to steric hindrance of the substrates: even highly steric hindered *tert*-butyl substituted ethylene (**15d**) can smoothly undergo the hydrosilylation and afford the desired product with satisfactory yield.

This iron-catalyzed hydrosilylation reaction has great potential synthetic utility (Supplementary Fig. 1). For instance, the hydride moiety of silane **8a** could readily be transformed to hydroxy, alkoxy, or fluoride in good yield. The silyl group of **8a** could also be directly transformed to other functional groups, such as hydroxyl and chloride. Moreover, the silane products also have potential applications as monomers for preparing polysilanes.

### Mechanistic studies

**Fig. 4:**
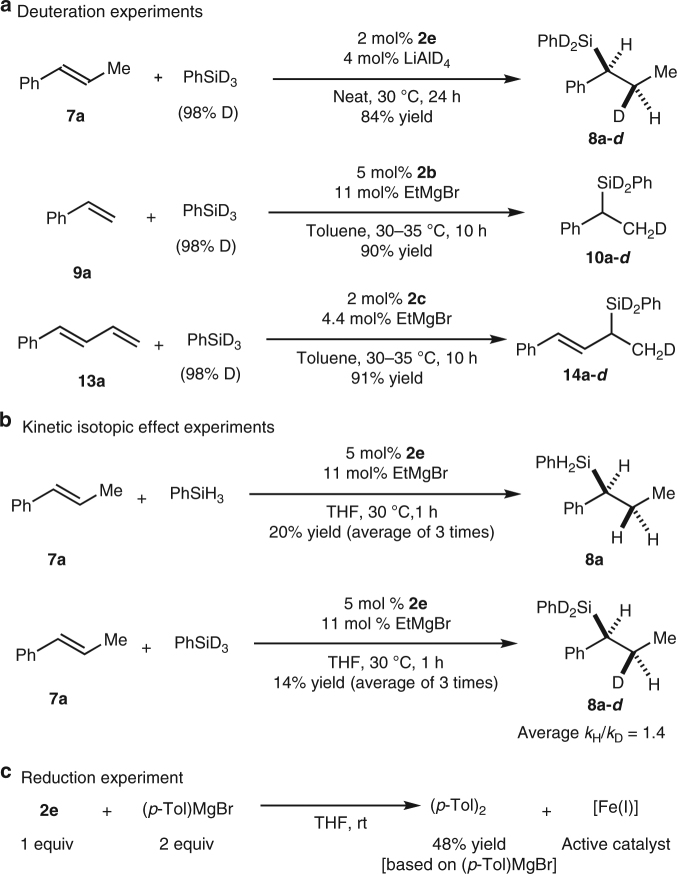
Control experiments. **a** Deuteration experiments. The D incorporation in the products are >95% in all cases according to ^1^H NMR. **b** Kinetic isotopic effect (KIE) experiments. A secondary KIE effect (average *k*_H_/*k*_D_ = 1.4) was observed. **c** Reduction experiment. The stoichiometric reduction experiment implies that a Fe(I) species is most likely generated as the active catalyst

We performed a set of deuteration experiments with alkenes **7a**, **9a**, and **13a** to gain some insight into the reaction mechanism (Fig. 4a). When deuterated silane PhSiD_3_ was used instead of PhSiH_3_, no obvious redistribution of the deuterium between the products and the recovered substrates was observed. These results differ from those observed for the hydrosilylation of styrene catalyzed by iron complexes with tridentate bis(imino)pyridine ligands, which shows extensive redistribution of deuterium in the products^45^. Notably, the deuteration experiment with internal alkene **7a** enabled us to uncover the details of the hydrogen transfer processes. The fact that the D and Si atoms in product **8a-d** were *cis* to each other (Supplementary Fig. 2) suggests that the addition of silane into the alkene substrate proceeded by means of an inner-sphere coplanar pathway.

A set of kinetic isotopic effect (KIE) experiments were performed through parallel reactions and a secondary KIE effect (*k*_H_/*k*_D_ = 1.4) was observed, which indicate that the C–H bond formation is not a rate-determining step (Fig. 4b and Supplementary Table 5).

Although the efforts on the isolation of single crystal of the active catalysts were unsuccessful, we performed a stoichiometric reduction experiment to determine the oxidation state of the active iron catalyst. When (*p*-Tol)MgBr was used as reductant, 4,4′-dimethyl-1,1′-biphenyl (*p*-Tol)_2_ was formed with 48% yield (based on the Grignard reagent) after quenching the reaction mixture. This experiment implies that a Fe(I) species is most likely generated^45^. The active iron catalyst prepared through this method exhibited the same reactivity for the hydrosilylaion reaction (Table 1, entry 9). Quite recently, Ritter and co-workers^46^ fully characterized a Fe(I) complex modified with a biimine ligand prepared through the reduction of corresponding Fe(II) complexes using the Grignard reagent. Considering the similarity of Ritter’s procedure and ours, it is reasonable to deduce that the Fe(I) complex is responsible for the catalytic cycle and play as the real catalyst in this study. We performed an electron paramagnetic resonance (EPR) analysis of the active iron catalyst generated from Fe(II) and EtMgBr. A strong signal was observed at 3375G (Supplementary Fig. 3), which implies that a high-spin iron species is detected.

**Fig. 5:**
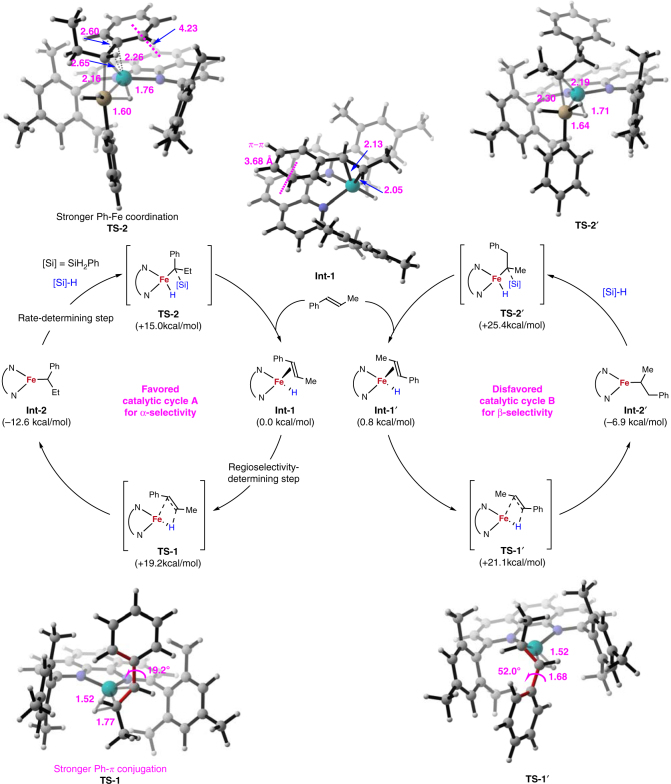
Computation studies on the mechanism. The catalytic cycles are based on high-spin Fe(I) catalysts. The migratory insertion of Fe-H to alkene is irreversible and determines the regioselectivity. The Si migration is the rate-determining step and passes through four-membered transition state. The benzylic selectivity (α-selectivity) initiates from *π*–*π* interaction between the phenyl of the alkene and the phenanthroline of the ligand

According to the above control experiments, we proposed two Fe(I)-catalyzed cycles, which lead to different regioselectivities via hydride insertion (**TS-1** or **TS-1′**) and direct Si migration (**TS-2** or **TS-2′**) steps (Fig. 5). DFT calculations were introduced to investigate this proposed mechanism in both high-spin (*S* = 3/2) and low-spin (*S* = 1/2) states at the (dispersion-corrected) unrestricted ωB97XD/6-31G*/TZVP level of theory with CPCM-UωB97XD/def2-TZVPP single-point energy calculations (see Supplementary Tables 6–27 and Supplementary Fig. 4 for details). The Fe(I) complex in low-spin state is ruled out because the calculated energy is more than 22 kcal/mol higher than that in the high-spin Fe(I). This result can be supported by the EPR experiment. The calculations also indicate all the *d* orbitals are occupied and only 4*s*/4*p* orbitals are available for coordination in the Fe(I) complex. The Fe(I)-H complex with β-methyl styrene (**7a**) coordination formed two distorted tetrahedral structures **Int-1** and **Int-1′** depending on the phenyl orientation of **7a** toward the phenanthroline ligand or not, which initiate two plausible catalytic cycles A and B. The *π–π* interaction between the phenyl of **7a** and phenanthroline ring of the catalyst (3.68 Å distance) in **Int-1** leads to favorable energy by 0.8 kcal/mol comparing to **Int-1′**. Hydride insertion into the alkyl end of the double bond is facile, via **TS-1** and **TS-1′** with an activation barrier 19.2 and 21.1 kcal/mol, respectively, forming the stable intermediates **Int-2** and **Int-2′**. The calculated **TS-1** and **TS-1′** were located to coplanar four-member-ring structures, suggesting an inner-sphere coplanar migration in agreement with the *cis*-substituent D and Si atoms of product **8a-d** from our deuteration experiment (Fig. 4a). This migratory insertion is predicted to be irreversible because the barrier in reverse direction is higher by 12.6 kcal/mol (total 31.8 kcal/mol for the benzylic selectivity pathway, catalytic cycle A), and therefore it determines the regioselectivity, which rationalize the experimental observation for no obvious redistribution of the deuterium (Fig. 4a). However, our kinetic isotope experiment (*k*_H_/*k*_D_ = 1.4) indicates that the hydride insertion is not rate-determining step. Our calculations predict that direct Si migration is responsible for rate-determining step with 27.6 kcal/mol barrier, and it proceeds via four-membered transition state, forming products and regenerating Fe(I) catalyst by **7a** coordination. The Fe-Ph coordination (Fe-C 2.26 Å) is enabled to stabilize **TS-2** and promotes the reaction affording benzylic selective product (Fig. 5). Furthermore, we extended our calculated mechanism to other substrates and the predicted *b*/*l* selectivities show good consistence with our experimental results (Table 5).

**Table 5:** Regioselectivity of iron-catalyzed alkene hydrosilylation


Entry	R	[Fe]	Exp. (*b*/*l*)	Δ*G*_cal(*b*−*l*)_ (kcal/mol)	Calc. (*b*/*l*)
1	Ph	**2b**	>98:2	−4.4	>99:1
2	Ph	**2c**	95:5	−1.5	93:7
3	c-Hex	**2e**	1:>99	+5.9	1:>99

## Discussion

In summary, we developed iron complex catalysts that have 2,9-diaryl-1,10-phenanthroline ligands and exhibit benzylic selectivity in the hydrosilylation of internal alkenes. Reactions of styrenes and 1,3-dienes showed unique Markovnikov selectivity, and these newly developed catalysts also showed excellent activity for the reactions of aliphatic terminal alkenes. Control experiments clearly showed that the 1,10-phenanthroline scaffold of ligands **1** was the basis of the unusual reactivity and selectivity of the catalysts. The introduction of 1,10-phenanthroline as a ligand scaffold for base-metal-catalyzed alkene hydrosilylation has great potential for bringing about significant advances of this area. The unusual catalytic activity associated with the phenanthroline ligands reported herein will inspire the design of additional related catalysts.

## Methods

### General methods

See Supplementary Methods for further details.

### Typical procedure for hydrosilylation of 7a

In an argon-filled glovebox, a vial (4 mL) was charged with **7a** (2 mmol), PhSiH_3_ (2.2 mmol), and complex **2e** (0.04 mmol). The reaction mixture was stirred at room temperature (25‒35 ^o^C) for 1 min, then EtMgBr (1 M in THF, 88 μL, 0.088 mmol, 4.4 mol %) was added. After stirring for 24 h at 30 ^o^C, the vial was removed from the glovebox and the reaction mixture was concentrated by rotating evaporation. The residue was purified by flash chromatography (hexane) to afford the desired product **8a** (430.2 mg, 95 %). ^1^H NMR (400 MHz, CDCl_3_) *δ* 7.42–7.34 (m, 3H, Ar-H), 7.33–7.20 (m, 4H, Ar-H), 7.12 (t, *J = *7.3 Hz, 1H, Ar-H), 7.05 (d, *J = *7.3 Hz, 2H, Ar-H), 4.37–4.24 (m, 2H, Si-H), 2.40–2.31 (m, 1H, CH), 1.97–1.79 (m, 2 H, CH_2_), 0.90 (t, *J = *7.2 Hz, 3H, CH_3_); ^13^C NMR (101 MHz, CDCl_3_) *δ* 142.8 (1C, Ar-C), 135.7 (2C, Ar-C), 131.4 (1C, Ar-C), 129.7 (1C, Ar-C), 128.3 (2C, Ar-C), 127.9 (2C, Ar-C), 127.8 (2C, Ar-C), 125.0 (1C, Ar-C), 34.3(1C, CH), 24.5 (1C, CH_2_), 13.9 (1C, CH_3_); ^29^Si NMR (79 MHz, CDCl_3_) *δ* −20.5, −20.7. HRMS (EI) calcd for [M, C_15_H_18_Si]^+^: 226.1178; found 226.1180.

### Data availability

Additional data supporting the findings described in this manuscript are available in the Supplementary Information. For full characterization data of new compounds and experimental details, see Supplementary Methods. For the ^1^H and ^13^C NMR spectra of new compounds, see Supplementary Figs. 5–146. Metrical parameters for **2c** are available free of charge from the Cambridge Crystallographic Data Centre under reference numbers CCDC-1455690. All other data are available from the authors upon reasonable request.

## Electronic supplementary material

Supplementary Information
