# Supplementary material for: Ligands with 1,10-phenanthroline scaffold for highly regioselective iron-catalyzed alkene hydrosilylation
Source: Nat Commun. 2018 Jan 15;9:221. doi: 10.1038/s41467-017-02472-6 (PMC5768772; doi:10.1038/s41467-017-02472-6)
Supplement: Supplementary file 1 — Supplementary Information [file 41467_2017_2472_MOESM1_ESM.pdf]

Supplementary Methods

All manipulations were carried out using standard Schlenk, high-vacuum and glovebox techniques. THF, Et_2O, 1,4-dioxane, and toluene were distilled from sodium benzophenone ketyl prior to use. Iron(II) chloride (99.99%) was purchased from Sigma-Aldrich and used as received. All commercial available olefins were purchased from Sigma-Aldrich, Alfa Aesar, Acros, and TCI. All olefins are dried over $LiAlH_4$ or CaH_2 and distilled prior to use. Phenylsilane was purchased from Sigma-Aldrich or J&K Chemical, dried over $LiAlH_4$ and distilled prior to use. Melting points were measured on a RY−I apparatus and uncorrected. Infrared spectra were recorded on a Bruker Fourier transform spectrometric (FT-IR) and reported in wave number. High resolution mass spectrometric (HRMS) analyses spectrum was determined on an IonSpec FT-ICR mass spectrometer and Waters GCT Premier mass spectrometer. Trace metal contamination analysis of iron precatalysts by ICP-OES (spectro-blue) was performed using a X7 (Thermo Electron Corporation) instruments. Magnetic moment was measured on SQUID VSM (Quantum Design). ^{1}H NMR, ^{13}C NMR, ^{29}Si NMR, ^{19}F NMR spectra were recorded with a Bruker AV 400 spectrometer at 400 MHz (^{1}H NMR), 101 MHz (^{13}C NMR), 79 MHz (^{29}Si NMR), and 376 MHz (^{19}F NMR) in $CDCl_3$. Chemical shifts were reported in ppm down field from internal Me_4Si (^{1}H NMR) and $CDCl_3$ (^{13}C NMR). Gas Chromatography (GC) analysis were performed using a Hewlett Packard Model HP 7890 Series instruments equipped with an FID detector and a capillary column, HP-5 (Agilent Technologies, 30 m × 0.032 mm × 0.25 μm film thickness).

All stationary points were fully optimized at the density functional theory level in Gaussian 09[1], using the unrestricted ωB97XD[2] functional without symmetry constraints. The triple-ζ valence basis set TZVP[3,4] were used for Fe, and the 6-31G(d) basis set was used for H, C, N and Si. The energies were further evaluated using a larger basis set def2-TZVPP[5,6] for all atoms involving the solvation effect with an implicit description of toluene using the CPCM treatment[7,8], where the United Atom Topological Model (UAHF) was used to define the solute cavity. For selected key steps of energy profile

were checked by M06/def2-TZVPP[9] and SMD solvent correction[10] at single point energy calculations. All optimized species were verified as either minima or transition structures by the presence of zero or a single imaginary vibrational frequency. Saddle points were connected to minima in the usual way with intrinsic reaction coordinate (IRC) calculations[11,12]. Computed structures are displayed with CYLview[13].

Synthesis of 2,9-diaryl-1,10-phenanthrolines

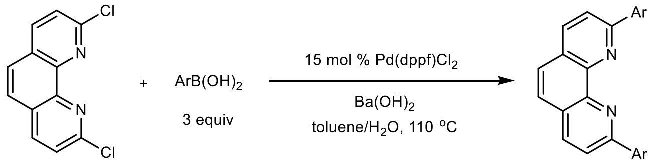

1 (Darphen)

A suspension of 2,9-dichloro-1,10-phenanthroline (747 mg, 3 mmol), which can be obtained according to reported procedure,[14] Pd(dppf)Cl_2 (329 mg, 0.45 mmol), $Ba(OH)_2 \cdot 8H_2O$ (4.74 g, 15 mmol) and $ArB(OH)_2$ (9 mmol) in a mixture of toluene (100 mL) and water (5 mL) was purged with N_2 to degas. The mixture was heated up to 110 ˚C and the reaction was monitored by TLC. After cooling to room temperature, the mixture was neutralized with saturated NH_4Cl (aq., 50 mL) and extracted with CH_2Cl_2 (100 mL × 3). The combined organic layer was dried over anhydrous $MgSO_4$ and concentrated under reduced pressure. The residue was purified by silica-gel column chromatography and recrystallization in *n*-hexane/CH_2Cl_2 [20:1(v/v)] to obtain 2,9-diaryl-1,10-phenanthrolines.

2,9-Diphenyl-1,10-phenanthroline (1a)[15]

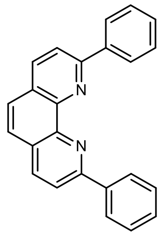

White powder, m.p. = 185−186 ºC (recrystallization from dichlromathane and hexane), 90% yield (898 mg). ^{1}H NMR (400 MHz, $CDCl_3$) δ 8.47 (d, *J* = 7.4 Hz, 4H, Ar-H), 8.31 (d, *J* = 8.1 Hz, 2H, 4,7-H), 8.15 (d, *J* = 8.3 Hz, 2H, 3,8-H), 7.79 (s, 2H, 5,6-H), 7.60 (t, *J* = 7.3 Hz, 4H, Ar-H), 7.50 (t, *J* = 7.1 Hz, 2H, Ar-H). ^{13}C NMR (101 MHz,

CDCl3) δ 156.8, 146.1, 139.5, 136.9, 129.4, 128.8, 127.9, 127.7 , 126.0, 120.0.

2,9-Bis(3,5-di-*tert*-butylphenyl)-1,10-phenanthroline (1b)

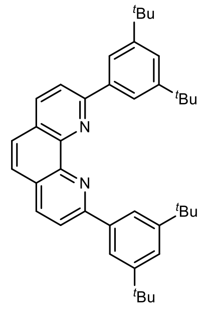

White powder, m.p. = 268 ºC (recrystallization from dichlromathane and hexane), 90% yield (1.50 g). ^{1}H NMR (400 MHz, $CDCl_3$) δ 8.30 (d, *J* = 8.4 Hz, 2H, 4,7-H), 8.07 (m, 6H), 7.80 (s, 2H, 5,6-H), 7.55 (t, *J* = 1.7 Hz, 2H), 1.44 (s, 36H, CH_3); ^{13}C NMR (101 MHz, $CDCl_3$) δ 159.2, 151.0, 146.4, 140.0, 136.7, 127.8, 126.0, 123.2, 122.6, 121.5, 35.0, 31.7. IR(neat): 3749w, 3446w, 2962s, 2903m, 2867m, 1598w, 1542w, 1505m, 1457m, 1362w, 1248w, 845m, 720m cm^{-1}. HRMS (ESI) calcd for $[M+H, C_{40}H_{49}N_2]^+$: 557.3896; found 557.3899.

2,9-Bis(2,4,6-trimethylphenyl)-1,10-phenanthroline (1c)[16]

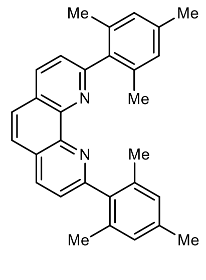

White powder, m.p. = 327−328 ºC (recrystallization from dichlromathane and hexane), 91% yield (1.14 g). ^{1}H NMR (400 MHz, $CDCl_3$) δ 8.28 (d, *J* = 8.2 Hz, 2H, 4,7-H), 7.85 (s, 2H, 5,6-H), 7.58 (d, *J* = 8.2 Hz, 2H, 3,8-H), 6.92 (s, 4H, Ar-H), 2.31 (s, 6H, CH_3), 2.15 (s, 12H, CH_3); ^{13}C NMR (101 MHz, $CDCl_3$) δ 160.1, 146.2, 138.1, 137.4, 136.3, 135.6, 128.5, 127.1, 126.1, 125.0, 21.1, 20.6. HRMS (EI) calcd for $[M, C_{30}H_{28}N_2]^+$: 416.2252; found 416.2249.

2,9-Bis(2,4,6-triethylphenyl)-1,10-phenanthroline (1d)

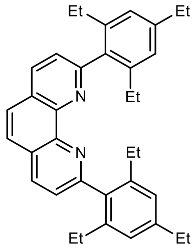

White powder, m.p. = 174−175 °C (recrystallization from dichlromathane and hexane), 95% yield (1.43 g). ^{1}H NMR (400 MHz, $CDCl_3$) δ 8.27 (d, *J* = 8.2 Hz, 2H, 4,7-H), 7.87 (s, 2H, 5,6-H), 7.59 (d, *J* = 8.1 Hz, 2H, 3,8-H), 6.96 (s, 4H, Ar-H), 2.64 (q, *J* = 7.4 Hz, 4H, CH_2), 2.50−2.30 (m, 8H, CH_2), 1.25 (t, *J* = 7.6 Hz, 6H, CH_3), 1.00−1.08 (m, 12H, CH_3); ^{13}C NMR (101 MHz, $CDCl_3$) δ 160.2, 146.3, 144.0, 142.1, 138.0, 135.4, 127.3, 126.2, 125.3, 125.0, 28.8, 26.8, 15.8, 15.3; IR(neat): 2962s, 2929s, 2867s, 1607s, 1583s, 1538m, 1492s, 1468s, 1352m, 1097m, 910m, 872s, 852s, 747w cm^{-1}; HRMS (ESI) calcd for [M+H, $C_{36}H_{41}N_2$]$^+$: 501.3270; found 501.3266.

2,9-Bis(2,4,6-triisopropylphenyl)-1,10-phenanthroline (1e)

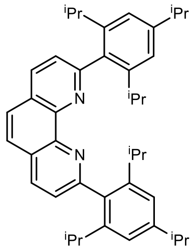

White powder, m.p. = 252−254 °C (recrystallization from dichlromathane and hexane), 93% yield (1.63 g). ^{1}H NMR (400 MHz, $CDCl_3$) δ 8.26 (d, *J* = 8.1 Hz, 2H, 4,7-H), 7.88 (s, 2H, 5,6-H), 7.59 (d, *J* = 8.1 Hz, 2H, 3,8-H), 7.05 (s, 4H, Ar-H), 2.92 (heptet, *J* = 6.9 Hz, 2H, CH), 2.50 (heptet, *J* = 6.9 Hz, 4H, CH), 1.28 (d, *J* = 6.9 Hz, 6H, CH_3), 1.07 (d, *J* = 6.8 Hz, 6H, CH_3), 1.06 (d, *J* = 6.9 Hz, 6H, CH_3); ^{13}C NMR (101 MHz, $CDCl_3$) δ 160.6, 148.6, 146.3, 146.1, 137.0, 135.2, 127.2, 126.2, 125.0, 120.6, 34.4, 30.5 24.1, 24.0 ; IR(neat): 3447w, 2961s, 2927m, 2868m, 1606w, 1505w, 1472w, 1361w,, 1100w, 860m cm^{-1}. HRMS (ESI) calcd for [M+H, $C_{42}H_{53}N_2$]$^+$: 585.4209; found 585.4208.

Synthesis and analytical data of Darphen-Fe

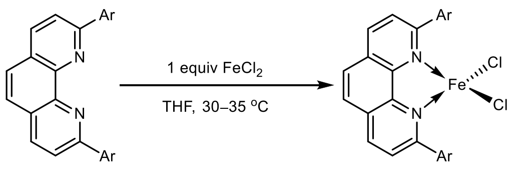

1 (Darphen) **2** (Darphen-Fe)

In an argon-filled glovebox, a Schlenk flask (50 mL) was charged with 2,9-diaryl-1,10 phenanthrolines **1** (2 mmol), $FeCl_2$ (253.5 mg, 2 mmol) and dry THF (20 mL). The reaction mixture was stirred at room temperature for 24 h. The solvent was partially removed under vacuum (about 5 mL left), then dry *n*-hexane (15 mL) was added, and solids precipitated. The product was collected by filtration, washed with 20 mL *n*-hexane, and dried under vacuum.

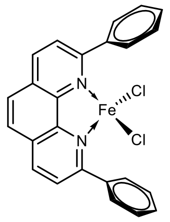

2a, yellow powder, 88% yield (808.3 mg), decomposition temperature 280−282 °C (recrystallization from tetrahydrofuran and hexane), ^{1}H NMR (400 MHz, $CDCl_3$) δ 58.38, 27.13, 2.38, 0.77, 1.26, -15.29. IR(neat) 3645w, 3301m, 3207m, 1728w, 1584m, 1548m, 1507m, 1446s, 1420m, 1356m, 1322m, 741s, 700s cm^{-1}.

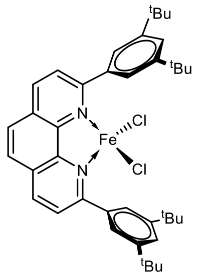

2b, orange powder, 81% yield (1.11 g), decomposition temperature 286−288 °C (recrystallization from tetrahydrofuran and hexane), ^{1}H NMR (400 MHz, $CDCl_3$) δ 58.72 (s, 2H), 3.55 (s, 2H), -0.11 (s, 36H), -13.71 (s, 2H), -20.75 (s, 2H). IR(neat): 3524w, 3436w, 3306s, 3210s, 3961s, 2905m, 1667w, 1629s, 1552m, 1496m, 1416w, 1363w, 1250w, 867m, 754m, 712m cm^{-1}. Anal. calcd. for $C_{40}H_{48}Cl_2FeN_2$: C, 70.28; H, 7.08; N, 4.10. found: C, 70.42; H, 7.14; N, 4.06. Magnetic moment χ_M=3.66 cm^{3}mol$^-$

^{1}K.

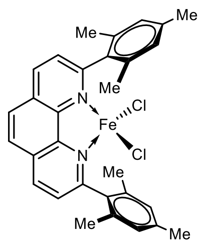

2c, Orange powder, 88% yield (956 mg), decomposition temperature 308–312 ºC (recrystallization from tetrahydrofuran and hexane), ^{1}H NMR (400 MHz, $CDCl_3$) δ 53.01 (s, 2H), 27.89 (s, 2H), 1.63 (s, 4H), 0.73 (s, 6H), -10.91 (s, 12H), -16.82 (s, 2H). IR(neat): 3666w, 3523w, 2919m, 1746s, 1612m, 1588s, 1556m, 1511s, 1481s, 1445m, 1426m, 1031m, 897s, 865m, 760w, 729w cm^{-1}. Anal. calcd. for $C_{30}H_{28}Cl_2FeN_2$ C, 66.32; H, 5.19; N, 5.16 found: C, 66.42; H, 5.22; N, 5.14. Magnetic moment χ_M=3.43 cm^{3}mol^{-1}K

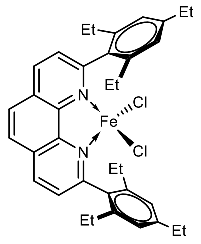

2d, orange powder, 83% yield (1.04 g), decomposition temperature 282−286 ºC (recrystallization from tetrahydrofuran and hexane), ^{1}H NMR (400 MHz, $CDCl_3$) δ 53.85 (s, 2H), 27.88 (s, 2H), 3.76 (s, 2H), 1.87 (s, 2H), 1.10 (s, 4H), 0.27 (s, 6H), -3.58 (s, 12H), -10.77 (s, 4H), -12.86 (s, 4H), -16.74 (s, 2H). IR(neat): 3555m, 3481s, 3416s, 3237w, 2967w, 2933, 2033w, 1639m, 1618m, 1555w, 1496w, 917w, 867w, 760w, 624.6 cm^{-1}.

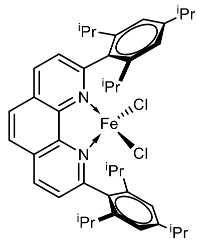

2e, red powder, 82% yield (1.17 g), decomposition temperature 270−274 ºC (recrystallization from tetrahydrofuran and hexane), ^{1}H NMR (400 MHz, $CDCl_3$) δ 54.62 (s, 2H), 29.46 (s, 2H), 2.43 (s, 4H), 2.04 (s, 2H), 1.26 (s, 12H), -6.0~-4.0 (m,

24H), -7.77 (s, 2H), -20.25 (s, 2H). IR(neat): 3555m, 3481s, 3416s, 3237w, 2967w, 2933, 2033w, 1639m, 1618m, 1555w, 1496w, 917w, 867w, 760w, 624.6 cm^{-1}. Anal. calcd. for $C_{42}H_{52}Cl_2FeN_2$ C, 70.89; H, 7.37; N, 3.94; found: C, 70.98; H, 7.40; N, 3.90. Magnetic moment χ_M=3.36 $cm^3mol^{-1}K$.

Supplementary Table 1 | Crystal data and structure refinement for 2c

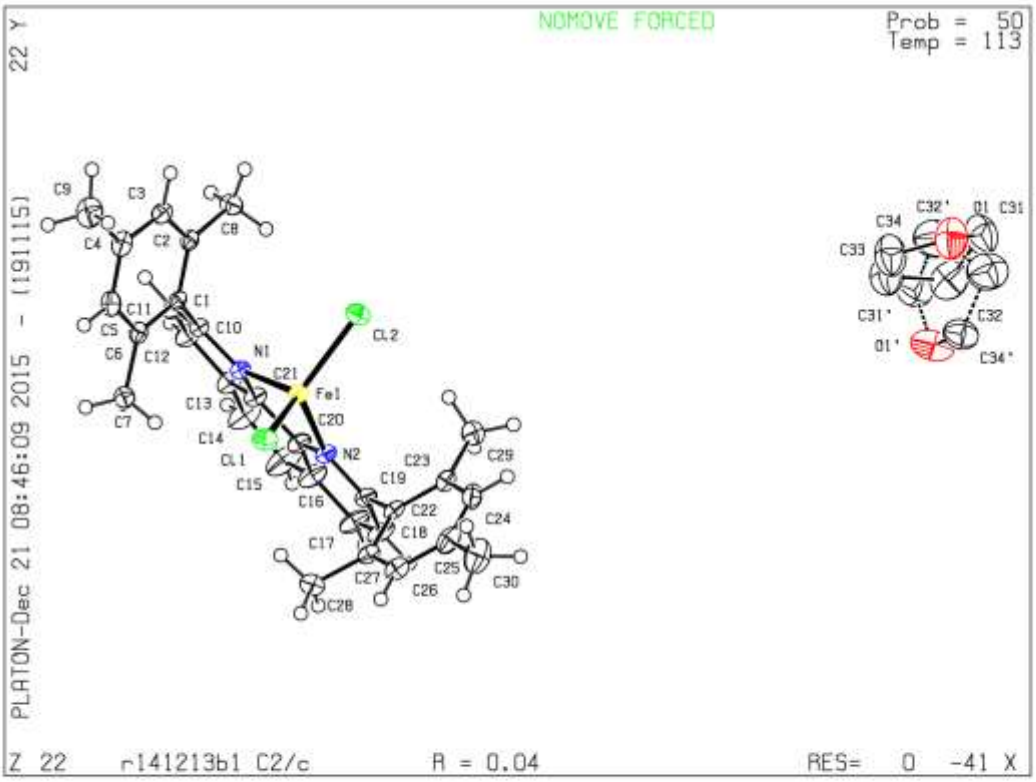

Empirical formula	C34 H28 Cl2 Fe N2 O
Moiety formula	$C_{20}H_{23}NO_3$
Formula weight	607.33
Temperature	113(2) K
Wavelength	0.71073 Å
Crystal system	Monoclinic, C2/c
Space group	P2(1)2(1)2(1)
Unit cell dimensions	a = 24.683(5) Å alpha = 90°
	b = 16.804(3) Å beta = 129.62(3)°
	c = 19.310(4) Å gamma = 90°
Volume	6170(2) $Å^3$

Z	4
Calculated density	8, 1.308 Mg/m^3
Absorption coefficient	0.691 mm^{-1}
F(000)	2512
Crystal size	0.20 x 0.18 x 0.12 mm
Theta range for data collection	1.62 to 27.91°
Limiting indices	-29<=h<=32, -22<=k<=20, -25<=l<=21
Reflections collected / unique	30576 / 7374 [R(int) = 0.0349]
Completeness to theta = 27.91	99.8 %
Absorption correction	Semi-empirical from equivalents
Max. and min. transmission	0.9217 and 0.8742
Refinement method	Full-matrix least-squares on F^2
Data / restraints / parameters	7374 / 180 / 413
Goodness-of-fit on F^2	1.086
Final R indices [I>2sigma(I)]	R_1 = 0.0445, wR_2 = 0.1180
R indices (all data)	R_1 = 0.0513, wR_2 = 0.1231
Largest diff. peak and hole	0.566 and -0.506 e.Å^{-3}

Typical procedure for synthesis of 13a-f

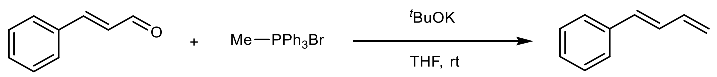

To a mixture of methyltriphenylphosphonium bromide (7.14 g, 20 mmol) and potassium *tert*-butanolate (2.47 g, 22 mmol), THF (100 mL) was added at 0 °C. The mixture was warmed to room temperature and stirred for 1 h. Then a solution of cinnamaldehyde (2.0 mL, 16 mmol) in THF (10 mL) was added and the resulting mixture was stirred for additional 12 h. A saturated solution of NH_4Cl (aq., 50 mL) was added and the mixture was extracted with Et_2O (3 × 100 mL). The combined organic phases were washed with saturated brine (100 mL), dried over Na_2SO_4, and the solvents were removed under reduced pressure. The residue was applied to a plug of silica, eluted with hexane, and the solvent was removed carefully under reduced pressure to obtain the desired product **13a** (1.89 g, 91%) as a colorless liquid.

(*E*)-Buta-1,3-dien-1-ylbenzene (13a)[17]

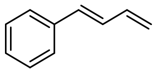

Colorless oil, 91% yield (1.90 g). ^{1}H NMR (400 MHz, $CDCl_3$) δ 7.45 (d, 2H, *J* = 7.3 Hz); 7.36 (t, 2H, *J* = 7.3 Hz); 7.27 (m, 1H); 6.84 (dd, 1H, *J* = 15.1, 10.3 Hz); 6.61 (d, 1H, *J* = 16.1 Hz); 6.56 (ddd, 1H, *J* = 17.3 Hz, 10.3, 10.3 Hz); 5.38 (d, 1H, *J* = 17.1 Hz); 5.22 (d, 1H, *J* = 9.8 Hz); ^{13}C NMR (101 MHz, $CDCl_3$) δ 137.2, 137.1, 132.8, 129.6, 128.6, 127.6, 126.4, 117.6.

(*E*)-1-(Buta-1,3-dien-1-yl)-4-methylbenzene (13b)[17]

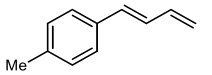

Colorless oil, 90% yield (2.08 g). ^{1}H NMR (400 MHz, $CDCl_3$) δ 7.30 (d, *J* = 8.1 Hz, 2H), 7.13 (d, *J* = 8.0 Hz, 2H), 6.75 (dd, *J* = 15.6, 10.5 Hz, 1H), 6.60−6.36 (m, 2H), 5.30 (d, *J* = 16.8 Hz, 1H), 5.14 (d, *J* = 10.0 Hz, 1H), 2.34 (s, 3H); ^{13}C NMR (101 MHz, $CDCl_3$) δ 137.5, 137.3, 134.3, 132.8, 129.3, 128.7, 126.3, 117.0, 21.2.

(*E*)-1-(Buta-1,3-dien-1-yl)-4-methoxybenzene (13c)[17]

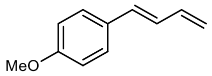

White solid, 80% yield (2.05 g). ^{1}H NMR (400 MHz, $CDCl_3$) δ 7.34 (d, *J* = 8.7 Hz, 2H), 6.86 (d, *J* = 8.8 Hz, 2H), 6.73−6.61 (m, 1H), 6.56−6.41 (m, 2H), 5.33−5.23 (m, 1H), 5.15−5.07 (m, 1H), 3.81 (s, 3H); ^{13}C NMR (101 MHz, $CDCl_3$) δ 159.3, 137.3, 132.4, 129.9, 127.6, 116.4, 114.0, 55.3.

(*E*)-4-(Buta-1,3-dien-1-yl)-N,N-dimethylaniline (13d)[18]

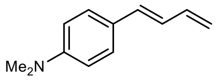

Slight yellow solid, 86% yield (2.38 g). ^{1}H NMR (400 MHz, $CDCl_3$) δ 7.36−7.31 (m, 2H), 6.72−6.62 (m, 3H), 6.58−6.47 (m, 2H), 5.30−5.23 (m, 1H), 5.08 (dd, *J* = 9.9, 1.3 Hz, 1H), 3.01 (s, 6H); ^{13}C NMR (101 MHz, $CDCl_3$) δ 150.0, 137.8, 133.1, 127.5, 125.5, 125.5, 114.9, 112.3, 40.4.

(*E*)-1-(Buta-1,3-dien-1-yl)-4-fluorobenzene (13e)[17]

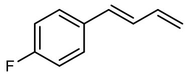

Colorless oil, 81% yield (1.92 g). ^{1}H NMR (400 MHz, $CDCl_3$) δ 7.40−7.32 (m, 2H), 7.06−6.96 (m, 2H), 6.70 (dd, *J* = 15.5, 10.6 Hz, 1H), 6.56−6.43 (m, 2H), 5.37−5.28 (m, 1H), 5.17 (d, *J* = 9.3 Hz, 1H); ^{13}C NMR (101 MHz, $CDCl_3$) δ 162.3 (d, *J* = 247.3 Hz), 137.0, 133.3 (d, *J* = 3.4 Hz), 131.6, 129.4 (d, *J* = 2.4 Hz), 127.9 (d, *J* = 7.9 Hz), 117.7, 115.5 (d, *J* = 21.7 Hz).

(*E*)-1-(Buta-1,3-dien-1-yl)-4-chlorobenzene (13f)[17]

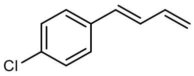

Colorless oil, 88% yield (2.31 g). ^{1}H NMR (400 MHz, $CDCl_3$) δ 7.31 (d, *J* = 8.6 Hz, 2H), 7.26 (d, *J* = 8.7 Hz, 2H), 6.74 (dd, *J* = 15.8, 10.3 Hz, 1H), 6.54−6.42 (m, 2H), 5.34 (d, *J* = 17.3 Hz, 1H), 5.19 (d, *J* = 10.0 Hz, 1H); ^{13}C NMR (101 MHz, $CDCl_3$) δ 136.8, 135.6, 133.1, 131.4, 130.1 128.7, 127.5, 118.2.

Synthesis of (*E*)-penta-2,4-dien-2-ylbenzene (13g)

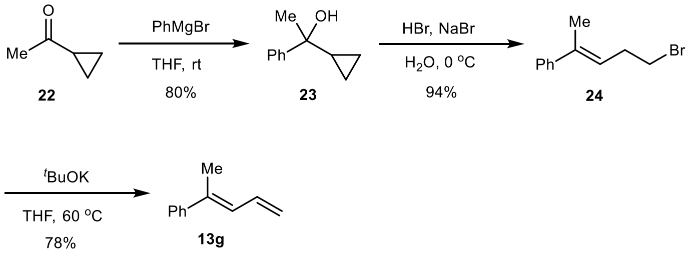

To a solution of cyclopropyl methyl ketone **22** (3.36 g, 40 mmol) in THF (70 mL) at 0 °C was added dropwise phenyl magnesium bromide (1.0 M in THF, 40 mL, 40 mmol). The reaction mixture was warmed to room temperature and stirred for farther 2 h. Saturated NH_4Cl (aq., 100 mL) was added to quench the reaction and the mixture was extracted with Et_2O (3 × 100 mL). The combined organic phases were washed with saturated brine (100 mL), dried over Na_2SO_4, and the solvents were removed under reduced pressure. The desired product **23** (5.19 g, 80%) was obtained by vacuum distillation.

To a emulsion of compound **23** (5.19 g, 32 mmol) and 1 M NaBr (aq., 40 mL, 40 mmol) was added 47% HBr (aq., 5.4 mL, 32 mmol) at 0 °C. The reaction was monitored by TLC. After completion of the reaction (about 2 h), the reaction mixture was diluted with water (40 mL) and extracted with ethyl acetate (3 × 50 mL). The combined organic

layers were dried over $MgSO_4$, filtered and concentrated under reduced pressure. The crude product was purified by column chromatography by using hexane as eluent and the solvent was removed carefully under reduced pressure to obtain the desired product **24** (6.77 g, 94%) as a colorless liquid.

Compound **24** (6.77 g, 30.1 mmol) was dissolved in 30 mL THF, and potassium *tert*-butanolate (5.07 g, 45.2 mmol) was added as solution in THF (20 mL) and stirred for 6 h at 60 °C. Saturated NH_4Cl (aq., 100 mL) was added and the mixture was extracted with Et_2O (3 × 50 mL). The combined organic phases were washed with saturated brine (100 mL), dried over Na_2SO_4, and the solvents were removed under reduced pressure. The crude product was purified by column chromatography by using hexane as eluent and the solvent was removed carefully under reduced pressure to obtain the desired product **13g** (3.37 g, 78%), a known compound[19] as a colorless liquid. ^{1}H NMR (400 MHz, $CDCl_3$) δ 7.45 (d, *J* = 7.8 Hz, 2H), 7.35−7.29 (m, 2H), 7.27−7.21 (m, 1H), 6.82−6.70 (m, 1H), 6.46 (d, *J* = 11.0 Hz, 1H), 5.32 (d, *J* = 16.7 Hz, 1H), 5.19 (d, *J* = 10.0 Hz, 1H), 2.17 (s, 3H); ^{13}C NMR (101 MHz, $CDCl_3$) δ 143.0, 136.7, 133.5, 132.7, 128.3, 127.7, 127.1, 125.7, 117.6, 16.0.

Synthesis of (*E*)-hexa-3,5-dien-3-ylbenzene (13h)

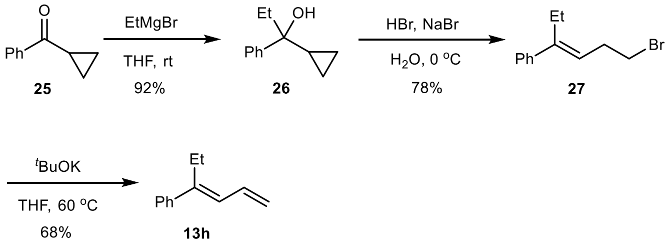

To a solution of cyclopropyl phenyl ketone **25** (5.85 g, 40 mmol) in THF (80 mL) at 0 °C was added dropwise ethyl magnesium bromide (1.0 M in THF, 40 mL, 40 mmol). The reaction mixture was warmed to room temperature and stirred for another 2 h. Saturated NH_4Cl (aq., 100 mL) was added to quench the reaction and the mixture was extracted with Et_2O (3 × 100 mL). The combined organic phases were washed with saturated brine (100 mL), dried over Na_2SO_4, and the solvents were removed under reduced pressure. The desired product **26** (6.49 g, 92%) was obtained by vacuum distillation.

To a emulsion of compound **26** (6.49 g, 36.8 mmol) and 1 M NaBr (aq., 40 mL, 40 mmol) was added 47% HBr (aq., 6.2 mL, 36.8 mmol) at 0 °C. The reaction was monitored by TLC. After completion of the reaction (about 3 h), the reaction mixture was diluted with water (40 mL) and extracted with ethyl acetate (3 × 50 mL). The combined organic layers were dried over $MgSO_4$, filtered and concentrated under reduced pressure. The crude product was purified by column chromatography by using hexane as eluent and the solvent was removed carefully under reduced pressure to obtain the desired product **27** (6.87 g, 78%) as a colorless liquid.

Compound **27** (6.87 g, 28.7 mmol) was dissolved in 30 mL THF, and potassium *tert*-butanolate (5.07 g, 45.2 mmol) was added as solution in THF (25 mL) and stirred for 6 h at 60 °C. Saturated NH_4Cl (aq., 100 mL) was added and the mixture was extracted with Et_2O (3 × 50 mL). The combined organic phases were washed with saturated brine (100 mL), dried over Na_2SO_4, and the solvents were removed under reduced pressure. The crude product was purified by column chromatography by using hexane as eluent and the solvent was removed carefully under reduced pressure to obtain the desired product **13h** (3.09 g, 68%.) as a colorless liquid. ^{1}H NMR (400 MHz, $CDCl_3$) δ 7.45−7.39 (m, 2H), 7.36−7.30 (m, 2H), 7.28−7.25 (m, 1H), 6.76 (ddd, *J* = 16.7, 10.9, 10.2 Hz, 1H), 6.34 (d, *J* = 11.1 Hz, 1H), 5.32 (dd, *J* = 16.7, 1.3 Hz, 1H), 5.19 (dd, *J* = 10.2, 1.7 Hz, 1H), 2.66 (q, *J* = 7.5 Hz, 2H), 1.05 (t, *J* = 7.6 Hz, 3H); ^{13}C NMR (101 MHz, $CDCl_3$) δ 143.7, 142.0, 133.3, 128.3, 127.3, 127.1, 126.2, 117.7, 23.2, 14.0.

Synthesis of buta-1,3-diene-1,1-diyldibenzene (13i)

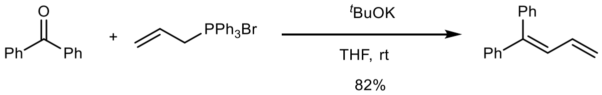

To a mixture of allyltriphenylphosphonium bromide (7.67 g, 20 mmol) and potassium *tert*-butanolate (2.47 g, 22 mmol), THF(100 mL) was added at 0 °C. The mixture was warmed to room temperature and stirred for 1 h. Then benzophenone (2.92 g, 16 mmol) was added as solution in THF (10 mL) and stirred for additional 12 h. A saturated NH_4Cl (aq., 50 mL) was added and the mixture was extracted with Et_2O (3 × 100 mL). The combined organic phases were washed with saturated brine (100 mL), dried over

anhydrous Na_2SO_4, and the solvents were removed under reduced pressure. The residue was applied to a plug of silica, eluted with hexane, and the solvent was removed carefully under reduced pressure to obtain the desired product **13i** (2.71 g, 82%), a known compound.[20] Colorless oil. ^{1}H NMR (400 MHz, $CDCl_3$) δ 7.42−7.18 (m, 10H, Ar-H), 6.72 (d, J = 11.0 Hz, 1H), 6.44 (dt, J = 16.9, 10.5 Hz, 1H), 5.39 (dd, J = 16.8, 1.1 Hz, 1H), 5.13 (d, J = 10.1 Hz, 1H); ^{13}C NMR (101 MHz, $CDCl_3$) δ 143.1, 142.1, 139.6, 134.9, 130.4, 128.5, 128.2, 128.1, 127.6, 127.5, 127.4, 118.6.

Synthesis of phenyl(silane-*d*3)

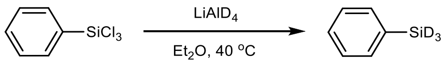

Phenyltrichlorosilane (4.23 g, 20 mmol) was added dropwise to a stirred suspension of lithium aluminium deuteride (1.68 g, 40 mmol, 98 atom % D) in anhydrous diethyl ether (30 mL) under nitrogen atmosphere. The reaction was heated at reflux for 24 hours, cooled to room temperature and ^{iso}propanol (0.5 mL) was added slowly, followed by water (5 mL) and saturated NH_4Cl aqueous (aq., 50 mL). The mixture was filtered through a short column of celite, which was washed with diethyl ether (50 mL). The aqueous layer was separated, and extracted with diethyl ether (2 × 30 mL). The organic fractions were combined, dried over anhydrous $MgSO_4$ and concentrated under reduced pressure to give a mixture of oil and colorless precipitates. Pentane (25 mL) was added and the suspension was filtered through a short column of celite, which was washed with pentane (50 mL). The filtrate was dried ($MgSO_4$) and concentrated under reduced pressure to give phenyl(silane-d3) as colourless oil (1.49 g, 13.4 mmol, 67%, 98 % D). ^{1}H NMR (400 MHz, $CDCl_3$) δ 7.59 (d, J = 6.7 Hz, 2H), 7.43−7.32 (m, 3H), 4.18 (s, 0.04H); ^{13}C NMR (101 MHz, $CDCl_3$) δ 135.8, 129.8, 128.2, 128.1.

Typical procedure for hydrosilylation of *β*-alkyl styrenes

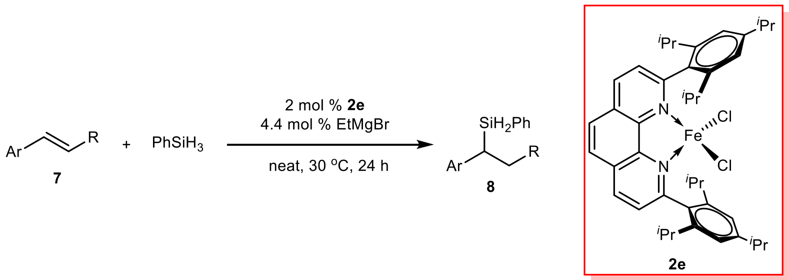

In an argon-filled glovebox, a vial (4 mL) was charged with alkene (2 mmol), silane (2.2 mmol), and complex **2e** (0.04 mmol). The reaction mixture was stirred at room temperature (25–35 °C) for 1 minute, then EtMgBr (1 M in THF, 88 μL, 0.088 mmol, 4.4 mol %) was added. After stirring for 24 hours at 30 °C, the vial was removed from the glovebox and the reaction mixture was concentrated by rotating evaporation. The residue was purified by flash chromatography to afford the desired product.

Phenyl(1-phenylpropyl)silane (8a)

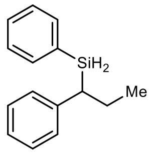

95% yield (430.2 mg), colorless oil. ^{1}H NMR (400 MHz, $CDCl_3$) δ 7.42−7.34 (m, 3H, Ar-H), 7.33−7.20 (m, 4H, Ar-H), 7.12 (t, J = 7.3 Hz, 1H, Ar-H), 7.05 (d, J = 7.3 Hz, 2H, Ar-H), 4.37−4.24 (m, 2H, Si-H), 2.40−2.31 (m, 1H, CH), 1.97−1.79 (m, 2H, CH_2), 0.90 (t, J = 7.2 Hz, 3H, CH_3); ^{13}C NMR (101 MHz, $CDCl_3$) δ 142.8 (1C, Ar-C), 135.7 (2C, Ar-C), 131.4 (1C, Ar-C), 129.7 (1C, Ar-C), 128.3 (2C, Ar-C), 127.9 (2C, Ar-C), 127.8 (2C, Ar-C), 125.0 (1C, Ar-C), 34.3(1C, CH), 24.5 (1C, CH_2), 13.9 (1C, CH_3); ^{29}Si NMR (79 MHz, $CDCl_3$) δ -20.5, -20.7. HRMS (EI) calcd for [M, $C_{15}H_{18}Si$]$^+$: 226.1178; found 226.1180.

Phenyl(1-phenylbutyl)silane (8b)

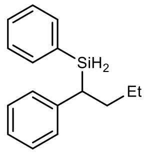

93% yield (447.1 mg), colorless oil. ^{1}H NMR (400 MHz, $CDCl_3$) δ 7.40−7.34 (m, 3H, Ar-H), 7.32−7.19 (m, 4H, Ar-H), 7.10 (t, J = 7.3 Hz, 1H, Ar-H), 7.07−7.02 (m, 2H, Ar-H), 4.37−4.23 (m, 2H, Si-H), 2.52−2.43 (m, 1H, CH), 1.92−1.72 (m, 2H, CH_2), 1.42−1.17 (m, 2H, CH_2), 0.84 (t, J = 7.3 Hz, 3H, CH_3); ^{13}C NMR (101 MHz, $CDCl_3$) δ 142.9 (1C, Ar-C), 135.7 (2C, Ar-C), 131.5 (1C, Ar-C), 129.7 (1C, Ar-C), 128.3 (2C, Ar-C), 127.9 (2C, Ar-C), 127.8 (2C, Ar-C), 125.0 (1C, Ar-C), 33.5 (1C, CH_2), 32.0 (1C, CH_2), 22.1 (1C, CH) 13.8 (1C, CH_3); ^{29}Si NMR (79 MHz, $CDCl_3$) δ -23.2. HRMS (EI) calcd for [M, $C_{16}H_{20}Si$]$^+$: 240.1334; found 240.1335.

Phenyl(1-phenylpentyl)silane (8c)

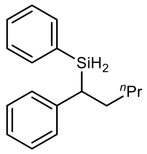

92% yield (468.1 mg), colorless oil. ^{1}H NMR (400 MHz, $CDCl_3$) δ 7.39−7.34 (m, 3H, Ar-H), 7.32−7.19 (m, 4H, Ar-H), 7.10 (t, *J* = 7.3 Hz, 1H, Ar-H), 7.04 (d, *J* = 7.3 Hz, 2H, Ar-H), 4.38−4.28 (m, 2H, Si-H), 2.50−2.39 (m, 1H, CH), 1.89−1.77 (m, 2H, CH_2), 1.35−1.17 (m, 4H, CH_2CH_2), 0.84−0.78 (m, 3H, CH_3); ^{13}C NMR (101 MHz, $CDCl_3$) δ 143.0 (1C, Ar-C), 135.7 (2C, Ar-C), 131.5 (1C, Ar-C), 129.6 (1C, Ar-C), 128.3 (2C, Ar-C), 127.9 (2C, Ar-C), 127.8 (2C, Ar-C), 125.0 (1C, Ar-C), 32.2 (1C, CH_2), 31.2 (1C, CH_2), 31.0 (1C, CH_2), 22.5 (1C, CH) 13.9 (1C, CH_3); ^{29}Si NMR (79 MHz, $CDCl_3$) δ -23.6. HRMS (EI) calcd for [M, $C_{17}H_{22}Si]^+$: 254.1491; found 254.1488.

Phenyl(1-phenylhexyl)silane (8d)

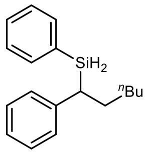

93% yield (499.4 mg), colorless oil. ^{1}H NMR (400 MHz, $CDCl_3$) δ 7.39−7.34 (m, 3H, Ar-H), 7.32−7.19 (m, 4H, Ar-H), 7.14−7.08 (m, 1H, Ar-H), 7.06−7.02 (m, 2H, Ar-H), 4.34 (dd, *J* = 6.6, 3.1 Hz, 1H, Si-H), 4.31 (dd, *J* = 6.6, 3.3 Hz, 1H, Si-H), 2.50−2.41 (m, 1H, CH), 1.88−1.77 (m, 2H, CH_2), 1.13−1.15 (m, 6H, $CH_2CH_2CH_2$), 0.84−0.78 (m, 3H, CH_3); ^{13}C NMR (101 MHz, $CDCl_3$) δ 143.0 (1C, Ar-C), 135.7 (2C, Ar-C), 131.5 (1C, Ar-C), 129.6 (1C, Ar-C), 128.3 (2C, Ar-C), 127.9 (2C, Ar-C), 127.8 (2C, Ar-C), 125.0 (1C, Ar-C), 32.2 (1C, CH_2), 31.7 (1C, CH_2), 31.3 (1C, CH_2), 28.7 (1C, CH_2), 22.4 (1C, CH), 14.0 (1C, CH_3); ^{29}Si NMR (79 MHz, $CDCl_3$) δ -23.3. HRMS (EI) calcd for [M, $C_{18}H_{24}Si]^+$: 268.1647; found 268.1643.

(4-Methyl-1-phenylpentyl)(phenyl)silane (8e)

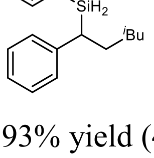

93% yield (499.5 mg), colorless oil. ^{1}H NMR (400 MHz, $CDCl_3$) δ 7.40−7.33 (m, 3H,

Ar-H), 7.31−7.19 (m, 4H, Ar-H), 7.11 (t, *J* = 7.3 Hz, 1H, Ar-H), 7.06−7.01 (m, 2H, Ar-H), 4.35 (dd, *J* = 6.6, 3.0 Hz, 1H, Si-H), 4.31 (dd, *J* = 6.6, 3.3 Hz, 1H, Si-H), 2.44−2.36 (m, 1H, CH), 1.88−1.80 (m, 2H, CH_2), 1.55−1.43 (m, 1H, CH), 1.23−1.09 (m, 2H, CH_2), 0.79−0.83 (m, 6H, CH_3); ^{13}C NMR (101 MHz, $CDCl_3$) δ 143.0 (1C, Ar-C), 135.7 (2C, Ar-C), 131.4 (1C, Ar-C), 129.7 (1C, Ar-C), 128.3 (2C, Ar-C), 127.8 (2C, Ar-C), 127.7 (2C, Ar-C), 125.0 (1C, Ar-C), 38.3 (1C, CH), 32.5 (1C, CH_2), 29.1 (1C, CH_2), 27.9 (1C, CH), 22.7 (1C, CH_3), 22.3 (1C, CH_3); ^{29}Si NMR (79 MHz, $CDCl_3$) δ -23.6. HRMS (EI) calcd for [M, $C_{18}H_{24}Si$]$^+$: 268.1647; found 268.1649.

(1,3-Diphenylpropyl)(phenyl)silane (8f)

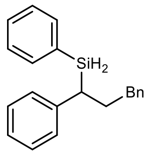

92% yield (556.5 mg), colorless oil. ^{1}H NMR (400 MHz, $CDCl_3$) δ 7.39−7.33 (m, 3H, Ar-H), 7.30−7.22 (m, 6H, Ar-H), 7.19−7.12 (m, 2H, Ar-H), 7.10−7.05 (m, 4H, Ar-H), 4.37−4.30 (m, 2H, Si-H), 2.70−2.60 (m, 1H, CH), 2.54−2.44 (m, 2H, CH_2), 2.25−2.08 (m, 2H, CH_2); ^{13}C NMR (101 MHz, $CDCl_3$) δ 142.3 (1C, Ar-C), 142.0 (1C, Ar-C), 135.7 (2C, Ar-C), 131.1 (1C, Ar-C), 129.7 (1C, Ar-C), 128.5 (2C, Ar-C), 128.5 (2C, Ar-C), 128.3 (2C, Ar-C), 127.9 (2C, Ar-C), 127.8 (2C, Ar-C), 125.8 (1C, Ar-C), 125.2 (1C, Ar-C), 34.9 (1C, CH_2), 33.0 (1C, CH), 31.6 (1C, CH_2); ^{29}Si NMR (79 MHz, $CDCl_3$) δ -23.0. HRMS (EI) calcd for [M, $C_{21}H_{22}Si$]$^+$: 302.1491; found 302.1493.

(3-Methyl-1-phenylbutyl)(phenyl)silane (8g)

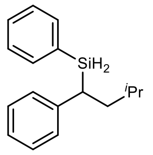

89% yield (452.8 mg), colorless oil. ^{1}H NMR (400 MHz, $CDCl_3$) δ 7.39−7.33 (m, 3H, Ar-H), 7.32−7.19 (m, 4H, Ar-H), 7.14−7.08 (m, 1H, Ar-H), 7.07−7.03 (m 2H, Ar-H), 4.33 (dd, *J* = 6.7, 3.1 Hz, 1H, Si-H), 4.29 (dd, *J* = 6.7, 3.3 Hz, 1H, Si-H), 2.63−2.56 (m, 1H, CH), 1.94−1.82 (m, 1H, CH), 1.58−1.45 (m, 2H, CH_2), 0.83−0.79 (m, 6H, CH_3); ^{13}C NMR (101 MHz, $CDCl_3$) δ 143.0 (1C, Ar-C), 135.7 (2C, Ar-C), 131.4 (1C, Ar-C), 129.7 (1C, Ar-C), 128.4 (2C, Ar-C), 127.9 (2C, Ar-C), 127.8 (2C, Ar-C), 125.0 (1C, Ar-

C), 40.2 (1C, CH), 29.9 (1C, CH_2), 26.1 (1C, CH_2), 23.5 (1C, CH_3) 21.2 (1C, CH_3); ^{29}Si NMR (79 MHz, $CDCl_3$) δ -23.4. HRMS (EI) calcd for [M, $C_{17}H_{22}Si$]$^+$: 254.1491; found 254.1488.

(2-Cyclopropyl-1-phenylethyl)(phenyl)silane (8h)

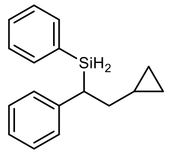

92% yield (464.4 mg), colorless oil. ^{1}H NMR (400 MHz, $CDCl_3$) δ 7.40−7.33 (m, 3H, Ar-H), 7.30−7.20 (m, 4H, Ar-H), 7.15−7.07 (m, 3H, Ar-H), 4.37 (dd, *J* = 6.6, 3.2 Hz, 1H, Si-H), 4.29 (dd, *J* = 6.6, 3.4 Hz, 1H, Si-H), 2.67−2.59 (m, 1H, CH), 1.99 (ddd, *J* = 14.3, 9.8, 5.7 Hz, 1H, CH_2), 1.49 (ddd, *J* = 13.9, 8.0, 5.7 Hz, 1H, CH_2), 0.70 (tdd, *J* = 8.0, 5.5, 3.0 Hz, 1H, CH), 0.38−0.31 (m, 2H, CH_2), 0.03−0.02 (m, 2H, CH_2); ^{13}C NMR (101 MHz, $CDCl_3$) δ 143.2 (1C, Ar-C), 135.6 (2C, Ar-C), 131.4 (1C, Ar-C), 129.6 (1C, Ar-C), 128.3 (2C, Ar-C), 128.0 (2C, Ar-C), 127.8 (2C, Ar-C), 125.0 (1C, Ar-C), 37.0 (1C, CH), 32.8 (1C, CH_2), 10.9 (1C, CH), 5.0 (1C, CH_2), 4.7 (1C, CH_2); ^{29}Si NMR (79 MHz, $CDCl_3$) δ -23.4. HRMS (EI) calcd for [M, $C_{17}H_{20}Si$]$^+$: 252.1334; found 254.1339.

(2-Cyclohexyl-1-phenylethyl)(phenyl)silane (8i)

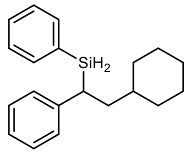

92% yield (541.9 mg), colorless oil. ^{1}H NMR (400 MHz, $CDCl_3$) δ 7.40−7.32 (m, 3H, Ar-H), 7.32−7.18 (m, 4H, Ar-H), 7.10 (t, *J* = 7.3 Hz, 1H, Ar-H), 7.04 (d, *J* = 7.2 Hz, 2H, Ar-H), 4.35−4.26 (m, 2H, Si-H), 2.68−2.59 (m, 1H, CH), 1.89−1.72 (m, 2H, CH_2), 1.66−1.53 (m, 5H), 1.30−1.00 (m, 4H), 0.94−0.70 (m, 2H); ^{13}C NMR (101 MHz, $CDCl_3$) δ 143.0 (1C, Ar-C), 135.7 (2C, Ar-C), 131.5 (1C, Ar-C), 129.6 (1C, Ar-C), 128.3 (2C, Ar-C), 127.9 (2C, Ar-C), 127.8 (2C, Ar-C), 124.9 (1C, Ar-C), 38.8 (1C, CH), 35.5 (1C, CH_2), 34.1 (1C, CH), 32.2 (1C, CH_2), 29.0 (1C, CH_2), 26.6 (1C, CH_2) 26.2 (1C, CH_2), 26.0 (1C, CH_2); ^{29}Si NMR (79 MHz, $CDCl_3$) δ -23.6. HRMS (EI) calcd for [M, $C_{20}H_{26}Si$]$^+$: 296.1804; found 296.1805.

(3,3-Dimethyl-1-phenylbutyl)(phenyl)silane (8j)

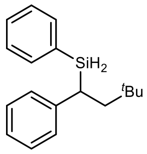

68% yield (365.2 mg), colorless oil. ^{1}H NMR (400 MHz, $CDCl_3$) δ 7.38−7.31 (m, 3H, Ar-H), 7.30−7.26 (m, 2H, Ar-H), 7.22−7.16 (m, 2H, Ar-H), 7.10−7.04 (m, 3H, Ar-H), 4.31 (dd, J = 6.9, 3.3 Hz, 1H, Si-H), 4.26 (dd, J = 6.9, 3.3 Hz, 1H, Si-H), 2.63−2.55 (m, 1H, CH), 1.98−1.89 (m, 1H, CH_2), 1.77−1.68 (m, 1H, CH_2), 0.76 (s, 9H, CH_3); ^{13}C NMR (101 MHz, $CDCl_3$) δ 144.6 (1C, Ar-C), 135.6 (2C, Ar-C), 131.6 (1C, Ar-C), 129.7 (1C, Ar-C), 128.3 (2C, Ar-C), 127.9 (2C, Ar-C), 127.7 (2C, Ar-C), 124.7 (1C, Ar-C), 44.8 (1C, CH_2), 32.8 (1C, C), 29.8 (3C, CH_3), 28.6 (1C, CH); ^{29}Si NMR (79 MHz, $CDCl_3$) δ -23.2. HRMS (EI) calcd for [M, $C_{18}H_{24}Si$]$^+$: 268.1647; found 268.1652.

Phenyl(1-(*o*-tolyl)hexyl)silane (8k)

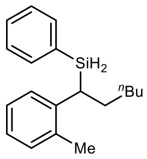

90% yield (508.5 mg), colorless oil. ^{1}H NMR (400 MHz, $CDCl_3$) δ 7.40−7.33 (m, 3H, Ar-H), 7.31−7.26 (m, 2H, Ar-H), 7.14 (t, J = 7.2 Hz, 1H, Ar-H), 7.08 (t, J = 6.3 Hz, 2H, Ar-H), 7.14 (t, J = 7.3 Hz, 1H, Ar-H), 4.32−4.24 (m, 2H, Si-H), 2.69−2.62 (m, 1H, CH), 2.14 (s, 3H, CH_3), 1.90−1.80 (m, 2H, CH_2), 1.33−1.15 (m, 6H, $CH_2CH_2CH_2$), 0.85−0.78 (m, 3H, CH_3); ^{13}C NMR (101 MHz, $CDCl_3$) δ 141.2 (1C, Ar-C), 135.6 (2C, Ar-C), 135.5(1C, Ar-C), 131.6 (1C, Ar-C), 130.1 (1C, Ar-C), 129.7 (1C, Ar-C), 127.8 (2C, Ar-C), 126.4 (1C, Ar-C), 126.1 (1C, Ar-C), 124.6 (1C, Ar-C), 31.8 (1C, CH_2), 31.5 (1C, CH_3), 28.8(1C, CH_2), 27.2 (1C, CH_2), 22.4(1C, CH_2), 20.2 (1C, CH), 14.0 (1C, CH_3); ^{29}Si NMR (79 MHz, $CDCl_3$) δ -23.8. HRMS (EI) calcd for [M, $C_{19}H_{26}Si$]$^+$: 282.1804; found 282.1808.

Phenyl(1-(*m*-tolyl)hexyl)silane (8l)

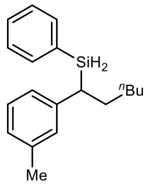

92% yield (519.2 mg), colorless oil. ^{1}H NMR (400 MHz, $CDCl_3$) δ 7.40−7.34 (m, 3H, Ar-H), 7.32−7.27 (m, 2H, Ar-H), 7.12 (t, *J* = 7.9 Hz, 1H, Ar-H), 6.93 (d, *J* = 7.4 Hz, 1H, Ar-H), 6.88−6.82 (m, 2H, Ar-H), 4.32 (dd, *J* = 6.7, 3.3 Hz, 1H, Si-H), 4.29 (dd, *J* = 6.7, 3.2 Hz, 1H, Si-H), 2.44−2.36 (m, 1H, CH), 2.28 (s, 3H, CH_3), 1.90−1.73 (m, 2H, CH_2), 1.35−1.15 (m, 6H, $CH_2CH_2CH_2$), 0.85−0.78 (m, 3H, CH_3); ^{13}C NMR (101 MHz, $CDCl_3$) δ 142.9 (1C, Ar-C), 137.8 (2C, Ar-C), 135.7 (2C, Ar-C), 131.6 (1C, Ar-C), 129.6 (1C, Ar-C), 128.7 (1C, Ar-C), 128.2 (1C, Ar-C), 127.7 (2C, Ar-C), 125.8 (1C, Ar-C), 124.8 (1C, Ar-C), 32.1 (1C, CH_2), 31.7 (1C, CH_3), 31.3 (1C, CH_2), 28.7 (1C, CH_2), 22.4 (1C, CH_2), 21.5 (1C, CH), 14.1 (1C, CH_3); ^{29}Si NMR (79 MHz, $CDCl_3$) δ -23.2. HRMS (EI) calcd for [M, $C_{19}H_{26}Si$]$^+$: 282.1804; found 282.1808.

Phenyl(1-(*p*-tolyl)hexyl)silane (8m)

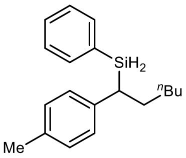

94% yield (531.1 mg), colorless oil. ^{1}H NMR (400 MHz, $CDCl_3$) δ 7.41−7.34 (m, 3H, Ar-H), 7.32−7.27 (m, 2H, Ar-H), 7.04 (d, *J* = 7.8 Hz, 2H, Ar-H), 6.95 (d, *J* = 7.9 Hz, 2H, Ar-H), 4.45−4.37 (m, 2H, Si-H), 2.50−2.39 (m, 1H, CH), 2.30 (s, 3H, CH_3), 1.87−1.73(m, 2H, CH_2), 1.35−1.14 (m, 6H, $CH_2CH_2CH_2$), 0.84−0.78 (m, 3H, CH_3); ^{13}C NMR (101 MHz, $CDCl_3$) δ 139.8 (1C, Ar-C), 135.7 (2C, Ar-C), 134.3 (1C, Ar-C), 131.7 (1C, Ar-C), 129.6 (1C, Ar-C), 129.1 (2C, Ar-C), 127.8 (2C, Ar-C), 127.7 (2C, Ar-C), 31.7 (1C, CH_2), 31.6 (1C, CH_2), 31.4 (1C, CH_3), 28.7 (1C, CH_2), 22.5 (1C, CH_2), 21.0 (1C, CH), 14.1 (1C, CH_3); ^{29}Si NMR (79 MHz, $CDCl_3$) δ -23.3. HRMS (EI) calcd for [M, $C_{19}H_{26}Si$]$^+$: 282.1804; found 282.1807.

(1-(2-Fluorophenyl)hexyl)(phenyl)silane (8n)

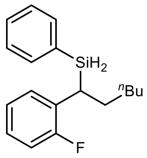

93% yield (532.9 mg), colorless oil. ^{1}H NMR (400 MHz, $CDCl_3$) δ 7.44−7.34 (m, 3H, Ar-H), 7.34−7.28 (m, 2H, Ar-H), 7.11−7.00 (m, 3H, Ar-H), 6.98−6.92 (m, 1H, Ar-H), 4.38−4.28 (m, 2H, Si-H), 2.86−2.77 (m, 1H, CH), 1.88−1.77 (m, 2H, CH_2), 1.34−1.15 (m, 6H, $CH_2CH_2CH_2$), 0.85−0.78 (m, 3H, CH_3); ^{13}C NMR (101 MHz, $CDCl_3$) δ 160.6 (d, 1C, J = 243.3 Hz, Ar-C), 135.6 (2C, Ar-C), 131.2 (1C, Ar-C), 130.1(d, 1C, J = 15.7 Hz,Ar-C), 129.8 (1C, Ar-C), 128.6 (d, 1C, J = 4.6 Hz,Ar-C), 127.8 (2C, Ar-C), 126.3 (d, 1C, J = 8.2 Hz, Ar-C), 124.0 (1C, Ar-C), 115.2 (d, 1C, J = 23.0 Hz, Ar-C), 31.6 (1C, CH_2), 30.6 (1C, CH_2), 28.7 (1C, CH_2), 23.8(1C, CH_2), 22.4 (1C, CH), 14.0(1C, CH_3); ^{29}Si NMR (79 MHz, $CDCl_3$) δ -23.9. HRMS (EI) calcd for [M, $C_{18}H_{23}FSi]^+$: 286.1553; found 286.1550.

(1-(4-Fluorophenyl)hexyl)(phenyl)silane (8o)

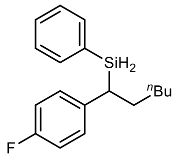

94% yield (538.6 mg), colorless oil. ^{1}H NMR (400 MHz, $CDCl_3$) δ 7.41−7.33 (m, 3H, Ar-H), 7.32−7.27 (m, 2H, Ar-H), 7.00−6.88 (m, 4H, Ar-H), 4.33 (dd, J = 6.6, 3.0 Hz, 1H, Si-H), 4.29 (dd, J = 6.0, 3.4 Hz, 1H, Si-H), 2.47−2.38 (m, 1H, CH), 1.83−1.75 (m, 2H, CH_2), 1.32−1.15 (m, 6H, $CH_2CH_2CH_2$), 0.84−0.79 (m, 3H, CH_3); ^{13}C NMR (101 MHz, $CDCl_3$) δ 160.7 (d, J = 242.8 Hz, 1C, F-C), 138.5 (d, J = 2.7 Hz, 1C, Ar-C), 138.5 (1C, Ar-C), 135.6 (2C, Ar-C), 131.2 (1C, Ar-C), 129.7 (1C, Ar-C), 129.0 (d, J = 7.7 Hz, 2C, Ar-C), 127.8 (2C, Ar-C), 115.1 (d, J = 21.0 Hz, 2C, Ar-C), 31.6 (1C, CH_2), 31.5 (2C, CH_2), 28.6 (1C, CH_2), 22.4 (1C, CH_2), 14.0 (1C, CH_3); ^{29}Si NMR (79 MHz, $CDCl_3$) δ -23.8. HRMS (EI) calcd for [M, $C_{18}H_{23}FSi]^+$: 286.1553; found 286.1554.

Phenyl(1-(4-(methoxymethyl)phenyl)hexyl)silane (8p)

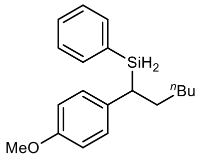

92% yield (548.7 mg), colorless oil. ^{1}H NMR (400 MHz, $CDCl_3$) δ 7.40−7.34 (m, 3H, Ar-H), 7.32−7.26 (m, 2H, Ar-H), 6.97 (d, *J* = 8.1 Hz, 2H, Ar-H), 6.79 (d, *J* = 8.1 Hz, 2H, Ar-H), 4.35−4.26 (m, 2H, Si-H), 3.78 (s, 3H, OCH_3), 2.43−2.34 (m, 1H, CH), 1.83−1.73(m, 2H, CH_2), 1.32−1.15 (m, 6H, $CH_2CH_2CH_2$), 0.84−0.78 (m, 3H, CH_3); ^{13}C NMR (101 MHz, $CDCl_3$) δ 157.1 (1C, Ar-C), 135.7 (2C, Ar-C), 134.9 (1C, Ar-C), 131.7 (1C, Ar-C), 129.6 (1C, Ar-C), 128.7 (2C, Ar-C), 127.8 (2C, Ar-C), 113.8 (2C, Ar-C), 55.2 (1C, OCH_3), 31.6 (1C, CH_2), 31.6 (1C, CH_2), 31.1 (1C, CH_2), 28.6 (1C, CH_2), 22.5 (1C, CH), 14.0 (1C, CH_3); ^{29}Si NMR (79 MHz, $CDCl_3$) δ -23.0. HRMS (EI) calcd for [M, $C_{19}H_{26}Si]^+$: 298.1753; found 298.1750.

(1-(Naphthalen-2-yl)hexyl)(phenyl)silane (8q)

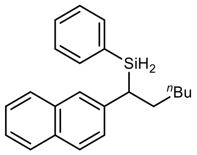

92% yield (586.0 mg), colorless oil.^{1}H NMR (400 MHz, $CDCl_3$) δ 7.77 (d, *J* = 7.8 Hz, 1H, Ar-H), 7.74−7.67 (m, 2H, Ar-H), 7.47 (s, 1H , Ar-H), 7.44−7.32 (m, 5H, Ar-H), 7.29−7.24 (m, 2H, Ar-H), 7.22−7.18 (m, 1H, Ar-H), 4.41−4.35 (m, 2H, Si-H), 2.67−2.57 (m, 1H, CH), 2.04−1.84 (m, 2H, CH_2), 1.37−1.15 (m, 6H, $CH_2CH_2CH_2$), 0.81 (t, *J* = 6.6 Hz, 3H, CH_3); ^{13}C NMR (101 MHz, $CDCl_3$) δ 140.7 (1C, Ar-C), 135.7 (2C, Ar-C), 133.8 (1C, Ar-C), 131.6 (1C, Ar-C), 131.4 (1C, Ar-C), 129.7 (1C, Ar-C), 127.9 (1C, Ar-C), 127.8 (2C, Ar-C), 127.6 (1C, Ar-C), 127.3 (1C, Ar-C), 127.0 (1C, Ar-C), 125.8 (1C, Ar-C), 125.6 (1C, Ar-C), 124.8 (1C, Ar-C), 32.5 (1C, CH), 31.6 (1C, CH_2), 31.3 (1C, CH_2), 28.8 (1C, CH_2), 22.4 (1C, CH_2), 14.0 (1C, CH_3); ^{29}Si NMR (79 MHz, $CDCl_3$) δ -24.1. HRMS (EI) calcd for [M, $C_{22}H_{26}Si]^+$: 318.1804; found 318.1801.

(1-(Benzo[d][1,3]dioxol-5-yl)hexyl)(phenyl)silane (8r)

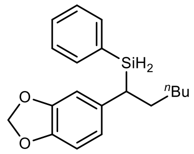

91% yield (71.1 mg) [conditions: **7a**/$PhSiH_3$/**2e**/EtMgBr = 0.25:0.275:0.0125:0.0275 (mmol), in 1 mL THF, at 30 °C, 24 h], colorless oil. ^{1}H NMR (400 MHz, $CDCl_3$) δ 7.42−7.35 (m, 3H, Ar-H), 7.33−7.28 (m, 2H, Ar-H), 6.68 (d, *J* = 7.9 Hz, 1H, Ar-H), 6.57 (d, *J* = 1.6 Hz, 1H, Ar-H), 6.49 (dd, *J* = 8.0, 1.7 Hz, 2H, Ar-H), 5.91 (s, 2H, CH_2), 4.32 (dd, *J* = 6.6, 3.2 Hz, 1H, Si-H), 4.29 (dd, *J* = 6.6, 3.3 Hz, 1H, Si-H), 2.37 (ddd, *J* = 12.4, 6.5, 3.2 Hz, 1H, CH), 2.40−2.33 (m, 2H, CH_2), 1.35−1.15 (m, 6H, $CH_2CH_2CH_2$), 0.82 (t, *J* = 6.7 Hz, 3H, CH_3); ^{13}C NMR (101 MHz, $CDCl_3$) δ 147.6 (1C, Ar-C) 144.9 (1C, Ar-C), 136.8 (1C, Ar-C), 135.6 (2C, Ar-C), 131.5 (1C, Ar-C), 129.7 (1C, Ar-C), 127.8 (2C, Ar-C), 120.6 (2C, Ar-C), 108.2 (2C, Ar-C), 100.7 (1C, CH_2), 31.9 (1C, CH_2), 31.7 (1C, CH_2), 31.6 (1C, CH_2), 28.6 (1C, CH_2), 22.5 (1C, CH_2) 14.1 (1C, CH_3); ^{29}Si NMR (79 MHz, $CDCl_3$) δ -23.2. HRMS (EI) calcd for [M, $C_{19}H_{24}O_2Si$]$^+$: 312.1546; found 312.1548.

Supplementary Table 2 | ICP-AES analysis of iron precatalysts (wt%)

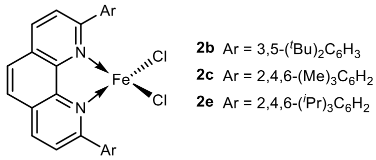

element	**2b**	**2c**	**2e**
Fe	7.48	8.97	8.00
Cu	0.007	0.003	0.052
Ni	0.010	0.003	0.026
Zn	0.018	0.013	0.090
Cr	0.010	0.003	0.056
Ru	0.006	0.008	0.010
Pt	-	-	-
Pd	-	-	-
Au	-	-	-

Cd	-	-	-
Pb	-	-	-
Co	-	-	-

'-' means < 0.001

Background experiments

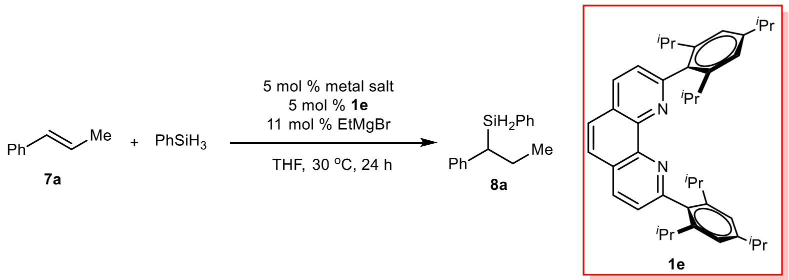

In an argon-filled glovebox, a vial (4 mL) was charged with metal salt (0.025 mmol), **1e** (14.6 mg, 0.025 mmol) and THF (0.5 mL). The reaction mixture was stirred at room temperature for 12 h, then **7a** (59.1 mg, 0.5 mmol), $PhSiH_3$ (59.5 mg, 0.55 mmol) and EtMgBr (1 M in THF, 55 μL, 0.055 mmol, 11 mol %) was added. After stirring for 24 hours at 30 °C, the vial was removed from the glovebox and the reaction mixture was concentrated by rotating evaporation. Iron was removed by flash column chromatography with hexane and **8a** detected by GC with *n*-dodecane as internal standard.

Supplementary Table 3 | Metal salt effect

entry	metal salt	yield
1	$FeCl_2$	91%
2	none	0
3	$CuCl_2$	0
4	$NiCl_2$	0
5	$ZnCl_2$	0
6	$CrCl_2$	0
7	$RuCl_3$	0

The robustness screen experiments

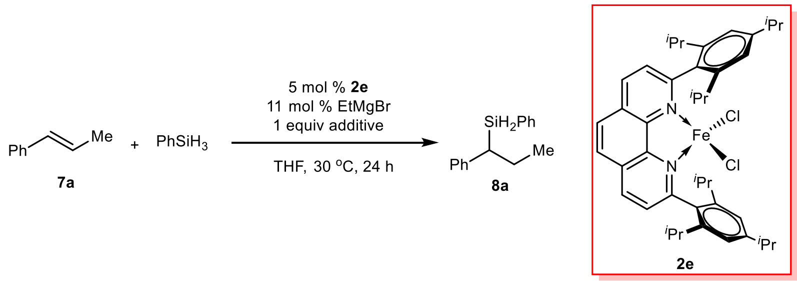

In an argon-filled glovebox, a vial (10 mL) was charged with **7a** (59.1 mg, 0.5 mmol), $PhSiH_3$ (64.9 mg, 0.6 mmol), additive (0.5 mmol), anhydrous THF (1 mL), and complex **2e** (17.8 mg, 0.025 mmol). The reaction mixture was stirred at room temperature for 1 minute, then EtMgBr (1 M in THF, 55 μL, 0.055 mmol, 11 mol %) was added. After stirring for 24 hours at 30 °C, the vial was removed from the glovebox and iron was removed by flash column chromatography with THF. The yield of **8a**, additive remaining, and remaning of **7a** were mersured by Gas Chromatography (GC) with *n*-dodecane as internal standard. Injector temperature was set at 230 °C. The carrier gas was nitrogen with a flow rate of 1.0 mL/min. The following temperature program was used in the analysis of **8a**: 50 °C-10 °C/min-300 °C (5 min). In these conditions, the following retention times were observed: **8a** (12.817 min), *n*-dodecane (7.361 min).

Supplementary Table 4 | Robustness screen experiments

entry	additive	yield of **8a** (%)	additive remaining (%)	remaining of **7a** (%)
1	none	92	-	< 5
2	PhCOPh	82	99	17
3	$PhCO_2{}^tBu$	78	99	20
4	PhB(Pin)	85	98	13
5	PhCCPh	89	99	10
6	*N*-Me-indole	89	99	10
7	*N*-Me-pyrrole	90	90	8
8	furan	91	93	5
9	PhCHO	2	94	97
10	$PhCON(CH_2)_5$	0	99	98
11	PhCN	0	99	99
12	$PhNO_2$	0	95	99

Hydrosilylation of terminal styrenes

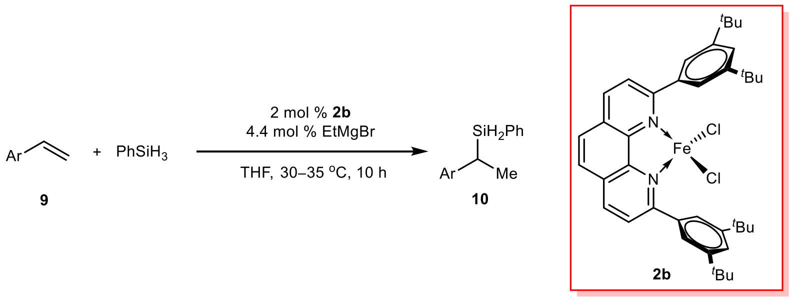

In an argon-filled glovebox, a vial (4 mL) was charged with alkene (0.5 mmol), silane (0.55 mmol), anhydrous THF (1 mL) and complex **2e** (0.01 mmol). The reaction mixture was stirred at room temperature (30–35 °C) for 3 minutes, then EtMgBr (1 M in THF, 22 μL, 0.022 mmol, 4.4 mol %) was added. After stirring for 10 hours, the vial was removed from the glovebox and the reaction mixture was concentrated by rotating evaporation. The residue was purified by flash chromatography to afford the desired product.

Phenyl(1-phenylethyl)silane (10a)[21]

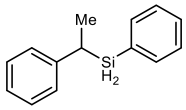

92% yield (97.7 mg), colorless oil. ^{1}H NMR (400 MHz, $CDCl_3$) δ 7.42−7.35 (m, 3H, Ar-H), 7.33−7.22 (m, 4H, Ar-H), 7.15−7.07 (m, 3H, Ar-H), 4.35−4.28 (m, 2H, Si-H), 2.67−2.57 (m, 1H, CH), 1.45 (d, J = 7.7 Hz, 3H, CH_3); ^{13}C NMR (101 MHz, $CDCl_3$) δ 144.5 (1C, Ar-C), 135.6 (2C, Ar-C), 131.4 (1C, Ar-C), 129.7 (1C, Ar-C), 128.4 (2C, Ar-C), 127.8 (2C, Ar-C), 127.1 (2C, Ar-C), 125.0 (1C, Ar-C), 25.4 (1C, CH), 16.3 (1C, CH_3); ^{29}Si NMR (79 MHz, $CDCl_3$) δ -20.8. HRMS (EI) calcd for [M, $C_{14}H_{16}Si$]$^+$: 212.1021; found 212.1023.

Phenyl(1-(*o*-tolyl)ethyl)silane (10b)

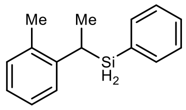

90% yield (101.9 mg), colorless oil. ^{1}H NMR (400 MHz, $CDCl_3$) δ 7.43−7.35 (m, 3H, Ar-H), 7.33−7.26 (m, 2H, Ar-H), 7.18−7.13 (m, 1H, Ar-H), 7.10 (d, J = 7.7 Hz, 2H, Ar-

H), 7.06−7.01 (m, 1H, Ar-H), 4.32−4.25 (m, 2H, Si-H), 2.81−2.73 (m, 1H, CH), 2.20 (s, 3H, CH_3), 1.45 (d, J = 7.4 Hz, 3H, CH_3); ^{13}C NMR (101 MHz, $CDCl_3$) δ 142.8 (1C, Ar-C), 135.6 (2C, Ar-C), 134.8 (1C, Ar-C), 131.6 (1C, Ar-C), 130.1 (1C, Ar-C), 129.7 (1C, Ar-C), 127.8 (2C, Ar-C), 126.2 (1C, Ar-C), 126.0 (1C, Ar-C), 124.8 (1C, Ar-C), 20.7 (1C, CH_3), 20.0 (1C, CH), 16.5 (1C, CH_3); ^{29}Si NMR (79 MHz, $CDCl_3$) δ -23.0. HRMS (EI) calcd for [M, $C_{15}H_{18}Si$]$^+$: 226.1178; found 226.1177.

(1-(2-Methoxyphenyl)ethyl)(phenyl)silane (10c)[22]

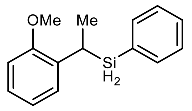

91% yield (110.3 mg), colorless oil. ^1H NMR (400 MHz, $CDCl_3$) δ 7.44 (dd, J = 7.8, 1.4 Hz, 2H, Ar-H), 7.39−7.34 (m, 1H, Ar-H), 7.34−7.27 (m, 2H , Ar-H), 7.15−7.07 (m, 2H , Ar-H), 6.91 (t, J = 7.4 Hz, 1H, Ar-H), 6.78 (d, J = 8.1 Hz, 1H, Ar-H), 4.27 (dd, J = 7.0, 3.2 Hz, 1H, Si-H), 4.24 (dd, J = 7.0, 2.4 Hz, 1H, Si-H), 3.68 (s, 3H, OMe), 3.05−2.95 (m, 1H, CH), 1.41 (d, J = 7.5 Hz, 3H, CH_3); ^{13}C NMR (101 MHz, $CDCl_3$) δ 156.1 (1C, Ar-C), 135.5 (2C, Ar-C), 133.3 (1C, Ar-C), 132.6 (1C, Ar-C), 129.4 (1C, Ar-C), 127.7 (2C, Ar-C), 126.7 (1C, Ar-C), 125.7 (1C, Ar-C), 120.6 (1C, Ar-C), 109.9 (1C, Ar-C), 55.0 (1C, OCH_3), 17.8 (1C, CH), 15.7 (1C, CH_3); ^{29}Si NMR (79 MHz, $CDCl_3$) δ -19.4. HRMS (EI) calcd for [M, $C_{15}H_{18}OSi$]$^+$: 242.1127; found 242.1119.

(1-(2-Fluorophenyl)ethyl)(phenyl)silane (10d)

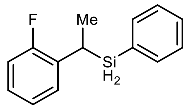

90% yield (103.7 mg) (Used 5 mol % **2e** and 11 mol % EtMgBr), colorless oil. ^1H NMR (400 MHz, $CDCl_3$) δ 7.48−7.42 (m, 2H, Ar-H), 7.42−7.36 (m, 1H, Ar-H), 7.34−7.28 (m, 2H, Ar-H), 7.14−7.02 (m, 3H, Ar-H), 7.00−6.93 (m, 1H, Ar-H), 4.37−4.28 (m, 2H, Si-H), 2.98−2.88 (m, 1H, CH), 1.43 (d, J = 7.5 Hz, 3H, CH_3); ^{13}C NMR (101 MHz, $CDCl_3$) δ 160.0 (1C, d, J = 243.8 Hz, F-C), 135.6 (2C, Ar-C), 131.7 (1C, d, J = 15.4 Hz, Ar-C), 131.0 (1C, Ar-C), 129.8 (1C, Ar-C), 127.9 (1C, d, J = 4.7 Hz, Ar-C), 127.9 (2C, Ar-C), 126.3 (1C, d, J = 8.1 Hz, Ar-C), 124.1 (1C, d, J = 3.5 Hz), 115.0 (1C, d, J = 22.6 Hz, Ar-C), 17.4 (1C, d, J = 1.7 Hz, CH), 15.6 (1C, CH_3); ^{19}F NMR (376 MHz,

CDCl$_3$) δ -117.3; ^{29}Si NMR (79 MHz, CDCl$_3$) δ -21.0. HRMS (EI) calcd for [M, $C_{14}H_{15}FSi$]$^+$: 230.0927; found 230.0924.

Phenyl(1-(*m*-tolyl)ethyl)silane (10e)

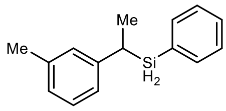

90% yield (101.8 mg), colorless oil. ^{1}H NMR (400 MHz, CDCl$_3$) δ 7.43−7.35 (m, 3H, Ar-H), 7.33−7.27 (m, 2H, Ar-H), 7.16−7.10 (m, 1H, Ar-H), 6.93 (d, J = 7.5 Hz, 1H, Ar-H), 6.91−6.87 (m, 2H, Ar-H), 4.34−4.28 (m, 2H, Si-H), 2.61−2.52 (m, 1H, CH), 2.29 (s, 3H, CH$_3$), 1.43 (d, J = 7.5 Hz, 3H, CH$_3$); ^{13}C NMR (101 MHz, CDCl$_3$) δ 144.4 (1C, Ar-C), 137.8 (2C, Ar-C), 135.6 (2C, Ar-C), 131.5 (1C, Ar-C), 129.7 (1C, Ar-C), 128.2 (1C, Ar-C), 127.9 (1C, Ar-C), 127.8 (2C, Ar-C), 125.8 (1C, Ar-C), 124.1 (1C, Ar-C), 25.2 (1C, CH), 21.5 (1C, CH$_3$), 16.4 (1C, CH$_3$); ^{29}Si NMR (79 MHz, CDCl$_3$) δ -17.1. HRMS (EI) calcd for [M, $C_{15}H_{18}Si$]$^+$: 226.1178; found 226.1174.

(1-(3-Methoxyphenyl)ethyl)(phenyl)silane (10f)

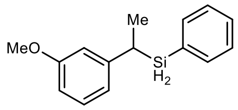

91% yield (110.3 mg), colorless oil. ^{1}H NMR (400 MHz, CDCl$_3$) δ 7.44−7.35 (m, 3H, Ar-H), 7.34−7.27 (m, 2H, Ar-H), 7.19−7.12 (m, 1H, Ar-H), 6.72−6.64 (m, 2H, Ar-H), 6.63−6.59 (m, 1H, Ar-H), 4.34−4.29 (m, 2H, Si-H), 3.73 (s, 3H, OCH$_3$), 2.65−2.54 (m, 1H, CH), 1.44 (d, J = 7.5 Hz, 3H, CH$_3$); ^{13}C NMR (101 MHz, CDCl$_3$) δ 159.6 (1C, Ar-C), 146.3 (1C, Ar-C), 135.7 (2C, Ar-C), 131.4 (1C, Ar-C), 129.8 (1C, Ar-C), 129.3 (1C, Ar-C), 127.8 (2C, Ar-C), 119.6 (1C, Ar-C), 112.8 (1C, Ar-C), 110.4 (1C, Ar-C), 55.0 (1C, OCH$_3$), 25.5 (1C, CH), 16.3 (1C, CH$_3$); ^{29}Si NMR (79 MHz, CDCl$_3$) δ -20.8. HRMS (EI) calcd for [M, $C_{15}H_{18}OSi$]$^+$: 242.1127; found 242.1125.

(1-(3-Fluorophenyl)ethyl)(phenyl)silane (10g)

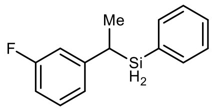

90% yield (103.7 mg) (used 5 mol % **2e** and 11 mol % EtMgBr), colorless oil. ^{1}H NMR

(400 MHz, $CDCl_3$) δ 7.43−7.37 (m, 3H, Ar-H), 7.35−7.29 (m, 2H, Ar-H), 7.22−7.16 (m, 1H, Ar-H), 6.89−6.74 (m, 3H), 4.34−4.29 (m, 2H, Si-H), 2.68−2.58 (m, 1H, CH), 1.44 (d, J = 7.4 Hz, 3H, CH_3); ^{13}C NMR (101 MHz, $CDCl_3$) δ 163.0 (d, J = 245.0 Hz, 1C, Ar-H), 147.4 (d, J = 7.0 Hz, 1C, Ar-H), 135.6 (2C, Ar-H), 130.8 (1C, Ar-C), 129.9 (2C, Ar-C), 129.6 (d, J = 8.5 Hz, 1C, Ar-H), 127.9 (2C, Ar-H), 122.8 (d, J = 2.3 Hz, 1C, Ar-H), 113.8 (d, J = 21.3 Hz, 1C, Ar-H), 111.8 (d, J = 21.0 Hz, 1C, Ar-H), 25.5 (1C, CH), 16.2 (1C, CH_3); ^{29}Si NMR (79 MHz, $CDCl_3$) δ -20.5; ^{19}F NMR (376 MHz, $CDCl_3$) δ -113.6. HRMS (EI) calcd for $[M, C_{14}H_{15}FSi]^+$: 230.0927; found 230.0921.

(1-(3-Chlorophenyl)ethyl)(phenyl)silane (10h)

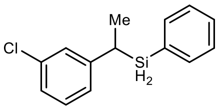

90% yield (111.1 mg) (used 5 mol % **2e** and 11 mol % EtMgBr), colorless oil. ^1H NMR (400 MHz, $CDCl_3$) δ 7.43−7.37 (m, 3H, Ar-H), 7.35−7.29 (m, 2H, Ar-H), 7.16 (t, J = 7.7 Hz, 1H, Ar-H), 7.12−7.04 (m, 2H, Ar-H), 6.94 (d, J = 7.5 Hz, 1H, Ar-H), 4.34−4.28 (m, 2H, Si-H), 2.64−2.55 (m, 1H, CH), 1.43 (d, J = 7.5 Hz, 3H, CH_3); ^{13}C NMR (101 MHz, $CDCl_3$) δ 146.8 (1C, Ar-C), 135.6 (2C, Ar-C), 134.1 (1C, Ar-C), 130.7 (1C, Ar-C), 129.9 (1C, Ar-C), 129.5 (1C, Ar-C), 127.9 (2C, Ar-C), 127.1 (1C, Ar-C), 125.3 (1C, Ar-C), 125.2 (1C, Ar-C), 25.4 (1C, CH), 16.1 (1C, CH_3); ^{29}Si NMR (79 MHz, $CDCl_3$) δ -20.6. HRMS (EI) calcd for $[M, C_{14}H_{15}ClSi]^+$: 246.0632; found 246.0629.

Phenyl(1-(*p*-tolyl)ethyl)silane (10i)[23]

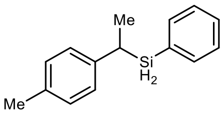

91% yield (103.0 mg), colorless oil. ^1H NMR (400 MHz, $CDCl_3$) δ 7.45−7.36 (m, 3H, Ar-H), 7.34−7.28 (m, 2H, Ar-H), 7.07 (d, J = 8.0 Hz, 2H, Ar-H), 7.00 (d, J = 8.1 Hz, 2H, Ar-H), 4.33−4.27 (m, 2H, Si-H), 2.62−2.53 (m, 1H, CH), 2.31 (s, 3H, CH_3), 1.43 (d, J = 3.7 Hz, 3H, CH_3). ^{13}C NMR (101 MHz, $CDCl_3$) δ 141.4 (1C, Ar-C), 135.6 (2C, Ar-C), 134.4 (1C, Ar-C), 131.6 (1C, Ar-C), 129.7 (1C, Ar-C), 129.1 (2C, Ar-C), 127.8 (2C, Ar-C), 127.0 (2C, Ar-C), 24.8 (1C, CH_3), 20.9 (1C, CH), 16.5 (1C, CH_3); ^{29}Si NMR (79 MHz, $CDCl_3$) δ -21.0. HRMS (EI) calcd for $[M, C_{15}H_{18}Si]^+$: 226.1178; found

226.1176.

(1-(4-Methoxyphenyl)ethyl)(phenyl)silane (10j)[22]

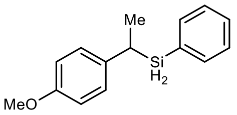

93% yield (112.7 mg), colorless oil. ^{1}H NMR (400 MHz, $CDCl_3$) δ 7.43−7.35 (m, 3H, Ar-H), 7.34−7.28 (m, 2H, Ar-H), 7.01 (d, J = 8.5 Hz, 2H, Ar-H), 6.81 (d, J = 8.5 Hz, 2H, Ar-H), 4.33−4.27 (m, 2H, Si-H), 3.78 (s, 3H, OCH_3), 2.51−2.61 (m, 1H, CH), 1.42 (d, J = 7.5 Hz, 3H, CH_3); ^{13}C NMR (101 MHz, $CDCl_3$) δ 157.2 (1C, Ar-C), 136.5 (2C, Ar-C), 135.6 (1C, Ar-C), 131.6 (1C, Ar-C), 129.7 (1C, Ar-C), 128.0 (2C, Ar-C), 127.8 (2C, Ar-C), 113.9 (2C, Ar-C), 55.2 (1C, Ar-C), 24.2 (1C, CH), 16.7 (1C, CH_3); ^{29}Si NMR (79 MHz, $CDCl_3$) δ -21.3. HRMS (EI) calcd for [M, $C_{15}H_{18}OSi$]$^+$: 242.1127; found 242.1134.

(1-(4-Fluorophenyl)ethyl)(phenyl)silane (10k)[22]

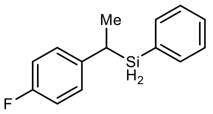

90% yield (103.7 mg), colorless oil. ^{1}H NMR (400 MHz, $CDCl_3$) δ 7.42−7.36 (m, 3H, Ar-H), 7.34−7.28 (m, 2H, Ar-H), 7.05−6.98 (m, 2H, Ar-H), 6.96−6.89 (m, 2H, Ar-H), 4.32−4.28 (m, 2H, Si-H), 2.64−2.55 (m, 1H, CH), 1.43 (d, J = 7.5 Hz, 3H, CH_3); ^{13}C NMR (101 MHz, $CDCl_3$) δ 160.7 (d, J = 242.8 Hz, 1C, F-C), 140.1 (d, J = 2.7 Hz, 1C, Ar-C), 135.6 (2C, Ar-C), 131.0 (1C, Ar-C), 129.8 (1C, Ar-C), 128.3 (d, J = 7.7 Hz, 2C, Ar-C), 127.9 (2C, Ar-C), 115.1 (d, J = 21.0 Hz, 2C, Ar-C), 24.6 (1C, CH), 16.6 (1C, CH_3); ^{29}Si NMR (79 MHz, $CDCl_3$) δ -21.9; ^{19}F NMR (376 MHz, $CDCl_3$) δ -118.7. HRMS (EI) calcd for [M, $C_{14}H_{15}FSi$]$^+$: 230.0927; found 230.0933.

(1-(4-Chlorophenyl)ethyl)(phenyl)silane (10l)

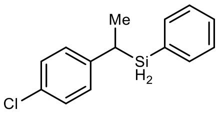

88% yield (108.6 mg) (Used 5 mol % **2e** and 11 mol % EtMgBr), colorless oil. ^{1}H NMR (400 MHz, $CDCl_3$) δ 7.41−7.36 (m, 3H, Ar-H), 7.35−7.29 (m, 2H, Ar-H), 7.20 (d, J = 8.4 Hz, 2H, Ar-H), 7.00 (d, J = 8.4 Hz, 2H, Ar-H), 4.30 (d, J = 3.1 Hz, 2H, Si-H),

2.64−2.55 (m, 1H, CH), 1.43 (d, J = 7.5 Hz, 3H, CH_3). ^{13}C NMR (101 MHz, $CDCl_3$) δ 143.1 (1C, Ar-C), 135.6 (2C, Ar-C), 130.9 (1C, Ar-C), 130.5 (1C, Ar-C), 129.9 (1C, Ar-C), 128.4 (2C, Ar-C), 128.4 (2C, Ar-C), 127.9 (2C, Ar-C), 25.0 (1C, CH), 16.3 (1C, CH_3); ^{29}Si NMR (79 MHz, $CDCl_3$) δ -20.9. HRMS (EI) calcd for [M, $C_{14}H_{15}ClSi$]$^+$: 246.0632; found 246.0628.

(1-(Naphthalene-1-yl)ethyl)(phenyl)silane (10m)

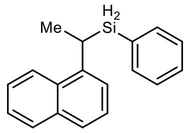

92% yield (120.7 mg), colorless oil. ^{1}H NMR (400 MHz, $CDCl_3$) δ 7.92−7.86 (m, 1H, Ar-H), 7.70−7.65 (m, 1H, Ar-H), 7.51−7.46 (m, 1H, Ar-H), 7.31−7.17 (m, 6H, Ar-H), 7.15−7.05 (m, 3H, Ar-H), 4.24 (d, J = 6.0 Hz, 1H, Si-H), 4.17 (dd, J = 6.8, 3.5 Hz, 1H, Si-H), 3.32−3.22 (m, 1H, CH), 1.42 (d, J = 7.3 Hz, 3H, CH_3); ^{13}C NMR (101 MHz, $CDCl_3$) δ 140.9 (1C, Ar-C), 135.7 (2C, Ar-C), 133.9 (1C, Ar-C), 131.4 (1C, Ar-C), 131.1 (1C, Ar-C), 129.8 (1C, Ar-C), 128.9 (1C, Ar-C), 127.8 (2C, Ar-C), 125.6 (2C, Ar-C), 125.5 (1C, Ar-C), 125.3 (1C, Ar-C), 123.4 (1C, Ar-C), 123.2 (1C, Ar-C), 20.0 (1C, CH), 16.8 (1C, CH_3); ^{29}Si NMR (79 MHz, $CDCl_3$) δ -21.0. HRMS (EI) calcd for [M, $C_{18}H_{18}Si$]$^+$: 262.1178; found 262.1175.

(1-(Naphthalene-2-yl)ethyl)(phenyl)silane (10n)[22]

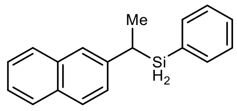

95% yield (124.6 mg), colorless oil. ^{1}H NMR (400 MHz, $CDCl_3$) δ 7.78 (d, J = 7.7 Hz, 1H, Ar-H), 7.72 (d, J = 8.3 Hz, 2H, Ar-H), 7.51 (s, 1H, Ar-H), 7.45−7.35 (m, 5H, Ar-H), 7.33−7.22 (m, 3H, Ar-H), 4.41−4.33 (m, 2H, Si-H), 2.84−2.74 (m, 1H, CH), 1.55 (d, 3H, J = 7.4 Hz, CH_3); ^{13}C NMR (101 MHz, $CDCl_3$) δ 142.2 (1C, Ar-C), 135.7 (2C, Ar-C), 133.8 (1C, Ar-C), 131.6 (1C, Ar-C), 131.3 (1C, Ar-C), 129.8 (1C, Ar-C), 127.9 (2C, Ar-C), 127.8 (1C, Ar-C), 127.6 (1C, Ar-C), 127.3 (1C, Ar-C), 126.7 (1C, Ar-C), 125.9 (1C, Ar-C), 124.8 (1C, Ar-C), 124.5 (1C, Ar-C), 25.7 (1C, CH), 16.4 (1C, CH_3); ^{29}Si NMR (79 MHz, $CDCl_3$) δ -19.0. HRMS (EI) calcd for [M, $C_{18}H_{18}Si$]$^+$: 262.1178;

found 262.1174.

(1-(Benzo[*d*][1,3]dioxol-5-yl)ethyl)(phenyl)silane (10o)

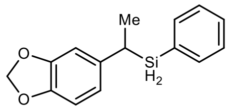

90% yield (115.4 mg) (Used 5 mol % **2e** and 11 mol % EtMgBr), colorless oil. ^{1}H NMR (400 MHz, $CDCl_3$) δ 7.45−7.36 (m, 3H, Ar-H), 7.34−7.29 (m, 2H, Ar-H), 6.70 (d, *J* = 8.0 Hz, 1H , Ar-H), 6.62 (d, *J* = 1.8 Hz, 1H , Ar-H), 6.53 (dd, *J* = 8.0, 1.7 Hz, 1H , Ar-H), 5.91 (s, 2H, OCH_2O) 4.31 (dd, *J* = 6.0, 3.1 Hz, 1H, Si-H), 4.29 (dd, *J* = 6.0, 3.4 Hz, 1H, Si-H), 2.59−2.50 (m, 1H), 1.40 (d, *J* = 7.5 Hz, 3H); ^{13}C NMR (101 MHz, $CDCl_3$) δ 147.6 (1C, Ar-C), 145.0 (1C, Ar-C), 138.5 (1C, Ar-C), 135.6 (2C, Ar-C), 131.4 (1C, Ar-C), 129.8 (1C, Ar-C), 127.9 (2C, Ar-C), 119.8 (1C, Ar-C), 108.2 (1C, Ar-C), 107.7 (1C, Ar-C), 100.7 (1C, OCH_2O), 25.1 (1C, CH), 16.9 (1C, CH_3); ^{29}Si NMR (79 MHz, $CDCl_3$) δ -21.0. HRMS (EI) calcd for [M, $C_{15}H_{16}O_2Si$]$^+$: 256.0920; found 256.0924.

Hydrosilylation of other internal alkenes

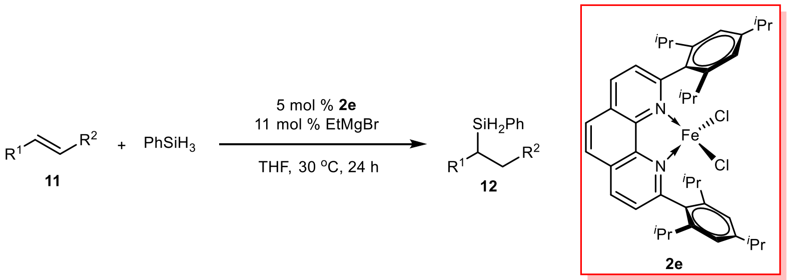

In an argon-filled glovebox, a vial (10 mL) was charged with alkene (0.5 mmol), silane (0.6 mmol), anhydrous THF (1 mL) and complex **2e** (0.025 mmol). The reaction mixture was stirred at 30 °C for 1 minutes, then EtMgBr (1 M in THF, 55 μL, 0.055 mmol, 11 mol %) was added. After stirring for 24 hours at 30 °C, the vial was removed from the glovebox and the reaction mixture was concentrated by rotating evaporation. The residue was purified by flash chromatography to afford the desired product.

(2,3-Dihydro-1*H*-inden-1-yl)(phenyl)silane (12a)

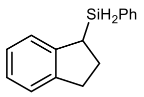

82% yield (92.0 mg) (used 0.55 mol $PhSiH_3$), 93% regioselectivity, colorless oil. ^{1}H

NMR (400 MHz, $CDCl_3$) δ 7.52−7.46 (m, 2H, Ar-H), 7.42−7.37 (m, 1H, Ar-H), 7.36−7.30 (m, 2H, Ar-H), 7.21−7.16 (m, 1H, Ar-H), 7.12−7.06 (m, 3H, Ar-H), 4.43−4.33 (m, 2H, Si-H), 2.99−2.74 (m, 3H, CH and CH_2), 2.44−2.30 (m, 1H, CH_2), 2.15−2.06 (m, 1H, CH_2); ^{13}C NMR (101 MHz, $CDCl_3$) δ 145.1 (1C, Ar-C), 143.5 (1C, Ar-C), 135.3 (2C, Ar-C),131.5 (1C, Ar-C), 129.8 (1C, Ar-C), 127.9 (2C, Ar-C), 126.2 (1C, Ar-C), 125.5 (1C, Ar-C), 124.4 (1C, Ar-C), 123.7 (1C, Ar-C), 32.4 (1C, CH), 29.6 (1C, CH_2), 28.4 (1C, CH_2); ^{29}Si NMR (79 MHz, $CDCl_3$) δ -20.5. HRMS (EI) calcd for [M, $C_{15}H_{16}Si$]$^+$: 224.1021; found 224.1023.

Phenyl(1-phenylheptyl)silane (12b)

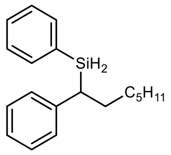

The **10b** can't be separated from $PhSiH_2SiH_2Ph$ [^{1}H NMR (400 MHz, $CDCl_3$) δ 4.38 (s, Si-H)], the yield was determined by ^{1}H NMR. 88% yield, 90% regioselectivity, colorless oil. ^{1}H NMR (400 MHz, $CDCl_3$) δ 7.39−7.34 (m, 3H, Ar-H), 7.32−7.19 (m, 4H, Ar-H), 7.14−7.08 (m, 1H, Ar-H), 7.06−7.02 (m, 2H, Ar-H), 4.34 (dd, J = 6.6, 3.1 Hz, 1H, Si-H), 4.31 (dd, J = 6.6, 3.3 Hz, 1H, Si-H), 2.50−2.41 (m, 1H, CH), 1.88−1.77 (m, 2H, CH_2), 1.35−1.15 (m, 8H, $CH_2CH_2CH_2CH_2$), 0.84−0.78 (m, 3H, CH_3); ^{13}C NMR (101 MHz, $CDCl_3$) δ 143.0 (1C, Ar-C), 135.7 (2C, Ar-C), 131.5 (1C, Ar-C), 129.7 (1C, Ar-C), 128.4 (2C, Ar-C), 127.9 (2C, Ar-C), 127.8 (2C, Ar-C), 125.0 (1C, Ar-C), 32.3 (1C, CH_2), 31.7 (1C, CH_2), 31.3 (1C, CH_2), 29.1 (1C, CH_2), 29.0 (1C, CH_2), 22.6 (1C, CH), 14.1 (1C, CH_3); ^{29}Si NMR (79 MHz, $CDCl_3$) δ -23.2. HRMS (EI) calcd for [M, $C_{19}H_{26}Si$]$^+$: 282.1804; found 282.1806.

Phenyl(1-phenyloctyl)silane (12c)

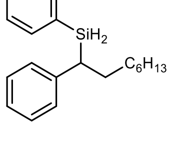

The **10c** can't be separated from $PhSiH_2SiH_2Ph$ [^{1}H NMR (400 MHz, $CDCl_3$) δ 4.38 (s, Si-H)], the yield was determined by ^{1}H NMR. 78% yield, 76% regioselectivity,

Colorless oil. ^{1}H NMR (400 MHz, $CDCl_3$) δ 7.39−7.34 (m, 3H, Ar-H), 7.32−7.19 (m, 4H, Ar-H), 7.14−7.08 (m, 1H, Ar-H), 7.06−7.02 (m, 2H, Ar-H), 4.34 (dd, J = 6.6, 3.1 Hz, 1H, Si-H), 4.31 (dd, J = 6.6, 3.3 Hz, 1H, Si-H), 2.50−2.41 (m, 1H, CH), 1.88−1.77 (m, 2H, CH_2), 1.36−1.14 (m, 10H, $CH_2CH_2CH_2CH_2CH_2$), 0.87−0.81 (m, 3H, CH_3); ^{13}C NMR (101 MHz, $CDCl_3$) δ 143.0 (1C, Ar-C), 135.7 (2C, Ar-C), 131.5 (1C, Ar-C), 129.6 (1C, Ar-C), 128.3 (2C, Ar-C), 127.8 (2C, Ar-C), 127.8 (2C, Ar-C), 125.0 (1C, Ar-C), 32.2 (1C, CH_2), 31.8 (1C, CH_2), 31.3 (1C, CH_2), 29.4 (1C, CH_2), 29.1 (1C, CH_2), 29.0 (1C, CH_2), 22.6 (1C, CH), 14.1 (1C, CH_3); ^{29}Si NMR (79 MHz, $CDCl_3$) δ -23.3. HRMS (EI) calcd for [M, $C_{20}H_{28}Si$]$^+$: 296.1960; found 296.1962.

Hexyl(phenyl)silane (12d)

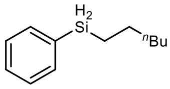

38% yield (36.6 mg), colorless oil. ^{1}H NMR (400 MHz, $CDCl_3$) δ 7.59−7.53 (m, 2H, Ar-H), 7.41−7.31 (m, 3H, Ar-H), 4.28 (t, J = 3.6 Hz, 2H, Si-H), 1.50−1.20 (m, 8H, CH_2), 0.98−0.90 (m, 2H, CH_2), 0.87 (t, J = 6.7 Hz, 3H, CH_3); ^{13}C NMR (101 MHz, $CDCl_3$) δ 135.2 (2C, Ar-C), 132.8 (1C, Ar-C), 129.4 (1C, Ar-C), 127.9 (2C, Ar-C), 32.5 (1C, CH_2), 31.5 (1C, CH_2), 25.0 (1C, CH_2), 22.5 (1C, CH_2), 14.1 (1C, CH_3), 10.0 (1C, CH_2-Si). ^{29}Si NMR (79 MHz, $CDCl_3$) δ -30.8. HRMS (EI) calcd for [M, $C_{12}H_{20}Si$]$^+$: 192.1334; found 192.1335.

Hydrosilylation of 1-substituted buta-1,3-dienes

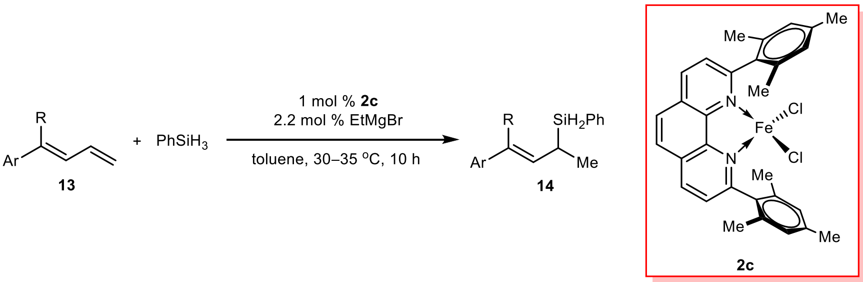

In an argon-filled glovebox, a vial (4 mL) was charged with alkene (0.5 mmol), silane (0.55 mmol), dry anhydrous toluene (1 mL) and complexes **2c** (0.005 mmol). The reaction mixture was stirred at 30–35 °C for 3 minutes, then EtMgBr (1 M in THF, 11 μL, 0.011 mmol, 2.2 mol %) was added. After 10 hours, the vial was removed from

the glovebox and the reaction mixture was concentrated by rotating evaporation. The residue was purified by flash chromatography to afford the desired product.

(*E*)-Phenyl(4-phenylbut-3-en-2-yl)silane (14a)[24]

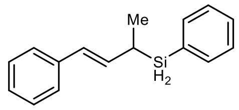

95% yield (113.2 mg), colorless oil. ^{1}H NMR (400 MHz, $CDCl_3$) δ 7.57 (d, J = 6.8 Hz, 2H, Ar-H), 7.44−7.32 (m, 3H, Ar-H), 7.32−7.23 (m, 4H, Ar-H), 7.20−7.14 (m, 1H, Ar-H), 6.33 (dd, J = 16.0, 7.0 Hz, 1H, CH), 6.26 (d, J = 16.0 Hz, 1H, CH), 4.32−4.26 (m, 2H, Si-H), 2.32−2.21 (m, 1H, CH), 1.29 (d, 3H, J = 7.2 Hz, CH_3); ^{13}C NMR (101 MHz, $CDCl_3$) δ 138.0 (1C, Ar-C), 135.7 (2C, Ar-C), 133.0 (1C, Ar-C), 131.1 (1C, Ar-C), 129.8 (1C, Ar-C), 128.5 (2C, Ar-C), 128.0 (2C, Ar-C), 127.2 (1C, Ar-C), 126.6 (1C, Ar-C), 125.7 (2C, Ar-C), 22.9 (1C, CH), 15.1 (1C, CH_3); ^{29}Si NMR (79 MHz, $CDCl_3$) δ -24.3; HRMS (EI) calcd for [M, $C_{16}H_{18}Si]^+$: 238.1178; found 238.1184.

(*E*)-Phenyl(4-(*p*-tolyl)but-3-en-2-yl)silane (14b)

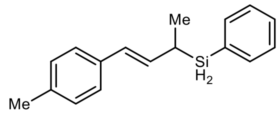

95% yield (119.9 mg), colorless oil. ^{1}H NMR (400 MHz, $CDCl_3$) δ 7.60−7.55 (m, 2H, Ar-H), 7.43−7.32 (m, 3H, Ar-H), 7.20 (d, J = 8.0 Hz, 2H, Ar-H), 7.09 (d, J = 8.0 Hz, 2H, Ar-H), 6.33−6.20 (m, 2H, CH), 4.32−4.24 (m, 2H, Si-H), 2.32 (s, 3H, CH_3), 2.28−2.20 (m, 1H, CH), 1.28 (d, 3H, J = 7.2 Hz, CH_3); ^{13}C NMR (101 MHz, $CDCl_3$) δ 136.3 (1C, Ar-C), 135.7 (2C, Ar-C), 135.2 (1C, Ar-C), 131.9 (1C, Ar-C), 131.2 (1C, Ar-C), 129.8 (2C, Ar-C), 129.2 (1C, Ar-C), 127.9 (2C, Ar-C), 127.1 (1C, Ar-C), 125.6 (2C, Ar-C), 22.7 (1C, CH_3), 21.1 (1C, CH), 15.1 (1C, CH_3); ^{29}Si NMR (79 MHz, $CDCl_3$) δ -24.3; HRMS (EI) calcd for [M, $C_{17}H_{20}Si]^+$: 252.1334; found 252.1337.

(*E*)-(4-(4-Methoxyphenyl)but-3-en-2-yl)(phenyl)silane (14c)

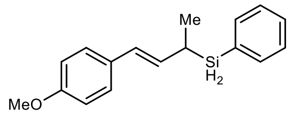

94% yield (126.1 mg), colorless oil. ^{1}H NMR (400 MHz, $CDCl_3$) δ 7.60−7.55 (m, 2H, Ar-H), 7.43−7.32 (m, 3H, Ar-H), 7.25−7.21 (m, 2H, Ar-H), 6.85−6.80 (m, 2H, Ar-H),

6.22 (d, J = 16.0 Hz, 1H, CH), 6.16 (dd, J = 16.0, 6.7 Hz, 1H, CH), 4.31−4.25 (m, 2H, Si-H), 3.80 (s, 3H, OCH_3), 2.28−2.18 (m, 1H, CH), 1.28 (d, 3H, J = 7.2 Hz, CH_3); ^{13}C NMR (101 MHz, $CDCl_3$) δ 158.5 (1C, Ar-C), 135.7 (2C, Ar-C), 131.3 (1C, Ar-C), 130.9 (1C, Ar-C), 130.8 (1C, Ar-C), 129.7 (1C, Ar-C), 127.9 (2C, Ar-C), 126.8 (2C, Ar-C), 126.6 (1C, Ar-C), 113.9 (2C, Ar-C), 55.3 (1C, OCH_3), 22.6 (1C, CH), 15.2 (1C, CH_3); ^{29}Si NMR (79 MHz, $CDCl_3$) δ -24.3; HRMS (EI) calcd for [M, $C_{17}H_{20}OSi$]$^+$: 268.1283; found 268.1285.

(*E*)-*N*,*N*-Dimethyl-4-(3-(phenylsilyl)but-1-en-1-yl)aniline (14d)

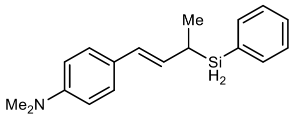

91% yield (128.1 mg), colorless oil. ^1H NMR (400 MHz, $CDCl_3$) δ 7.60−7.55 (m, 2H, Ar-H), 7.44−7.31 (m, 3H, Ar-H), 7.23−7.18 (m, 2H, Ar-H), 6.70−6.64 (m, 2H, Ar-H), 6.20 (d, J = 15.9 Hz, 1H, CH), 6.10 (dd, J = 15.9, 7.5 Hz, 1H, CH), 4.32−4.24 (m, 2H, Si-H), 2.93 (s, 6H, NCH_3), 2.26−2.16 (m, 1H, CH), 1.27 (d, 3H, J = 7.2 Hz, CH_3); ^{13}C NMR (101 MHz, $CDCl_3$) δ 149.6 (1C, Ar-C), 135.8 (2C, Ar-C), 131.6 (1C, Ar-C), 129.7 (1C, Ar-C), 128.8 (1C, Ar-C), 127.9 (2C, Ar-C), 127.1 (1C, Ar-C), 126.9 (1C, Ar-C), 126.6 (2C, Ar-C), 112.7 (2C, Ar-C), 40.7 (2C, NCH_3), 22.6 (1C, CH), 15.4 (1C, CH_3); ^{29}Si NMR (79 MHz, $CDCl_3$) δ -24.4; HRMS (EI) calcd for [M, $C_{18}H_{23}NSi$]$^+$: 281.1600; found 281.1605.

(*E*)-(4-(4-Fluorophenyl)but-3-en-2-yl)(phenyl)silane (14e)

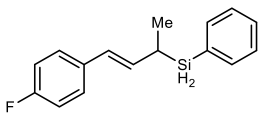

94% yield (120.5 mg), colorless oil. ^1H NMR (400 MHz, $CDCl_3$) δ 7.60−7.55 (m, 2H, Ar-H), 7.44−7.33 (m, 3H, Ar-H), 7.27−7.22 (m, 2H, Ar-H), 7.00−6.93 (m, 2H, Ar-H), 6.27−6.18 (m, 2H, CH), 4.32−4.26 (m, 2H, Si-H), 2.30−2.19 (m, 1H, CH), 1.28 (d, 3H, J = 7.2 Hz, CH_3); ^{13}C NMR (101 MHz, $CDCl_3$) δ 161.7 (d, J = 254.4 Hz, 1C, F−C), 135.7 (2C, Ar-C), 134.2 (d, J = 3.1 Hz, 1C, Ar-C), 132.8 (d, J = 1.9 Hz, 1C, Ar-C), 131.1 (1C, Ar-C), 129.8 (1C, Ar-C), 128.0 (1C, Ar-C), 127.1 (d, J = 7.6 Hz, 2C, Ar-C), 126.1 (1C, Ar-C), 115.3 (d, J = 21.5 Hz, 2C, Ar-C), 22.8 (1C, CH), 15.1 (1C, CH_3); ^{29}Si

NMR (79 MHz, $CDCl_3$) δ -24.2; ^{19}F NMR (376 MHz, $CDCl_3$) δ -115.9. HRMS (EI) calcd for [M, $C_{16}H_{17}FSi$]$^+$: 256.1084; found 256.1085.

(*E*)-(4-(4-Chlorophenyl)but-3-en-2-yl)(phenyl)silane (14f)

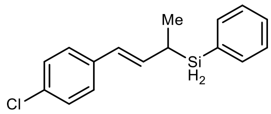

92% yield (125.5 mg), colorless oil. ^1H NMR (400 MHz, $CDCl_3$) δ 7.58−7.53 (m, 2H, Ar-H), 7.44−7.38 (m, 1H, Ar-H), 7.38−7.32 (m, 2H, Ar-H), 7.26−7.18 (m, 4H, Ar-H), 6.30 (dd, *J* = 15.9, 7.4 Hz, 1H, CH), 6.20 (d, *J* = 16.0 Hz, 1H, CH), 4.32−4.25 (m, 2H, Si-H), 2.30−2.20 (m, 1H, CH), 1.28 (d, 3H, *J* = 7.1 Hz, CH_3); ^{13}C NMR (101 MHz, $CDCl_3$) δ 136.5 (1C, Ar-C), 135.7 (2C, Ar-C), 133.8 (1C, Ar-C), 132.1 (1C, Ar-C), 130.9 (1C, Ar-C), 129.9 (1C, Ar-C), 128.6 (2C, Ar-C), 128.0 (2C, Ar-C), 126.9 (2C, Ar-C), 126.0 (1C, Ar-C), 23.0 (1C, CH), 15.0 (1C, CH_3); ^{29}Si NMR (79 MHz, $CDCl_3$) δ -24.1. HRMS (EI) calcd for [M, $C_{16}H_{17}ClSi$]$^+$: 272.0788; found 272.0783.

(*E*)-Phenyl(4-phenylpent-3-en-2-yl)silane (14g)

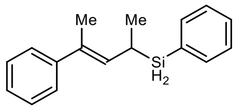

93% yield (117.4 mg), colorless oil. ^1H NMR (400 MHz, $CDCl_3$) δ 7.61−7.55 (m, 2H, Ar-H), 7.44−7.26 (m, 7H, Ar-H), 7.23−7.17 (m, 1H, Ar-H), 5.64 (dq, *J* = 10.7, 1.3 Hz, 1H, CH), 4.29 (dd, *J* = 6.7, 3.0 Hz, 1H, Si-H), 4.26 (dd, *J* = 6.7, 3.0 Hz, 1H, Si-H), 2.47−2.36 (m, 1H, CH), 1.86 (d, *J* = 1.2 Hz, 3H, CH_3), 1.25 (d, *J* = 7.1 Hz, 3H, CH_3); ^{13}C NMR (101 MHz, $CDCl_3$) δ 143.9 (1C, Ar-C), 135.7 (2C, Ar-C), 132.6 (1C, Ar-C), 131.6 (1C, Ar-C), 130.9 (1C, Ar-C), 129.7 (1C, Ar-C), 128.1 (2C, Ar-C), 127.9 (2C, Ar-C), 126.3 (1C, Ar-C), 125.5 (2C, Ar-C), 19.9 (1C, CH), 16.6 (1C, CH_3), 16.1 (1C, CH_3); ^{29}Si NMR (79 MHz, $CDCl_3$) δ -27.5. HRMS (EI) calcd for [M, $C_{17}H_{20}Si$]$^+$: 252.1334; found 252.1332.

(*E*)-Phenyl(4-phenylhex-3-en-2-yl)silane (14h)

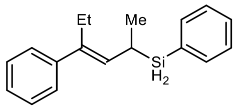

96% yield (127.9 mg), colorless oil. ^1H NMR (400 MHz, $CDCl_3$) δ 7.61−7.57 (m, 2H,

Ar-H), 7.44−7.34 (m, 3H, Ar-H), 7.32−7.26 (m, 4H, Ar-H), 7.23−7.18 (m, 1H, Ar-H), 5.49 (d, *J* = 10.7 Hz, 1H, CH), 4.31−4.26 (m, 2H, Si-H), 2.47−2.30 (m, 3H, CH, CH_2), 1.24 (d, *J* = 7.1 Hz, 3H, CH_3), 0.87 (t, *J* = 7.5 Hz, 3H, CH_3); ^{13}C NMR (101 MHz, $CDCl_3$) δ 143.0 (1C, Ar-C), 139.6 (1C, Ar-C), 135.7 (2C, Ar-C), 131.5 (1C, Ar-C), 130.5 (1C, Ar-C), 129.7 (1C, Ar-C), 128.1 (2C, Ar-C), 127.9 (2C, Ar-C), 126.3 (1C, Ar-C), 126.2 (2C, Ar-C), 23.0 (1C, CH_2), 19.5 (1C, CH), 16.8 (1C, CH_3), 13.4 (1C, CH_3); ^{29}Si NMR (79 MHz, $CDCl_3$) δ -25.1. HRMS (EI) calcd for [M, $C_{18}H_{22}Si$]$^+$: 266.1491; found 266.1488.

(4,4-Diphenylbut-3-en-2-yl)(phenyl)silane (14i)

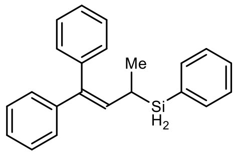

90% yield (141.5 mg), colorless oil. ^{1}H NMR (400 MHz, $CDCl_3$) δ 7.52−7.46 (m, 2H, Ar-H), 7.42−7.36 (m, 1H, Ar-H), 7.35−7.27 (m, 5H, Ar-H), 7.26−7.14 (m, 5H, Ar-H), 7.04−7.00 (m, 2H, Ar-H), 5.98 (d, *J* = 11.5 Hz, 1H, CH), 4.26 (dd, *J* = 6.7, 3.0 Hz, 1H, Si-H), 4.24 (dd, *J* = 6.7, 3.0 Hz, 1H, Si-H), 2.33−2.22 (m, 1H, CH), 1.22 (d, *J* = 7.2 Hz, 3H, CH_3); ^{13}C NMR (101 MHz, $CDCl_3$) δ 142.6 (1C, Ar-C), 140.2 (1C, Ar-C), 139.8 (1C, Ar-C), 135.7 (2C, Ar-C), 132.0 (1C, Ar-C), 131.2 (1C, Ar-C), 129.8 (2C, Ar-C), 129.7 (1C, Ar-C), 128.2 (2C, Ar-C), 128.1 (2C, Ar-C), 127.9 (2C, Ar-C), 126.9 (2C, Ar-C), 126.8 (1C, Ar-C), 126.6 (1C, Ar-C), 20.7 (1C, CH), 16.8 (1C, CH_3); ^{29}Si NMR (79 MHz, $CDCl_3$) δ -25.2. HRMS (EI) calcd for [M, $C_{22}H_{22}Si$]$^+$: 314.1491; found 314.1492.

Hydrosilylation of 1-alkyl ethylenes

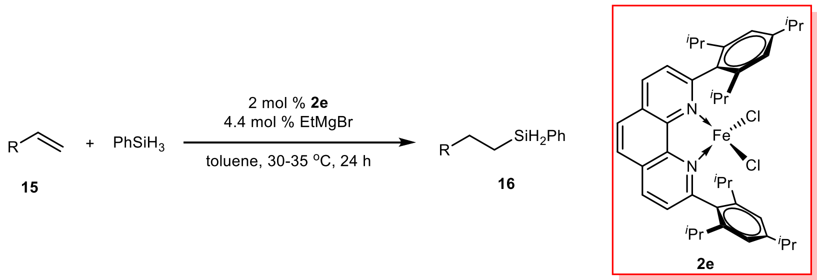

In an argon-filled glovebox, a vial (10 mL) was charged with alkene **15** (0.5 mmol), $PhSiH_3$ (59.5 mg, 0.55 mmol), dry anhydrous toluene (1 mL) and complexes **2e** (7.1

mg, 0.01 mmol). The reaction mixture was stirred at 30–35 °C for 3 minutes, then EtMgBr (1 M in THF, 22 μL, 0.022 mmol, 4.4 mol %) was added. After 24 hours, the vial was removed from the glovebox and the reaction mixture was concentrated by rotating evaporation. The residue was purified by flash chromatography to afford the desired product.

A procedure with low catalyst loading

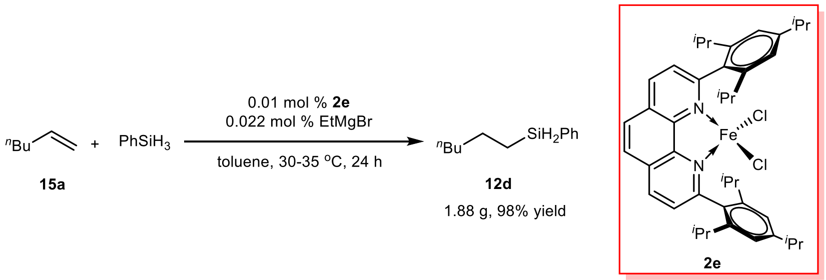

The **2e** (0.7 mg, 0.001 mmol, 0.01 mol %) was introduced into an oven-dried 15 mL sealed tube in an argon-filled glovebox. After toluene (2 mL), 1-hexene (0.842 g, 10.0 mmol), and phenylsilane (1.082 g, 10.0 mmol) were introduced into the mixture, the resulting mixture was stirred at room temperature for 5 min. Then EtMgBr (1M in THF, 2.2 μL, 0.0022 mmol, 0.022 mol %) was injected into the mixture with a microinjector. The ^{1}H NMR spectrum showed that the reaction accomplished in 24 hours. The reaction mixture was concentrated and purified by a flash chromatography on silica gel (hexane) to give **12d** as colorless oil, 1.88 g, 98% yield.

Octyl(phenyl)silane (16b)[25]

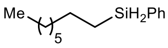

94% yield (103.6 mg), colorless oil. ^{1}H NMR (400 MHz, $CDCl_3$) δ 7.60−7.53 (m, 2H), 7.42−7.32 (m, 3H), 4.28 (t, J = 3.6 Hz, 2H), 1.50−1.41 (m, 2H), 1.40−1.20 (m, 10H), 0.97−0.91 (m, 2H), 0.88 (t, J = 6.7 Hz, 3H, CH_3); ^{13}C NMR (101 MHz, $CDCl_3$) δ 135.2, 132.8, 129.4, 127.9, 32.8, 31.9, 29.2, 29.2, 25.1, 22.7, 14.1, 10.0; ^{29}Si NMR (79 MHz, $CDCl_3$) δ -30.9; HRMS (EI) calcd for [M, $C_{14}H_{24}Si]^+$: 220.1647; found 220.1640.

(2-Cyclohexylethyl)(phenyl)silane (16c)[22]

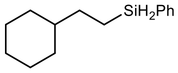

92% yield (100.5 mg), colorless oil. ^{1}H NMR (400 MHz, CDCl$_3$) δ 7.62−7.50 (m, 2H), 7.44−7.32 (m, 3H), 4.31−4.24 (m, 2H), 1.78−1.60 (m, 5H), 1.38−1.28 (m, 2H), 1.27−1.07 (m, 4H), 0.97−0.76 (m, 4H); ^{13}C NMR (101 MHz, CDCl$_3$) δ 135.2, 132.8, 129.4, 127.9, 40.2, 32.9, 32.6, 26.7, 26.4, 7.1; ^{29}Si NMR (79 MHz, CDCl$_3$) δ -30.1; HRMS (EI) calcd for [M, $C_{14}H_{22}Si$]$^+$: 218.1491; found 218.1490.

(3,3-Dimethylbutyl)(phenyl)silane (16d)[25]

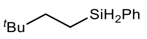

90% yield (86.6 mg), colorless oil. ^{1}H NMR (400 MHz, CDCl$_3$) δ 7.60−7.52 (m, 2H), 7.42−7.32(m, 3H), 4.30−4.25 (m, 2H), 1.35−1.26 (m, 2H), 0.91−0.83 (m, 9H); ^{13}C NMR (101 MHz, CDCl$_3$) δ 135.2, 132.7, 129.5, 127.9, 39.1, 31.3, 28.7, 4.5; ^{29}Si NMR (79 MHz, CDCl$_3$) δ -30.9; HRMS (EI) calcd for [M, $C_{12}H_{20}Si$]$^+$: 192.1334; found 192.1338.

Phenyl(3-phenylpropyl)silane (16e)[25]

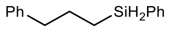

92% yield (104.1 mg), colorless oil. ^{1}H NMR (400 MHz, CDCl$_3$) δ 7.58−7.52 (m, 2H), 7.42−7.30 (m, 3H), 7.28−7.23 (m, 2H), 7.18−7.12 (m, 3H), 4.30 (t, J = 3.2 Hz, 2H), 2.60 (t, J = 7.6 Hz, 2H), 1.83−1.73 (m, 2H), 1.02−0.93 (m, 2H); ^{13}C NMR (101 MHz, CDCl$_3$) δ 142.6, 135.2, 132.4, 129.5, 128.4, 128.2, 128.0, 125.6, 38.9, 27.0, 9.8; ^{29}Si NMR (79 MHz, CDCl$_3$) δ -30.9; HRMS (EI) calcd for [M, $C_{15}H_{18}Si$]$^+$: 226.1178; found 226.1180.

Phenyl(4-phenylbutyl)silane (16f)[24]

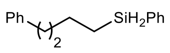

92% yield (110.6 mg), colorless oil. ^{1}H NMR (400 MHz, CDCl$_3$) δ 7.59−7.55 (m, 2H), 7.42−7.35 (m, 3H), 7.31−7.25 (m, 2H), 7.21−7.14 (m, 3H), 4.30 (t, J = 3.2 Hz, 2H), 2.65−2.59 (m, 2H), 1.75−1.66 (m, 2H), 1.57−1.48 (m, 2H), 1.03−0.96 (m, 2H); ^{13}C NMR (101 MHz, CDCl$_3$) δ 142.6, 135.2, 132.6, 129.5, 128.4, 128.2, 128.0, 125.6, 35.6, 34.6, 24.7, 9.9; ^{29}Si NMR (79 MHz, CDCl$_3$) δ -30.9; HRMS (EI) calcd for [M, $C_{16}H_{20}Si$]$^+$: 240.1334; found 240.1336.

(6-Chlorohexyl)(phenyl)silane (16g)[25]

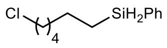

93% yield (105.5 mg), colorless oil. ^{1}H NMR (400 MHz, $CDCl_3$) δ 7.61−7.53 (m, 2H), 7.43−7.32 (m, 3H), 4.28 (t, *J* = 3.6 Hz, 2H), 3.50 (t, *J* = 6.7 Hz, 2H, $ClCH_2$), 1.81−1.71 (m, 2H), 1.53−1.34 (m, 6H), 0.99−0.91 (m, 2H); ^{13}C NMR (101 MHz, $CDCl_3$) δ 135.3, 132.7, 129.5, 128.1, 45.1, 32.6, 32.1, 26.6, 25.0, 9.9; ^{29}Si NMR (79 MHz, $CDCl_3$) δ -30.9; HRMS (EI) calcd for [M, $C_{12}H_{19}ClSi]^+$: 226.0945; found 226.0940.

Trimethyl(3-(phenylsilyl)propyl)silane (16h)[25]

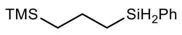

91.2 mg (82% yield), colorless oil. ^{1}H NMR (400 MHz, $CDCl_3$) δ 7.61−7.53 (m, 2H), 7.42−7.33 (m, 3H), 4.28 (t, *J* = 3.7 Hz, 2H), 1.55−1.46 (m, 2H), 1.05−0.97 (m, 2H), 0.66−0.58 (m, 2H), -0.03 (s, 9H, $Si(CH_3)_3$); ^{13}C NMR (101 MHz, $CDCl_3$) δ 135.2, 132.8, 129.4, 127.9, 20.5, 19.7, 14.3, -1.6; ^{29}Si NMR (79 MHz, $CDCl_3$) δ 0.48, -30.2; HRMS (EI) calcd for [M, $C_{12}H_{22}Si_2]^+$: 222.1260; found 226.1258.

Trimethyl((6-(phenylsilyl)hexyl)oxy)silane (16i)

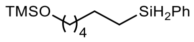

93% yield (130.5 mg), colorless oil. ^{1}H NMR (400 MHz, $CDCl_3$) δ 7.58−7.53 (m, 2H), 7.42−7.33 (m, 3H), 4.28 (t, *J* = 3.7 Hz, 2H, Si-H), 3.55 (t, *J* = 6.7 Hz, 2H), 1.56−1.28 (m, 8H), 0.98−0.89 (m, 2H), 0.10 (s, 9H, $OSi(CH_3)_3$); ^{13}C NMR (101 MHz, $CDCl_3$) δ 135.2, 132.7, 129.4, 128.0, 62.7, 32.6, 32.6, 29.7, 25.5, 25.0, 10.0, -0.5. ^{29}Si NMR (79 MHz, $CDCl_3$) δ 16.7, -30.8; HRMS (EI) calcd for [M, $C_{15}H_{28}OSi_2]^+$: 280.1679; found 280.1680.

4-(6-(Phenylsilyl)hexyl)morpholine (16j)

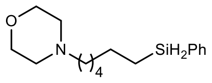

90% yield (124.9 mg) (used 5 mol % **2e** and 11 mol % EtMgBr), colorless oil. ^{1}H NMR (400 MHz, $CDCl_3$) δ 7.60−7.52 (m, 2H), 7.43−7.30 (m, 3H), 4.27 (t, *J* = 3.7 Hz, 2H,

Si-H), 3.72 (t, J = 4.8 Hz, 4H), 2.50−2.35 (m, 6H), 2.31 (t, J = 7.8 Hz, 2H), 1.52−1.24 (m, 8H), 0.98−0.89 (m, 2H); ^{13}C NMR (101 MHz, $CDCl_3$) δ 135.2, 132.7, 129.5, 127.9, 66.9, 59.1, 53.7, 32.7, 27.1, 26.3, 25.0, 10.0; ^{29}Si NMR (79 MHz, $CDCl_3$) δ -30.9; HRMS (EI) calcd for [M, $C_{16}H_{27}NOSi$]$^+$: 277.1862; found 277.1860.

(4-(2-Methyl-1,3-dioxolan-2-yl)butyl)(phenyl)silane (16k)[25]

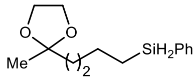

89% yield (111.4 mg), colorless oil. ^{1}H NMR (400 MHz, $CDCl_3$) δ 7.61−7.53 (m, 2H), 7.42−7.33 (m, 3H), 4.32−4.25 (m, 2H, Si-H), 3.97−3.86 (m, 4H), 1.70−1.58 (m, 2H), 1.50−1.40 (m, 4H), 1.29 (s. 3H), 0.98−0.90 (m, 2H); ^{13}C NMR (101 MHz, CDCl3) δ 135.2, 132.6, 129.5, 127.9, 110.0, 64.6, 38.8, 27.3, 25.3, 23.7, 10.0; ^{29}Si NMR (79 MHz, $CDCl_3$) δ -29.9; HRMS (EI) calcd for [M, $C_{14}H_{22}O_2Si$]$^+$: 250.1389; found 250.1392.

(2*R*)-2-((*R*)-(6-Methoxyquinolin-4-yl)((trimethylsilyl)oxy)methyl)-5-(2-(phenylsilyl)ethyl)quinuclidine (16l)

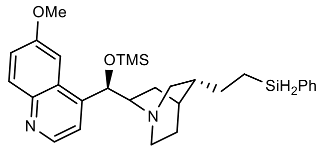

72% yield (181.7 mg) (used 5 mol % **2e** and 11 mol % EtMgBr), white solid. ^{1}H NMR (400 MHz, $CDCl_3$) δ 8.75 (d, J = 4.4 Hz, 1H), 8.04 (d, J = 9.2 Hz, 1H), 7.66−7.60 (m, 1H), 7.50−7.31 (m, 4H), 7.36−7.31 (m, 1H), 7.29−7.24 (m, 2H), 4.20 (t, J = 7.1 Hz, 2H, Si-H), 4.17 (s, 3H), 4.10−3.96 (m, 1H), 3.50−3.37 (m, 1H), 3.27−3.18 (m, 1H), 3.14−3.02 (m, 1H), 2.77−2.66 (m, 1H), 2.28−2.16 (m, 1H), 2.12−2.02 (m, 2H), 2.20−1.85 (m, 1H), 1.48 (d, J = 7.2 Hz, 1H), 1.42−1.31 (m, 3H), 1.26 (t, J = 7.1 Hz, 1H), 0.88−0.78 (m, 2H), 0.05 (s, 9H, $Si(CH_3)_3$) ; ^{13}C NMR (101 MHz, $CDCl_3$) δ 159.1, 146.8, 146.7, 144.5, 134.9, 134.9, 131.7, 129.8, 128.0, 125.8, 123.1, 118.9, 100.5, 60.2, 56.8, 55.8, 43.0, 36.5, 29.3, 25.3, 24.7, 18.2, 12.6, 7.6, 0.2; ^{29}Si NMR (79 MHz, $CDCl_3$) δ 19.4, -31.2; HRMS (EI) calcd for [M, $C_{29}H_{40}N_2O_2Si_2$]$^+$: 504.2628; found 504.2633.

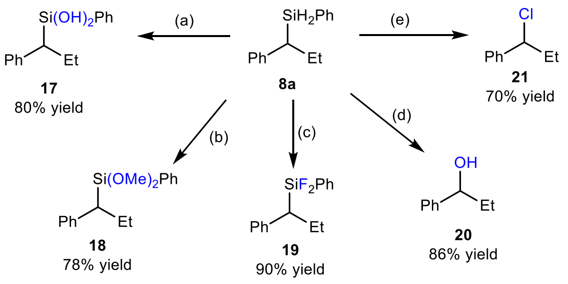

Supplementary Figure 1 |Transformations of hydrosilylation product 8a. Conditions: (a) 1 mol% Pd/C, dioxane/phosphate buffer, rt, 12 h. (b) 0.5 mol% $[RuCl_2(p\text{-cymene})]_2$, MeOH, 0 °C, 1 h. (c) 1 mol% CuI, $CuCl_2$, KF, Et_2O, rt, 8 h. (d) $KHCO_3$, H_2O_2 (30% aq.), MeOH/THF = 1:1 (v/v), 50 °C, 12 h. (e) N-chlorosuccinimide, tetra-n-butylammonium fluoride, DCM, 50 °C. 12 h.

Synthesis of phenyl(1-phenylpropyl)silanediol (17)[26]

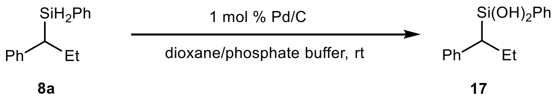

To a mixture of Pd/C (0.01 mmol, 10% wt, 1 mol %) dioxane (5 mL), phosphate buffer (1 mL) was added **8a** (1 mmol) under an air atmosphere. The reaction mixture was stirred at room temperture for 12 h and the progress was monitored by TLC. The mixture was extracted with EtOAc and then the organic layer was separated and washed with brine, dried over Na_2SO_4 and concentrated in vacuo. The residue was purified by column chromatography using petroleum ether/EtOAc = 10:1 as the eluent to give 0.207 g (0.80 mmol, 80% yield) of **17** as white soild. ^{1}H NMR (400 MHz, $CDCl_3$) δ 7.59−7.53 (m, 2H, Ar-H), 7.45−7.40 (m, 1H, Ar-H), 7.36 (t, J = 7.5 Hz, 2H, Ar-H), 7.28−7.22 (m, 2H, Ar-H), 7.17−7.08 (m, 3H, Ar-H), 2.56 (s, 1H, OH), 2.53 (s, 1H, OH), 2.24 (dd, J = 11.1, 4.2 Hz, 1H, CH), 1.98−1.76 (m, 2H, CH_2), 0.82 (t, J = 7.2 Hz, 3H, CH_3). ^{13}C NMR (101 MHz, $CDCl_3$) δ 141.3 (1C, Ar-C), 134.3 (2C, Ar-C), 133.8 (1C, Ar-C), 130.4 (1C, Ar-C), 128.5 (2C, Ar-C), 128.3 (2C, Ar-C), 127.8 (2C, Ar-C), 125.2 (1C, Ar-C), 38.3 (1C, CH), 22.3 (1C, CH_2), 13.8 (1C, CH_3). HRMS (EI) calcd for [M, $C_{15}H_{18}O_2Si]^+$: 258.1076; found 258.1066.

Synthesis of dimethoxy(phenyl)(1-phenylpropyl)silane (18)[27]

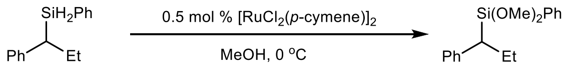

8a **18**

To a mixture of MeOH (5 mmol) and $[RuCl_2(p\text{-cymene})]_2$ (0.01 mmol, 0.5 mol %) was added **8a** (2 mmol) at 0 °C (ice bath) under an air atmosphere. The reaction mixture was stirred at 0 °C for 10 min and the progress was monitored by GC-MS. The mixture was added hexane and filtered. The desired product **18** was obtained in 78% isolated yield (446.8 mg) by concentrating the filtrate in vacuo. ^{1}H NMR (400 MHz, $CDCl_3$) δ 7.48−7.38 (m, 3H, Ar-H), 7.33 (t, J = 7.2 Hz, 2H, Ar-H), 7.19 (t, J = 7.4 Hz, 2H, Ar-H), 7.11 (t, J = 7.1 Hz, 1H, Ar-H), 7.03 (d, J = 7.5 Hz, 2H, Ar-H), 3.53 (s, 3H, Me), 3.50 (s, 3H, Me), 2.25 (dd, J = 11.7, 3.7 Hz, 1H, CH), 2.00−1.90 (m, 1H, CH_2), 1.82−1.70 (m, 1H, CH_2), 0.81 (t, J = 7.2 Hz, 3H, CH_3). ^{13}C NMR (101 MHz, $CDCl_3$) δ 141.3 (1C, Ar-C), 134.9 (2C, Ar-C), 131.7 (1C, Ar-C), 130.1 (1C, Ar-C), 128.8 (2C, Ar-C), 128.0 (2C, Ar-C), 127.7 (2C, Ar-C), 124.8 (1C, Ar-C), 51.1 (1C, OMe), 51.0 (1C, OMe) 36.9 (1C, CH), 22.8 (1C, CH_2), 13.7 (1C, CH_3). HRMS (EI) calcd for $[M, C_{17}H_{22}O_2Si]^+$: 286.1389; found 286.1385.

Synthesis of difluoro(phenyl)(1-phenylpropyl)silane (19)

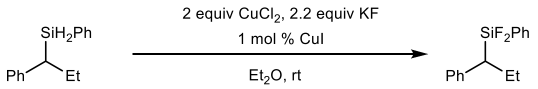

8a **19**

To a Schlenk tube (15 mL) which was charged with $CuCl_2$ (4 mmol), KF (2.2 mmol), CuI (0.01 mmol) and Et_2O (2 mL), **8a** (1 mmol) was added. Then the mixture was stirred at room temperature. After 8 h, the mixture was added hexane (10 mL) and filtered. The desired product **19** was obtained in 90% isolated yield (236.2 mg) by concentrating the filtrate in vacuo. ^{1}H NMR (400 MHz, $CDCl_3$) δ 7.50−7.44 (m, 1H, Ar-H), 7.44−7.38 (m, 2H, Ar-H), 7.34 (t, J = 7.5 Hz, 2H, Ar-H), 7.28−7.23 (m, 2H, Ar-H), 7.20−7.15 (m, 1H, Ar-H), 7.14−7.09 (m, 2H, Ar-H), 2.42 (dtd, J = 10.5, 5.2, 2.4 Hz, 1H, CH), 2.06 (dqd, J = 14.5, 7.3, 5.0 Hz, 1H, CH_2), 1.80−1.98 (m, 1H, CH_2), 0.93 (t, J = 7.3 Hz, 3H, CH_3). ^{13}C NMR (101 MHz, $CDCl_3$) δ 138.3 (1C, Ar-C), 134.1 (2C, Ar-

C), 131.7 (1C, Ar-C), 130.1 (1C, Ar-C), 128.7 (2C, Ar-C), 128.6 (2C, Ar-C), 128.0 (2C, Ar-C), 126.0 (1C, Ar-C), 35.9 (t, J = 19.8 Hz, 1C, CH), 22.0 (1C, CH_2), 13.5 (1C, CH_3). ^{19}F NMR (376 MHz, $CDCl_3$) δ -144.9 (d, J = 21.0 Hz), -145.2 (d, J = 21.0 Hz). HRMS (EI) calcd for [M, $C_{15}H_{16}F_2Si]^+$: 262.0989; found 262.0994.

Synthesis of 1-phenylpropan-1-ol (20)

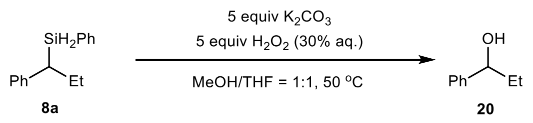

To a solution of **8a** (1 mmol, 1.0 equiv) in MeOH and THF (6 mL, MeOH/THF = 1:1, v/v), K_2CO_3 (0.69 g, 5 mmol, 5.0 equiv) and hydrogen peroxide (0.6 mL, 30% aqueous solution) were added insequencely. Then the mixture was stirred at 50 °C. After 8 h, the mixture was etracted with EtOAc and then the organic layer was separated and washed with brine, dried over Na_2SO_4 and concentrated in vacuo. The residue was purified by column chromatography using petroleum ether/EtOAc = 10:1 as the eluent to give 117 mg (0.86 mmol, 86% yield) of the desired product **20**, a known compound[28] as a colorless oil. ^1H NMR (400 MHz, $CDCl_3$) δ 7.36−7.29 (m, 5H), 4.56 (t, J = 6.6 Hz, 2H), 2.12 (s, 1H), 1.83−1.72 (m 1H), 0.92 (t, 3H, J = 6.2 Hz); ^{13}C NMR (101 MHz, $CDCl_3$) δ 144.6, 128.1, 127.5, 126.0, 76.0, 31.9, 10.2.

Synthesis of (1-chloropropyl)benzene (19)[29]

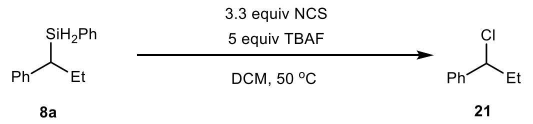

To a Schlenk tube (25 mL) which was charged with *N*-chlorosuccinimide (NCS, 3 mmol), tetra-*n*-butylammonium fluoride (TBAF, 5mmol) and DCM (5 mL), **8a** (1 mmol) was added. Then the mixture was stirred at 50 °C for 8 h and concentrated in vacuo, purified by column chromatography using petroleum ether as the eluent to give 108 mg (0.70 mmol, 70% yield) of the desired product **21**, a known compound[30] as a colorless oil. ^1H NMR (400 MHz, $CDCl_3$) δ 7.40−7.25 (m, 5H), 4.78 (dd, J = 7.8 Hz, J

= 6.8 Hz, 1H), 2.22−1.98 (m, 2H), 0.99 (t, *J* = 7.4 Hz, 3H); ^{13}C NMR (101 MHz, $CDCl_3$) δ 141.8, 128.6, 128.2, 127.1, 65.5, 33.3, 11.8.

Deuteration experiments

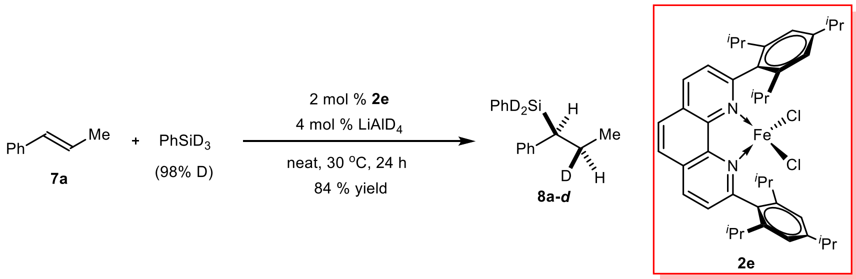

In an argon-filled glovebox, a vial (10 ml) was charged with alkene (**7a**, 52.1 mg, 0.5 mmol), $PhSiD_3$ (61.1 mg, 0.55 mmol) and complex **2e** (0.025 mmol). The reaction mixture was stirred at room temperature (30–35 °C) for 3 minutes, then the $LiAlD_4$ solution (1 M in THF) was added to the reaction mixture. After 24 hours, the vial was removed from the glovebox and the reaction mixture was concentrated under dynamic vacuum. The residue was purified by flash chromatography to afford **8a-*d***, colorless oil, 96.3 mg (84% yield). ^{1}H NMR (400 MHz, $CDCl_3$) δ 7.40−7.34 (m, 3H, Ar-H), 7.32−7.20 (m, 4H, Ar-H), 7.14−7.09 (m, 1H, Ar-H), 7.07−7.03 (m, 2H, Ar-H), 4.34−4.30 (m, 0.07H, Si-H), 2.34 (d, *J* = 10.3 Hz, 1H, CH), 1.89−1.78 (m, 1.02H, CHD), 0.90 (d, *J* = 7.2 Hz, 3H, CH_3); ^{13}C NMR (101 MHz, $CDCl_3$) δ 142.8 (1C, Ar-C), 135.7 (2C, Ar-C), 131.4 (1C, Ar-C), 129.7 (1C, Ar-C), 128.4 (2C, Ar-C), 127.9 (2C, Ar-C), 127.8 (2C, Ar-C), 125.0 (1C, Ar-C), 34.2 (1C, CH), 24.2 (t, *J* =19.7 Hz, 1C, CHD), 13.8 (1C, CH_3).

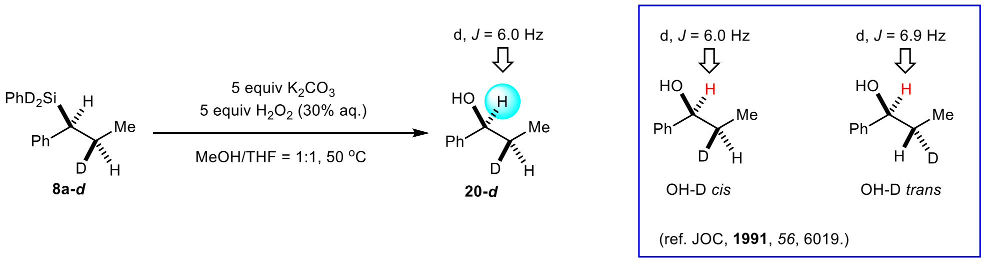

Supplementary Figure 2 | Determination of the relative configuration of 8a-*d*. Compound **8a-*d*** was oxidized to the corresponding alcohol *cis*-[2-D]-1-phenylpropan-1-ol (**20-*d***) using the procedure for the preparation of **20** (*vide infra*). The relative

configuration of **20-*d*** was determined through comparing its ^{1}H NMR with literature data[31]. ^{1}H NMR (400 MHz, $CDCl_3$) δ 7.50−7.18 (m, 5H), 4.60 (d, J = 6.0 Hz, 1H), 1.75(m, 1H), 1.80 (br, 1H), 0.92 (d, J = 7.3 Hz, 3H).

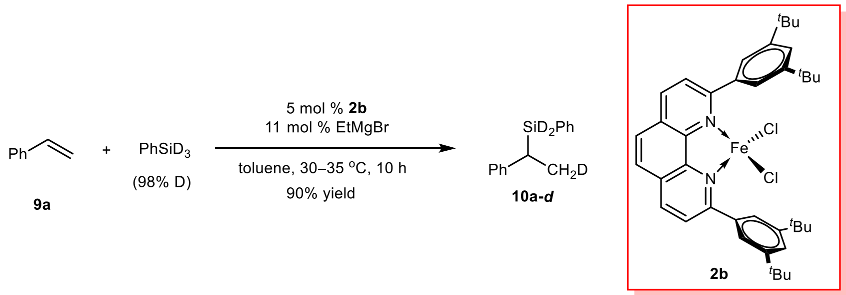

In an argon-filled glovebox, a vial (4 mL) was charged with styrene (**9a**, 52.1 mg, 0.5 mmol), $PhSiD_3$ (61.1 mg, 0.55 mmol), dry anhydrous toluene (1 mL) and complex **2b** (17.1 g, 0.025 mmol). The reaction mixture was stirred at room temperature (30–35 °C) for 3 minutes, then EtMgBr (1M in THF, 55 μL, 0.055 mmol, 11 mol %) was added to the reaction mixture. After 10 hours, the vial was removed from the glovebox and the reaction mixture was concentrated by rotating evaporation. The residue was purified by flash chromatography to afford **10a-*d***, colorless oil, 96.9 mg 90% yield. ^{1}H NMR (400 MHz, $CDCl_3$) δ 7.43−7.36 (m, 3H, Ar-H), 7.34−7.28 (m, 2H, Ar-H), 7.28−7.22 (m, 2H, Ar-H), 7.15−7.07 (m, 3H, Ar-H), 4.32−4.29 (m, 0.12H, Si-H), 2.61 (t, J = 7.3 Hz, 1H, CH), 1.22 (dt, J = 7.3, 1.8 Hz, 1.95H, CH_2D); ^{13}C NMR (101 MHz, $CDCl_3$) δ 144.5 (1C, Ar-C), 135.7 (2C, Ar-C), 131.3 (1C, Ar-C), 129.8 (1C, Ar-C), 128.4 (2C, Ar-C), 127.9 (2C, Ar-C), 127.1 (2C, Ar-C), 125.0 (1C, Ar-C), 25.1 (1C, CH), 16.2 (CH_2D), 16.0 (CH_2D), 15.8 (CH_2D).

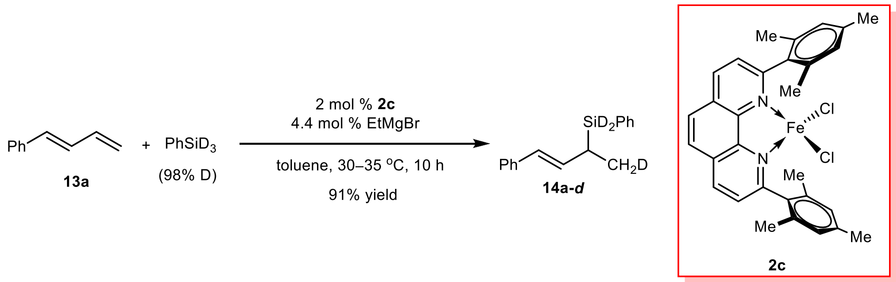

In an argon-filled glovebox, a vial (4 mL) was charged with (E)-buta-1,3-dien-1-ylbenzene (**13a**, 65.1 mg, 0.5 mmol), $PhSiD_3$ (61.1 mg, 0.55 mmol), dry anhydrous toluene (1 mL) and complex **2c** (0.01 mmol). The reaction mixture was stirred at room

temperature (30−35 °C) for 3 minutes, then EtMgBr (1 M in THF, 22 μL, 0.022 mmol, 4.4 mol %) was added to the reaction mixture. After 10 hours, the vial was removed from the glovebox and the reaction mixture was concentrated by rotating evaporation. The residue was purified by flash chromatography to afford **14a-*d***, colorless oil, 109.8 mg, 91% yield. ^{1}H NMR (400 MHz, $CDCl_3$) δ 7.61−7.54 (m, 2H, Ar-H), 7.44−7.33 (m, 3H, Ar-H), 7.33−7.24 (m, 4H, Ar-H), 7.20−7.14 (m, 1H, Ar-H), 6.33 (dd, J = 15.9, 7.0 Hz, 1H, CH), 6.26 (d, J = 16.0 Hz, 1H, CH), 4.30−4.26 (m, 0.03H, Si-H), 2.25 (q, J = 7.0 Hz, 1H, CH), 1.30−1.25 (m, 2.03H, CH_2D); ^{13}C NMR (101 MHz, $CDCl_3$) δ 138.2 (1C, Ar-C), 135.8 (2C, Ar-C), 133.2 (1C, Ar-C), 130.0 (1C, Ar-C), 128.6 (2C, Ar-C), 128.1 (2C, Ar-C), 127.4 (1C, Ar-C), 126.7 (1C, Ar-C), 125.9 (2C, Ar-C), 22.8 (1C, CH), 15.1 (CH_2D), 14.9 (CH_2D), 14.7 (CH_2D).

Kinetic isotopic effect experiments

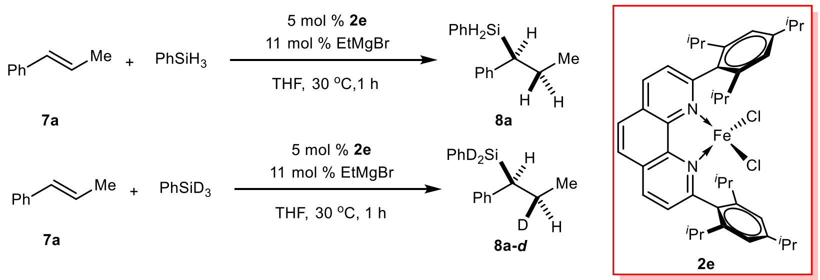

In an argon-filled glovebox, a vial (10 mL) was charged with **7a** (0.5 mmol), $PhSiH_3$ (59.5 mg, 0.55 mmol) or $PhSiD_3$ (60.0 mg, 0.55 mmol), and complex **2e** (17.8 mg, 0.025 mmol). The reaction mixture was stirred at 30 °C for 1 minute, then EtMgBr (1 M in THF, 55 μL, 0.055 mmol, 11 mol %) was added. After stirring for 1 hours at 30 °C, the vial was removed from the glovebox and quenched with 2 drops water. The reaction mixture was concentrated by rotating evaporation then remove the meatal salt by flash chromatography. The yield of **8a** and **8a-*d*** were measured by GC with n-dodecane as internal stander. We repeated the experiment three times, the average of k_H/k_D is 1.4.

Supplementary Table 5 | Kinetic isotopic effect experiments

entry	GC yield of **8a**	GC yield of **8a-*d***	k_H/k_D
1	20	14	1.4
2	20	17	1.2

3	21	14	1.5

Reduction experiments

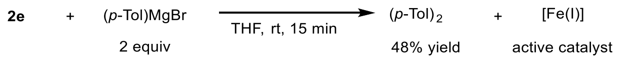

In an argon-filled glovebox, a bottle (25 mL) was charged with complex **2e** (142.3 mg, 0.2 mmol) and 5 mL THF. Then (*p*-Tol)MgBr (0.4 mL, 1.0 M in THF, 0.4 mmol) was added via syringe. the reaction mixture was stirred at room temperature (25–35 ^oC). After stirring for 15 min, the vial was removed from the glovebox and quenched with 2 drops water. The reaction mixture was concentrated by rotating evaporation. The residue was purified by flash chromatography to afford (*p*-Tol)$_2$ [17.5 mg, 48% based on (*p*-Tol)MgBr] and **1e** (102.8 mg, 88% yield based on **2e**).The catalyst prepared by this procedure can catalyzed the hydrosilylation of **7a** with $PhSiH_3$ and give the same results as those obtained in Table 1, entry 9. Extend the reduction time to 3 hours, the same results were obtained.

Electron paramagnetic resonance analysis

In an argon-filled glovebox, a bottle (25 mL) was charged with complex **2e** (284.6 mg, 0.4 mmol) and 5 mL THF. Then EtMgBr (0.8 mL, 1.0 M in THF, 0.8 mmol) was added via syringe. the reaction mixture was stirred at room temperature (25–35 ^oC). After stirring for 15 min, the solvent was pumped out through vacuum and the black red powder was obtained and subjected for EPR analysis.

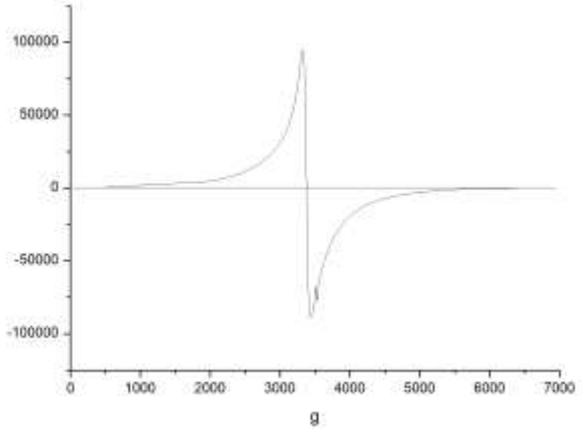

Supplementary Figure 3 | Electron paramagnetic resonance analysis of

complex 2e reduced by EtMgBr.

Supplementary Table 6 | Calculated energy of all the stationary points

Int-1	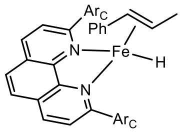	E= -2882.422546	E_{CPCM}= -2883.014985<br>E_{SMD}= -2883.042553
TS-1	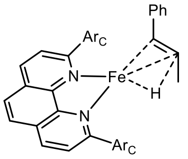	E= -2882.400667	E_{CPCM}= -2882.983872<br>E_{SMD}= -2883.012463
Int-2	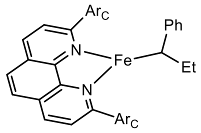	E= -2882.450896	E_{CPCM}= -2883.038002<br>E_{SMD}= -2883.068141
TS-2	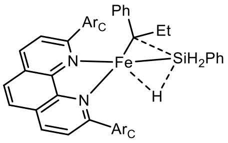	E= -3405.268228	E_{CPCM}= -3405.968771<br>E_{Smd}= -3405.999663
Pro	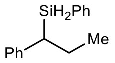	E= -871.725111	E_{CPCM}= -871.970325<br>E_{SMD}= -871.982514
Int-1′	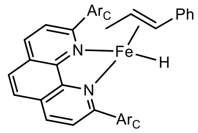	E= -2882.419634	E_{CPCM}= -2883.013254<br>E_{SMD}= -2883.040962
TS-1′	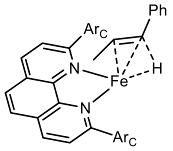	E= -2882.392142	E_{CPCM}= -2882.982082<br>E_{SMD}= -2883.010242
Int-2′	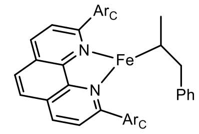	E= -2882.412161	E_{CPCM}= -2883.030251<br>E_{SMD}= -2883.060725
TS-2′	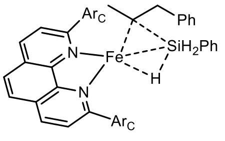	E= -3405.256232	E_{CPCM}= -3405.957609<br>E_{SMD}= -3405.989828
Pro′	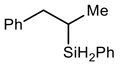	E= -871.721247	E_{CPCM}= -871.967294<br>E_{SMD}= -871.980259
Int-1" Low spin		E= -2882.384023	

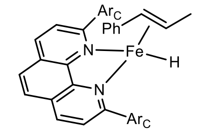

E_{CPCM}= -2882.975726
E_{SMD}= -2883.003232

Int-1''' Low spin

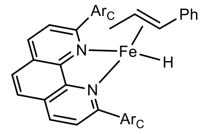

E= -2882.393703

E_{CPCM}= -2882.984630
E_{SMD}= -2883.010145

TS-3′

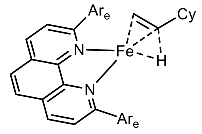

E= -3318.383056

E_{CPCM}= -3319.128515
E_{SMD}= -3319.159812

TS-3

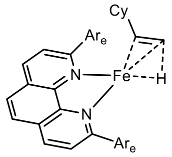

E= -3318.378433

E_{CPCM}= -3319.118136
E_{SMD}= -3319.150063

TS-4

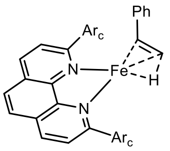

E= -2843.094458

E_{CPCM}= -2843.663574
E_{SMD}= -2843.691743

TS-4′

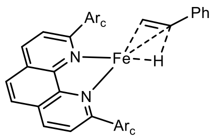

E= -2843.089139

E_{CPCM}= -2843.659316
E_{SMD}= -2843.687410

TS-5

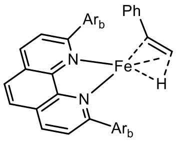

E= -3236.117388

E_{CPCM}= -3236.836134
E_{SMD}= -3236.866895

TS-5′

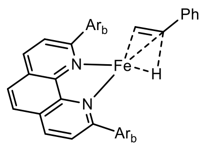

E= -3236.112393

E_{CPCM}= -3236.832401
E_{SMD}= -3236.863834

$PhSiH_3$

E= -522.831870

E_{CPCM}= -522.949872
E_{SMD}= -522.955351

trans-PhCH=CHMe

E= -348.839445

E_{CPCM}= -348.972558
E_{SMD}= -348.981083

Ar^b=3,5-(^{t}Bu)$_2$C$_6$H$_3$; Ar^c=2,4,6-(Me)$_3$C$_6$H$_2$; Ar^e=2,4,6-(^{i}Pr)$_3$C$_6$H$_2$

Supplementary Table 7 | Calculated stereo-structures shown in Table 5 of main text

Markovnikov	*anti*-Markovnikov
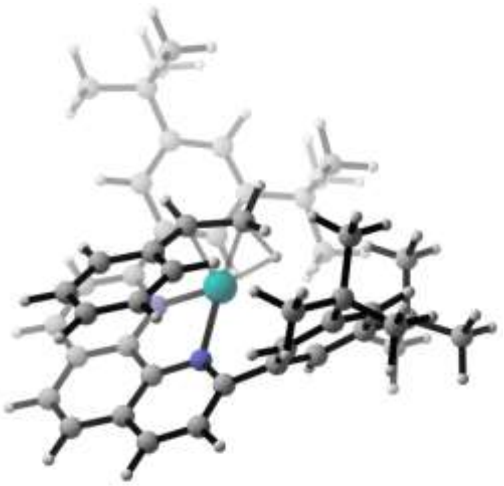 **TS-5** G= 0.0 kcal/mol	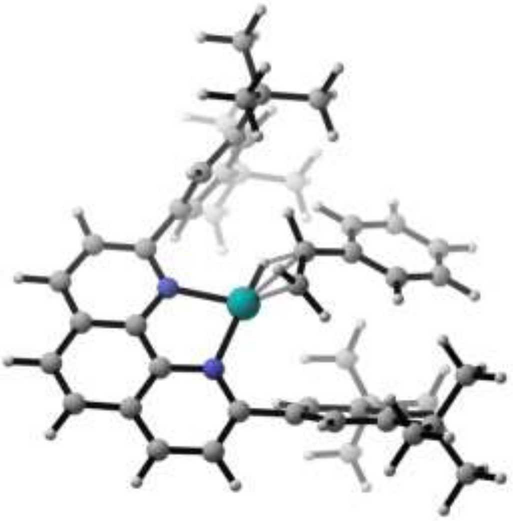 **TS-5′** G= +4.4 kcal/mol
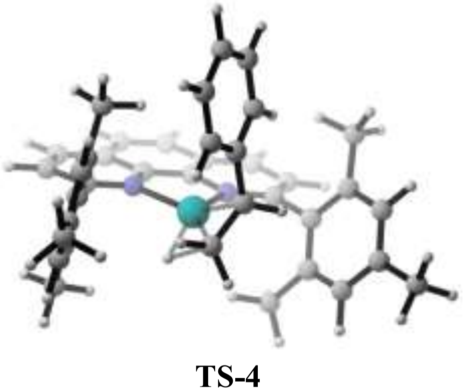 **TS-4** G= 0.0 kcal/mol	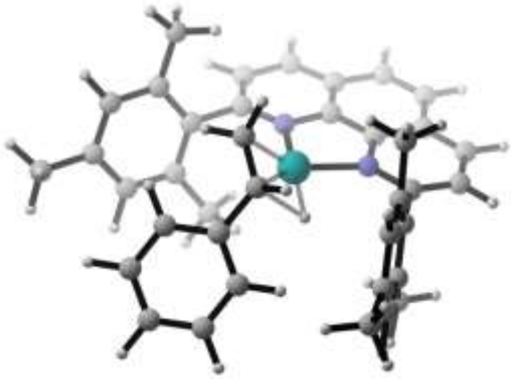 **TS-4′** G= +1.5 kcal/mol

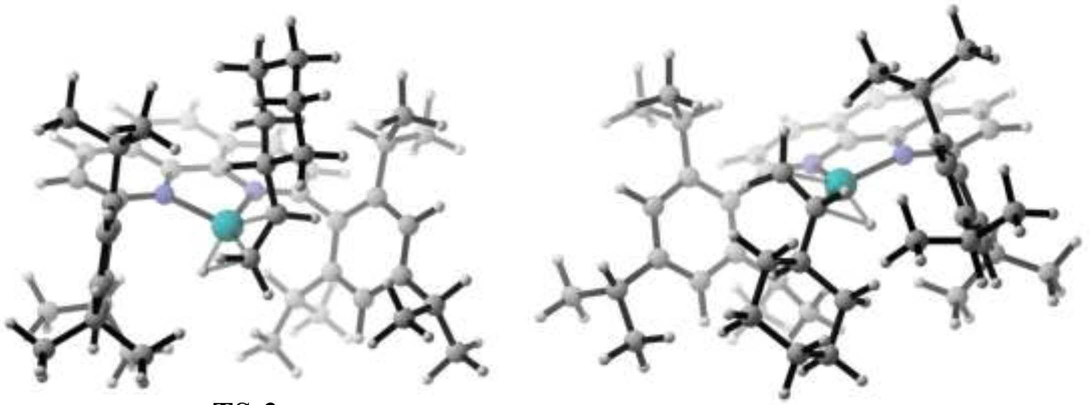

TS-3
G= 0.0 kcal/mol

TS-3′
G= -5.9 kcal/mol

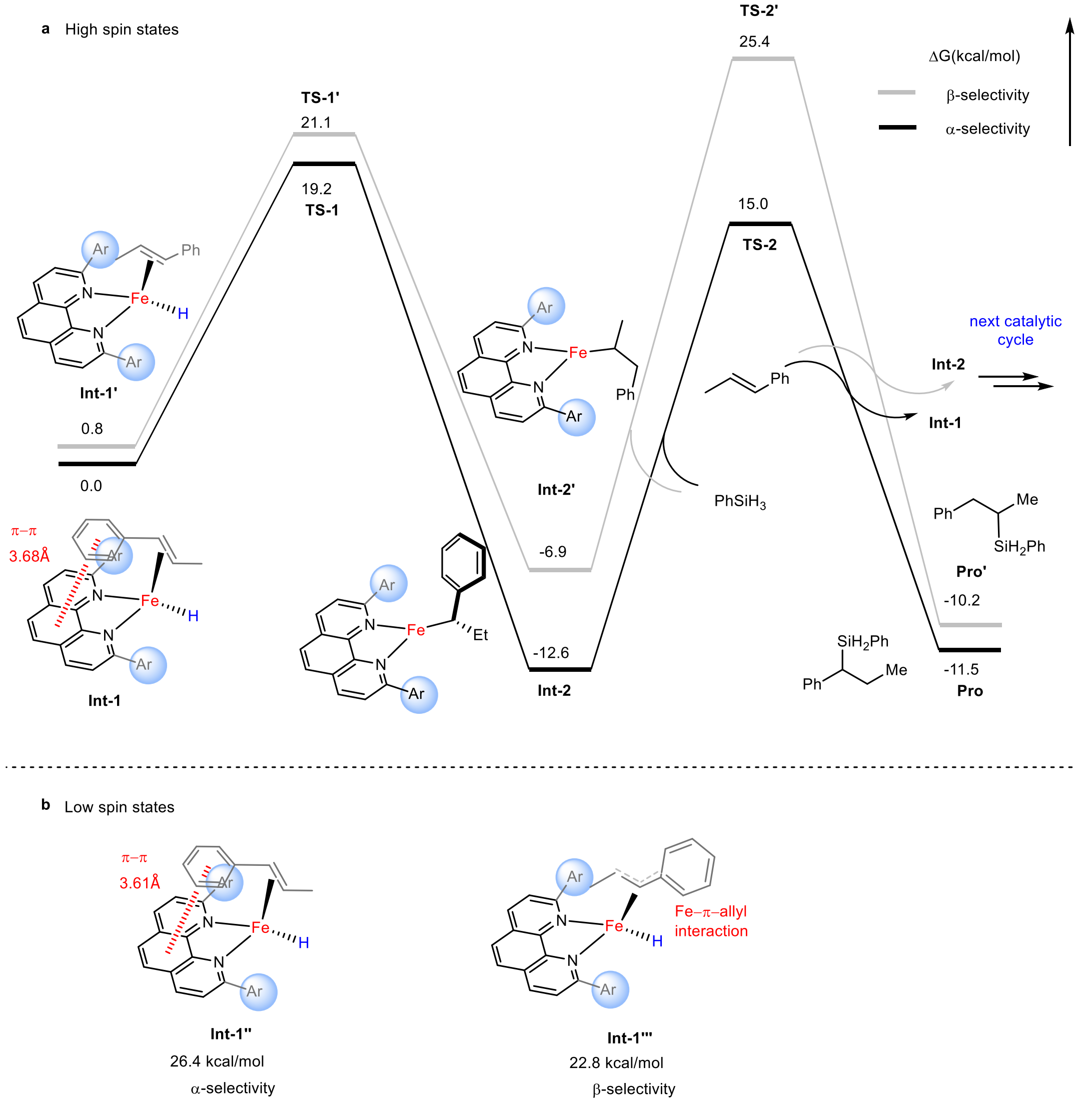

Supplementary Figure 4 | A: Calculated Energy Profiles of High Spin Fe(I) Catalyzed Alkene Hydrosilylation; B: Relative Energy and Key Interaction of Low Spin Fe(I) intermediates **Int 1”** and **Int 1’”**. Although the predicted π-π and Fe- π allyl interaction can be found in the calculated

low spin Fe(I) intermediates, the splitting energy of *d* orbital still dominate the total energy of Fe(I) intermediates and therefore the electrons tend to occupy all the *d* orbital rather than maximizing paired electrons.

Supplementary Table 8 | Int-1

Fe	-0.1804858	0.6304087	-0.0515285
N	1.3515982	-0.7628623	-0.8614925
N	-1.3388108	-0.9979853	-0.8889315
C	3.2100842	0.7724297	-0.6748615
C	2.8917692	1.7648087	-1.6220395
C	3.3502882	3.0626217	-1.4131755
H	3.0932512	3.8298707	-2.1399255
C	4.1145282	3.4053737	-0.3007355
C	4.4558672	2.3974707	0.5963945
H	5.0721592	2.6393067	1.4609005
C	4.0350972	1.0797327	0.4211325
C	2.6735602	-0.6030183	-0.8758205
C	3.5384612	-1.6889723	-1.1235155
H	4.6089572	-1.5153253	-1.1296225
C	3.0190742	-2.9398703	-1.3540745
H	3.6726772	-3.7883873	-1.5373155
C	1.6237672	-3.1151433	-1.3836715
C	0.9969562	-4.3739753	-1.6690795
H	1.6301042	-5.2394703	-1.8423785
C	-0.3521798	-4.4808693	-1.7346595
H	-0.8230618	-5.4332853	-1.9609385
C	-1.1926458	-3.3411803	-1.4985445
C	-2.5966258	-3.3969243	-1.5488785
H	-3.0907888	-4.3314623	-1.8002375
C	-3.3284658	-2.2642943	-1.2738345
H	-4.4124378	-2.2733913	-1.3095365
C	-2.6696458	-1.0684073	-0.9309705
C	-0.6088098	-2.1012883	-1.1770065
C	0.8306132	-1.9811023	-1.1345235
C	-3.4377308	0.1586847	-0.5767955
C	-4.1845258	0.1930407	0.6152425
C	-4.8381888	1.3724697	0.9631665
H	-5.3952968	1.4098417	1.8972925
C	-4.7943028	2.5042187	0.1493135
C	-4.0823068	2.4301387	-1.0437735
H	-4.0437958	3.2994427	-1.6965225

C	-3.3986898	1.2763587	-1.4256335
C	-4.3085418	-1.0094093	1.5214645
H	-4.6369898	-0.7069533	2.5198455
H	-5.0444858	-1.7256953	1.1359915
H	-3.3636658	-1.5474303	1.6317595
C	-2.6258418	1.2668827	-2.7189115
H	-2.6584638	0.2904877	-3.2126775
H	-3.0241578	2.0137587	-3.4122195
H	-1.5769428	1.5130697	-2.5145935
C	2.0834482	1.4642917	-2.8592375
H	2.2919202	2.2048587	-3.6372395
H	2.3020132	0.4720457	-3.2666175
H	1.0144742	1.5101857	-2.6229275
C	4.5235852	0.0476097	1.4118015
H	4.3991202	0.4077997	2.4387695
H	4.0024342	-0.9058523	1.3260495
H	5.5938652	-0.1423043	1.2636825
C	4.5914132	4.8197177	-0.0864315
H	4.4516312	5.1286787	0.9548235
H	5.6594742	4.9160927	-0.3160245
H	4.0478452	5.5233717	-0.7236005
C	-5.5260898	3.7613177	0.5464005
H	-6.6044818	3.6577927	0.3756925
H	-5.3820018	3.9818417	1.6091955
H	-5.1781788	4.6241907	-0.0289325
C	0.2004132	-1.4671633	2.0498375
C	-0.4622198	-2.6919583	1.8463745
C	0.2079602	-3.9076573	1.9159625
C	1.5718692	-3.9450703	2.1931615
C	2.2418112	-2.7441693	2.4261895
C	1.5689172	-1.5310483	2.3633185
H	-1.5239448	-2.6844563	1.6098845
H	-0.3378008	-4.8310243	1.7404415
H	2.1003992	-4.8923213	2.2440545
H	3.3015152	-2.7547293	2.6711225
H	2.1038772	-0.6086703	2.5632505
C	-0.5224938	-0.2048353	1.8815615
C	0.0040382	1.1112337	1.9290605
H	-1.6052148	-0.2875233	1.9856885
H	1.0686242	1.2451727	2.1303195
C	-0.8696338	2.2699427	2.3611045
H	-0.8679238	2.3821727	3.4556405
H	-0.5227078	3.2073877	1.9178765
H	-1.9088608	2.1202777	2.0442145

H	-0.1800228	2.1190857	-0.7885485

Supplementary Table 9 | TS-1

Fe	-0.1587261	0.2848175	-0.3929192
N	-1.4815741	1.7219685	0.0287158
N	1.1868869	1.8100745	0.0225828
C	-3.4458011	0.3355565	-0.3037752
C	-3.6961461	-0.1059095	-1.6123512
C	-4.2131781	-1.3853365	-1.8012522
H	-4.3991311	-1.7357705	-2.8148292
C	-4.5000911	-2.2273775	-0.7252082
C	-4.2752371	-1.7515195	0.5647158
H	-4.5097221	-2.3874075	1.4163608
C	-3.7512761	-0.4777645	0.7951828
C	-2.8345921	1.6744235	-0.0661652
C	-3.6281311	2.7935465	0.1090858
H	-4.7051931	2.6865225	0.0389238
C	-3.0406571	4.0335885	0.4095608
H	-3.6549481	4.9153315	0.5676738
C	-1.6593581	4.1200235	0.5090618
C	-0.9556481	5.3383875	0.8243418
H	-1.5396611	6.2364205	1.0073838
C	0.3950509	5.3711425	0.8928358
H	0.9141789	6.2960145	1.1300858
C	1.1860959	4.1959865	0.6290818
C	2.5729199	4.1976705	0.6142208
H	3.1197879	5.1111685	0.8310698
C	3.2493049	3.0187755	0.2885908
H	4.3318779	2.9920635	0.2293798
C	2.5356589	1.8617825	-0.0020002
C	0.5118479	2.9789525	0.3293378
C	-0.8928201	2.9423325	0.2901218
C	3.2799159	0.6365585	-0.4051052
C	3.9945349	-0.0913015	0.5562798
C	4.6671809	-1.2485985	0.1603788
H	5.2109659	-1.8239305	0.9072538
C	4.6517969	-1.6903895	-1.1608902
C	3.9611199	-0.9283625	-2.1034962
H	3.9571459	-1.2467195	-3.1444942
C	3.2745989	0.2318855	-1.7500122
C	4.0152879	0.3399335	2.0032518

H	4.4939509	-0.4197295	2.6285638
H	4.5620449	1.2794025	2.1361538
H	3.0000829	0.4979895	2.3820568
C	2.5329349	1.0233885	-2.7988472
H	2.7589529	2.0923115	-2.7237242
H	2.7964289	0.6837185	-3.8046932
H	1.4483299	0.9185475	-2.6745432
C	-3.4081251	0.7901655	-2.7904202
H	-3.5535211	0.2582855	-3.7351972
H	-4.0656261	1.6669445	-2.7895022
H	-2.3765721	1.1571125	-2.7570892
C	-3.5139991	0.0126015	2.2021928
H	-3.8169201	-0.7395255	2.9363828
H	-2.4549471	0.2381645	2.3674648
H	-4.0758641	0.9317675	2.4000588
C	-5.0313961	-3.6191655	-0.9605342
H	-4.2311041	-4.2951265	-1.2849842
H	-5.4697341	-4.0391695	-0.0502692
H	-5.7993871	-3.6248115	-1.7410562
C	5.3438059	-2.9680985	-1.5636662
H	5.8370069	-2.8644715	-2.5356912
H	6.0979989	-3.2643805	-0.8285662
H	4.6220879	-3.7894825	-1.6477372
C	0.4934869	-1.8933875	1.4274588
C	1.5912829	-2.0836425	2.2793318
C	1.4186099	-2.4045005	3.6204888
C	0.1386649	-2.5550655	4.1482458
C	-0.9618131	-2.3916965	3.3099768
C	-0.7885531	-2.0676565	1.9695288
H	2.5915939	-1.9894655	1.8682338
H	2.2898909	-2.5428275	4.2550758
H	-0.0002451	-2.8017155	5.1965808
H	-1.9676371	-2.5161405	3.7025288
H	-1.6637081	-1.9475085	1.3386528
C	0.7324089	-1.5382185	0.0126338
C	-0.2112141	-1.7076975	-1.0270512
H	1.7805579	-1.5278845	-0.2783632
H	-1.1857451	-2.1256275	-0.7685192
C	0.2513069	-2.0798525	-2.4210822
H	0.4295109	-3.1611235	-2.4683892
H	-0.4954331	-1.8199565	-3.1770982
H	1.1896849	-1.5771475	-2.6720832
H	-1.0033431	-0.2198645	-1.5554842

Supplementary Table 10 | Int-2

Fe	0.2713268	0.3004961	-0.1121259
N	-0.8866872	2.0205801	0.0076981
N	1.7533098	1.6819761	0.0964011
C	-3.0672132	0.9656421	-0.2933279
C	-3.1288762	0.3915061	-1.5744799
C	-3.9951312	-0.6786859	-1.7915909
H	-4.0446912	-1.1240249	-2.7832519
C	-4.8070522	-1.1839759	-0.7763239
C	-4.7229702	-0.6009759	0.4855331
H	-5.3384132	-0.9912269	1.2935741
C	-3.8527472	0.4552651	0.7520561
C	-2.2251522	2.1771401	-0.0677229
C	-2.8295942	3.4244051	0.0243891
H	-3.9091822	3.4896291	-0.0540739
C	-2.0489442	4.5741861	0.2056331
H	-2.5163122	5.5521911	0.2800991
C	-0.6696562	4.4541671	0.2774451
C	0.2306908	5.5689421	0.4409091
H	-0.1979312	6.5635481	0.5317011
C	1.5728518	5.3937161	0.4771011
H	2.2365148	6.2461291	0.5958401
C	2.1636668	4.0822711	0.3555141
C	3.5290928	3.8363011	0.3672971
H	4.2307748	4.6585791	0.4741941
C	3.9956478	2.5127881	0.2325951
H	5.0577508	2.2928781	0.2315891
C	3.0936858	1.4761031	0.1019711
C	1.2841008	2.9744821	0.2097641
C	-0.1059222	3.1523541	0.1679621
C	3.5118048	0.0513321	-0.0383839
C	3.7360338	-0.7217639	1.1119891
C	4.0534678	-2.0700319	0.9632111
H	4.2150238	-2.6758879	1.8528201
C	4.1532198	-2.6655249	-0.2950479
C	3.9283528	-1.8759219	-1.4203039
H	3.9956888	-2.3261359	-2.4084769
C	3.6019228	-0.5237669	-1.3135909
C	3.6027348	-0.1107819	2.4856661
H	4.3440198	0.6799111	2.6444231
H	2.6142458	0.3430051	2.6196751
H	3.7370598	-0.8669989	3.2647241

C	3.3331678	0.2980371	-2.5499579
H	4.0578048	1.1140961	-2.6485249
H	3.3837418	-0.3192399	-3.4511029
H	2.3394718	0.7581521	-2.5099859
C	-2.2863892	0.9228091	-2.7084719
H	-2.5610152	0.4489681	-3.6547689
H	-2.4015172	2.0068401	-2.8167479
H	-1.2206352	0.7308721	-2.5369719
C	-3.7247182	0.9915491	2.1559241
H	-4.4228042	0.4871411	2.8297791
H	-2.7118152	0.8163221	2.5363511
H	-3.9136142	2.0682631	2.2073491
C	-5.7187932	-2.3569589	-1.0334609
H	-6.1945092	-2.2841589	-2.0168719
H	-5.1571562	-3.2986319	-1.0092419
H	-6.5072112	-2.4227859	-0.2776049
C	4.4966558	-4.1279559	-0.4231549
H	4.3286108	-4.4896909	-1.4418589
H	5.5490888	-4.3085239	-0.1741339
H	3.8893858	-4.7361239	0.2554921
C	-0.4139622	-1.9876969	0.9880701
C	0.3713088	-1.9787479	2.1605701
C	-0.1968252	-2.1193549	3.4196691
C	-1.5746952	-2.2845709	3.5571041
C	-2.3656652	-2.3124929	2.4115901
C	-1.7989882	-2.1656789	1.1495621
H	1.4486948	-1.8721619	2.0603031
H	0.4412248	-2.1066379	4.2998271
H	-2.0225972	-2.3969349	4.5400231
H	-3.4413582	-2.4445009	2.4969391
H	-2.4435952	-2.1769579	0.2763361
C	0.2141788	-1.7194379	-0.3368089
C	-0.4764482	-2.2998909	-1.5695409
H	1.2637128	-2.0477029	-0.3137309
H	-0.6370112	-3.3831929	-1.4451749
C	0.3343068	-2.0601359	-2.8428569
H	1.3351238	-2.4993319	-2.7544719
H	-0.1530012	-2.4970339	-3.7216049
H	0.4663148	-0.9865419	-3.0287709
H	-1.4749782	-1.8637959	-1.7021469

Supplementary Table 11 | TS-2

Fe	0.5935780	-0.1112680	0.2250506
N	2.1252380	0.5662010	-0.9353324
N	1.2355350	-1.9162670	-0.7225274
C	1.4485470	2.8717990	-0.9549354
C	0.3575310	2.9022270	-1.8496084
C	-0.7049060	3.7580970	-1.5903094
H	-1.5597230	3.7576760	-2.2629094
C	-0.7196140	4.5862950	-0.4658784
C	0.3962160	4.5913500	0.3629596
H	0.4152450	5.2538050	1.2264026
C	1.4929150	3.7559260	0.1309376
C	2.5059380	1.8481880	-1.1795454
C	3.7760840	2.1554960	-1.6410734
H	4.0299490	3.1961980	-1.8134224
C	4.7071310	1.1404680	-1.8877084
H	5.7072570	1.3769660	-2.2386944
C	4.3160930	-0.1892170	-1.7212014
C	5.1620860	-1.3214300	-2.0077784
H	6.1801900	-1.1303290	-2.3360734
C	4.7056090	-2.5932460	-1.8954514
H	5.3519470	-3.4336640	-2.1340894
C	3.3535230	-2.8627560	-1.4838414
C	2.7886080	-4.1361510	-1.4181594
H	3.3847130	-5.0087570	-1.6722934
C	1.4493470	-4.2733500	-1.0681814
H	0.9706820	-5.2464680	-1.0609184
C	0.6942850	-3.1469640	-0.7320864
C	2.5245980	-1.7670250	-1.1425704
C	3.0021220	-0.4373240	-1.2630094
C	-0.7606940	-3.2328010	-0.4203354
C	-1.2291670	-3.8406310	0.7506066
C	-2.5925670	-3.7565470	1.0509616
H	-2.9539090	-4.2016640	1.9762806
C	-3.4945240	-3.1235430	0.2032206
C	-3.0144460	-2.5902050	-0.9953814
H	-3.7072030	-2.0962110	-1.6731154
C	-1.6659040	-2.6350790	-1.3221734
C	-0.3292880	-4.6170180	1.6835956
H	-0.4105460	-5.6928970	1.4847566
H	0.7225610	-4.3467130	1.5709426
H	-0.6156110	-4.4617280	2.7292486

C	-1.1893030	-2.0449520	-2.6255824
H	-0.5633190	-2.7533670	-3.1803264
H	-2.0374650	-1.7604260	-3.2529924
H	-0.5838430	-1.1496980	-2.4509654
C	0.3596240	2.0542060	-3.0991174
H	-0.5513660	2.2193060	-3.6809244
H	1.2198490	2.2983820	-3.7334094
H	0.4308400	0.9866250	-2.8670074
C	2.6969410	3.8729460	1.0358966
H	2.3942890	4.0077720	2.0801846
H	3.3551540	3.0031690	0.9737726
H	3.2952980	4.7497840	0.7588606
C	-1.9360640	5.4159030	-0.1489074
H	-2.7287130	4.7770910	0.2585866
H	-1.7146490	6.1909760	0.5908966
H	-2.3357160	5.9001250	-1.0460324
C	-4.9591450	-3.0245720	0.5416586
H	-5.5546150	-3.6978550	-0.0866714
H	-5.1482800	-3.2853580	1.5874356
H	-5.3227900	-2.0066910	0.3673336
C	0.9613400	-0.1766400	2.4517116
C	1.1591450	-1.5787900	2.4911446
C	2.4147570	-2.1448380	2.3142346
C	3.5293590	-1.3419690	2.0680616
C	3.3674280	0.0379250	2.0222186
C	2.1109450	0.6117870	2.2054806
H	0.2991260	-2.2129840	2.6839886
H	2.5280670	-3.2241670	2.3619086
H	4.5061740	-1.7885550	1.9137546
H	4.2209070	0.6808840	1.8247776
H	2.0117550	1.6903010	2.1953426
C	-0.4013060	0.4119750	2.6236966
C	-0.4172190	1.7532870	3.3598836
H	-1.0029340	-0.3203100	3.1774126
H	0.1032840	2.5159750	2.7626156
C	0.1668770	1.7082610	4.7742326
H	1.2195130	1.4029100	4.7643336
H	0.1051580	2.6879570	5.2624136
H	-0.3803430	0.9886770	5.3941236
H	-1.4572860	2.1003360	3.4163626
C	-4.4899530	0.6585160	-0.0995594
C	-3.1293270	0.7545650	-0.4039634
C	-2.7846140	0.8194580	-1.7585614
C	-3.7410210	0.7844230	-2.7682664

C	-5.0921720	0.6712530	-2.4379864
C	-5.4651610	0.6105970	-1.0979884
H	-4.7972560	0.6081270	0.9451816
H	-1.7326530	0.8785990	-2.0293994
H	-3.4381840	0.8372150	-3.8118804
H	-5.8465790	0.6344740	-3.2191804
H	-6.5159170	0.5240140	-0.8312814
Si	-1.7744330	0.7486310	0.9856196
H	-1.0802250	2.0341630	0.6320036
H	-2.7942170	0.7750640	2.1032536
H	-1.1291910	-0.4859230	0.1952646

Supplementary Table 12 | Int-1′

Fe	0.0792836	0.2009919	-0.4059829
N	-0.7513884	2.1759469	-0.1833169
N	1.8846236	1.5266309	-0.2766649
C	-3.0453804	1.3778059	0.0096091
C	-3.5688904	0.7403959	-1.1233799
C	-4.5480094	-0.2380471	-0.9407069
H	-4.9493364	-0.7452121	-1.8149529
C	-5.0057894	-0.5934081	0.3246401
C	-4.4693504	0.0628479	1.4343751
H	-4.8147494	-0.2052261	2.4308381
C	-3.4984334	1.0506849	1.2978641
C	-2.0477764	2.4752679	-0.1476929
C	-2.5037184	3.8063829	-0.2446119
H	-3.5716334	3.9928439	-0.2075929
C	-1.6013544	4.8314939	-0.3861719
H	-1.9344304	5.8626219	-0.4674439
C	-0.2256954	4.5356839	-0.4307069
C	0.7796746	5.5488039	-0.5840189
H	0.4615196	6.5843059	-0.6651829
C	2.0938816	5.2233049	-0.6322279
H	2.8528046	5.9913419	-0.7511719
C	2.5132286	3.8546659	-0.5310079
C	3.8654046	3.4642359	-0.5837259
H	4.6379126	4.2183999	-0.7082879
C	4.1892806	2.1339379	-0.4763299
H	5.2200896	1.7981119	-0.5110149
C	3.1668086	1.1754779	-0.3179209
C	1.5532006	2.8358159	-0.3756459

C	0.1497876	3.1828119	-0.3229629
C	3.4976006	-0.2690611	-0.1570039
C	3.9304956	-0.7286621	1.0958941
C	4.1375226	-2.0960731	1.2725161
H	4.4457206	-2.4617991	2.2497271
C	3.9527286	-3.0026401	0.2302931
C	3.5947406	-2.5064771	-1.0227159
H	3.4776936	-3.1978381	-1.8546879
C	3.3636936	-1.1498511	-1.2417779
C	4.2006626	0.2267159	2.2354271
H	4.2189346	-0.3035561	3.1918091
H	5.1738816	0.7177389	2.1101651
H	3.4459356	1.0157769	2.3042891
C	2.9741946	-0.6579881	-2.6110749
H	3.5064346	0.2634739	-2.8736889
H	3.2030186	-1.4110381	-3.3705089
H	1.8968136	-0.4472301	-2.6486889
C	-3.0783934	1.0864509	-2.5053059
H	-3.6616174	0.5633849	-3.2680749
H	-3.1521134	2.1631339	-2.7000939
H	-2.0264324	0.7918369	-2.6131519
C	-2.9352314	1.7477219	2.5115941
H	-3.3386124	1.3170779	3.4321421
H	-1.8443594	1.6575129	2.5451931
H	-3.1729994	2.8184329	2.5077581
C	-6.0244674	-1.6895231	0.5080321
H	-5.5471414	-2.5971851	0.8966601
H	-6.8012834	-1.3970851	1.2226001
H	-6.5102504	-1.9465671	-0.4379919
C	4.1098156	-4.4854731	0.4503681
H	4.7135796	-4.6993781	1.3376221
H	3.1298156	-4.9566581	0.5947951
H	4.5824196	-4.9690061	-0.4107239
C	-0.6568304	-2.6368151	-0.0534409
C	-0.0398314	-3.7394251	-0.6620319
C	-0.7849864	-4.7206051	-1.3059049
C	-2.1735094	-4.6246081	-1.3647059
C	-2.8007294	-3.5343451	-0.7644599
C	-2.0558664	-2.5597511	-0.1131859
H	1.0452686	-3.8133841	-0.6331449
H	-0.2785724	-5.5642571	-1.7682339
H	-2.7575204	-5.3861641	-1.8736879
H	-3.8828094	-3.4346301	-0.8107579
H	-2.5675174	-1.7152831	0.3361931

C	0.1748616	-1.6084471	0.6005011
C	-0.3040424	-0.5904411	1.4671571
H	1.2269796	-1.8823861	0.6964191
H	-1.3768114	-0.5619541	1.6628391
C	0.5545966	-0.0568341	2.5913901
H	0.4396236	1.0263939	2.7320461
H	0.3126566	-0.5390071	3.5492711
H	1.6101866	-0.2547161	2.3873541
H	-0.0595124	-0.0684291	-2.0636139

Supplementary Table 13 | TS-1′

Fe	0.3840762	0.3181276	0.0122175
N	0.0744352	2.3197296	-0.1080515
N	2.2999012	0.9214436	-0.2290615
C	-2.3246088	2.1988796	0.1264015
C	-2.8255328	1.4641676	-0.9582915
C	-3.9382788	0.6473716	-0.7605005
H	-4.3078088	0.0514686	-1.5917185
C	-4.5629658	0.5499966	0.4792745
C	-4.0565088	1.3040436	1.5389135
H	-4.5304798	1.2334356	2.5163375
C	-2.9409778	2.1238066	1.3852225
C	-1.0870788	3.0108376	-0.0472815
C	-1.1337168	4.3902166	-0.1732535
H	-2.0956768	4.8891526	-0.1282385
C	0.0490742	5.1190886	-0.3860565
H	0.0180902	6.1991966	-0.4999965
C	1.2576132	4.4421536	-0.4714425
C	2.5319612	5.0711646	-0.7260225
H	2.5570392	6.1516886	-0.8413005
C	3.6707162	4.3453136	-0.8348925
H	4.6190362	4.8369766	-1.0353745
C	3.6648872	2.9094386	-0.6847915
C	4.7907722	2.1030556	-0.7758075
H	5.7628152	2.5440586	-0.9784525
C	4.6607752	0.7158276	-0.5902965
H	5.5263112	0.0633466	-0.6315175
C	3.4179992	0.1662606	-0.3189675
C	2.4197502	2.2815826	-0.4143235
C	1.2381432	3.0300456	-0.3206875
C	3.2407292	-1.2881094	-0.0405415
C	3.2839172	-1.7350434	1.2873165

C	3.0316862	-3.0815394	1.5526935
H	3.0632682	-3.4318444	2.5827095
C	2.7364242	-3.9852054	0.5332725
C	2.7157692	-3.5170184	-0.7810595
H	2.4898902	-4.2105484	-1.5886225
C	2.9660262	-2.1805974	-1.0862175
C	3.6001262	-0.7771084	2.4102795
H	3.5303542	-1.2737854	3.3823165
H	4.6108102	-0.3668804	2.3059745
H	2.9092552	0.0729686	2.4110685
C	2.9079592	-1.6915834	-2.5112285
H	3.8704982	-1.2794794	-2.8333795
H	2.6355212	-2.5005044	-3.1952015
H	2.1643002	-0.8922594	-2.6093815
C	-2.1756098	1.5390716	-2.3185725
H	-2.7880968	1.0328206	-3.0694625
H	-2.0255738	2.5786236	-2.6296825
H	-1.1893468	1.0604716	-2.3162735
C	-2.3708858	2.8692276	2.5669555
H	-2.9012578	2.6080716	3.4873285
H	-1.3104418	2.6285706	2.7031425
H	-2.4375198	3.9542416	2.4329245
C	-5.7381838	-0.3702214	0.6894445
H	-5.4615618	-1.2112184	1.3367195
H	-6.5739958	0.1512026	1.1692485
H	-6.0927808	-0.7860244	-0.2580095
C	2.4149402	-5.4254724	0.8445715
H	2.8973422	-5.7512624	1.7714075
H	1.3342082	-5.5658254	0.9685615
H	2.7411642	-6.0913524	0.0393905
C	-1.5992038	-1.9850774	-0.5816155
C	-1.5123708	-1.9356654	-1.9745235
C	-2.5661068	-2.3720724	-2.7731675
C	-3.7246588	-2.8790894	-2.1913115
C	-3.8197268	-2.9447404	-0.8033305
C	-2.7694738	-2.4955434	-0.0097635
H	-0.6162158	-1.5326104	-2.4371045
H	-2.4795608	-2.3119154	-3.8544485
H	-4.5461788	-3.2210624	-2.8142995
H	-4.7177458	-3.3391804	-0.3362865
H	-2.8539688	-2.5327744	1.0728725
C	-0.4850168	-1.5530604	0.3342705
C	-0.7553478	-0.6512184	1.3990125
H	0.2540072	-2.3363174	0.5262575

H	-1.7472038	-0.2015584	1.4116265
C	-0.0885048	-0.7828404	2.7498415
H	0.0769742	0.1935826	3.2196305
H	-0.7066008	-1.3768004	3.4383825
H	0.8800292	-1.2870114	2.6646805
H	0.5581722	-0.9393054	-0.8304585

Supplementary Table 14 | Int-2′

Fe	0.1710418	0.4503612	-0.4519075
N	-0.9869622	2.0956922	0.0638725
N	1.6618958	1.7732292	0.0159855
C	-3.1409792	1.0026792	-0.1585695
C	-3.7687652	0.8437732	-1.4040525
C	-4.5566332	-0.2842258	-1.6199075
H	-5.0364452	-0.4164828	-2.5874695
C	-4.7256002	-1.2575098	-0.6359765
C	-4.0924022	-1.0768228	0.5915525
H	-4.2073862	-1.8281678	1.3691675
C	-3.2973842	0.0390532	0.8495355
C	-2.3285372	2.2245592	0.1096315
C	-2.9448542	3.4347322	0.3987665
H	-4.0278712	3.4783172	0.4299535
C	-2.1670602	4.5745452	0.6454815
H	-2.6406452	5.5276122	0.8648615
C	-0.7843012	4.4787322	0.6101835
C	0.1085188	5.5850052	0.8485435
H	-0.3256232	6.5583292	1.0612435
C	1.4523058	5.4252852	0.8088675
H	2.1125838	6.2692462	0.9904435
C	2.0520228	4.1415532	0.5319235
C	3.4199128	3.9176332	0.4896045
H	4.1136008	4.7344602	0.6667445
C	3.8970658	2.6191452	0.2201565
H	4.9612138	2.4115032	0.1888975
C	3.0067918	1.5885062	-0.0058695
C	1.1825508	3.0417772	0.2906785
C	-0.2092272	3.2079362	0.3248045
C	3.4747738	0.1970172	-0.2700945
C	3.8273638	-0.6269378	0.8085625
C	4.2535928	-1.9304728	0.5508595
H	4.5152678	-2.5757408	1.3868365
C	4.3375528	-2.4279808	-0.7474985

C	3.9913558	-1.5849858	-1.8044515
H	4.0530308	-1.9599688	-2.8241625
C	3.5581858	-0.2791618	-1.5888895
C	3.7469118	-0.1177888	2.2281735
H	3.8538228	-0.9379678	2.9443895
H	4.5345328	0.6160192	2.4327355
H	2.7907118	0.3816212	2.4187965
C	3.1847698	0.6057832	-2.7521245
H	3.7165918	1.5622602	-2.7100555
H	3.4167208	0.1210662	-3.7044275
H	2.1126418	0.8349902	-2.7454555
C	-3.5764842	1.8611572	-2.5024015
H	-4.0183012	1.5102452	-3.4393575
H	-4.0365272	2.8227002	-2.2495755
H	-2.5121202	2.0527472	-2.6787865
C	-2.6246672	0.2000742	2.1892855
H	-3.0302602	-0.5067758	2.9169245
H	-1.5489792	0.0040692	2.1176285
H	-2.7484132	1.2157862	2.5800975
C	-5.5255372	-2.5048118	-0.9133385
H	-4.8770502	-3.2942638	-1.3130705
H	-5.9926152	-2.8926008	-0.0024975
H	-6.3134282	-2.3218758	-1.6507345
C	4.7453748	-3.8550228	-1.0114715
H	5.2173718	-4.3061908	-0.1336765
H	3.8700648	-4.4635968	-1.2686145
H	5.4487078	-3.9206288	-1.8483295
C	-0.0510062	-2.6422108	0.7521095
C	-1.2588662	-3.3191108	0.9418525
C	-1.8318952	-3.4249348	2.2068635
C	-1.2059372	-2.8539218	3.3122955
C	-0.0045332	-2.1709588	3.1395185
C	0.5612748	-2.0668478	1.8719095
H	-1.7489792	-3.7773608	0.0864195
H	-2.7688682	-3.9618808	2.3304745
H	-1.6497362	-2.9434028	4.2997035
H	0.4968048	-1.7234958	3.9935475
H	1.5116108	-1.5558718	1.7415815
C	0.5892748	-2.5165858	-0.6116485
C	0.0800158	-1.3247728	-1.4513825
H	1.6711948	-2.4170268	-0.4739795
H	0.7854568	-1.2272818	-2.2942235
C	-1.3091872	-1.6066108	-2.0348165
H	-1.6386832	-0.8084538	-2.7108965

H	-1.3297452	-2.5497308	-2.6077085
H	-2.0702542	-1.6899768	-1.2511025
H	0.4282718	-3.4587878	-1.1629435

Supplementary Table 15 | Ts-2′

Fe	-0.3042549	-0.0483680	-0.0189341
N	-1.3558269	-1.2973850	-1.1376551
N	-1.6806559	1.3262230	-0.9369851
C	0.2226961	-3.0643570	-0.8203741
C	1.3866841	-2.6411820	-1.4889721
C	2.6298601	-2.9292490	-0.9284311
H	3.5282221	-2.5757760	-1.4288371
C	2.7451081	-3.6111550	0.2805929
C	1.5800081	-4.0425390	0.9133929
H	1.6559351	-4.5762570	1.8590779
C	0.3169331	-3.7811620	0.3843419
C	-1.1075889	-2.6266870	-1.3284901
C	-2.0330189	-3.4547510	-1.9258801
H	-1.7931799	-4.5051320	-2.0533391
C	-3.2716799	-2.9438370	-2.3623061
H	-4.0086879	-3.5964070	-2.8201101
C	-3.5305979	-1.5807990	-2.2193851
C	-4.7423029	-0.9315440	-2.6575031
H	-5.5211719	-1.5478000	-3.0986991
C	-4.9165499	0.4091910	-2.5417261
H	-5.8335549	0.8773240	-2.8896331
C	-3.8917959	1.2450700	-1.9764891
C	-3.9745899	2.6305570	-1.8608451
H	-4.8653069	3.1502590	-2.2043191
C	-2.9016759	3.3456310	-1.3304761
H	-2.9269289	4.4276840	-1.2622761
C	-1.7678259	2.6638500	-0.8818561
C	-2.7063439	0.6199620	-1.5096151
C	-2.5351539	-0.7738100	-1.6218881
C	-0.5575699	3.3605450	-0.3555031
C	-0.5151029	3.9146570	0.9292959
C	0.7102021	4.3828250	1.4164829
H	0.7503841	4.7897880	2.4251289
C	1.8730841	4.3266850	0.6590829
C	1.7865121	3.8451560	-0.6500461
H	2.6822791	3.8195300	-1.2668151
C	0.5929361	3.3656750	-1.1709131

C	-1.7292069	4.0108230	1.8251619
H	-1.6884259	3.2734100	2.6351069
H	-1.7774999	4.9997260	2.2928689
H	-2.6636959	3.8533240	1.2820889
C	0.5342641	2.8510900	-2.5879371
H	-0.2852879	3.3167090	-3.1466411
H	1.4697501	3.0577970	-3.1154301
H	0.3602281	1.7692640	-2.6104181
C	1.3095731	-1.8796710	-2.7900831
H	2.3043761	-1.7517980	-3.2241791
H	0.6706681	-2.3965680	-3.5144101
H	0.8812751	-0.8814950	-2.6421471
C	-0.9205999	-4.2368110	1.1199119
H	-1.3589639	-5.1261340	0.6520009
H	-0.6831509	-4.4892480	2.1582529
H	-1.6964689	-3.4645500	1.1246099
C	4.0919381	-3.8210500	0.9210029
H	4.1802241	-4.8198920	1.3615429
H	4.9020141	-3.6869370	0.1982689
H	4.2416411	-3.0884220	1.7237439
C	3.2033501	4.7483660	1.2261229
H	3.0863181	5.2736970	2.1786519
H	3.8327741	3.8679220	1.4017249
H	3.7439051	5.4048660	0.5360319
Si	2.0504411	0.1599890	1.0108349
H	2.1390031	1.3407040	1.9179169
H	2.6154041	-1.0461850	1.7036739
C	3.5157141	0.2545910	-0.2381101
C	3.4003741	0.7469220	-1.5409381
C	4.7589691	-0.2715500	0.1382799
C	4.4698041	0.7216390	-2.4347511
H	2.4437361	1.1404540	-1.8754481
C	5.8405851	-0.2969800	-0.7383541
H	4.8773671	-0.6940480	1.1354649
C	5.6960891	0.1981410	-2.0335521
H	4.3442011	1.1032840	-3.4451311
H	6.7924921	-0.7130550	-0.4180641
H	6.5319001	0.1695380	-2.7274211
C	-2.4200059	-0.1274790	2.2624719
C	-2.7024699	-1.4059140	2.7566029
C	-3.9649869	-1.9697920	2.6077239
C	-4.9759219	-1.2665470	1.9563259
C	-4.7154839	0.0118820	1.4760369
C	-3.4524679	0.5759150	1.6375489

H	-1.9213569	-1.9782860	3.2488179
H	-4.1553599	-2.9672570	2.9936609
H	-5.9555779	-1.7150120	1.8214549
H	-5.4878199	0.5703580	0.9551729
H	-3.2607099	1.5687500	1.2421549
C	-1.0206199	0.4609130	2.3614379
C	0.1363071	-0.5468660	2.0632859
H	-0.8501719	0.8738050	3.3646429
H	-0.3068379	-1.4706690	1.6476369
C	0.7951061	-1.0042840	3.3672289
H	0.0322341	-1.2442690	4.1206989
H	1.4047141	-1.8978490	3.2128509
H	1.4415451	-0.2256200	3.7916319
H	1.1526971	0.8340280	-0.1811041
H	-0.9773759	1.3539130	1.7143819

Supplementary Table 16 | Int-1'' Low Spin

Fe	0.1358752	-0.5182315	-0.1043017
N	-1.1611528	0.8068705	-0.6953537
N	1.4677162	0.8358205	-0.8816777
C	-3.1750828	-0.5768395	-0.5000287
C	-3.1269908	-1.4668075	-1.5931257
C	-3.7491128	-2.7053205	-1.4780167
H	-3.6994318	-3.3966025	-2.3166637
C	-4.4369998	-3.0824475	-0.3252547
C	-4.5278688	-2.1614655	0.7119873
H	-5.0904178	-2.4190335	1.6081393
C	-3.9258208	-0.9039505	0.6381323
C	-2.5023968	0.7460825	-0.6631297
C	-3.2851088	1.8900555	-0.9082157
H	-4.3636188	1.7885665	-0.8517237
C	-2.7026658	3.0933895	-1.2363587
H	-3.3071538	3.9759395	-1.4241617
C	-1.3066608	3.1524405	-1.3698847
C	-0.5912898	4.3288425	-1.7843667
H	-1.1615438	5.2309625	-1.9868207
C	0.7569462	4.3218945	-1.9316767
H	1.2822782	5.2165555	-2.2536627
C	1.5206122	3.1376235	-1.6525637
C	2.9177532	3.0507045	-1.7530907
H	3.4912802	3.9092995	-2.0912197
C	3.5509812	1.8694585	-1.4129927

H	4.6289862	1.7723195	-1.4860547
C	2.8001512	0.7698825	-0.9709637
C	0.8400442	1.9864485	-1.2158467
C	-0.5850928	1.9826555	-1.0871377
C	3.4146022	-0.5254645	-0.5632557
C	4.1220012	-0.6190205	0.6496723
C	4.5807042	-1.8668395	1.0632763
H	5.1080612	-1.9473065	2.0116203
C	4.3770762	-3.0156305	0.2977363
C	3.7160052	-2.8879895	-0.9199797
H	3.5641392	-3.7695235	-1.5389647
C	3.2302162	-1.6599405	-1.3713777
C	4.4001702	0.5956175	1.5024163
H	4.8513862	0.3047645	2.4550253
H	5.0910312	1.2856525	1.0046743
H	3.4888302	1.1581145	1.7235813
C	2.5240962	-1.5794205	-2.7013957
H	2.7628172	-0.6520425	-3.2321327
H	2.8096982	-2.4210365	-3.3393917
H	1.4385862	-1.6133085	-2.5503407
C	-2.4403878	-1.0939245	-2.8810327
H	-2.6749998	-1.8182025	-3.6663437
H	-2.7493578	-0.1018305	-3.2307887
H	-1.3557118	-1.0820465	-2.7289307
C	-4.1388298	0.0449795	1.7939443
H	-3.6291888	-0.3136025	2.6962563
H	-3.7769198	1.0524795	1.5839383
H	-5.2056948	0.1138475	2.0336063
C	-5.1118998	-4.4273085	-0.2241017
H	-4.9733628	-4.8676145	0.7688743
H	-6.1916238	-4.3385175	-0.3955707
H	-4.7154888	-5.1294295	-0.9638527
C	4.8941772	-4.3513275	0.7689133
H	5.9718852	-4.4419645	0.5868403
H	4.7316302	-4.4822715	1.8436813
H	4.3992712	-5.1767955	0.2486393
C	0.0614172	1.6810425	1.9856253
C	0.9232652	2.7549855	1.6977023
C	0.4878512	4.0751695	1.7455853
C	-0.8308878	4.3711625	2.0779693
C	-1.6932618	3.3238675	2.4013003
C	-1.2501518	2.0089475	2.3682793
H	1.9493472	2.5479315	1.4042623
H	1.1822302	4.8747195	1.5013923

H	-1.1769568	5.4002855	2.1039193
H	-2.7194488	3.5338255	2.6931343
H	-1.9222778	1.2124555	2.6598633
C	0.5102512	0.2964715	1.8170943
C	-0.3121788	-0.8661595	1.7968123
H	1.5745522	0.1356255	1.9963303
H	-1.3800098	-0.7654045	1.9899383
C	0.2820202	-2.2015365	2.2035663
H	0.3299132	-2.3076175	3.2970023
H	-0.3059328	-3.0322125	1.8025663
H	1.3099722	-2.3123725	1.8279603
H	-0.2268448	-1.7393955	-1.0795087

Supplementary Table 17 | Int-1''' Low spin

Fe	0.0636712	-0.0265595	-0.0516910
N	-1.4471428	1.4746615	-0.3405200
N	1.2425532	1.5869175	-0.2172190
C	-3.4719868	0.1075675	-0.2169580
C	-3.5764508	-0.7775195	-1.2990010
C	-4.2876288	-1.9623905	-1.1211690
H	-4.3761328	-2.6523515	-1.9583960
C	-4.8634308	-2.3008415	0.1026420
C	-4.7538698	-1.3964845	1.1566570
H	-5.2071558	-1.6374895	2.1160410
C	-4.0836798	-0.1817745	1.0117100
C	-2.7780638	1.4135695	-0.3879060
C	-3.5771138	2.5604235	-0.5725920
H	-4.6539018	2.4366285	-0.6171850
C	-2.9942388	3.7970435	-0.6878520
H	-3.5932198	4.6922885	-0.8298550
C	-1.5947438	3.8944195	-0.6126340
C	-0.9066718	5.1496815	-0.7023790
H	-1.4946888	6.0540205	-0.8308840
C	0.4439502	5.2062645	-0.6296790
H	0.9660562	6.1562285	-0.6996350
C	1.2195732	4.0105815	-0.4662320
C	2.6234072	4.0247465	-0.4062250
H	3.1600182	4.9667805	-0.4747160
C	3.2915242	2.8318945	-0.2821170
H	4.3754172	2.7937375	-0.2566330
C	2.5809522	1.6190325	-0.2001300
C	0.5723922	2.7628845	-0.3686080

C	-0.8642088	2.7001295	-0.4424730
C	3.3707502	0.3532185	-0.1400530
C	4.0278902	-0.0064715	1.0472850
C	4.8152872	-1.1571375	1.0617880
H	5.3087112	-1.4467265	1.9874770
C	4.9890772	-1.9382615	-0.0797240
C	4.3680342	-1.5306465	-1.2577860
H	4.5089172	-2.1154535	-2.1642020
C	3.5628302	-0.3943285	-1.3145750
C	3.9320402	0.8443395	2.2909890
H	2.9286612	1.2545045	2.4332340
H	4.1889732	0.2641785	3.1821870
H	4.6232502	1.6949745	2.2399610
C	2.9383232	0.0250715	-2.6182860
H	3.1679982	1.0718735	-2.8507810
H	3.3033172	-0.5949575	-3.4418530
H	1.8476052	-0.0730555	-2.5570290
C	-2.9003898	-0.4751915	-2.6095080
H	-3.1722018	-1.2129615	-3.3698630
H	-3.1731158	0.5182905	-2.9846070
H	-1.8108638	-0.4927025	-2.4762030
C	-4.0453078	0.7951275	2.1633220
H	-4.2524328	0.2874835	3.1098220
H	-3.0738458	1.2892715	2.2520770
H	-4.7992728	1.5825045	2.0379940
C	-5.5536518	-3.6281065	0.2878580
H	-4.8312608	-4.3987455	0.5825220
H	-6.3191448	-3.5773965	1.0684180
H	-6.0317248	-3.9627765	-0.6382500
C	5.8008882	-3.2078795	-0.0348080
H	6.5751782	-3.1627325	0.7374430
H	5.1591522	-4.0682425	0.1921280
H	6.2868832	-3.4046885	-0.9956000
C	0.3961292	-1.9081265	0.7937720
C	1.4964822	-2.6719445	0.2838540
C	1.3331392	-3.5273875	-0.7689640
C	0.0639902	-3.6898945	-1.3765880
C	-1.0132658	-3.0001975	-0.8822070
C	-0.8901168	-2.0883555	0.1997130
H	2.4705272	-2.5416335	0.7458460
H	2.1882372	-4.0843485	-1.1443290
H	-0.0549598	-4.3758715	-2.2102160
H	-1.9994008	-3.1645005	-1.3049900
H	-1.7977188	-1.7893365	0.7115900

C	0.6200552	-0.8266705	1.7158420
C	-0.4166208	0.0827795	2.0031800
H	1.6350682	-0.6593375	2.0675940
H	-1.4347228	-0.2995475	2.0731780
C	-0.1724048	1.3338535	2.8062130
H	-0.8261588	2.1517895	2.4735700
H	-0.3602058	1.1861875	3.8787490
H	0.8581982	1.6881885	2.6936720
H	0.1480072	-0.2521135	-1.6152140

Supplementary Table 18 | Pro

C	1.2055716	-0.2886607	0.1339525
C	0.7652686	-1.1499617	1.1434785
C	0.8514916	-2.5314397	0.9998705
C	1.3863676	-3.0827477	-0.1609025
C	1.8354216	-2.2380877	-1.1733355
C	1.7439016	-0.8571497	-1.0273815
H	0.3337826	-0.7293587	2.0482605
H	0.4975086	-3.1783637	1.7973965
H	1.4564656	-4.1602147	-0.2759945
H	2.2573286	-2.6554647	-2.0831995
H	2.0878476	-0.2132417	-1.8327385
C	1.0260926	1.2086123	0.2669285
C	2.2838826	2.0334863	-0.0728125
H	0.7518566	1.4248883	1.3094475
H	2.5460556	1.9011533	-1.1304175
C	3.4882536	1.6751733	0.7985555
H	3.7632596	0.6229913	0.6725005
H	4.3581256	2.2882233	0.5398145
H	3.2637216	1.8371663	1.8591785
H	2.0499686	3.0989033	0.0480765
C	-3.0971464	1.3450833	0.2053955
C	-1.9935964	0.7365283	-0.4083075
C	-2.0676884	-0.6381227	-0.6865185
C	-3.2016654	-1.3742177	-0.3588875
C	-4.2871824	-0.7502427	0.2524895
C	-4.2348924	0.6116363	0.5341495
H	-3.0716344	2.4095333	0.4283635
H	-1.2281114	-1.1444397	-1.1570895
H	-3.2382974	-2.4372157	-0.5793555
H	-5.1732284	-1.3249947	0.5073905
H	-5.0798164	1.1031893	1.0079995

Si	-0.4567504	1.7318363	-0.8046825
H	-0.0641344	1.5520903	-2.2303415
H	-0.7580264	3.1734293	-0.5712845

Supplementary Table 19 | Pro′

Si	2.1170354	-0.5680778	1.0511650
H	2.4917494	0.4171652	2.1058920
H	2.7981934	-1.8629278	1.3312480
C	2.6762354	0.0810692	-0.6175280
C	2.9786434	-0.7966478	-1.6684560
C	2.7384924	1.4587312	-0.8730730
C	3.3254424	-0.3188598	-2.9292360
H	2.9487624	-1.8715098	-1.5015880
C	3.0840644	1.9429022	-2.1320130
H	2.5166094	2.1660742	-0.0767220
C	3.3769794	1.0532472	-3.1622510
H	3.5573784	-1.0156698	-3.7295980
H	3.1268934	3.0138652	-2.3082500
H	3.6478354	1.4284952	-4.1450250
C	-1.9882906	0.3093122	0.5854180
C	-2.8839406	0.6335012	1.6061790
C	-4.2526916	0.4259442	1.4503960
C	-4.7470846	-0.1107988	0.2654990
C	-3.8637516	-0.4376498	-0.7614590
C	-2.4981556	-0.2289678	-0.5998430
H	-2.5040736	1.0555492	2.5337710
H	-4.9326486	0.6855902	2.2567970
H	-5.8140336	-0.2696208	0.1400420
H	-4.2401926	-0.8535068	-1.6917480
H	-1.8133466	-0.4812738	-1.4068470
C	-0.4977706	0.4927332	0.7550100
C	0.2412864	-0.8243118	1.0877340
H	-0.3070006	1.2227062	1.5534460
H	0.0091074	-1.5471028	0.2908100
C	-0.2128326	-1.4179668	2.4282300
H	-1.2908896	-1.6150418	2.4286850
H	0.3002594	-2.3611378	2.6461950
H	-0.0044406	-0.7259178	3.2543870
H	-0.0838256	0.9201032	-0.1672680

Supplementary Table 20 | PhSiH$_3$

Si	-0.8207295	2.3351441	-0.0082508
H	-0.3264995	3.0526961	1.1959052
H	-0.3459075	3.0742481	-1.2087468
H	-2.3069205	2.3516051	-0.0182978
C	-0.2049725	0.5648121	0.0085772
C	1.0200425	0.2327881	0.6042122
C	-0.9481375	-0.4631849	-0.5884368
C	1.4913705	-1.0771309	0.5963672
H	1.6158765	1.0044351	1.0867062
C	-0.4812445	-1.7747449	-0.5990518
H	-1.9084145	-0.2418089	-1.0489718
C	0.7405845	-2.0827709	-0.0067508
H	2.4425785	-1.3134779	1.0643972
H	-1.0730025	-2.5566799	-1.0660698
H	1.1053755	-3.1059299	-0.0115888

Supplementary Table 21 | PhCH=CHMe

C	-1.6067581	1.3125294	0.4372286
C	-0.4114971	1.1262284	-0.1296514
H	-2.0961621	0.4862794	0.9521396
H	0.0413769	1.9633564	-0.6626654
C	0.3774899	-0.1189746	-0.1422974
C	1.4566599	-0.2289786	-1.0280874
C	0.1020079	-1.2072026	0.6976276
C	2.2231169	-1.3880896	-1.0904494
H	1.6919249	0.6075584	-1.6819924
C	0.8647529	-2.3666776	0.6360796
H	-0.7091681	-1.1428106	1.4170206
C	1.9281579	-2.4647846	-0.2597044
H	3.0532199	-1.4494406	-1.7883384
H	0.6336879	-3.1972146	1.2971206
H	2.5242839	-3.3713186	-0.3045044
C	-2.3634221	2.6059924	0.4138076
H	-3.3435831	2.4800884	-0.0626504
H	-2.5496271	2.9720624	1.4313046
H	-1.8164611	3.3813974	-0.1319884

Supplementary Table 22 | TS-3′

Fe	0.1190341	1.0294964	0.1576479
N	-1.2571159	2.5279554	-0.0586371
N	1.3993941	2.5436184	-0.1332571
C	-3.2781819	1.1710884	-0.0331111
C	-3.2475169	0.3308374	-1.1572371
C	-3.8620569	-0.9207506	-1.0781581
H	-3.8378759	-1.5671566	-1.9507481
C	-4.5105589	-1.3508926	0.0750729
C	-4.5334939	-0.4908966	1.1733330
H	-5.0344649	-0.8216396	2.0809379
C	-3.9240069	0.7611594	1.1477849
C	-2.6107179	2.5028304	-0.1014141
C	-3.3715319	3.6497344	-0.2669471
H	-4.4499259	3.5562714	-0.3263271
C	-2.7475929	4.9008824	-0.3914331
H	-3.3369109	5.8043904	-0.5200750
C	-1.3628709	4.9668844	-0.3823281
C	-0.6156069	6.1886294	-0.5604170
H	-1.1704689	7.1170644	-0.6672191
C	0.7370691	6.1900534	-0.6109351
H	1.2820341	7.1193874	-0.7540581
C	1.4912451	4.9681144	-0.4782041
C	2.8760541	4.8984514	-0.5177840
H	3.4619791	5.8020494	-0.6612080
C	3.5085851	3.6542964	-0.3661591
H	4.5894751	3.5685394	-0.3869101
C	2.7494801	2.5080794	-0.1817671
C	0.7668871	3.7596494	-0.2832941
C	-0.6353169	3.7544474	-0.2258141
C	3.3872201	1.1706564	0.0011329
C	3.5160501	0.6371784	1.2979370
C	4.0506371	-0.6399056	1.4430250
H	4.1662401	-1.0559206	2.4401039
C	4.4303571	-1.4100156	0.3426339
C	4.2969331	-0.8546486	-0.9250091
H	4.5871041	-1.4481486	-1.7897090
C	3.7902441	0.4319374	-1.1204701
C	3.1266651	1.4570334	2.5213119
H	2.3355821	2.1508424	2.2193929
C	3.6466711	0.9760084	-2.5350661
H	3.3624121	2.0299364	-2.4637331
C	-2.6224579	0.7652574	-2.4764621

H	-2.1360539	1.7324844	-2.3251461
C	-3.9524979	1.6412534	2.3902909
H	-3.2114089	2.4356564	2.2554659
C	-5.2384019	-2.6825446	0.1575389
H	-5.0942059	-3.0652156	1.1771129
C	5.0042871	-2.8035746	0.5284279
H	5.1240161	-3.2451186	-0.4696531
C	0.2768771	-0.9534696	0.8492120
C	-0.6762289	-0.2104506	1.5604600
H	1.2838601	-1.0355076	1.2634970
H	-1.7303679	-0.4210726	1.4053600
H	1.0235211	0.0344304	-0.5581730
H	-0.4265549	0.2504194	2.5158049
C	-0.1245209	-2.1555756	0.0140129
C	0.8814891	-2.4960726	-1.0912700
C	-0.2918639	-3.3539056	0.9699600
H	-1.1030199	-1.9526696	-0.4474091
C	0.5187141	-3.7871176	-1.8268561
H	1.8813061	-2.5909746	-0.6440540
H	0.9489691	-1.6679756	-1.8025220
C	-0.6391499	-4.6494976	0.2330199
H	0.6504031	-3.4951836	1.5211230
H	-1.0605829	-3.1184856	1.7150470
C	0.3854171	-4.9644316	-0.8583890
H	1.2715791	-4.0043136	-2.5938441
H	-0.4366619	-3.6440336	-2.3529371
H	-0.7086389	-5.4780696	0.9478020
H	-1.6306919	-4.5475556	-0.2292241
H	0.1021201	-5.8764886	-1.3971721
H	1.3619011	-5.1611246	-0.3922141
C	-6.7462639	-2.4691836	-0.0511911
H	-7.2911439	-3.4146256	0.0508289
H	-7.1502849	-1.7578396	0.6765849
H	-6.9378079	-2.0683006	-1.0534390
C	-4.7077099	-3.7411136	-0.8116030
H	-4.9387339	-3.4876076	-1.8528620
H	-3.6225909	-3.8553376	-0.7233720
H	-5.1721839	-4.7106096	-0.6032171
C	4.0671311	-3.7164636	1.3300010
H	3.0859941	-3.7972986	0.8505259
H	4.4912431	-4.7233886	1.4137780
H	3.9125631	-3.3361216	2.3462699
C	6.3933911	-2.7349616	1.1790079
H	6.8330541	-3.7347966	1.2692290

H	7.0714721	-2.1110096	0.5878360
H	6.3303231	-2.3020636	2.1843240
C	-1.5399889	-0.2092596	-2.9542091
H	-0.7496509	-0.2976196	-2.2018961
H	-1.9437839	-1.2108136	-3.1443051
H	-1.0849319	0.1507494	-3.8837961
C	-3.7094569	0.9623054	-3.5425221
H	-4.2256109	0.0204084	-3.7626301
H	-4.4582619	1.6869534	-3.2057341
H	-3.2691459	1.3319984	-4.4754691
C	2.5265491	0.2571624	-3.2983701
H	2.4251391	0.6705944	-4.3085930
H	2.7323451	-0.8162016	-3.3897421
H	1.5709681	0.3779024	-2.7793491
C	4.9719501	0.9188194	-3.3055171
H	5.7695421	1.4333044	-2.7588811
H	5.2956681	-0.1138296	-3.4792321
H	4.8616801	1.3987964	-4.2843471
C	-5.3272169	2.3051804	2.5522179
H	-5.3397979	2.9619504	3.4295909
H	-5.5845559	2.9049244	1.6735580
H	-6.1094629	1.5476844	2.6827419
C	-3.5708849	0.8805784	3.6672749
H	-4.3249309	0.1330934	3.9383179
H	-2.6114729	0.3673694	3.5549129
H	-3.4893679	1.5803274	4.5064100
C	4.3242701	2.2940504	2.9962509
H	5.1561391	1.6421194	3.2886749
H	4.6756751	2.9618784	2.2036379
H	4.0490271	2.9074304	3.8620719
C	2.5679861	0.6170444	3.6751539
H	1.7712811	-0.0551536	3.3396300
H	3.3441431	0.0081934	4.1532269
H	2.1554131	1.2761804	4.4465259

Supplementary Table 23 | TS-3

Fe	-0.0148585	0.4781384	-0.4936006
N	-1.2871595	2.0471784	-0.3949926
N	1.3688745	1.9711884	-0.5887646
C	-3.3246535	0.7293724	-0.2535666
C	-3.6207325	0.0432514	-1.4454166
C	-4.2272305	-1.2075086	-1.3692136

H	-4.4540115	-1.7409686	-2.2885996
C	-4.5336715	-1.8001396	-0.1466166
C	-4.2319045	-1.1006906	1.0179884
H	-4.4678375	-1.5644516	1.9713184
C	-3.6286905	0.1591844	0.9955674
C	-2.6408535	2.0559234	-0.3486526
C	-3.3692195	3.2327974	-0.4151766
H	-4.4520395	3.1814714	-0.3925216
C	-2.7088835	4.4701314	-0.4984336
H	-3.2746125	5.3966824	-0.5396646
C	-1.3229605	4.4982004	-0.5324196
C	-0.5423395	5.7085394	-0.6205016
H	-1.0690525	6.6591154	-0.6337816
C	0.8083655	5.6711964	-0.6962366
H	1.3812495	6.5915634	-0.7737446
C	1.5249595	4.4195074	-0.7035856
C	2.8964675	4.3152624	-0.8757456
H	3.5007305	5.2103474	-0.9941676
C	3.4872995	3.0440654	-0.9267696
H	4.5496635	2.9339324	-1.1074256
C	2.7094135	1.9066904	-0.7684826
C	0.7738685	3.2176874	-0.5751546
C	-0.6284335	3.2579944	-0.4909926
C	3.2919225	0.5335194	-0.8315366
C	4.0872375	0.0388884	0.2175474
C	4.4627955	-1.3063266	0.1916414
H	5.0549335	-1.7081086	1.0099174
C	4.0673245	-2.1662526	-0.8265756
C	3.3359115	-1.6348376	-1.8880366
H	3.0517405	-2.2851246	-2.7102066
C	2.9507785	-0.2976716	-1.9198766
C	4.5528575	0.9172144	1.3718414
H	3.8813765	1.7806134	1.4292034
C	2.2625785	0.2612084	-3.1602856
H	1.7049695	1.1553634	-2.8714736
C	-3.3068205	0.6547494	-2.8040506
H	-2.5412945	1.4226524	-2.6570256
C	-3.3376385	0.8916824	2.3027464
H	-2.3833225	1.4204194	2.1779284
C	-5.1510145	-3.1851526	-0.0791356
H	-5.3365105	-3.4087316	0.9796344
C	4.3713645	-3.6499826	-0.7435926
H	5.0855405	-3.7914586	0.0782304
C	0.6749775	-0.9046336	0.8009744

C	-0.2360735	-1.5506286	-0.0619226
H	1.7376955	-1.0751656	0.6331794
H	-1.2266565	-1.8324136	0.2959704
H	-1.0025985	-0.4758666	-1.1802726
H	0.1531085	-2.2029666	-0.8425386
C	0.2952875	-0.5227726	2.2175684
C	0.8086555	-1.5835206	3.2086654
C	0.7764285	0.8855394	2.6093334
H	-0.8011155	-0.5186246	2.2767694
C	0.5028725	-1.2267816	4.6654794
H	1.8954345	-1.6936216	3.0801644
H	0.3688005	-2.5551406	2.9513874
C	0.4885825	1.2211884	4.0743314
H	1.8529915	0.9671754	2.4135674
H	0.2960555	1.6308114	1.9645134
C	1.0473935	0.1583754	5.0226264
H	0.9207255	-1.9879166	5.3354164
H	-0.5854995	-1.2338666	4.8197664
H	0.9029265	2.2069994	4.3163584
H	-0.5988045	1.2940194	4.2186554
H	0.8044645	0.4072934	6.0626224
H	2.1443445	0.1464674	4.9471074
C	-3.2041245	-0.0409626	3.5139784
H	-2.7987125	0.5136554	4.3668024
H	-4.1787535	-0.4376956	3.8217214
H	-2.5423955	-0.8900936	3.3188674
C	-4.4255055	1.9343894	2.6101374
H	-4.4835655	2.7018114	1.8363854
H	-5.4044635	1.4466534	2.6874294
H	-4.2172645	2.4315954	3.5645704
C	5.9735185	1.4408734	1.1071224
H	6.2927995	2.1139354	1.9109674
H	6.6840785	0.6070904	1.0588774
H	6.0381045	1.9847744	0.1607954
C	4.5181715	0.2106094	2.7340134
H	5.3167085	-0.5341966	2.8269944
H	4.6617285	0.9425584	3.5365244
H	3.5648535	-0.2961396	2.9036194
C	-6.4976395	-3.2503556	-0.8109266
H	-6.9546275	-4.2391936	-0.6910766
H	-7.1941525	-2.4998296	-0.4233486
H	-6.3727795	-3.0696076	-1.8848196
C	-4.1796115	-4.2457226	-0.6163226
H	-3.2256975	-4.2127556	-0.0795826

H	-4.6032605	-5.2506996	-0.5075636
H	-3.9714015	-4.0811056	-1.6799696
C	3.0918685	-4.4233016	-0.3892056
H	2.3503715	-4.3311666	-1.1919596
H	3.3044145	-5.4887606	-0.2451836
H	2.6390145	-4.0312336	0.5276564
C	5.0172935	-4.2039206	-2.0187066
H	5.9257085	-3.6496946	-2.2760376
H	5.2833245	-5.2580376	-1.8831016
H	4.3326145	-4.1460156	-2.8725586
C	1.2585885	-0.7035376	-3.7990596
H	1.7500285	-1.5669316	-4.2621166
H	0.5395295	-1.0720286	-3.0598606
H	0.6970365	-0.1891586	-4.5863566
C	3.3295495	0.6975624	-4.1762016
H	4.0019565	1.4431294	-3.7389186
H	3.9344755	-0.1592796	-4.4963756
H	2.8609885	1.1373424	-5.0639906
C	-2.7298585	-0.3577596	-3.8000316
H	-3.4753085	-1.0957546	-4.1176806
H	-2.3828035	0.1609174	-4.7006536
H	-1.8798335	-0.8921836	-3.3645776
C	-4.5550595	1.3385624	-3.3796356
H	-5.3593745	0.6086524	-3.5313966
H	-4.9248625	2.1140484	-2.7012126
H	-4.3321445	1.8066814	-4.3452916

Supplementary Table 24 | TS-4′

Fe	0.2117289	0.4596963	0.2885261
N	-0.8229051	2.1668923	-0.0076859
N	1.7853709	1.6621843	-0.0390489
C	-3.0708781	1.2205273	-0.0476639
C	-3.1202421	0.3569073	-1.1521059
C	-4.0058781	-0.7214937	-1.1254079
H	-4.0364081	-1.3959927	-1.9783409
C	-4.8256921	-0.9720637	-0.0302249
C	-4.7540451	-0.1040377	1.0605841
H	-5.3797811	-0.2908807	1.9314491
C	-3.8939371	0.9916643	1.0688521
C	-2.1544941	2.3971823	-0.0716929
C	-2.6797931	3.6712003	-0.2286799
H	-3.7556331	3.7894833	-0.2954299

C	-1.8248821	4.7790343	-0.3451489
H	-2.2304241	5.7784193	-0.4761769
C	-0.4537071	4.5778383	-0.3317979
C	0.5181309	5.6294523	-0.5122919
H	0.1542209	6.6470743	-0.6283519
C	1.8449759	5.3661063	-0.5574599
H	2.5623719	6.1689553	-0.7062109
C	2.3483699	4.0215443	-0.4132269
C	3.6920229	3.6838593	-0.4581929
H	4.4430749	4.4538703	-0.6116529
C	4.0701489	2.3390793	-0.3015059
H	5.1140659	2.0461323	-0.3219939
C	3.1043309	1.3664313	-0.0964759
C	1.4022269	2.9791963	-0.2070019
C	0.0270439	3.2490263	-0.1661969
C	3.4908389	-0.0618207	0.0955091
C	3.5833489	-0.5910377	1.3900711
C	3.8913609	-1.9424227	1.5451001
H	3.9591269	-2.3578507	2.5488071
C	4.1034059	-2.7756437	0.4476891
C	4.0179589	-2.2212287	-0.8294319
H	4.1794509	-2.8576687	-1.6975889
C	3.7100569	-0.8760597	-1.0248879
C	3.2974139	0.2713083	2.5935191
H	3.5329149	-0.2543457	3.5232341
H	3.8721269	1.2030913	2.5664271
H	2.2374599	0.5526123	2.6179361
C	3.5531749	-0.3183387	-2.4166459
H	4.2734829	0.4824483	-2.6152029
H	3.6905569	-1.0978107	-3.1715809
H	2.5522239	0.1101813	-2.5445949
C	-2.2317341	0.5510943	-2.3563929
H	-2.6728511	0.0834943	-3.2415039
H	-2.0610341	1.6098393	-2.5710859
H	-1.2497241	0.0893543	-2.1935179
C	-3.8181121	1.8839873	2.2844141
H	-4.3384431	1.4297143	3.1327231
H	-2.7772831	2.0618213	2.5754721
H	-4.2697631	2.8643733	2.0981841
C	-5.7350061	-2.1744397	0.0051101
H	-5.3553211	-2.9304047	0.7040591
H	-6.7435491	-1.9049717	0.3370271
H	-5.8167271	-2.6429237	-0.9803079
C	4.3661869	-4.2490017	0.6325791

H	4.8240489	-4.4548877	1.6049811
H	3.4285089	-4.8165087	0.5817351
H	5.0301709	-4.6383207	-0.1458349
C	-0.5118641	-2.5471497	0.2627201
C	0.3611019	-3.2816817	-0.5496069
C	-0.1091111	-4.3143757	-1.3498309
C	-1.4683641	-4.6286437	-1.3612579
C	-2.3422491	-3.9076107	-0.5556329
C	-1.8659591	-2.8808337	0.2572061
H	1.4175579	-3.0211247	-0.5590429
H	0.5838269	-4.8721467	-1.9735759
H	-1.8393081	-5.4306317	-1.9929379
H	-3.4031411	-4.1434247	-0.5514269
H	-2.5589111	-2.3379877	0.8910641
C	0.0322879	-1.4605317	1.1421061
C	-0.7833211	-0.4905627	1.7633691
H	0.9591129	-1.7410417	1.6428311
H	-1.8539361	-0.4758167	1.5832021
H	1.0630559	-0.7453397	-0.1017679
H	-0.4744341	-0.0660207	2.7171381

Supplementary Table 25 | TS-4

Fe	-0.0319392	0.2578636	-0.5574650
N	-1.1945552	1.8262606	-0.1412460
N	1.4508438	1.6062476	-0.0636800
C	-3.2391062	0.6038876	-0.4650620
C	-3.6017002	0.2467746	-1.7724010
C	-4.1798842	-1.0015364	-1.9906120
H	-4.4568532	-1.2853854	-3.0042960
C	-4.4074972	-1.8963424	-0.9434690
C	-4.0509302	-1.5102954	0.3464360
H	-4.2243062	-2.1925274	1.1759480
C	-3.4618202	-0.2722764	0.6063500
C	-2.5413122	1.8960986	-0.2072380
C	-3.2271932	3.0809966	0.0103270
H	-4.3106352	3.0788876	-0.0394560
C	-2.5180542	4.2511206	0.3246840
H	-3.0448582	5.1837416	0.5066870
C	-1.1333222	4.2060376	0.4195000
C	-0.3059982	5.3342156	0.7694720
H	-0.7899532	6.2877466	0.9635320
C	1.0395778	5.2148996	0.8623310

H	1.6504648	6.0726976	1.1315430
C	1.7073178	3.9633546	0.5963570
C	3.0846988	3.7980846	0.6377280
H	3.7288878	4.6329156	0.8992860
C	3.6302728	2.5470936	0.3157710
H	4.7036068	2.3916276	0.3089040
C	2.7999828	1.4917186	-0.0325620
C	0.9095638	2.8406546	0.2463050
C	-0.4884002	2.9648876	0.1712160
C	3.3746068	0.1767196	-0.4317520
C	3.9610568	-0.6608254	0.5293990
C	4.4007818	-1.9276244	0.1414500
H	4.8287758	-2.5889434	0.8924730
C	4.2945128	-2.3713184	-1.1743550
C	3.7454248	-1.5045214	-2.1192380
H	3.6705008	-1.8269054	-3.1560940
C	3.2862228	-0.2356624	-1.7736540
C	4.1305408	-0.2349604	1.9689170
H	5.1066958	0.2421676	2.1197580
H	3.3735048	0.4891696	2.2805070
H	4.0787828	-1.0993654	2.6381590
C	2.7021978	0.6650246	-2.8346200
H	3.0697368	1.6910686	-2.7303740
H	2.9609838	0.3051256	-3.8347150
H	1.6093668	0.7079876	-2.7581900
C	-3.3316722	1.1828436	-2.9233440
H	-3.6690592	0.7501686	-3.8696260
H	-3.8365592	2.1451036	-2.7875130
H	-2.2578122	1.3890186	-3.0039700
C	-3.0621592	0.1047176	2.0103620
H	-3.4189852	-0.6338234	2.7319700
H	-1.9715172	0.1567466	2.1046340
H	-3.4616902	1.0865266	2.2864160
C	-5.0022162	-3.2573414	-1.2078240
H	-5.7764582	-3.2120944	-1.9806020
H	-4.2354112	-3.9607764	-1.5554890
H	-5.4500482	-3.6796854	-0.3030240
C	4.7920578	-3.7379444	-1.5725530
H	4.2903038	-4.0978804	-2.4759540
H	5.8691488	-3.7169234	-1.7787350
H	4.6248318	-4.4703864	-0.7763900
C	-0.0074962	-1.9128354	1.2934710
C	0.6034098	-1.3334324	2.4162340
C	0.1292508	-1.5747934	3.6994310

C	-0.9714342	-2.4076984	3.8947200
C	-1.5837162	-2.9960714	2.7917930
C	-1.1071052	-2.7530354	1.5070810
H	1.4469458	-0.6673874	2.2626210
H	0.6160048	-1.1064864	4.5503880
H	-1.3465392	-2.5960574	4.8962960
H	-2.4366712	-3.6553654	2.9306460
H	-1.5903252	-3.2293764	0.6594640
C	0.5304638	-1.6282084	-0.0587910
C	-0.2528562	-1.6959544	-1.2402250
H	1.6082288	-1.7213054	-0.1598600
H	-1.2798692	-2.0594614	-1.2013090
H	-0.8048332	-0.2303194	-1.7977690
H	0.2531948	-1.9270674	-2.1751100

Supplementary Table 26 | TS-5′

Fe	0.0831091	1.4487793	-0.2582017
N	1.3753601	2.9322693	0.0571913
N	-1.2553269	2.9490423	0.0661463
C	3.3016521	1.5164873	0.2311773
C	2.8369161	0.7140343	1.2696433
C	3.3063711	-0.5898477	1.4499673
C	4.2357561	-1.0689107	0.5293283
C	4.7261091	-0.2890017	-0.5288277
C	4.2610541	1.0188643	-0.6531787
C	2.7269501	2.8762243	0.0852163
C	3.5125891	4.0172793	0.0808993
H	4.5915881	3.9169013	0.1237233
C	2.9027741	5.2863873	0.1121713
H	3.5098291	6.1871813	0.1355863
C	1.5193741	5.3773353	0.1706023
C	0.7780321	6.6097403	0.3005393
H	1.3332711	7.5437073	0.3297983
C	-0.5728729	6.6143243	0.4085503
H	-1.1094499	7.5521113	0.5271323
C	-1.3393429	5.3910123	0.3647883
C	-2.7190689	5.3193743	0.4797273
H	-3.3022789	6.2245713	0.6252073
C	-3.3537919	4.0676623	0.3939513
H	-4.4326219	3.9831323	0.4587123
C	-2.6018349	2.9231113	0.1731773
C	-0.6253039	4.1748783	0.1844783

C	0.7725481	4.1692603	0.1274653
C	-3.2672529	1.6026923	-0.0067477
C	-3.4078229	1.0740873	-1.2887677
C	-3.9872849	-0.1794657	-1.4846377
C	-4.4204399	-0.8756407	-0.3523377
C	-4.2933619	-0.3736787	0.9454193
C	-3.7178209	0.8885943	1.0999813
C	0.0560201	-1.6747667	-0.8096017
C	-0.9858929	-2.3397387	-0.1523217
C	-0.8197439	-3.6345577	0.3223223
C	0.4001371	-4.2897957	0.1543493
C	1.4412901	-3.6374617	-0.4977497
C	1.2693671	-2.3408587	-0.9790017
H	-1.9322819	-1.8240827	-0.0076127
H	-1.6420479	-4.1304917	0.8306103
H	0.5358731	-5.2997697	0.5299383
H	2.3951131	-4.1382087	-0.6416977
H	2.0905281	-1.8510267	-1.4907127
C	-0.1760189	-0.3022417	-1.3714877
C	0.8720281	0.5212773	-1.8688907
H	-1.1284409	-0.2364727	-1.9004257
H	1.9108171	0.2436623	-1.7128637
H	-0.9153219	0.3165803	-0.0102667
H	0.6954351	1.1273693	-2.7547437
H	4.5985941	1.6608103	-1.4595667
H	2.1167161	1.1366553	1.9649103
H	4.5963131	-2.0866297	0.6300093
H	-3.0370599	1.6555843	-2.1261087
H	-4.8666409	-1.8571457	-0.4873147
H	-3.5843819	1.3265163	2.0828303
C	-4.1548679	-0.8110757	-2.8730387
C	-3.5824139	0.0770433	-3.9867387
C	-3.4238799	-2.1670647	-2.9195767
C	-5.6549249	-1.0279447	-3.1497427
H	-4.0859569	1.0489263	-4.0327357
H	-2.5078539	0.2507863	-3.8570537
H	-3.7212699	-0.4140967	-4.9559827
H	-3.8077599	-2.8664507	-2.1698677
H	-3.5526709	-2.6311707	-3.9045607
H	-2.3504289	-2.0474257	-2.7383947
H	-5.7991409	-1.4743447	-4.1408017
H	-6.1088849	-1.6980337	-2.4121037
H	-6.1972659	-0.0766077	-3.1188307
C	-4.7540549	-1.2266737	2.1343703

C	-3.9420289	-2.5367087	2.1567393
C	-4.5458829	-0.5148227	3.4782033
C	-6.2532729	-1.5485357	1.9868103
H	-4.0710969	-3.1074407	1.2304883
H	-2.8728499	-2.3288427	2.2780563
H	-4.2647969	-3.1721957	2.9900663
H	-5.1097189	0.4231023	3.5327093
H	-4.8941889	-1.1598097	4.2924013
H	-3.4882259	-0.2942377	3.6597083
H	-6.5962859	-2.1584107	2.8309123
H	-6.8465209	-0.6277097	1.9658933
H	-6.4634369	-2.1039567	1.0670153
C	5.7272461	-0.9080457	-1.5129177
C	5.0625411	-2.1057037	-2.2190317
C	6.1812561	0.0893173	-2.5875447
C	6.9748401	-1.3869627	-0.7460027
H	4.7379541	-2.8684097	-1.5032067
H	4.1840901	-1.7810807	-2.7886007
H	5.7649311	-2.5770017	-2.9167567
H	6.6745031	0.9628113	-2.1467597
H	6.8993371	-0.3946427	-3.2585207
H	5.3413551	0.4383463	-3.1984977
H	7.7023261	-1.8243217	-1.4400657
H	7.4560401	-0.5497967	-0.2285497
H	6.7285401	-2.1475237	0.0016113
C	2.8277641	-1.4014457	2.6599803
C	1.2891401	-1.4601697	2.6886833
C	3.3583951	-2.8411977	2.6440723
C	3.3380881	-0.7093797	3.9393813
H	0.8371711	-0.4641937	2.7418273
H	0.8993091	-1.9581757	1.7980153
H	0.9541181	-2.0207907	3.5696633
H	4.4529041	-2.8751957	2.6935013
H	2.9732381	-3.3812797	3.5159313
H	3.0275141	-3.3774117	1.7486653
H	3.0304471	-1.2766197	4.8261223
H	4.4317101	-0.6404457	3.9378973
H	2.9371161	0.3053153	4.0346703

Supplementary Table 27 | TS-5

Fe	0.1253196	0.6192059	-0.6530019
N	-1.2989534	1.9662269	-1.4147979

N	1.3529756	2.0691809	-1.3831839
C	-3.1910234	0.5726509	-0.9329659
C	-2.7837194	-0.5911881	-1.5814149
C	-3.2017724	-1.8430881	-1.1306319
C	-4.0239764	-1.8849691	-0.0021249
C	-4.4617394	-0.7357411	0.6636661
C	-4.0488774	0.4977559	0.1639971
C	-2.6331494	1.8884549	-1.3281229
C	-3.4538874	3.0046699	-1.5256619
H	-4.5294304	2.8888649	-1.4506829
C	-2.8824374	4.2374469	-1.7967009
H	-3.5045194	5.1171699	-1.9369719
C	-1.4911694	4.3502489	-1.8742299
C	-0.7984494	5.5911379	-2.0879749
H	-1.3856284	6.4910099	-2.2495639
C	0.5558156	5.6489129	-2.0647669
H	1.0686746	6.5958869	-2.2096009
C	1.3516666	4.4729399	-1.8259149
C	2.7446686	4.4862709	-1.7209029
H	3.2931396	5.4130309	-1.8612739
C	3.4115526	3.3026709	-1.4115999
H	4.4924536	3.2848489	-1.3205219
C	2.6970916	2.1214119	-1.2310939
C	0.6910066	3.2356659	-1.6526089
C	-0.7305654	3.1766929	-1.6776329
C	3.3435146	0.8595589	-0.8032929
C	4.3251556	0.8727619	0.1889241
C	4.8497086	-0.3172121	0.6937301
C	4.3563926	-1.5188811	0.1769581
C	3.3827666	-1.5699261	-0.8228299
C	2.8961616	-0.3598091	-1.3146559
C	-0.3033744	2.1579769	1.6326281
C	-1.6807784	2.1452029	1.9299391
C	-2.4020114	3.3240839	2.0563871
C	-1.7790094	4.5641309	1.9122831
C	-0.4078174	4.5992079	1.6652241
C	0.3162206	3.4220699	1.5318551
H	-2.1945934	1.1949369	2.0428301
H	-3.4674434	3.2752449	2.2729341
H	-2.3480894	5.4840819	2.0054511
H	0.1006506	5.5533609	1.5537271
H	1.3803956	3.4662379	1.3087741
C	0.4749116	0.9576569	1.3443561
C	-0.0558374	-0.3790931	1.1450251

H	1.5479896	1.0418179	1.5112601
H	-1.0963874	-0.5698821	1.4188061
H	-0.1773074	-0.9219681	-0.2527989
H	0.5913566	-1.1962071	1.4640691
H	-4.3300194	1.4208359	0.6575481
H	-2.1215174	-0.5004061	-2.4338449
H	-4.3251504	-2.8539331	0.3864241
H	4.6376226	1.8285169	0.5950601
H	4.7387256	-2.4534211	0.5791241
H	2.1563286	-0.3459851	-2.1065129
C	-5.3169664	-0.8658551	1.9298081
C	-5.6728824	0.5002009	2.5334671
C	-6.6256914	-1.6059581	1.5973411
C	-4.5259454	-1.6611531	2.9868731
H	-4.7771914	1.0581149	2.8304481
H	-6.2503634	1.1165569	1.8353211
H	-6.2835904	0.3562209	3.4313911
H	-6.4357904	-2.6043431	1.1899501
H	-7.2364264	-1.7228211	2.5003871
H	-7.2105794	-1.0478641	0.8579401
H	-5.1059214	-1.7482781	3.9132421
H	-4.2917054	-2.6738341	2.6427461
H	-3.5806694	-1.1587851	3.2215941
C	-2.7172734	-3.1462451	-1.7778529
C	-2.0002314	-2.8934401	-3.1117789
C	-1.7255314	-3.8213261	-0.8105549
C	-3.9039814	-4.0887961	-2.0491779
H	-2.6507154	-2.3768411	-3.8266949
H	-1.0915074	-2.2986561	-2.9782799
H	-1.7024444	-3.8491111	-3.5573069
H	-2.2052994	-4.0740091	0.1414981
H	-1.3274344	-4.7452481	-1.2478789
H	-0.8853994	-3.1509651	-0.5986499
H	-3.5484654	-5.0120181	-2.5213099
H	-4.4279744	-4.3719221	-1.1308549
H	-4.6304634	-3.6189851	-2.7214349
C	5.8992856	-0.3488681	1.8119241
C	6.3406976	1.0589429	2.2358241
C	5.3028366	-1.0576391	3.0433051
C	7.1440166	-1.1165971	1.3273971
H	6.7789926	1.6146439	1.3991451
H	5.5063746	1.6419989	2.6409931
H	7.1021856	0.9843979	3.0197231
H	5.0206756	-2.0915921	2.8186651

H	6.0341686	-1.0802761	3.8598871
H	4.4079726	-0.5342071	3.3971121
H	7.9053936	-1.1406731	2.1158241
H	6.9077726	-2.1522871	1.0622841
H	7.5800726	-0.6353111	0.4451161
C	2.8546216	-2.9239571	-1.3123859
C	2.1941096	-3.6536711	-0.1266739
C	1.7988956	-2.7614501	-2.4137189
C	4.0148276	-3.7726581	-1.8627919
H	2.9065856	-3.8411961	0.6835351
H	1.3689676	-3.0576381	0.2773931
H	1.7891566	-4.6197211	-0.4508669
H	2.2147816	-2.2878791	-3.3104769
H	1.4097206	-3.7436851	-2.7039209
H	0.9553916	-2.1578751	-2.0601239
H	3.6406146	-4.7406371	-2.2168499
H	4.5025426	-3.2669241	-2.7034709
H	4.7761526	-3.9682111	-1.1001839

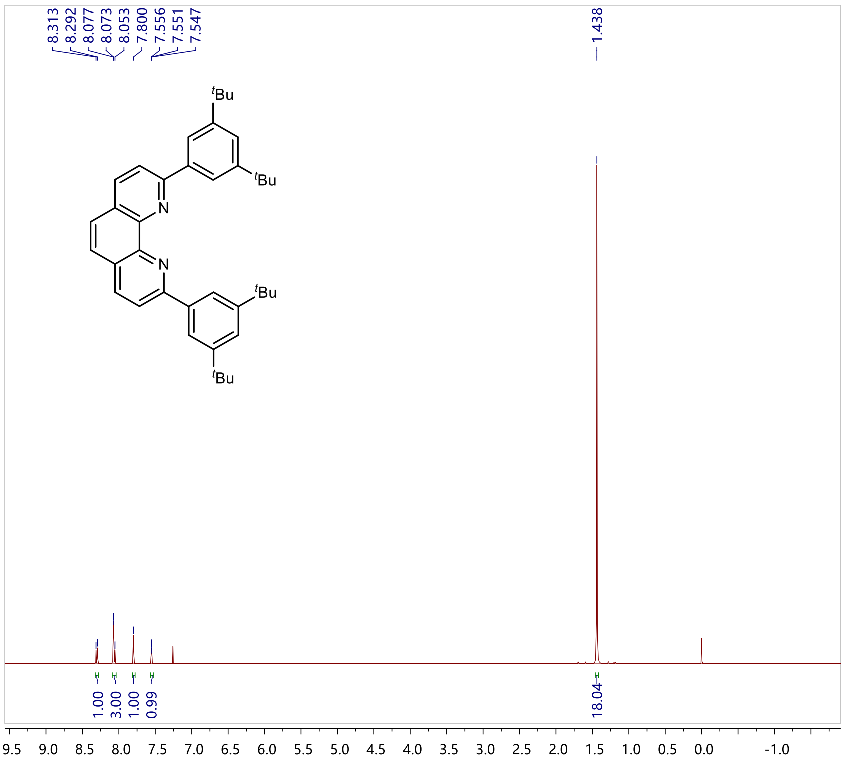

	Parameter	Value
1	Data File Name	Z:/ Data/ NMR/ 2016-01/ hmy-3,5-DitBu-Phen/ 1/ fid
2	Title	hmy-3,5-DitBu-Phen
3	Comment	PROTON
4	Origin	Bruker BioSpin GmbH
5	Owner	nmr
6	Site	
7	Spectrometer	spect
8	Author	
9	Solvent	CDCl3
10	Temperature	0.0
11	Pulse Sequence	zg30
12	Experiment	1D
13	Number of Scans	8
14	Receiver Gain	111
15	Relaxation Delay	1.0000
16	Pulse Width	9.1200
17	Acquisition Time	4.0894
18	Acquisition Date	2016-01-18T23:36:56
19	Modification Date	2016-01-19T07:37:00
20	Spectrometer Frequency	400.13
21	Spectral Width	8012.8
22	Lowest Frequency	-1546.3
23	Nucleus	1H
24	Acquired Size	32768
25	Spectral Size	65536

Supplementary Figure 5 | ^{1}H NMR (400 MHz, $CDCl_3$) spectra for compound 1b.

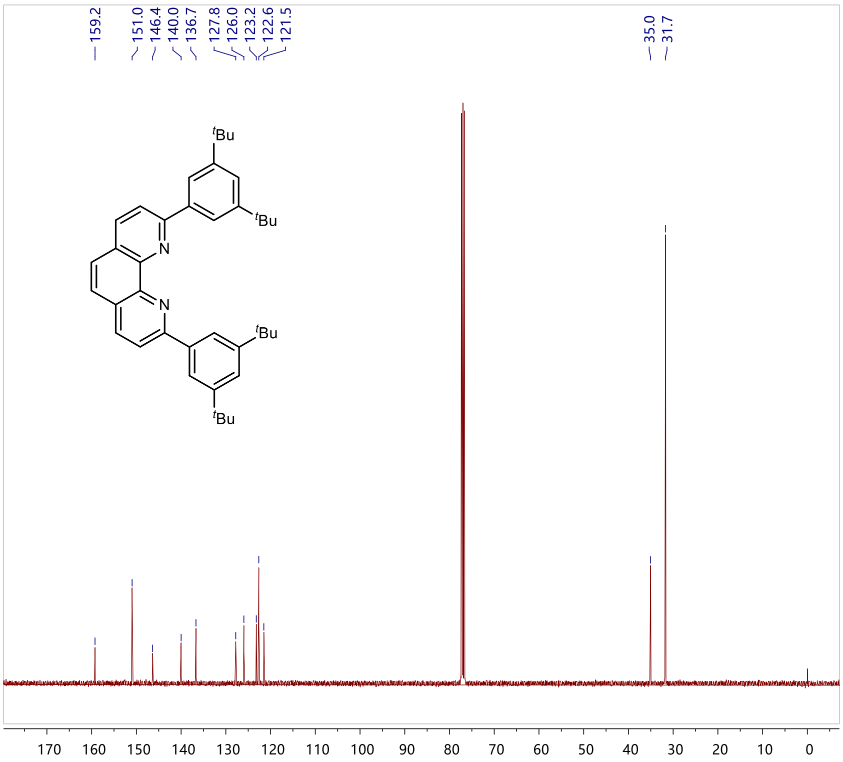

	Parameter	Value
1	Data File Name	Z:/ Data/ NMR/ 2016-01/ hmy-3,5-DitBu-Phen/ 3/ fid
2	Title	hmy-3,5-DitBu-Phen
3	Comment	C13CPD
4	Origin	Bruker BioSpin GmbH
5	Owner	nmr
6	Site	
7	Spectrometer	spect
8	Author	
9	Solvent	CDCl3
10	Temperature	0.0
11	Pulse Sequence	zgpg30
12	Experiment	1D
13	Number of Scans	681
14	Receiver Gain	27
15	Relaxation Delay	2.0000
16	Pulse Width	9.4000
17	Acquisition Time	1.3631
18	Acquisition Date	2016-01-19T05:13:26
19	Modification Date	2016-01-19T13:47:00
20	Spectrometer Frequency	100.61
21	Spectral Width	24038.5
22	Lowest Frequency	-1960.3
23	Nucleus	13C
24	Acquired Size	32768
25	Spectral Size	65536

Supplementary Figure 6 | ^{13}C NMR (101 MHz, $CDCl_3$) spectra for compound 1b.

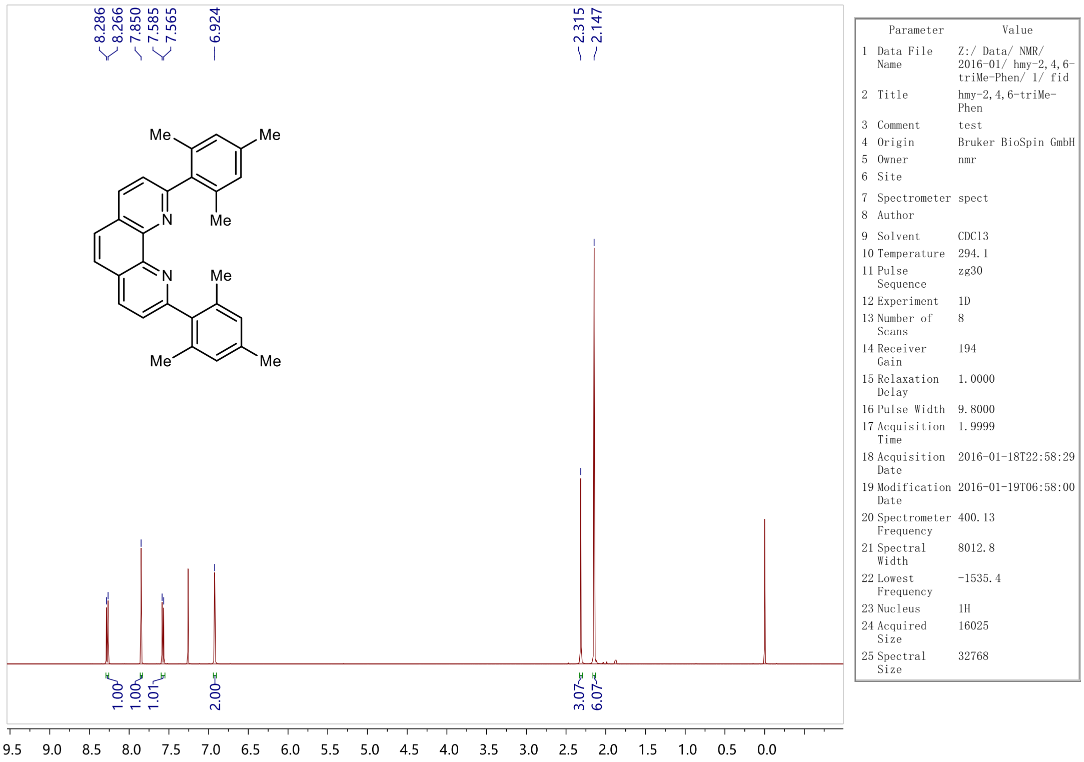

Supplementary Figure 7 | ^{1}H NMR (400 MHz, $CDCl_3$) spectra for compound 1c.

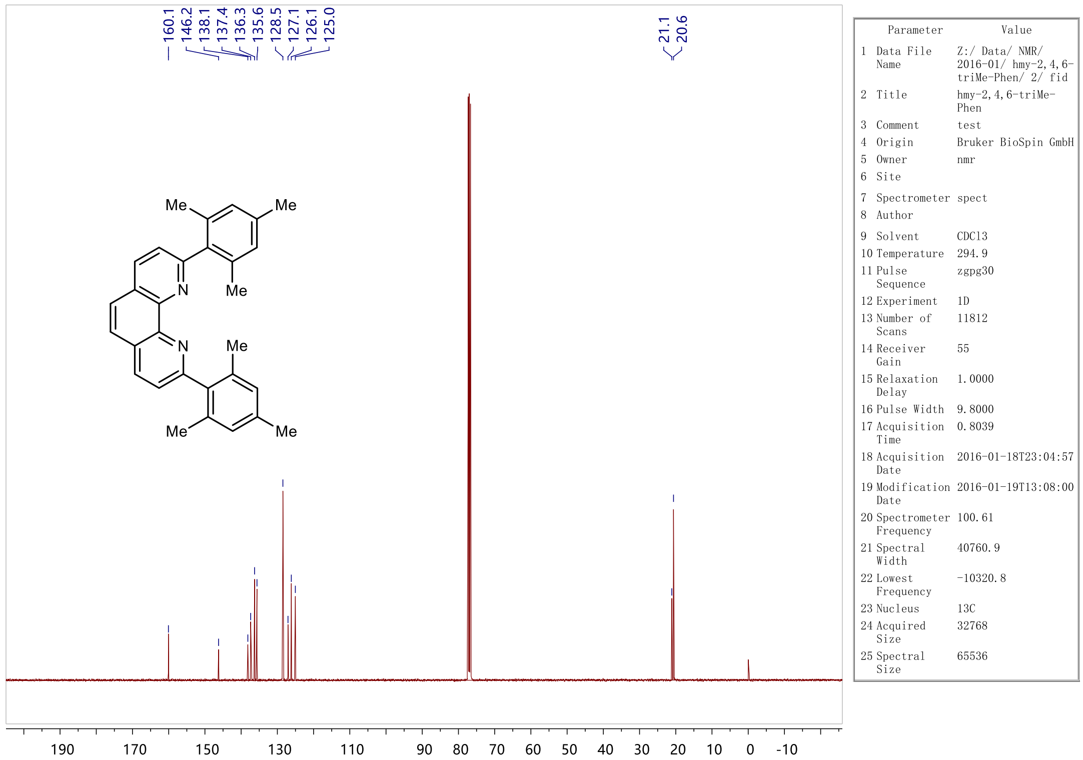

Supplementary Figure 8 | ^{13}C NMR (101 MHz, $CDCl_3$) spectra for compound 1c.

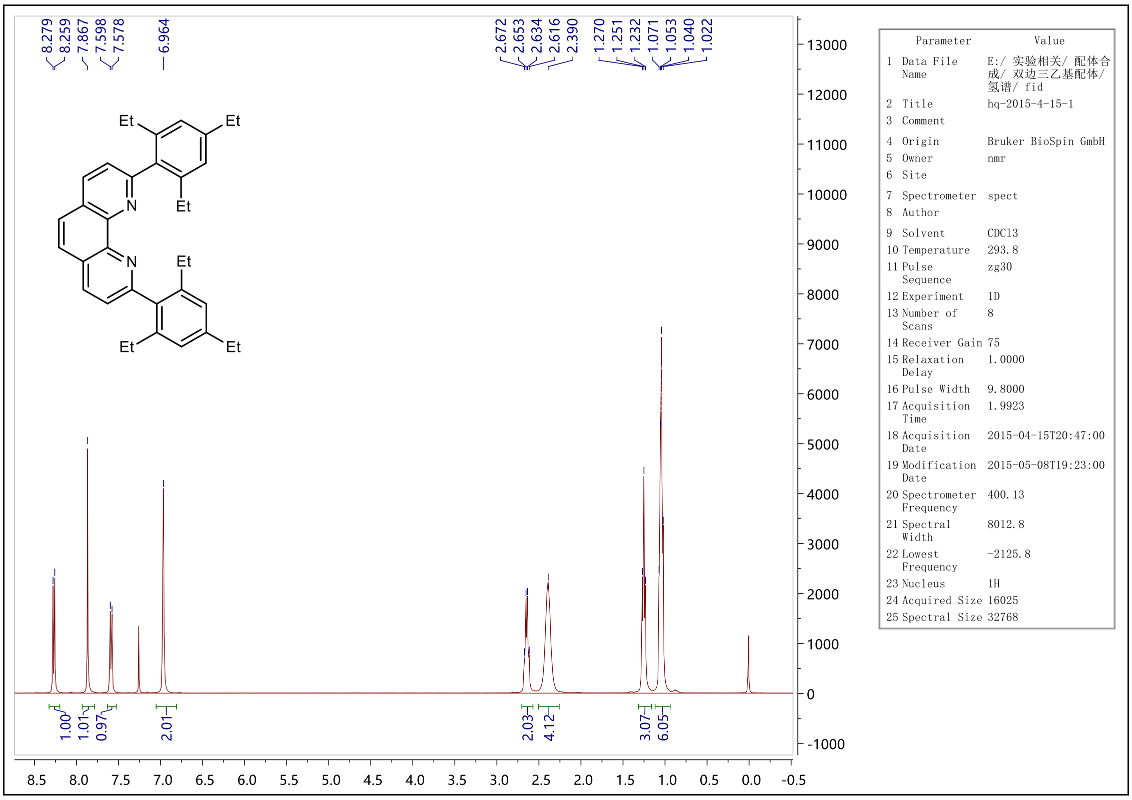

Supplementary Figure 9 | ^{1}H NMR (400 MHz, $CDCl_3$) spectra for compound 1d.

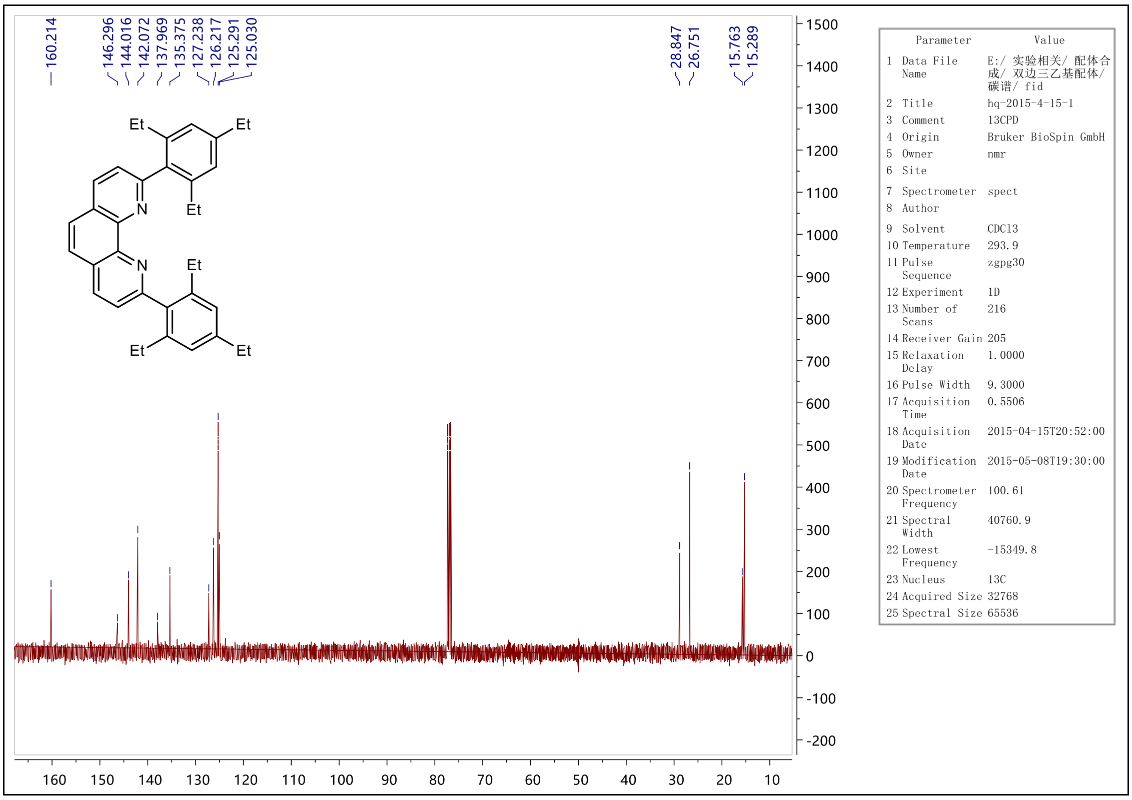

Supplementary Figure 10 | ^{13}C NMR (101 MHz, $CDCl_3$) spectra for compound 1d.

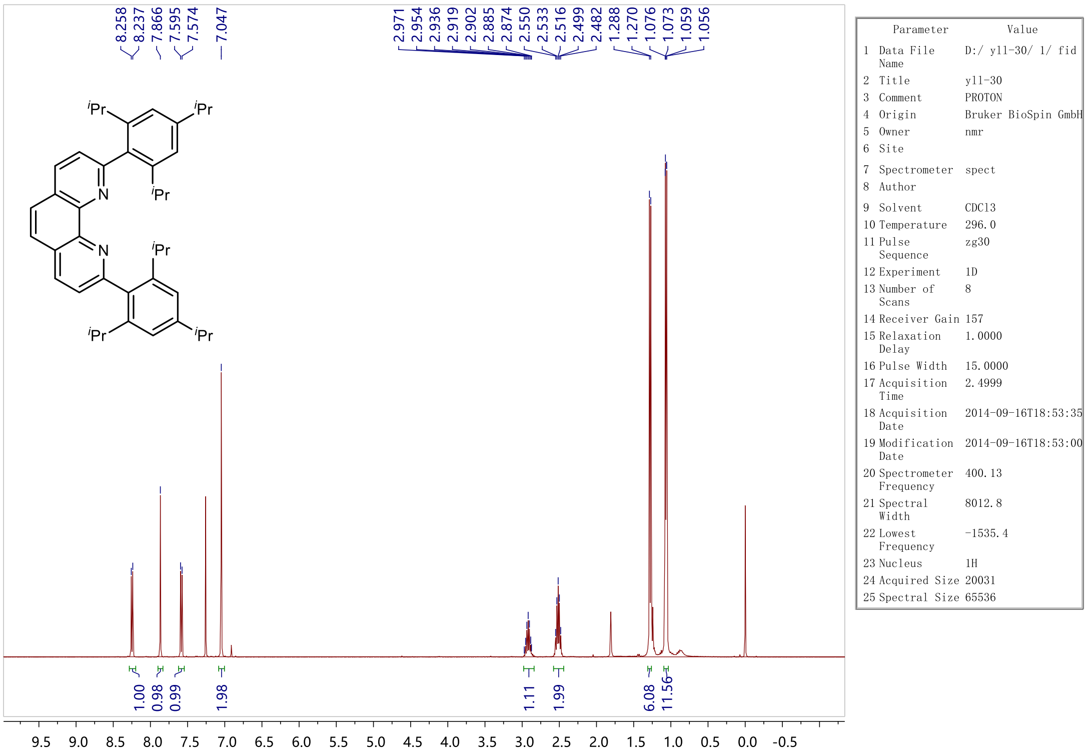

Supplementary Figure 11 | ^{1}H NMR (400 MHz, $CDCl_3$) spectra for compound 1e.

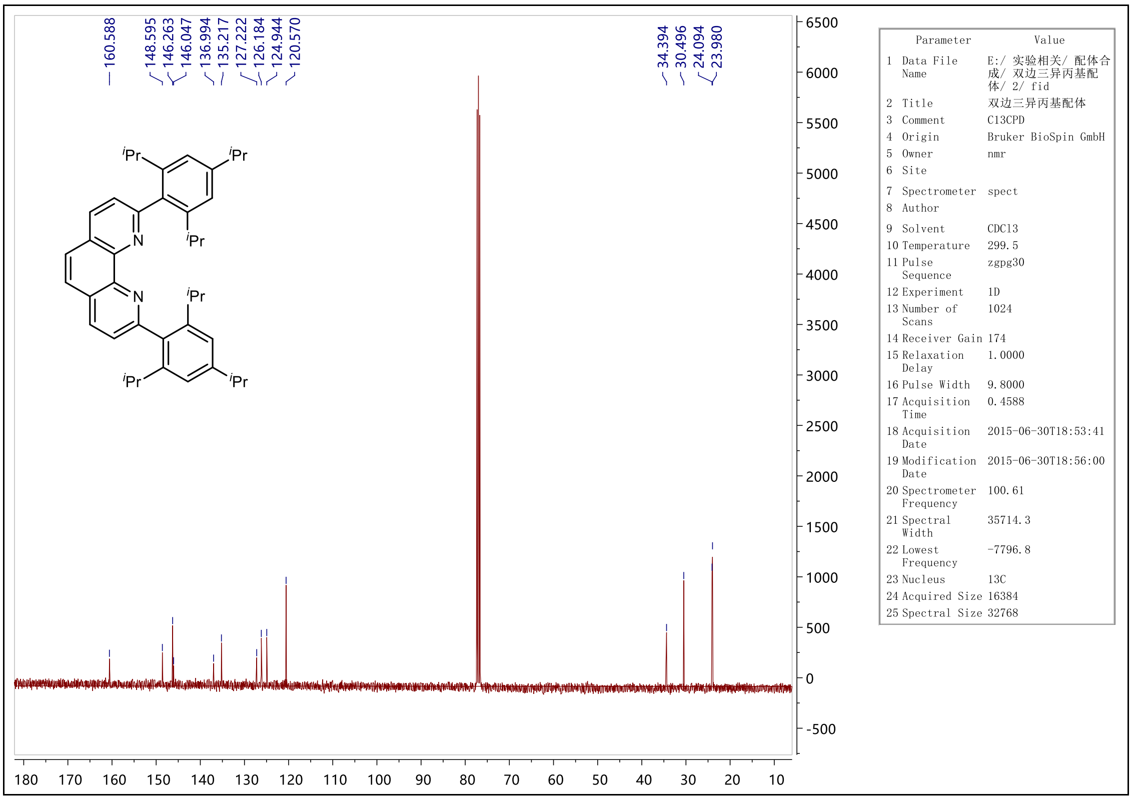

Supplementary Figure 12 | ^{13}C NMR (101 MHz, $CDCl_3$) spectra for compound 1e.

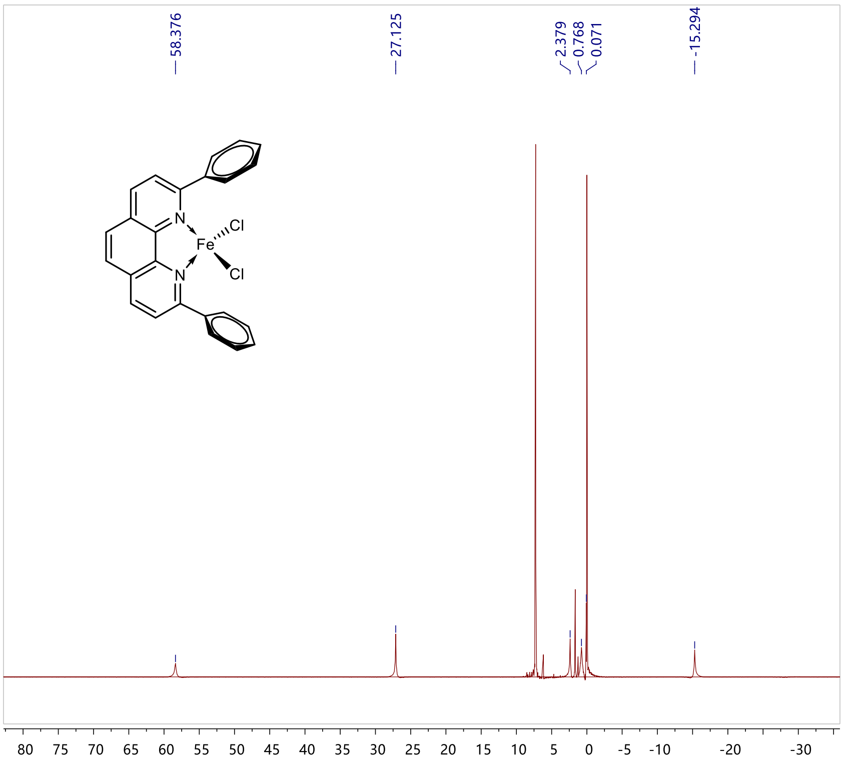

	Parameter	Value
1	Data File Name	Z:/ Data/ NMR/ 2016-02/ hmy-Ph-phen/ 1/ fid
2	Title	hmy-Ph-phen
3	Comment	test
4	Origin	Bruker BioSpin GmbH
5	Owner	nmr
6	Site	
7	Spectrometer	spect
8	Author	
9	Solvent	CDCl3
10	Temperature	292.4
11	Pulse Sequence	zg30
12	Experiment	1D
13	Number of Scans	73
14	Receiver Gain	194
15	Relaxation Delay	1.0000
16	Pulse Width	9.8000
17	Acquisition Time	0.1966
18	Acquisition Date	2016-02-16T08:59:26
19	Modification Date	2016-02-16T09:00:00
20	Spectrometer Frequency	400.13
21	Spectral Width	81521.7
22	Lowest Frequency	-38293.9
23	Nucleus	1H
24	Acquired Size	16025
25	Spectral Size	32768

Supplementary Figure 13 | ^{1}H NMR (400 MHz, $CDCl_3$) spectra for compound 2a.

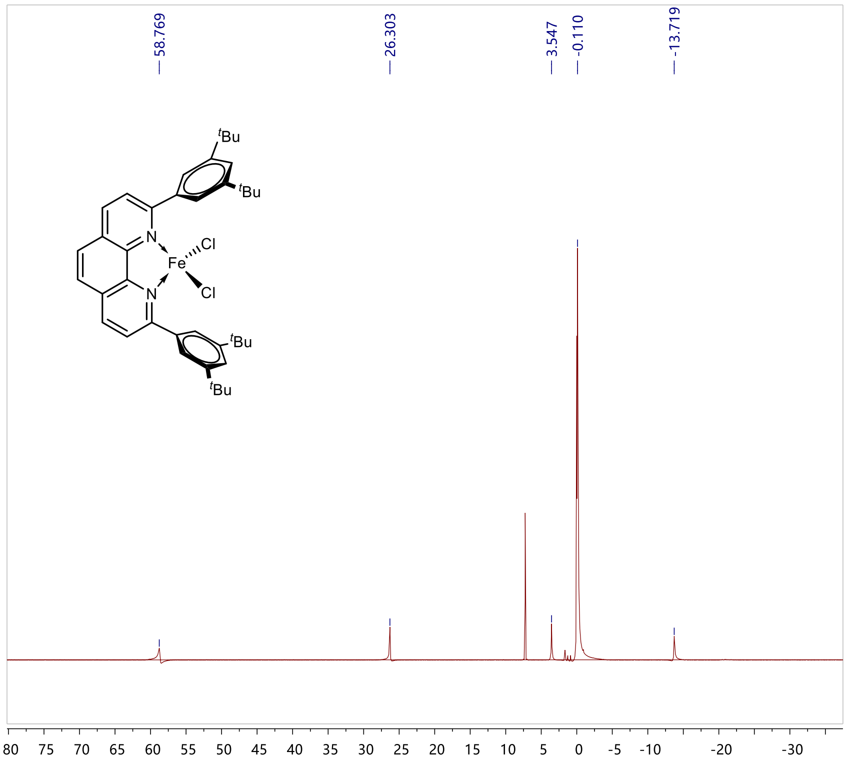

	Parameter	Value
1	Data File Name	Z:/ Data/ NMR/ 2016-01/ hmy-tbu/ 1/ fid
2	Title	hmy-tbu
3	Comment	PROTON
4	Origin	Bruker BioSpin GmbH
5	Owner	nmr
6	Site	
7	Spectrometer	spect
8	Author	
9	Solvent	CDCl3
10	Temperature	0.0
11	Pulse Sequence	zg30
12	Experiment	1D
13	Number of Scans	91
14	Receiver Gain	126
15	Relaxation Delay	1.0000
16	Pulse Width	9.1200
17	Acquisition Time	0.4020
18	Acquisition Date	2016-01-18T23:07:47
19	Modification Date	2016-01-19T07:08:00
20	Spectrometer Frequency	400.13
21	Spectral Width	81521.7
22	Lowest Frequency	-38254.6
23	Nucleus	1H
24	Acquired Size	32768
25	Spectral Size	65536

Supplementary Figure 14 | ^{1}H NMR (400 MHz, $CDCl_3$) spectra for compound 2b.

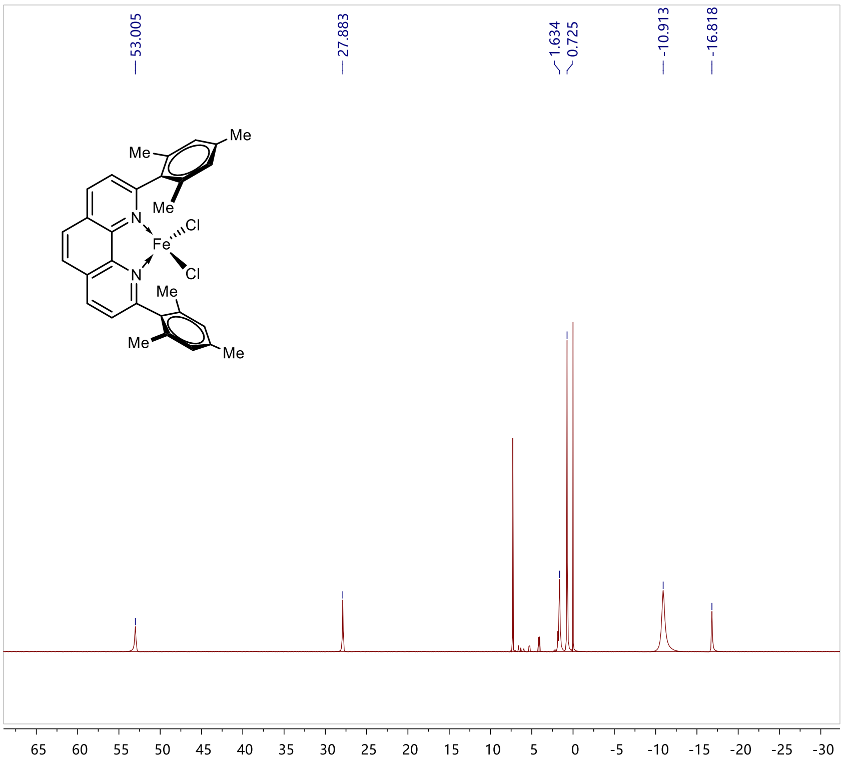

	Parameter	Value
1	Data File Name	Z:/ Data/ NMR/ 2016-01/ hmy-Me/ 1/ fid
2	Title	hmy-Me
3	Comment	PROTON
4	Origin	Bruker BioSpin GmbH
5	Owner	nmr
6	Site	
7	Spectrometer	spect
8	Author	
9	Solvent	CDCl3
10	Temperature	0.0
11	Pulse Sequence	zg30
12	Experiment	1D
13	Number of Scans	205
14	Receiver Gain	141
15	Relaxation Delay	1.0000
16	Pulse Width	9.1200
17	Acquisition Time	0.4020
18	Acquisition Date	2016-01-18T23:30:32
19	Modification Date	2016-01-19T07:33:00
20	Spectrometer Frequency	400.13
21	Spectral Width	81521.7
22	Lowest Frequency	-38288.3
23	Nucleus	1H
24	Acquired Size	32768
25	Spectral Size	65536

Supplementary Figure 15 | ^{1}H NMR (400 MHz, $CDCl_3$) spectra for compound 2c.

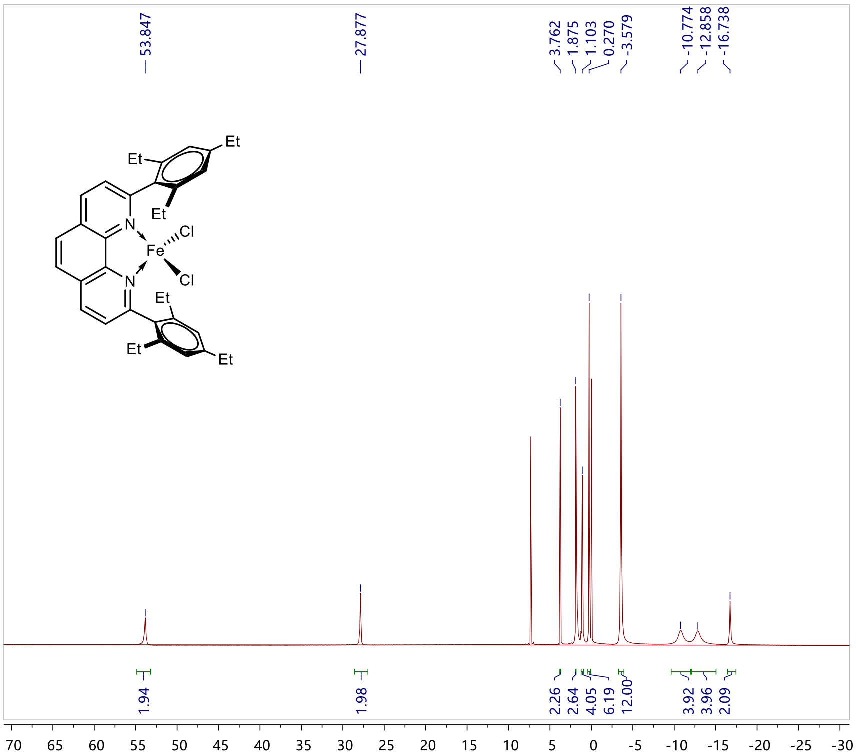

	Parameter	Value
1	Data File Name	C:/ Users/ 萌羊/ Desktop/ hmy-2b/ 1/ fid
2	Title	hmy-2b
3	Comment	PROTON
4	Origin	Bruker BioSpin GmbH
5	Owner	nmr
6	Site	
7	Spectrometer	spect
8	Author	
9	Solvent	CDCl3
10	Temperature	291.2
11	Pulse Sequence	zg30
12	Experiment	1D
13	Number of Scans	362
14	Receiver Gain	141
15	Relaxation Delay	1.0000
16	Pulse Width	9.1200
17	Acquisition Time	0.4020
18	Acquisition Date	2016-03-13T11:07:19
19	Modification Date	2016-03-13T11:13:00
20	Spectrometer Frequency	400.13
21	Spectral Width	81521.7
22	Lowest Frequency	-38289.9
23	Nucleus	1H
24	Acquired Size	32768
25	Spectral Size	65536

Supplementary Figure 16 | ^{1}H NMR (400 MHz, $CDCl_3$) spectra for compound 2d.

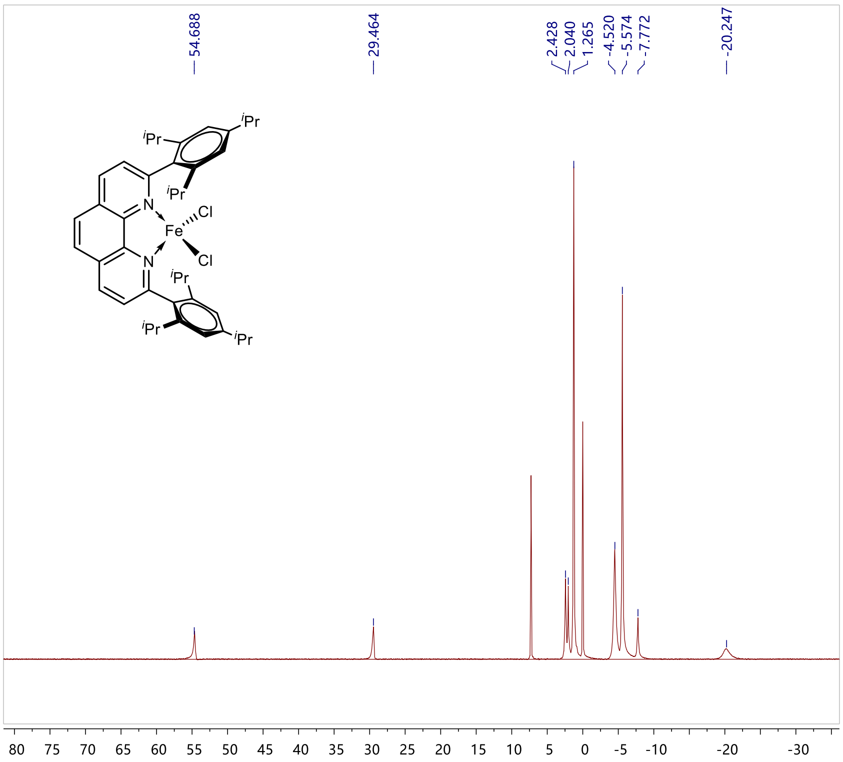

Parameter		Value
1	Data File Name	Z:/ Data/ NMR/ 2016-01/ hmy-iPr/ 2/ fid
2	Title	hmy-iPr
3	Comment	PROTON
4	Origin	Bruker BioSpin GmbH
5	Owner	nmr
6	Site	
7	Spectrometer	spect
8	Author	
9	Solvent	CDCl3
10	Temperature	0.0
11	Pulse Sequence	zg30
12	Experiment	1D
13	Number of Scans	129
14	Receiver Gain	141
15	Relaxation Delay	1.0000
16	Pulse Width	9.1200
17	Acquisition Time	0.4020
18	Acquisition Date	2016-01-18T23:15:50
19	Modification Date	2016-01-19T07:17:00
20	Spectrometer Frequency	400.13
21	Spectral Width	81521.7
22	Lowest Frequency	-38291.2
23	Nucleus	1H
24	Acquired Size	32768
25	Spectral Size	65536

Supplementary Figure 17 | ^{1}H NMR (400 MHz, $CDCl_3$) spectra for compound 2e.

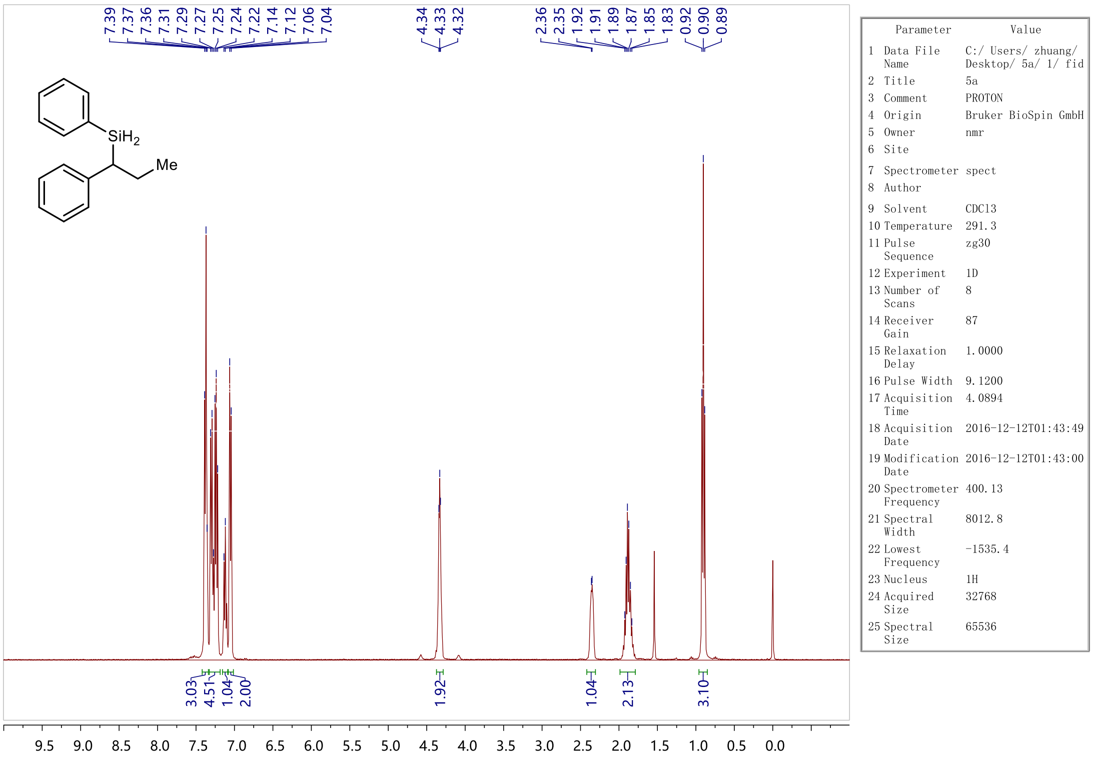

Supplementary Figure 18 | ^{1}H NMR (400 MHz, $CDCl_3$) spectra for compound 8a.

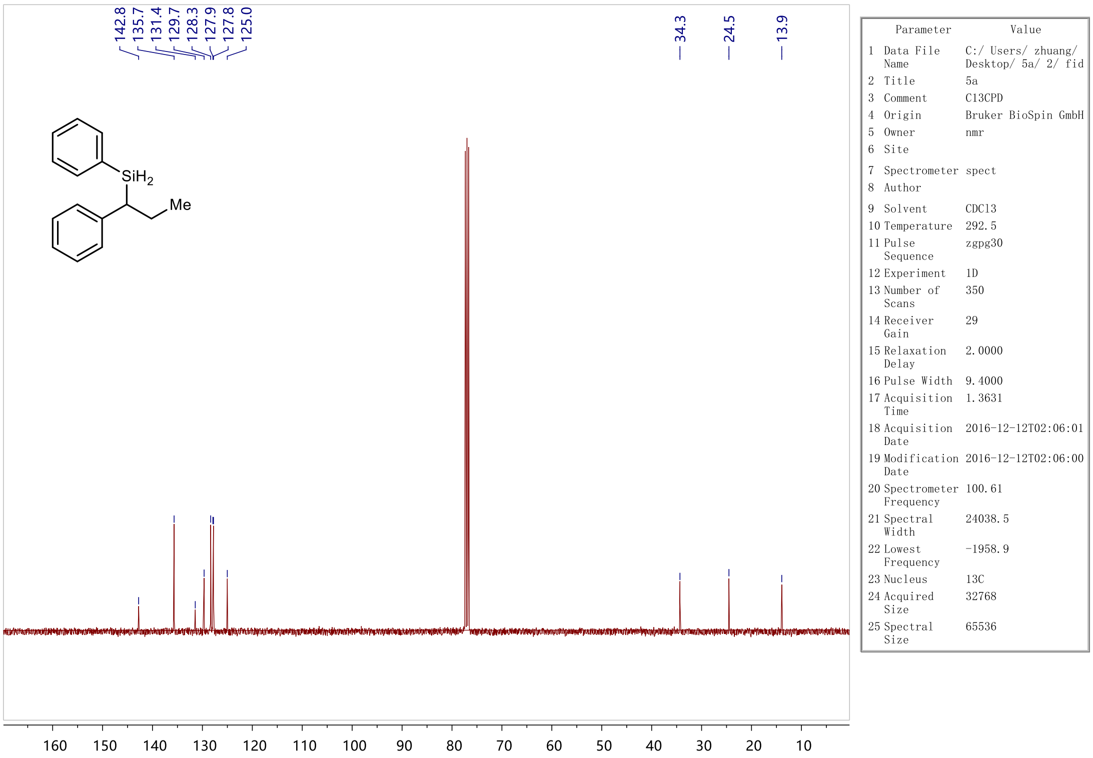

Supplementary Figure 19 | ^{13}C NMR (101 MHz, $CDCl_3$) spectra for compound 8a.

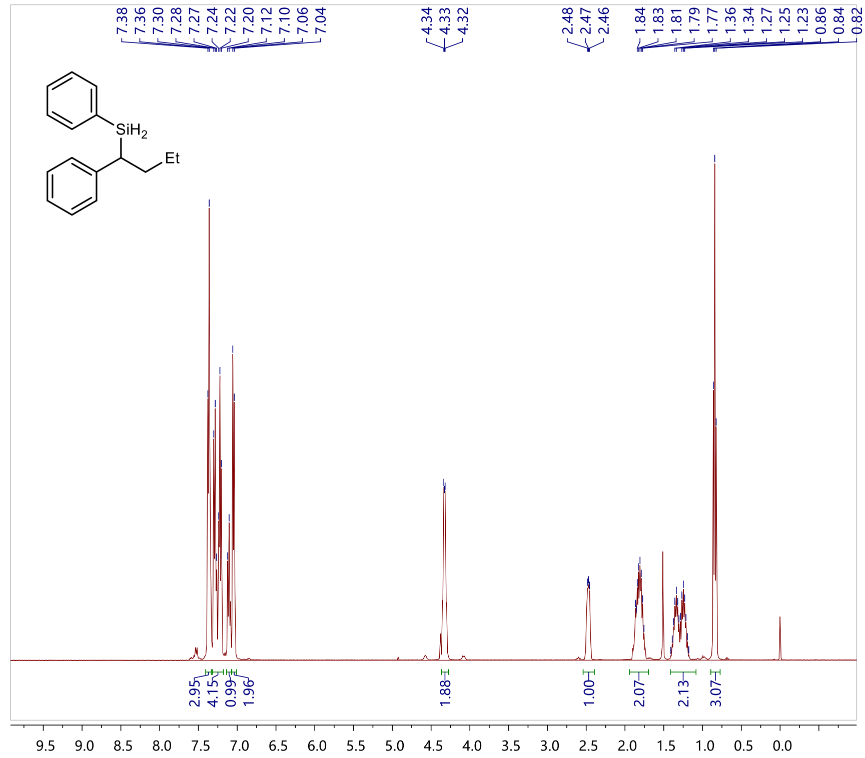

	Parameter	Value
1	Data File Name	C:/ Users/ yuanyuan/ Desktop/ 新建文件夹 (3)/ 产物nmr/ b (β-Et)/ 1/ fid
2	Title	b (β-Et)
3	Comment	PROTON
4	Origin	Bruker BioSpin GmbH
5	Owner	nmr
6	Site	
7	Spectrometer	spect
8	Author	
9	Solvent	CDCl3
10	Temperature	0.0
11	Pulse Sequence	zg30
12	Experiment	1D
13	Number of Scans	8
14	Receiver Gain	87
15	Relaxation Delay	1.0000
16	Pulse Width	15.0000
17	Acquisition Time	2.4999
18	Acquisition Date	2016-06-11T23:34:14
19	Modification Date	2016-06-11T23:34:00
20	Spectrometer Frequency	400.13
21	Spectral Width	8012.8
22	Lowest Frequency	-1604.4
23	Nucleus	1H
24	Acquired Size	20031
25	Spectral Size	65536

Supplementary Figure 20 | ^{1}H NMR (400 MHz, $CDCl_3$) spectra for compound 8b.

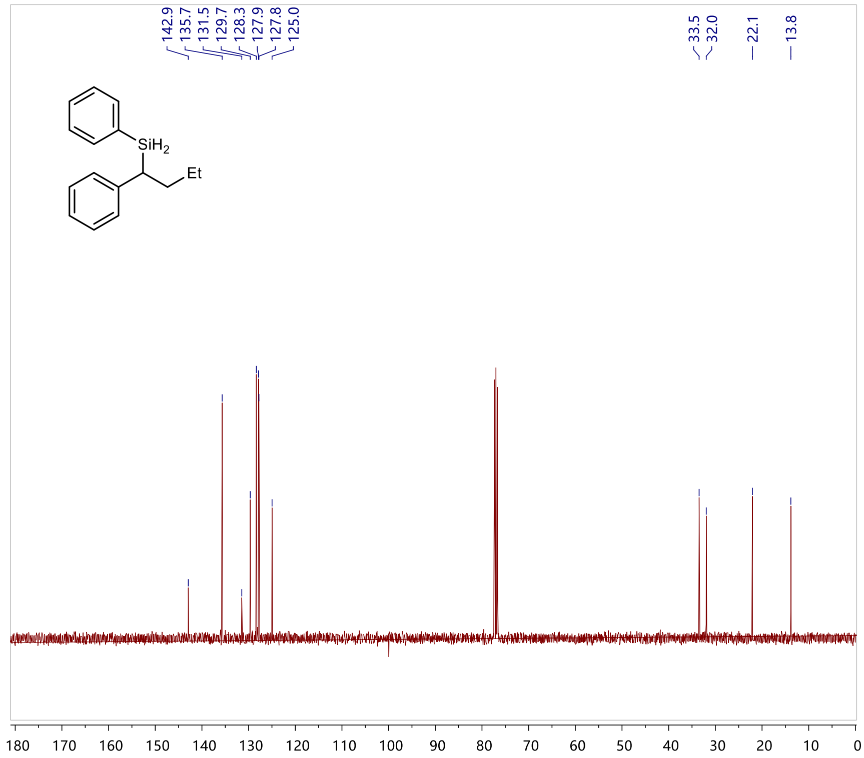

	Parameter	Value
1	Data File Name	C:/ Users/ yuanyuan/ Desktop/ 新建文件夹 (3)/ 产物nmr/ b (β-Et)/ 2/ fid
2	Title	b (β-Et)
3	Comment	C13CPD
4	Origin	Bruker BioSpin GmbH
5	Owner	nmr
6	Site	
7	Spectrometer	spect
8	Author	
9	Solvent	CDCl3
10	Temperature	0.0
11	Pulse Sequence	zgpg30
12	Experiment	1D
13	Number of Scans	130
14	Receiver Gain	51
15	Relaxation Delay	1.0000
16	Pulse Width	9.8000
17	Acquisition Time	0.4588
18	Acquisition Date	2016-06-11T23:38:37
19	Modification Date	2016-06-11T23:39:00
20	Spectrometer Frequency	100.61
21	Spectral Width	35714.3
22	Lowest Frequency	-7796.8
23	Nucleus	13C
24	Acquired Size	16384
25	Spectral Size	32768

Supplementary Figure 21 | ^{13}C NMR (101 MHz, $CDCl_3$) spectra for compound 8b.

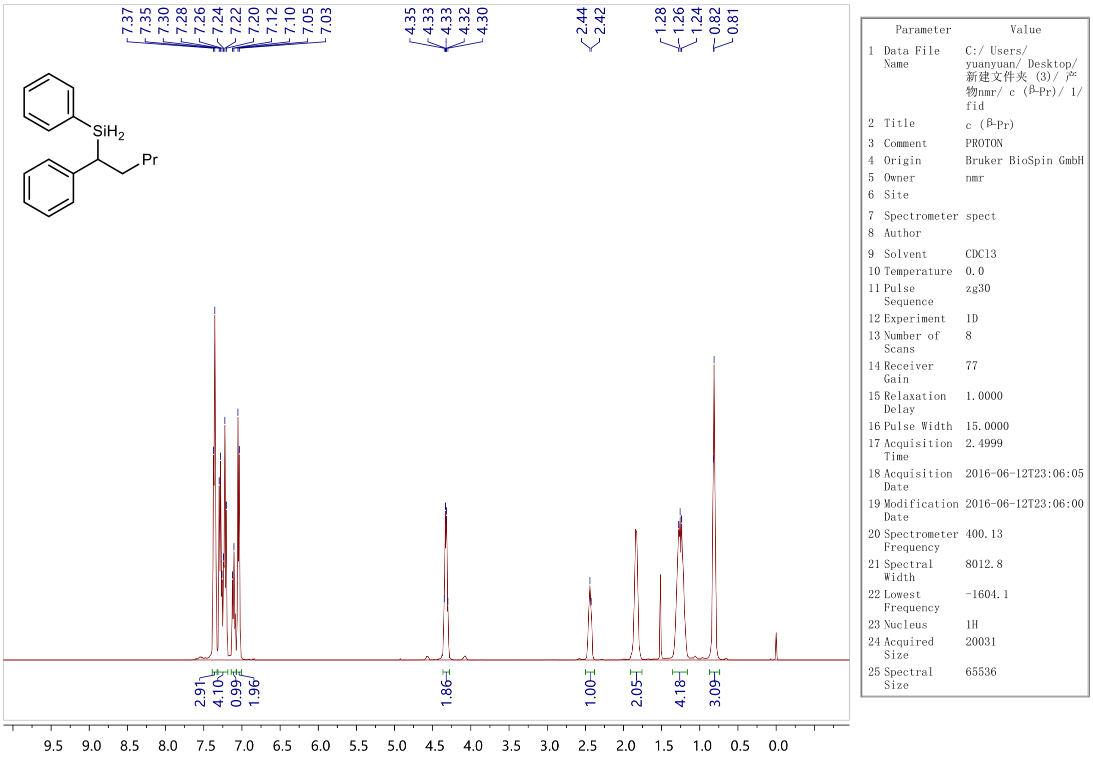

Supplementary Figure 22 | ^{1}H NMR (400 MHz, $CDCl_3$) spectra for compound 8c.

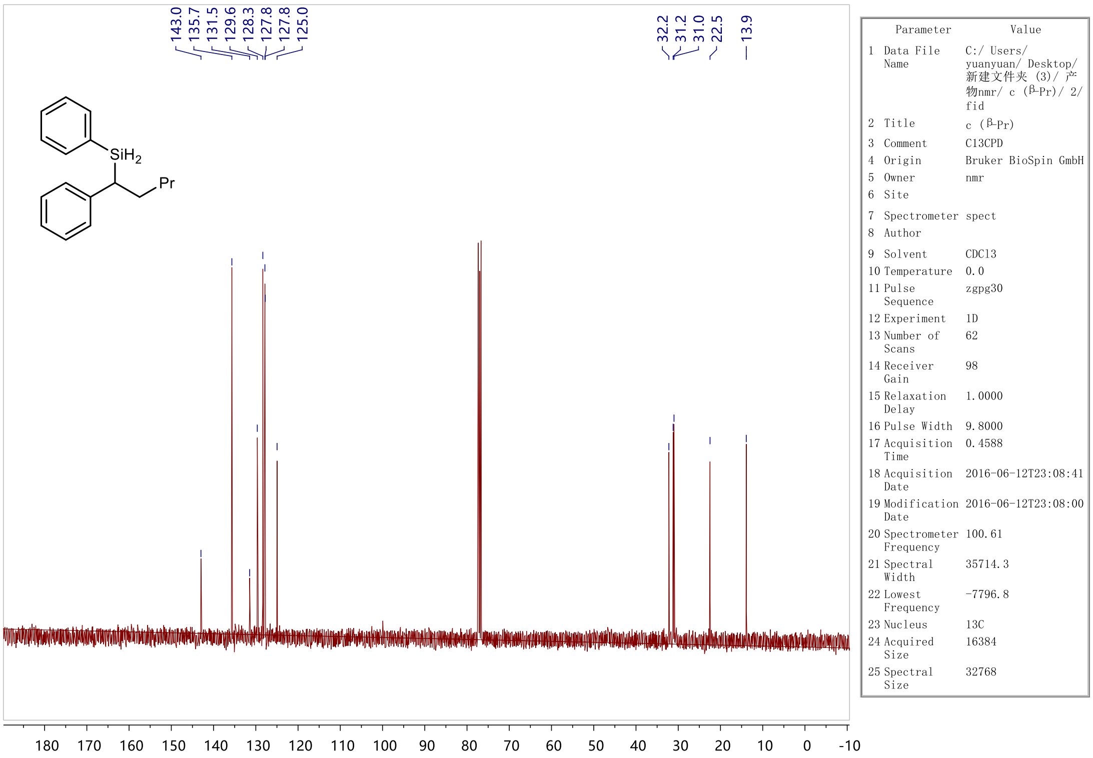

Supplementary Figure 23 | ^{13}C NMR (101 MHz, $CDCl_3$) spectra for compound 8c

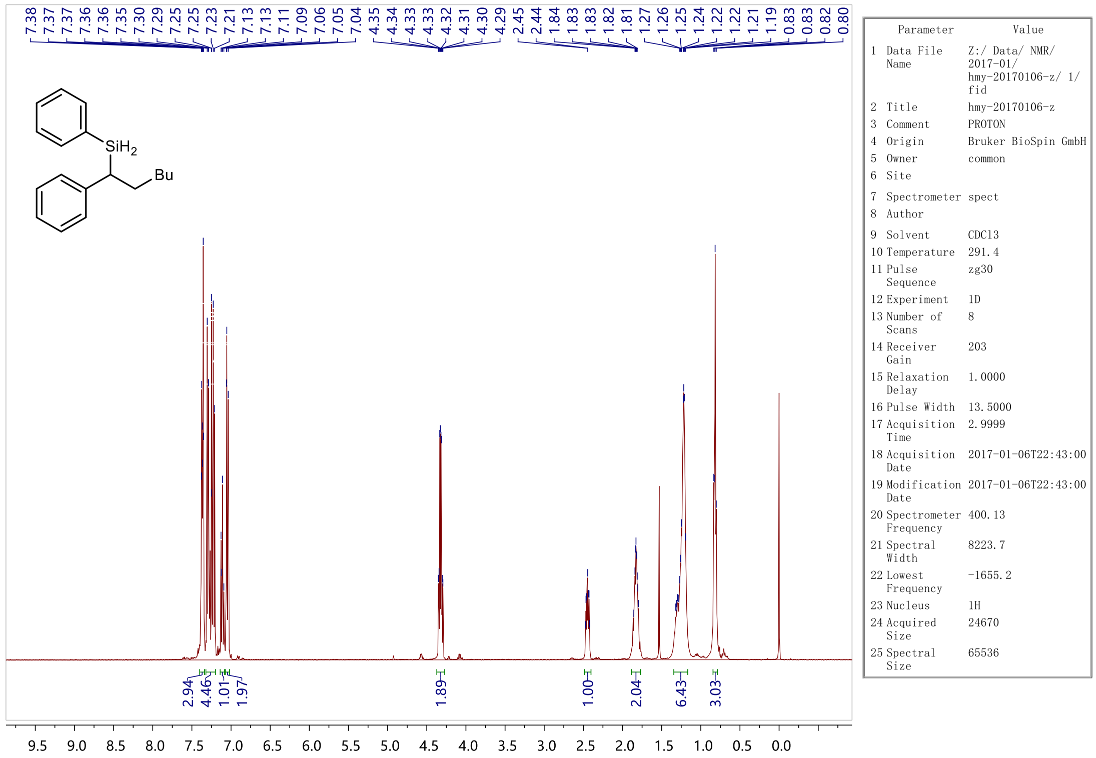

Supplementary Figure 24 | ^{1}H NMR (400 MHz, $CDCl_3$) spectra for compound 8d.

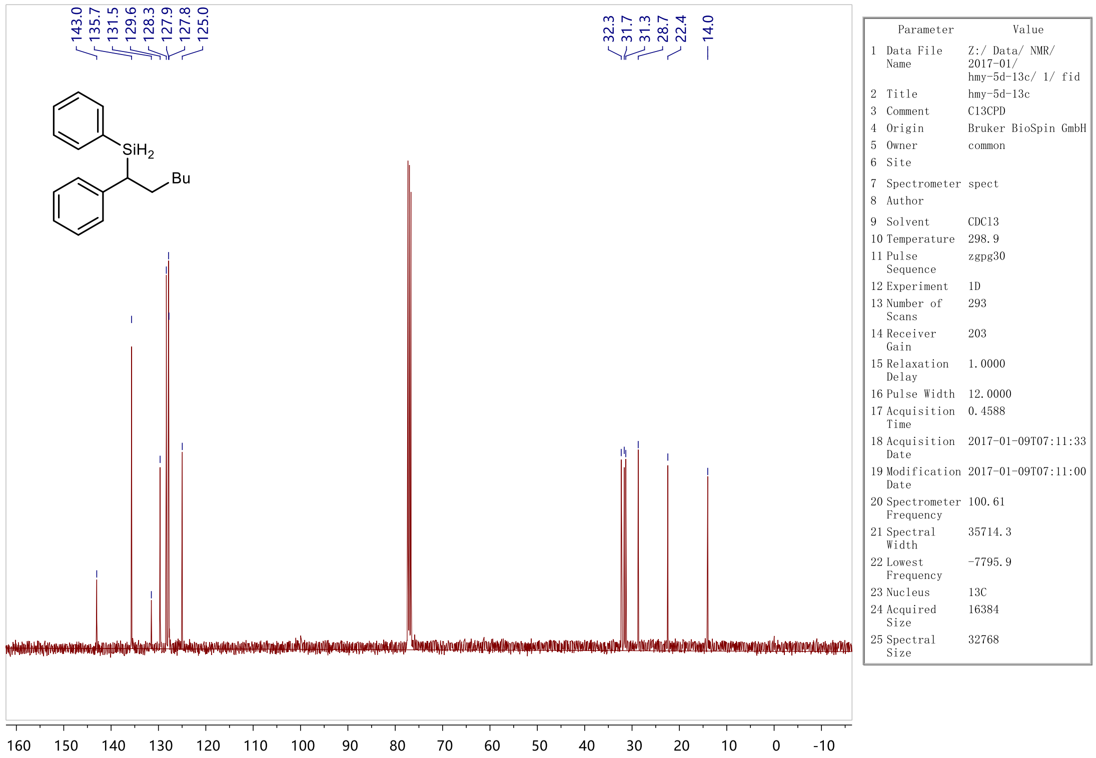

Supplementary Figure 25 | ^{13}C NMR (101 MHz, $CDCl_3$) spectra for compound 8d.

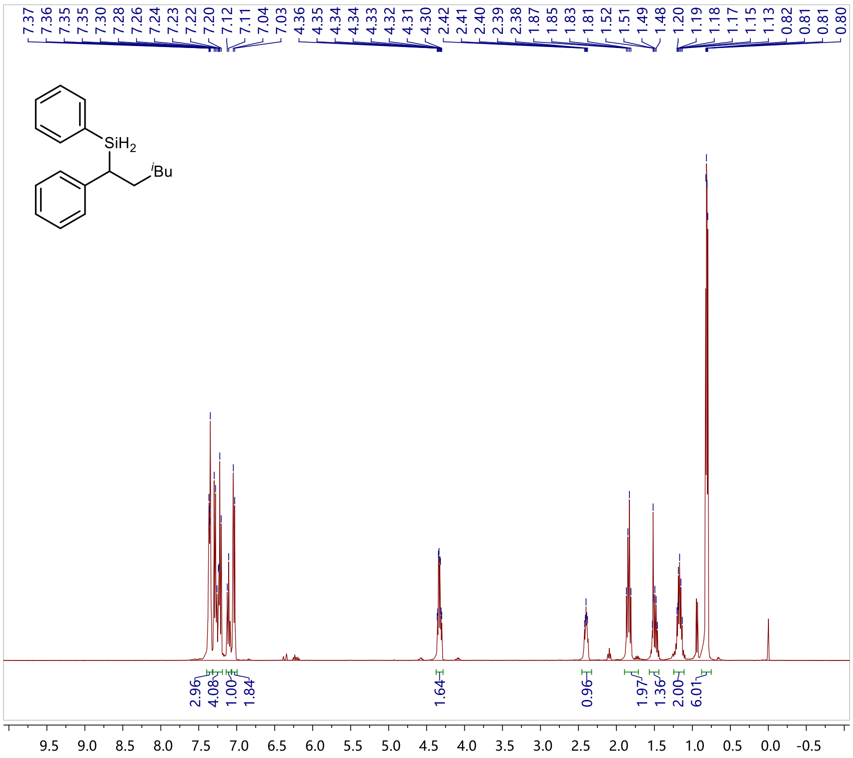

Parameter		Value
1	Data File Name	C:/ Users/ yuanyuan/ Desktop/ 新建文件夹 (3)/ 产物nmr/ e (β-iBu)/ 1/ fid
2	Title	hmy-2016-7-29-1a
3	Comment	PROTON
4	Origin	Bruker BioSpin GmbH
5	Owner	common
6	Site	
7	Spectrometer	spect
8	Author	
9	Solvent	CDCl3
10	Temperature	-2569.2
11	Pulse Sequence	zg30
12	Experiment	1D
13	Number of Scans	8
14	Receiver Gain	101
15	Relaxation Delay	1.0000
16	Pulse Width	13.7000
17	Acquisition Time	1.9999
18	Acquisition Date	2016-07-29T22:30:00
19	Modification Date	2016-07-29T22:30:00
20	Spectrometer Frequency	400.13
21	Spectral Width	8223.7
22	Lowest Frequency	-1660.9
23	Nucleus	1H
24	Acquired Size	16446
25	Spectral Size	65536

Supplementary Figure 26 | ^{1}H NMR (400 MHz, $CDCl_3$) spectra for compound 8e.

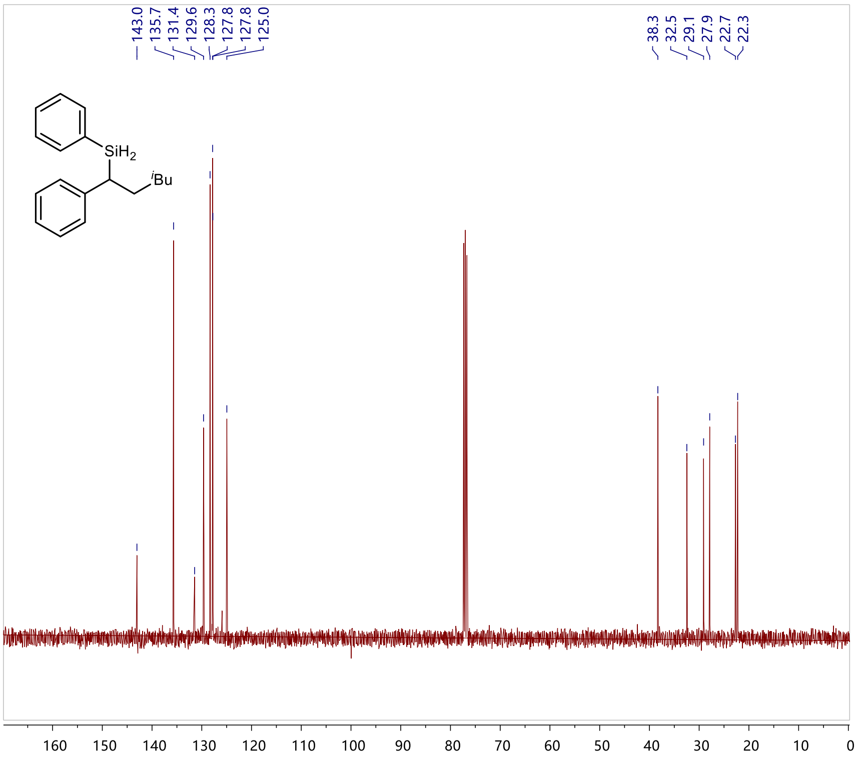

Parameter		Value
1	Data File Name	C:/ Users/ yuanyuan/ Desktop/ 新建文件夹 (3)/ 产物nmr/ e (β-iBu)/ 2/ fid
2	Title	hmy-2016-7-29-1a
3	Comment	C13CPD
4	Origin	Bruker BioSpin GmbH
5	Owner	common
6	Site	
7	Spectrometer	spect
8	Author	
9	Solvent	CDCl3
10	Temperature	-1952.6
11	Pulse Sequence	zgpg30
12	Experiment	1D
13	Number of Scans	119
14	Receiver Gain	203
15	Relaxation Delay	1.0000
16	Pulse Width	12.0000
17	Acquisition Time	0.4588
18	Acquisition Date	2016-07-29T22:36:00
19	Modification Date	2016-07-29T22:36:00
20	Spectrometer Frequency	100.61
21	Spectral Width	35714.3
22	Lowest Frequency	-7795.9
23	Nucleus	13C
24	Acquired Size	16384
25	Spectral Size	32768

Supplementary Figure 27 | ^{13}C NMR (101 MHz, $CDCl_3$) spectra for compound 8e.

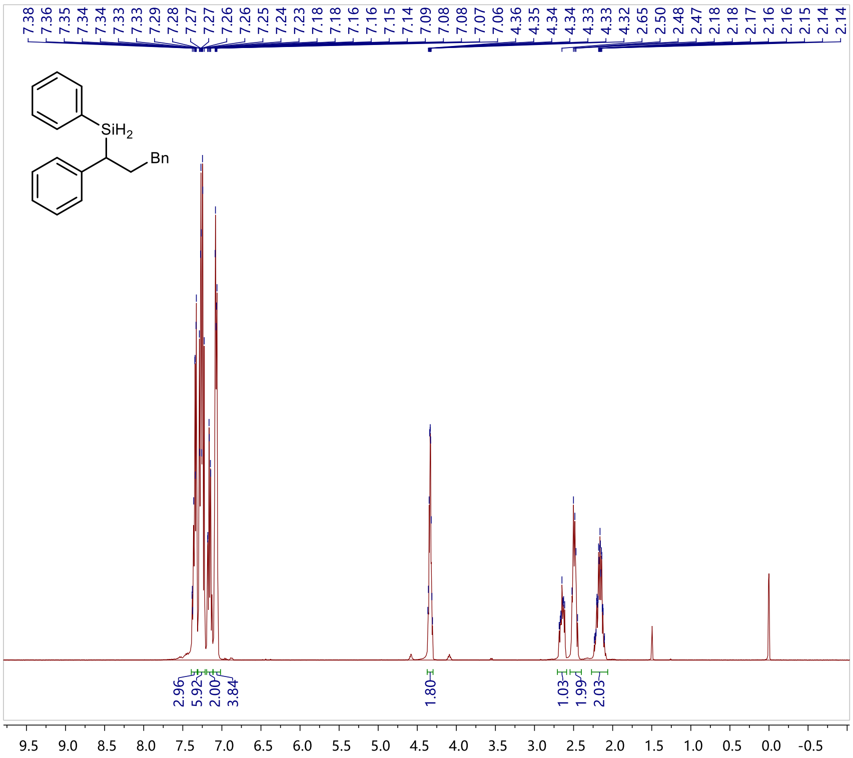

Parameter		Value
1	Data File Name	C:/ Users/ yuanyuan/ Desktop/ 新建文件夹 (3)/ 产物nmr/ f (β-Bn)/ 1/ fid
2	Title	f (β-Bn)
3	Comment	PROTON
4	Origin	Bruker BioSpin GmbH
5	Owner	nmr
6	Site	
7	Spectrometer	spect
8	Author	
9	Solvent	CDCl3
10	Temperature	294.4
11	Pulse Sequence	zg30
12	Experiment	1D
13	Number of Scans	8
14	Receiver Gain	87
15	Relaxation Delay	1.0000
16	Pulse Width	15.0000
17	Acquisition Time	2.4999
18	Acquisition Date	2016-12-09T01:02:48
19	Modification Date	2016-12-09T01:02:00
20	Spectrometer Frequency	400.13
21	Spectral Width	8012.8
22	Lowest Frequency	-1562.0
23	Nucleus	1H
24	Acquired Size	20031
25	Spectral Size	65536

Supplementary Figure 28 | ^{1}H NMR (400 MHz, $CDCl_3$) spectra for compound 8f.

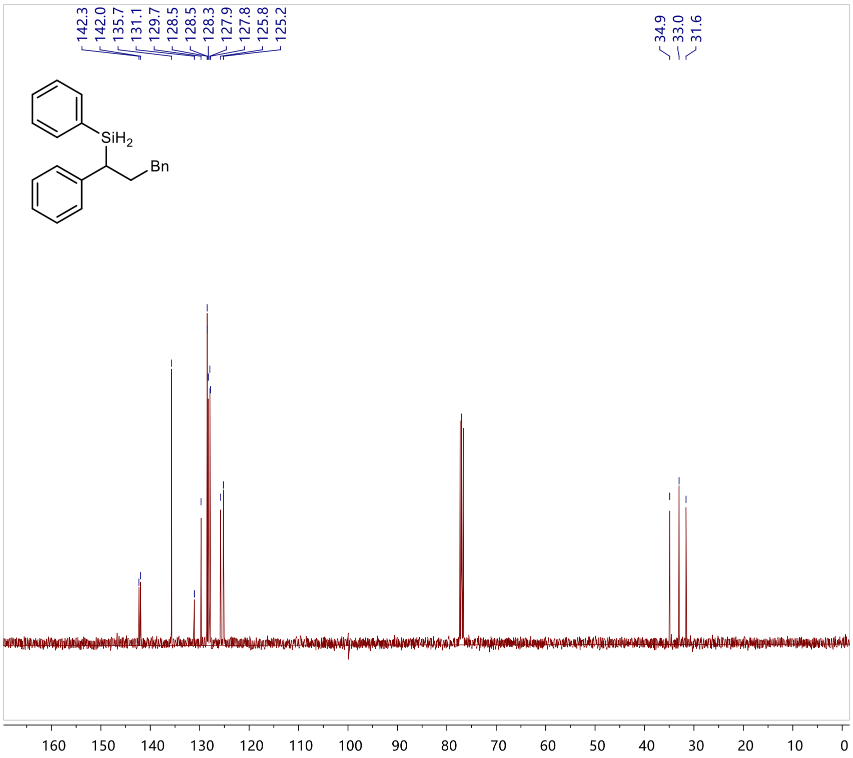

Parameter		Value
1	Data File Name	C:/ Users/ yuanyuan/ Desktop/ 新建文件夹 (3)/ 产物nmr/ f (β-Bn)/ 2/ fid
2	Title	f (β-Bn)
3	Comment	C13CPD
4	Origin	Bruker BioSpin GmbH
5	Owner	nmr
6	Site	
7	Spectrometer	spect
8	Author	
9	Solvent	CDCl3
10	Temperature	295.0
11	Pulse Sequence	zgpg30
12	Experiment	1D
13	Number of Scans	117
14	Receiver Gain	77
15	Relaxation Delay	1.0000
16	Pulse Width	9.8000
17	Acquisition Time	0.4588
18	Acquisition Date	2016-12-09T01:05:04
19	Modification Date	2016-12-09T01:06:00
20	Spectrometer Frequency	100.61
21	Spectral Width	35714.3
22	Lowest Frequency	-7796.8
23	Nucleus	13C
24	Acquired Size	16384
25	Spectral Size	32768

Supplementary Figure 29 | ^{13}C NMR (101 MHz, $CDCl_3$) spectra for compound 8f.

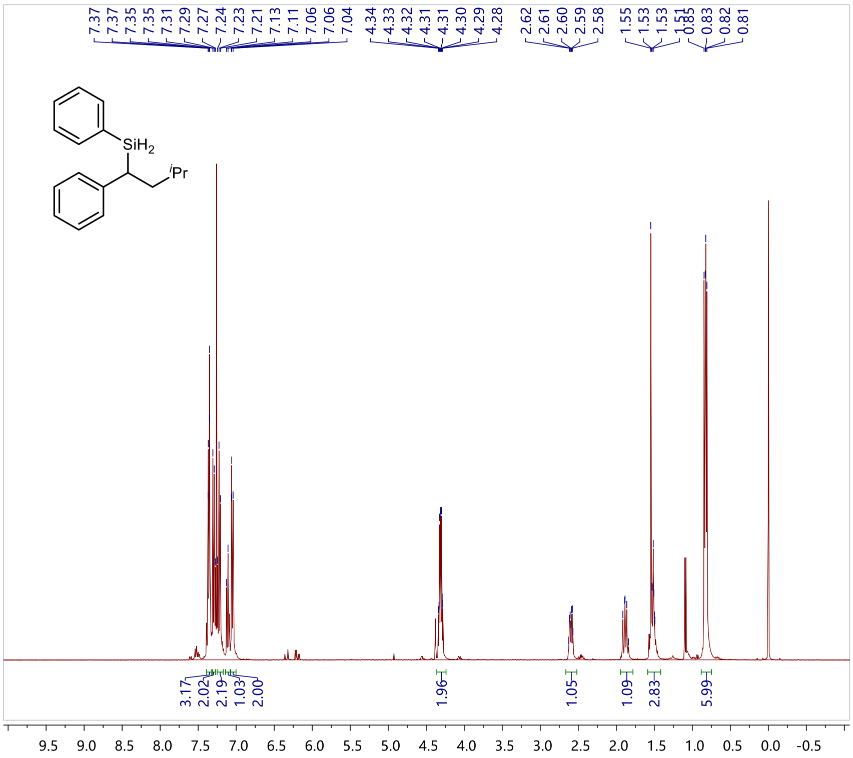

Parameter	Value
1 Data File Name	C:/ Users/ yuanyuan/ Desktop/ 新建文件夹 (3)/ 产物nmr/ h (β-iPr)/ 1/ fid
2 Title	h (β-iPr)
3 Comment	
4 Origin	Bruker BioSpin GmbH
5 Owner	nmr
6 Site	
7 Spectrometer	spect
8 Author	
9 Solvent	CDCl3
10 Temperature	293.0
11 Pulse Sequence	zg30
12 Experiment	1D
13 Number of Scans	8
14 Receiver Gain	81
15 Relaxation Delay	1.0000
16 Pulse Width	9.8000
17 Acquisition Time	4.0894
18 Acquisition Date	2016-07-30T00:54:17
19 Modification Date	2016-07-30T00:54:00
20 Spectrometer Frequency	400.13
21 Spectral Width	8012.8
22 Lowest Frequency	-1535.4
23 Nucleus	1H
24 Acquired Size	32768
25 Spectral Size	65536

Supplementary Figure 30 | ^{1}H NMR (400 MHz, $CDCl_3$) spectra for compound 8g.

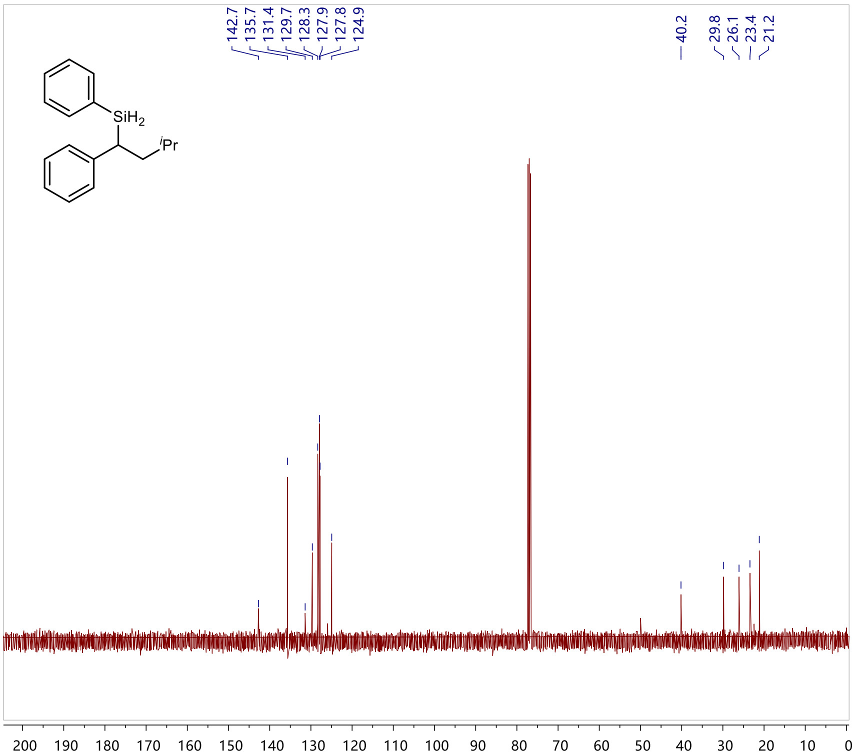

Parameter	Value
1 Data File Name	C:/ Users/ yuanyuan/ Desktop/ 新建文件夹 (3)/ 产物nmr/ h (β-iPr)/ 2/ fid
2 Title	h (β-iPr)
3 Comment	13CPD
4 Origin	Bruker BioSpin GmbH
5 Owner	nmr
6 Site	
7 Spectrometer	spect
8 Author	
9 Solvent	CDCl3
10 Temperature	293.5
11 Pulse Sequence	zgpg30
12 Experiment	1D
13 Number of Scans	371
14 Receiver Gain	205
15 Relaxation Delay	1.0000
16 Pulse Width	9.3000
17 Acquisition Time	0.8039
18 Acquisition Date	2016-07-30T01:00:37
19 Modification Date	2016-07-30T01:06:00
20 Spectrometer Frequency	100.61
21 Spectral Width	40760.9
22 Lowest Frequency	-15349.8
23 Nucleus	13C
24 Acquired Size	32768
25 Spectral Size	65536

Supplementary Figure 31 | ^{13}C NMR (101 MHz, $CDCl_3$) spectra for compound 8g.

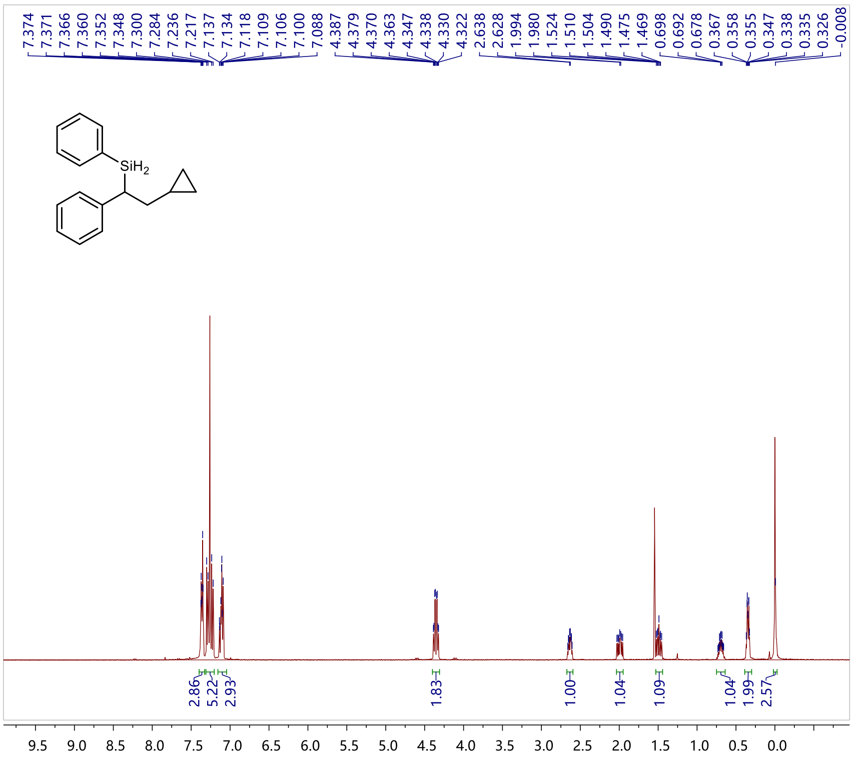

	Parameter	Value
1	Data File Name	Z:/ Data/ NMR/ 2017-01/ hmy-hbw-1/ 1/ fid
2	Title	hmy-hbw-1
3	Comment	test
4	Origin	Bruker BioSpin GmbH
5	Owner	nmr
6	Site	
7	Spectrometer	spect
8	Author	
9	Solvent	CDCl3
10	Temperature	292.9
11	Pulse Sequence	zg30
12	Experiment	1D
13	Number of Scans	8
14	Receiver Gain	194
15	Relaxation Delay	1.0000
16	Pulse Width	9.8000
17	Acquisition Time	1.9999
18	Acquisition Date	2017-01-25T06:39:27
19	Modification Date	2017-01-25T06:39:00
20	Spectrometer Frequency	400.13
21	Spectral Width	8012.8
22	Lowest Frequency	-1535.4
23	Nucleus	1H
24	Acquired Size	16025
25	Spectral Size	32768

Supplementary Figure 32 | ^{1}H NMR (400 MHz, $CDCl_3$) spectra for compound 8h.

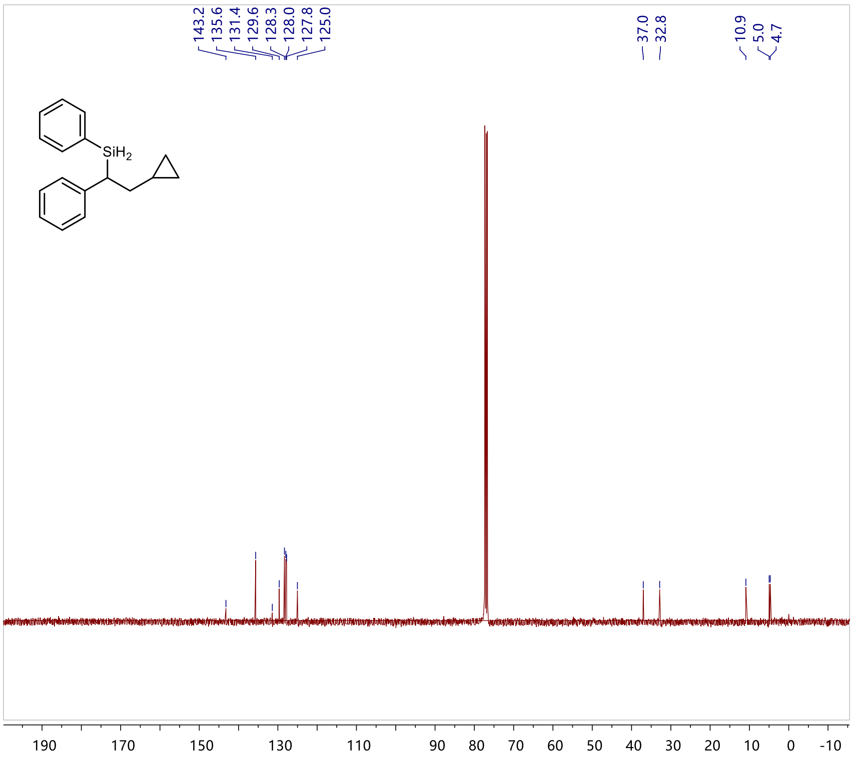

	Parameter	Value
1	Data File Name	Z:/ Data/ NMR/ 2017-01/ hmy-hbw-1/ 2/ fid
2	Title	hmy-hbw-1
3	Comment	test
4	Origin	Bruker BioSpin GmbH
5	Owner	nmr
6	Site	
7	Spectrometer	spect
8	Author	
9	Solvent	CDCl3
10	Temperature	294.2
11	Pulse Sequence	zgpg30
12	Experiment	1D
13	Number of Scans	1024
14	Receiver Gain	78
15	Relaxation Delay	1.0000
16	Pulse Width	9.8000
17	Acquisition Time	0.8039
18	Acquisition Date	2017-01-25T07:12:22
19	Modification Date	2017-01-25T07:12:00
20	Spectrometer Frequency	100.61
21	Spectral Width	40760.9
22	Lowest Frequency	-10320.1
23	Nucleus	13C
24	Acquired Size	32768
25	Spectral Size	65536

Supplementary Figure 33 | ^{13}C NMR (101 MHz, $CDCl_3$) spectra for compound 8h.

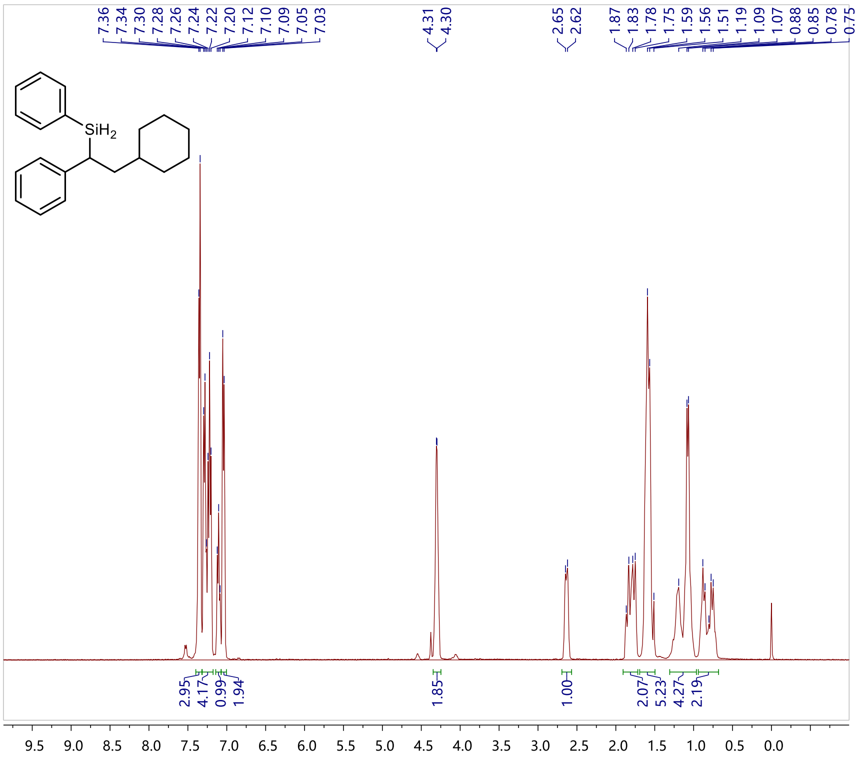

Parameter	Value
1 Data File Name	Z:/ Data/ NMR/ 2017-01/ hmy-20170109-3-5i/ 1/ fid
2 Title	hmy-20170109-3-5i
3 Comment	PROTON
4 Origin	Bruker BioSpin GmbH
5 Owner	common
6 Site	
7 Spectrometer	spect
8 Author	
9 Solvent	CDCl3
10 Temperature	297.6
11 Pulse Sequence	zg30
12 Experiment	1D
13 Number of Scans	8
14 Receiver Gain	90
15 Relaxation Delay	1.0000
16 Pulse Width	13.7000
17 Acquisition Time	1.9999
18 Acquisition Date	2017-01-09T06:46:00
19 Modification Date	2017-01-09T06:46:00
20 Spectrometer Frequency	400.13
21 Spectral Width	8223.7
22 Lowest Frequency	-1657.0
23 Nucleus	1H
24 Acquired Size	16446
25 Spectral Size	65536

Supplementary Figure 34 | ^{1}H NMR (400 MHz, $CDCl_3$) spectra for compound 8i.

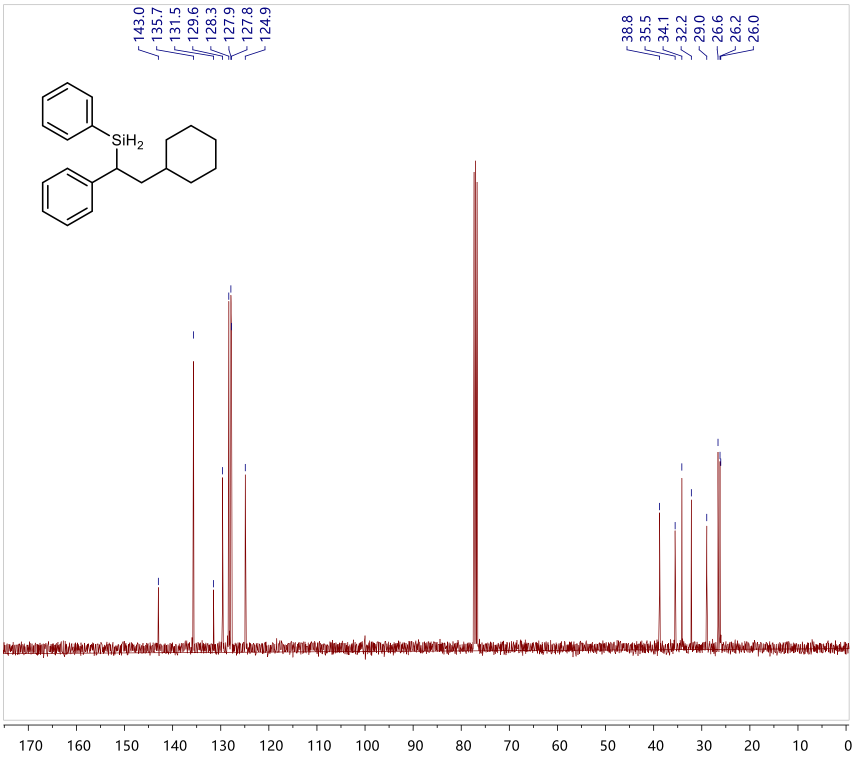

Parameter	Value
1 Data File Name	Z:/ Data/ NMR/ 2017-01/ hmy-20170109-3-5i/ 2/ fid
2 Title	hmy-20170109-3-5i
3 Comment	C13CPD
4 Origin	Bruker BioSpin GmbH
5 Owner	common
6 Site	
7 Spectrometer	spect
8 Author	
9 Solvent	CDCl3
10 Temperature	298.9
11 Pulse Sequence	zgpg30
12 Experiment	1D
13 Number of Scans	427
14 Receiver Gain	203
15 Relaxation Delay	1.0000
16 Pulse Width	12.0000
17 Acquisition Time	0.4588
18 Acquisition Date	2017-01-09T06:58:00
19 Modification Date	2017-01-09T06:59:00
20 Spectrometer Frequency	100.61
21 Spectral Width	35714.3
22 Lowest Frequency	-7795.9
23 Nucleus	13C
24 Acquired Size	16384
25 Spectral Size	32768

Supplementary Figure 35 | ^{13}C NMR (101 MHz, $CDCl_3$) spectra for compound 8i.

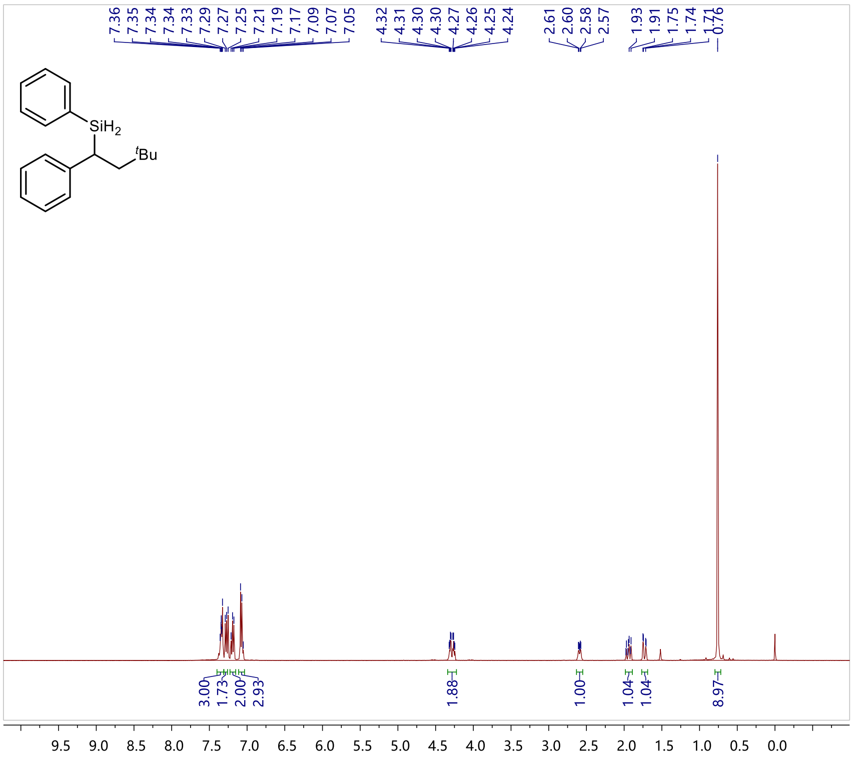

	Parameter	Value
1	Data File Name	Z:/ Data/ NMR/ 2017-01/ hmy-20170101-2/ 1/ fid
2	Title	hmy-20170101-2
3	Comment	PROTON
4	Origin	Bruker BioSpin GmbH
5	Owner	common
6	Site	
7	Spectrometer	spect
8	Author	
9	Solvent	CDCl3
10	Temperature	300.1
11	Pulse Sequence	zg30
12	Experiment	1D
13	Number of Scans	8
14	Receiver Gain	203
15	Relaxation Delay	1.0000
16	Pulse Width	13.7000
17	Acquisition Time	1.9999
18	Acquisition Date	2017-01-02T06:43:00
19	Modification Date	2017-01-02T06:43:00
20	Spectrometer Frequency	400.13
21	Spectral Width	8223.7
22	Lowest Frequency	-1640.9
23	Nucleus	1H
24	Acquired Size	16446
25	Spectral Size	65536

Supplementary Figure 36 | ^{1}H NMR (400 MHz, $CDCl_3$) spectra for compound 8j.

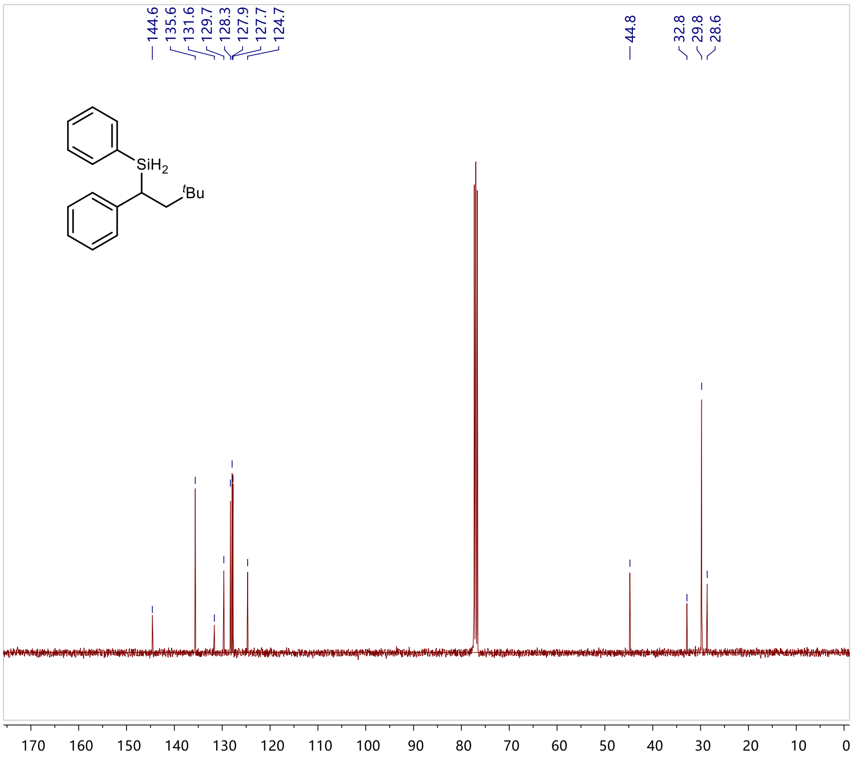

	Parameter	Value
1	Data File Name	Z:/ Data/ NMR/ 2017-01/ hmy-20170101-2/ 2/ fid
2	Title	hmy-20170101-2
3	Comment	C13CPD
4	Origin	Bruker BioSpin GmbH
5	Owner	common
6	Site	
7	Spectrometer	spect
8	Author	
9	Solvent	CDCl3
10	Temperature	300.9
11	Pulse Sequence	zgpg30
12	Experiment	1D
13	Number of Scans	1024
14	Receiver Gain	203
15	Relaxation Delay	1.0000
16	Pulse Width	12.0000
17	Acquisition Time	0.4588
18	Acquisition Date	2017-01-02T07:10:00
19	Modification Date	2017-01-02T07:10:00
20	Spectrometer Frequency	100.61
21	Spectral Width	35714.3
22	Lowest Frequency	-7725.4
23	Nucleus	13C
24	Acquired Size	16384
25	Spectral Size	32768

Supplementary Figure 37 | ^{13}C NMR (101 MHz, $CDCl_3$) spectra for compound 8j.

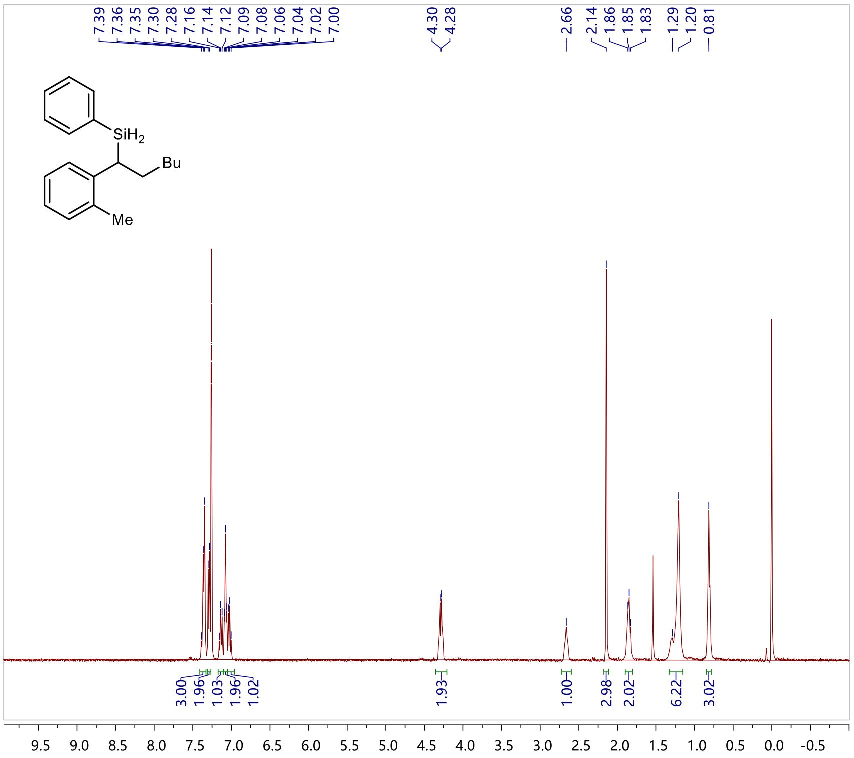

	Parameter	Value
1	Data File Name	C:/ Users/ yuanyuan/ Desktop/ 0000/ hmy/ hmy-2Me-2/ 1/ fid
2	Title	hmy-2Me-2
3	Comment	PROTON
4	Origin	Bruker BioSpin GmbH
5	Owner	common
6	Site	
7	Spectrometer	spect
8	Author	
9	Solvent	CDCl3
10	Temperature	296.1
11	Pulse Sequence	zg30
12	Experiment	1D
13	Number of Scans	8
14	Receiver Gain	203
15	Relaxation Delay	1.0000
16	Pulse Width	13.7000
17	Acquisition Time	1.9999
18	Acquisition Date	2017-01-24T10:15:00
19	Modification Date	2017-01-24T18:15:00
20	Spectrometer Frequency	400.13
21	Spectral Width	8223.7
22	Lowest Frequency	-1640.9
23	Nucleus	1H
24	Acquired Size	16446
25	Spectral Size	65536

Supplementary Figure 38 | ^{1}H NMR (400 MHz, $CDCl_3$) spectra for compound 8k.

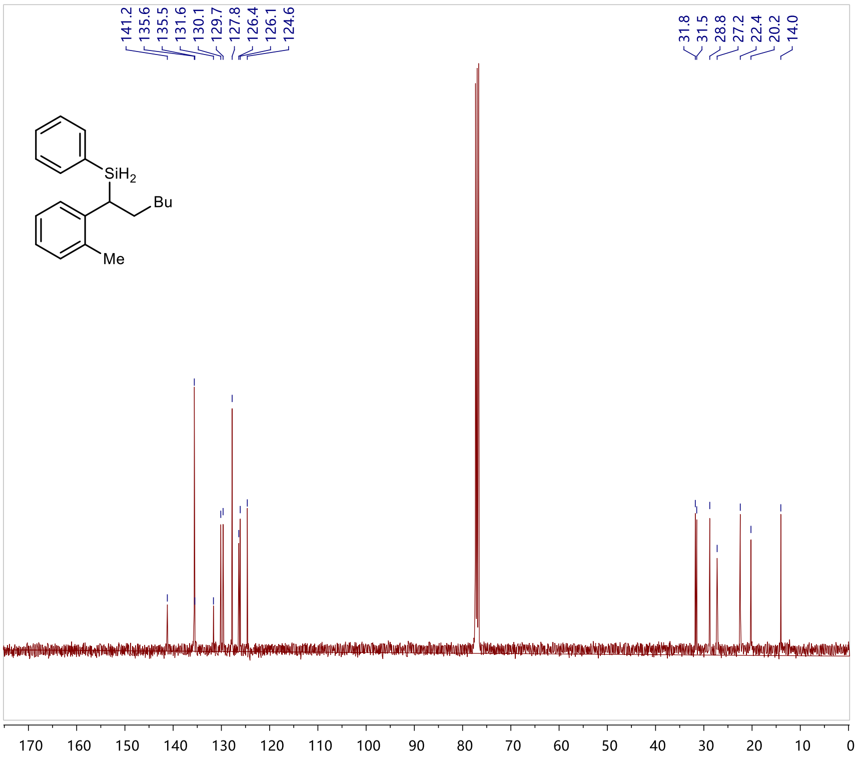

	Parameter	Value
1	Data File Name	C:/ Users/ yuanyuan/ Desktop/ hmy-2Me-1/ 2/ fid
2	Title	hmy-2Me-1
3	Comment	C13CPD
4	Origin	Bruker BioSpin GmbH
5	Owner	common
6	Site	
7	Spectrometer	spect
8	Author	
9	Solvent	CDCl3
10	Temperature	297.9
11	Pulse Sequence	zgpg30
12	Experiment	1D
13	Number of Scans	1718
14	Receiver Gain	203
15	Relaxation Delay	1.0000
16	Pulse Width	12.0000
17	Acquisition Time	0.4588
18	Acquisition Date	2017-01-21T11:25:00
19	Modification Date	2017-01-21T11:25:00
20	Spectrometer Frequency	100.61
21	Spectral Width	35714.3
22	Lowest Frequency	-7795.9
23	Nucleus	13C
24	Acquired Size	16384
25	Spectral Size	32768

Supplementary Figure 39 | ^{13}C NMR (101 MHz, $CDCl_3$) spectra for compound 8k.

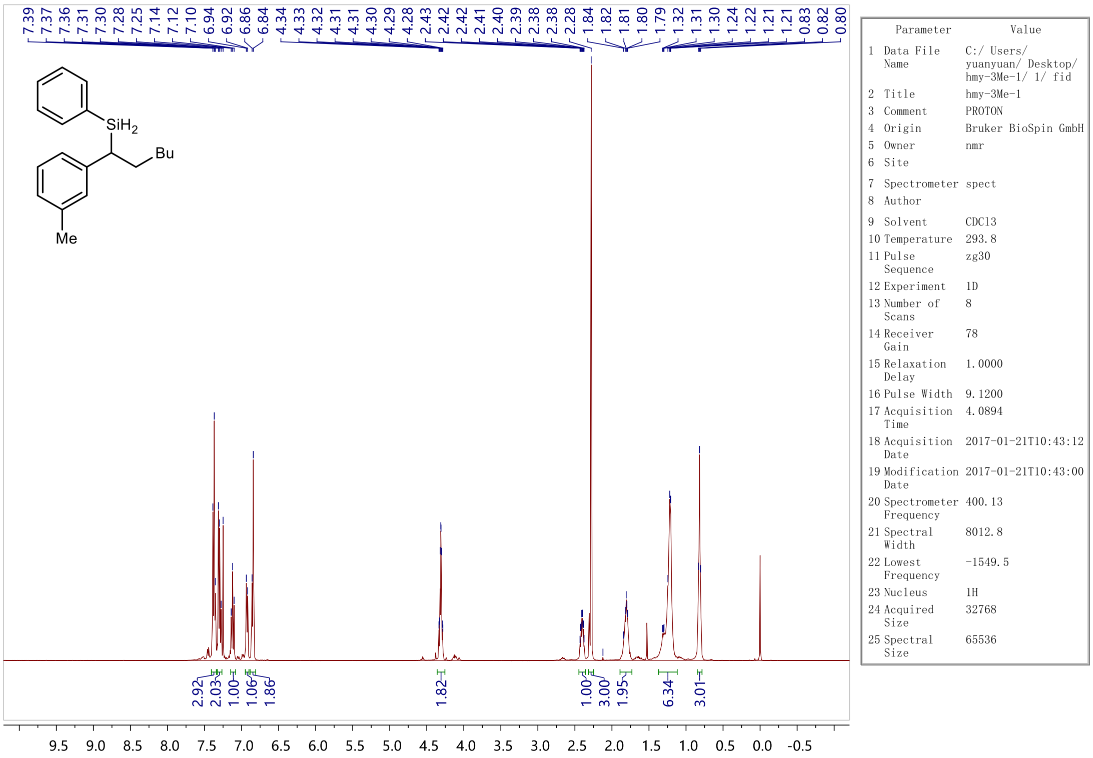

Supplementary Figure 40 | ^{1}H NMR (400 MHz, CDCl$_3$) spectra for compound 8l.

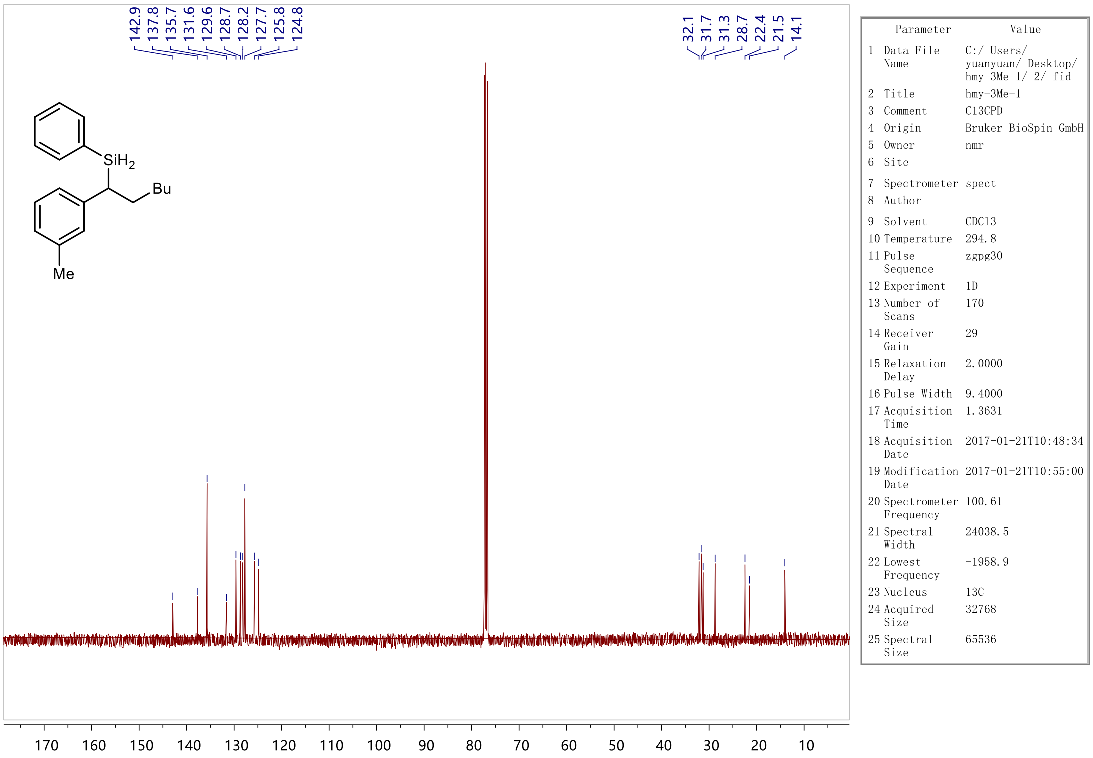

Supplementary Figure 41 | ^{13}C NMR (101 MHz, CDCl$_3$) spectra for compound 8l.

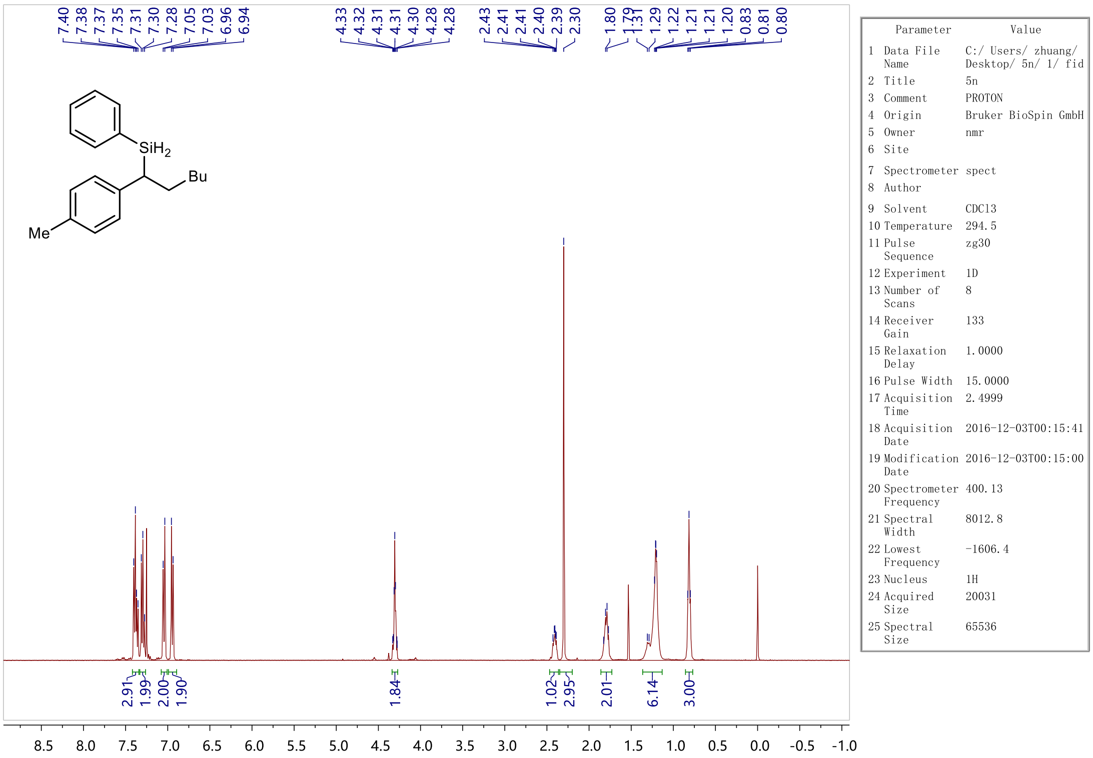

Supplementary Figure 42 | ^{1}H NMR (400 MHz, $CDCl_3$) spectra for compound 8m.

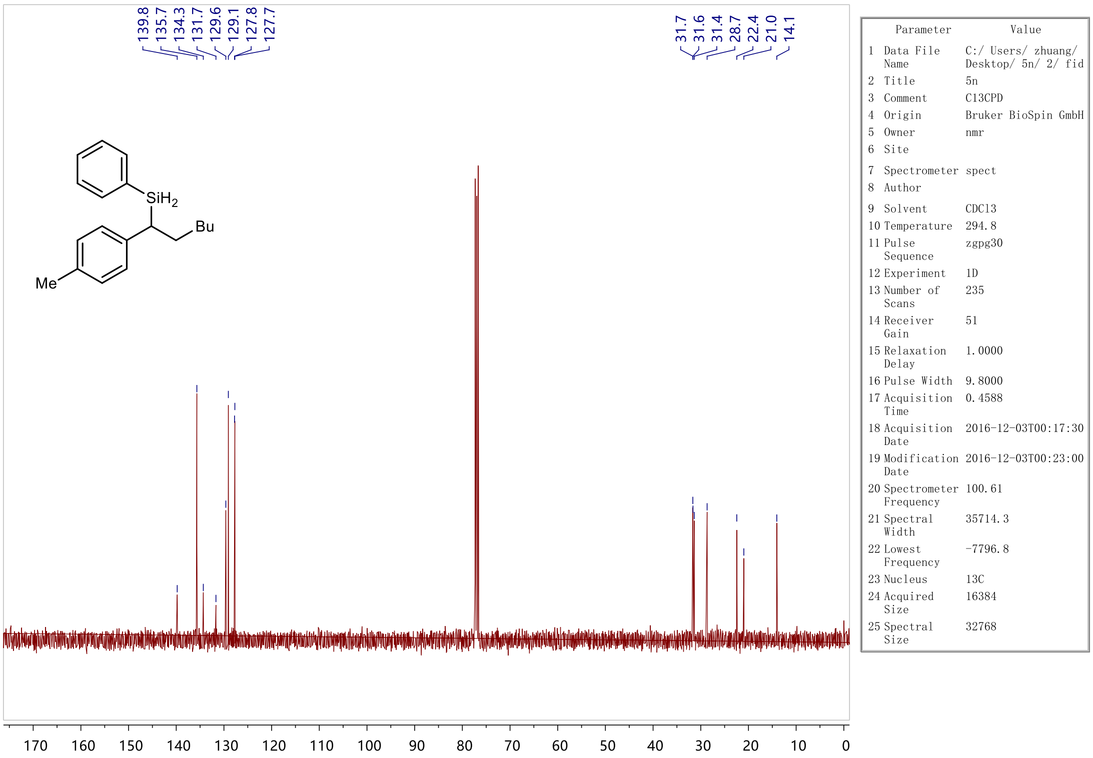

Supplementary Figure 43 | ^{13}C NMR (101 MHz, $CDCl_3$) spectra for compound 8m.

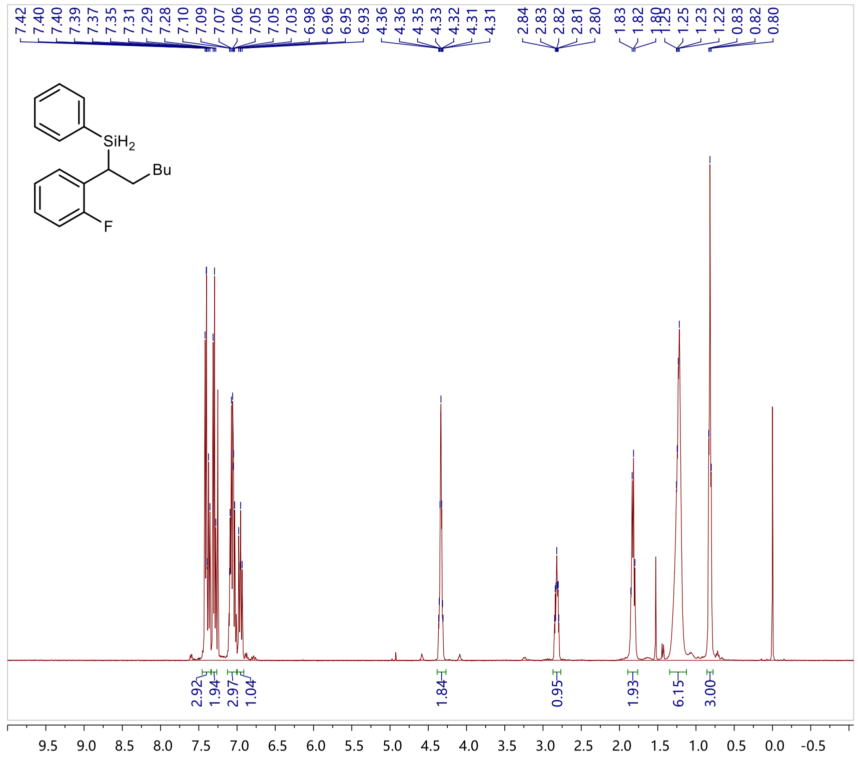

Parameter	Value
1 Data File Name	C:/ Users/ zhuang/ Desktop/ 5o/ 1/ fid
2 Title	5o
3 Comment	PROTON
4 Origin	Bruker BioSpin GmbH
5 Owner	nmr
6 Site	
7 Spectrometer	spect
8 Author	
9 Solvent	CDCl3
10 Temperature	0.0
11 Pulse Sequence	zg30
12 Experiment	1D
13 Number of Scans	8
14 Receiver Gain	71
15 Relaxation Delay	1.0000
16 Pulse Width	9.1200
17 Acquisition Time	4.0894
18 Acquisition Date	2016-12-28T06:26:46
19 Modification Date	2016-12-28T06:27:00
20 Spectrometer Frequency	400.13
21 Spectral Width	8012.8
22 Lowest Frequency	-1549.1
23 Nucleus	1H
24 Acquired Size	32768
25 Spectral Size	65536

Supplementary Figure 44 | ^{1}H NMR (400 MHz, $CDCl_3$) spectra for compound 8n.

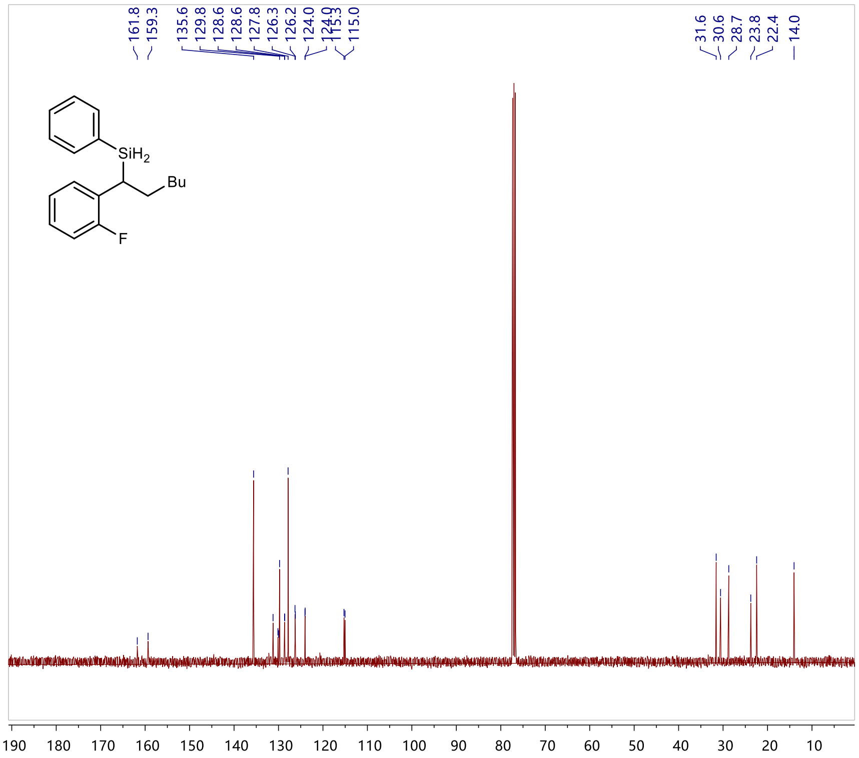

Parameter	Value
1 Data File Name	C:/ Users/ zhuang/ Desktop/ 5o/ 2/ fid
2 Title	5o
3 Comment	C13CPD
4 Origin	Bruker BioSpin GmbH
5 Owner	nmr
6 Site	
7 Spectrometer	spect
8 Author	
9 Solvent	CDCl3
10 Temperature	0.0
11 Pulse Sequence	zgpg30
12 Experiment	1D
13 Number of Scans	250
14 Receiver Gain	29
15 Relaxation Delay	2.0000
16 Pulse Width	9.4000
17 Acquisition Time	1.3631
18 Acquisition Date	2016-12-28T06:42:14
19 Modification Date	2016-12-28T06:42:00
20 Spectrometer Frequency	100.61
21 Spectral Width	24038.5
22 Lowest Frequency	-1958.9
23 Nucleus	13C
24 Acquired Size	32768
25 Spectral Size	65536

Supplementary Figure 45 | ^{13}C NMR (101 MHz, $CDCl_3$) spectra for compound 8n.

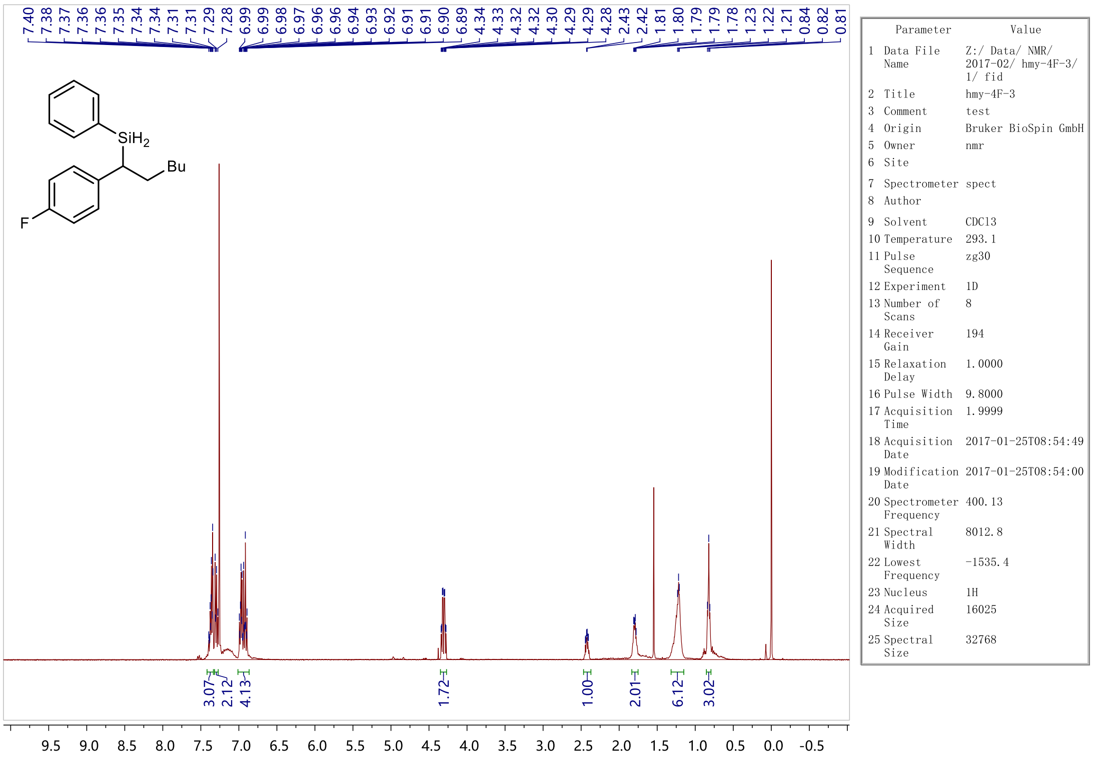

Supplementary Figure 46 | ^{1}H NMR (400 MHz, $CDCl_3$) spectra for compound 8o.

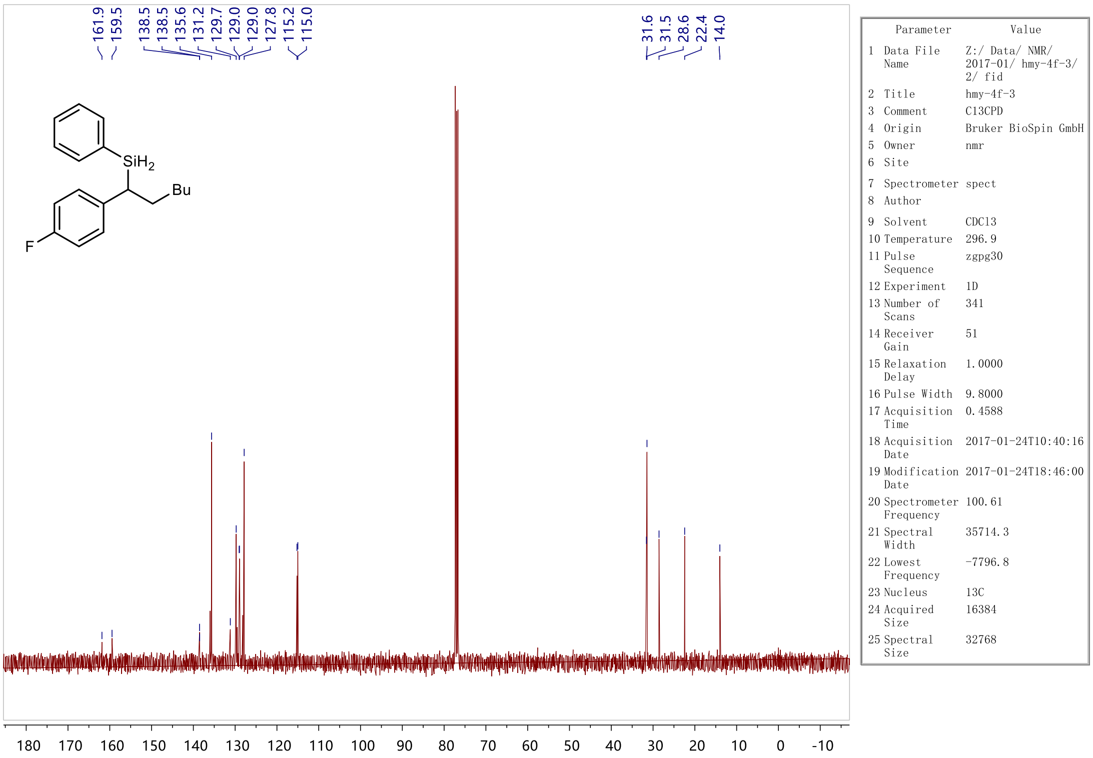

Supplementary Figure 47 | ^{13}C NMR (101 MHz, $CDCl_3$) spectra for compound 8o.

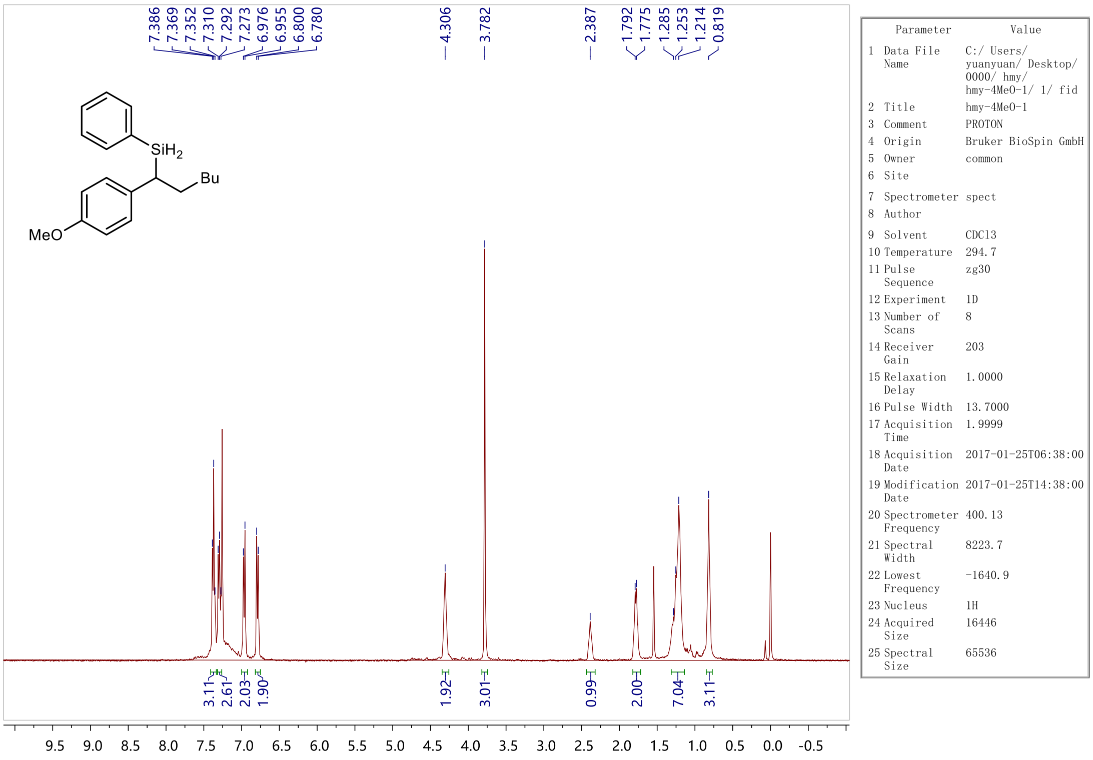

Supplementary Figure 48 | ^{1}H NMR (400 MHz, $CDCl_3$) spectra for compound 8p.

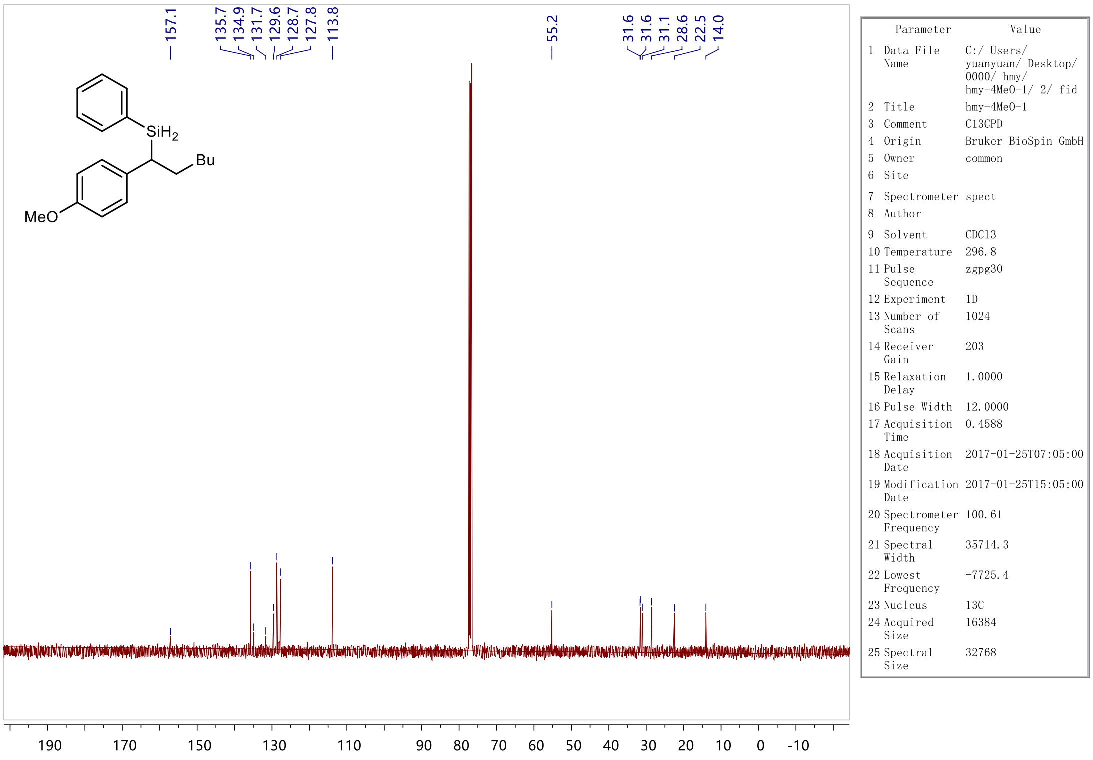

Supplementary Figure 49 | ^{13}C NMR (101 MHz, $CDCl_3$) spectra for compound 8p.

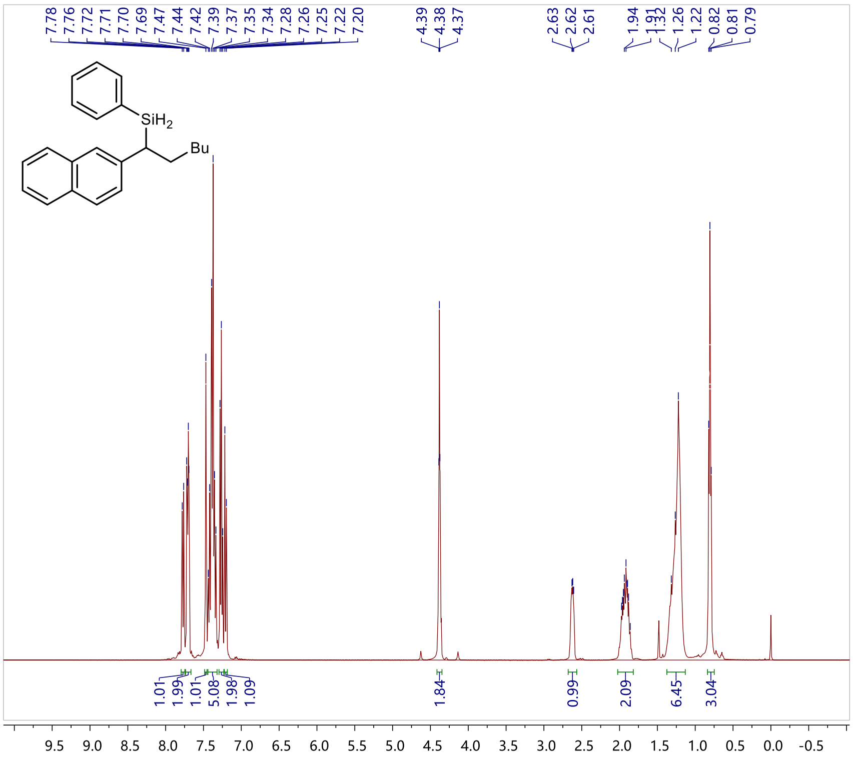

	Parameter	Value
1	Data File Name	Z:/ Data/ NMR/ 2017-01/ hmy-20170117-5Na/ 1/ fid
2	Title	hmy-20170117-5Na
3	Comment	PROTON
4	Origin	Bruker BioSpin GmbH
5	Owner	nmr
6	Site	
7	Spectrometer	spect
8	Author	
9	Solvent	CDCl3
10	Temperature	297.4
11	Pulse Sequence	zg30
12	Experiment	1D
13	Number of Scans	8
14	Receiver Gain	71
15	Relaxation Delay	1.0000
16	Pulse Width	15.0000
17	Acquisition Time	2.4999
18	Acquisition Date	2017-01-17T11:24:23
19	Modification Date	2017-01-17T11:24:00
20	Spectrometer Frequency	400.13
21	Spectral Width	8012.8
22	Lowest Frequency	-1615.7
23	Nucleus	1H
24	Acquired Size	20031
25	Spectral Size	65536

Supplementary Figure 50 | ^{1}H NMR (400 MHz, $CDCl_3$) spectra for compound 8q.

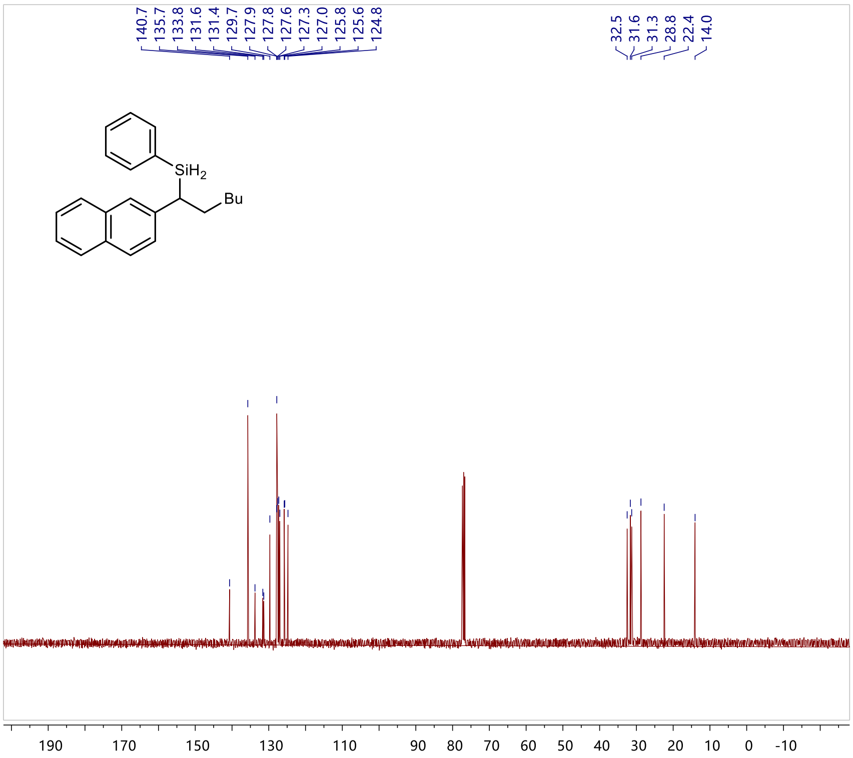

	Parameter	Value
1	Data File Name	Z:/ Data/ NMR/ 2017-01/ hmy-20170117-5Na/ 2/ fid
2	Title	hmy-20170117-5Na
3	Comment	C13CPD
4	Origin	Bruker BioSpin GmbH
5	Owner	nmr
6	Site	
7	Spectrometer	spect
8	Author	
9	Solvent	CDCl3
10	Temperature	298.0
11	Pulse Sequence	zgpg30
12	Experiment	1D
13	Number of Scans	112
14	Receiver Gain	98
15	Relaxation Delay	1.0000
16	Pulse Width	9.8000
17	Acquisition Time	0.4588
18	Acquisition Date	2017-01-17T11:27:20
19	Modification Date	2017-01-17T11:28:00
20	Spectrometer Frequency	100.61
21	Spectral Width	35714.3
22	Lowest Frequency	-7796.8
23	Nucleus	13C
24	Acquired Size	16384
25	Spectral Size	32768

Supplementary Figure 51 | ^{13}C NMR (101 MHz, $CDCl_3$) spectra for compound 8q.

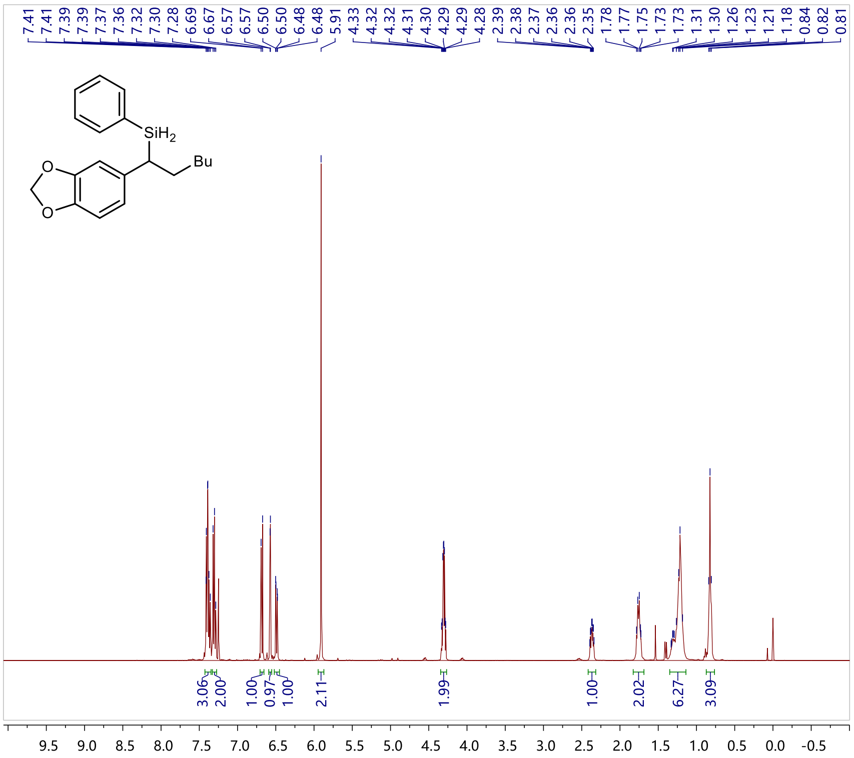

Parameter	Value
1 Data File Name	Z:/ Data/ NMR/ 2017-01/ hmy-20170109-2-5u-1/ 1/ fid
2 Title	hmy-20170109-2-5u-1
3 Comment	PROTON
4 Origin	Bruker BioSpin GmbH
5 Owner	nmr
6 Site	
7 Spectrometer	spect
8 Author	
9 Solvent	CDCl3
10 Temperature	294.0
11 Pulse Sequence	zg30
12 Experiment	1D
13 Number of Scans	8
14 Receiver Gain	64
15 Relaxation Delay	1.0000
16 Pulse Width	9.1200
17 Acquisition Time	4.0894
18 Acquisition Date	2017-01-09T06:29:27
19 Modification Date	2017-01-09T06:29:00
20 Spectrometer Frequency	400.13
21 Spectral Width	8012.8
22 Lowest Frequency	-1550.0
23 Nucleus	1H
24 Acquired Size	32768
25 Spectral Size	65536

Supplementary Figure 52 | ^{1}H NMR (400 MHz, $CDCl_3$) spectra for compound 8r.

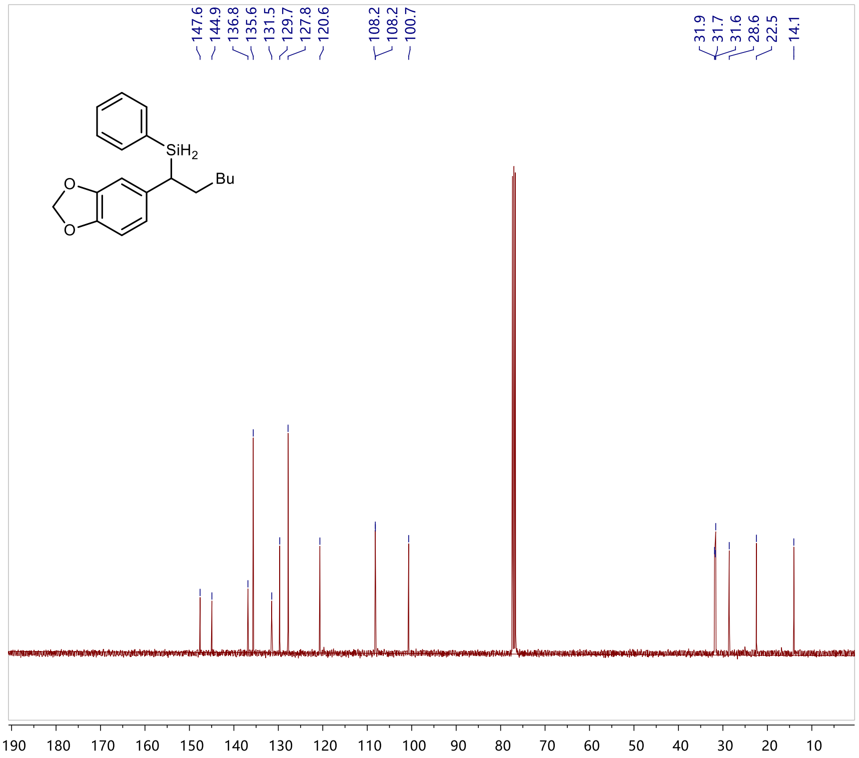

Parameter	Value
1 Data File Name	Z:/ Data/ NMR/ 2017-01/ hmy-20170109-2-5u-1/ 2/ fid
2 Title	hmy-20170109-2-5u-1
3 Comment	C13CPD
4 Origin	Bruker BioSpin GmbH
5 Owner	nmr
6 Site	
7 Spectrometer	spect
8 Author	
9 Solvent	CDCl3
10 Temperature	294.8
11 Pulse Sequence	zgpg30
12 Experiment	1D
13 Number of Scans	347
14 Receiver Gain	29
15 Relaxation Delay	2.0000
16 Pulse Width	9.4000
17 Acquisition Time	1.3631
18 Acquisition Date	2017-01-09T06:32:45
19 Modification Date	2017-01-09T06:50:00
20 Spectrometer Frequency	100.61
21 Spectral Width	24038.5
22 Lowest Frequency	-1958.9
23 Nucleus	13C
24 Acquired Size	32768
25 Spectral Size	65536

Supplementary Figure 53 | ^{13}C NMR (101 MHz, $CDCl_3$) spectra for compound 8r.

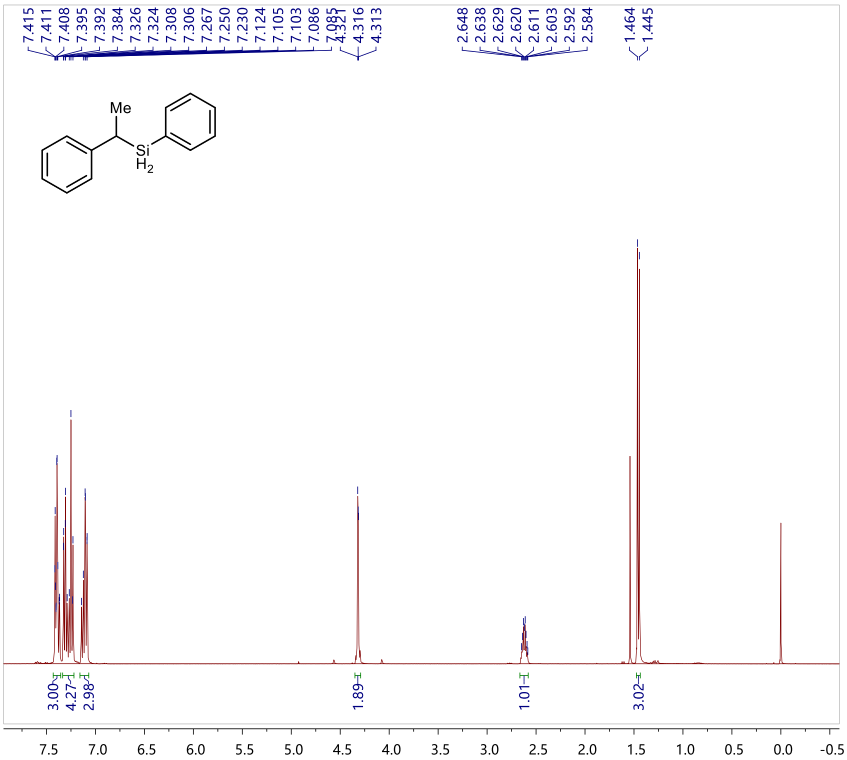

	Parameter	Value
1	Data File Name	I:/ 数据整理/ 数据/产物NMR/ 苯乙烯/ 1/fid
2	Title	苯乙烯
3	Comment	
4	Origin	Bruker BioSpin GmbH
5	Owner	nmr
6	Site	
7	Spectrometer	spect
8	Author	
9	Solvent	CDCl3
10	Temperature	293.5
11	Pulse Sequence	zg30
12	Experiment	1D
13	Number of Scans	8
14	Receiver Gain	117
15	Relaxation Delay	1.0000
16	Pulse Width	9.8000
17	Acquisition Time	1.9999
18	Acquisition Date	2015-11-26T23:36:29
19	Modification Date	2015-11-26T23:36:00
20	Spectrometer Frequency	400.13
21	Spectral Width	8012.8
22	Lowest Frequency	-2125.8
23	Nucleus	1H
24	Acquired Size	16025
25	Spectral Size	32768

Supplementary Figure 54 | ^{1}H NMR (400 MHz, $CDCl_3$) spectra for compound 10a.

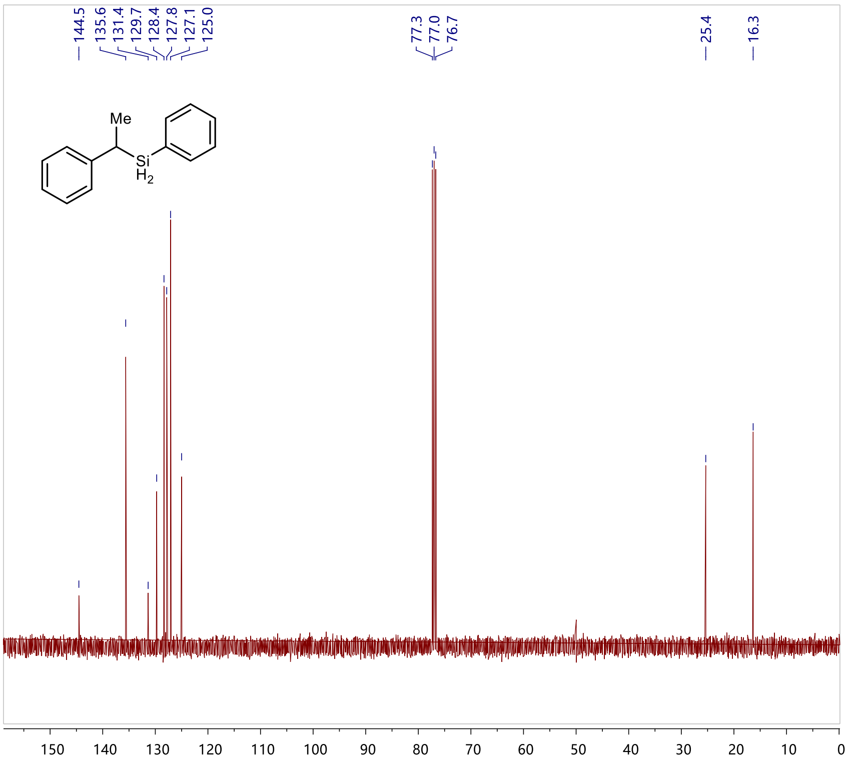

	Parameter	Value
1	Data File Name	I:/ 数据整理/ 数据/产物NMR/ 苯乙烯/ 2/fid
2	Title	苯乙烯
3	Comment	13CPD
4	Origin	Bruker BioSpin GmbH
5	Owner	nmr
6	Site	
7	Spectrometer	spect
8	Author	
9	Solvent	CDCl3
10	Temperature	293.7
11	Pulse Sequence	zgpg30
12	Experiment	1D
13	Number of Scans	222
14	Receiver Gain	205
15	Relaxation Delay	1.0000
16	Pulse Width	9.3000
17	Acquisition Time	0.8039
18	Acquisition Date	2015-11-26T23:41:30
19	Modification Date	2015-11-26T23:45:00
20	Spectrometer Frequency	100.61
21	Spectral Width	40760.9
22	Lowest Frequency	-15349.8
23	Nucleus	13C
24	Acquired Size	32768
25	Spectral Size	65536

Supplementary Figure 55 | ^{13}C NMR (101 MHz, $CDCl_3$) spectra for compound 10a.

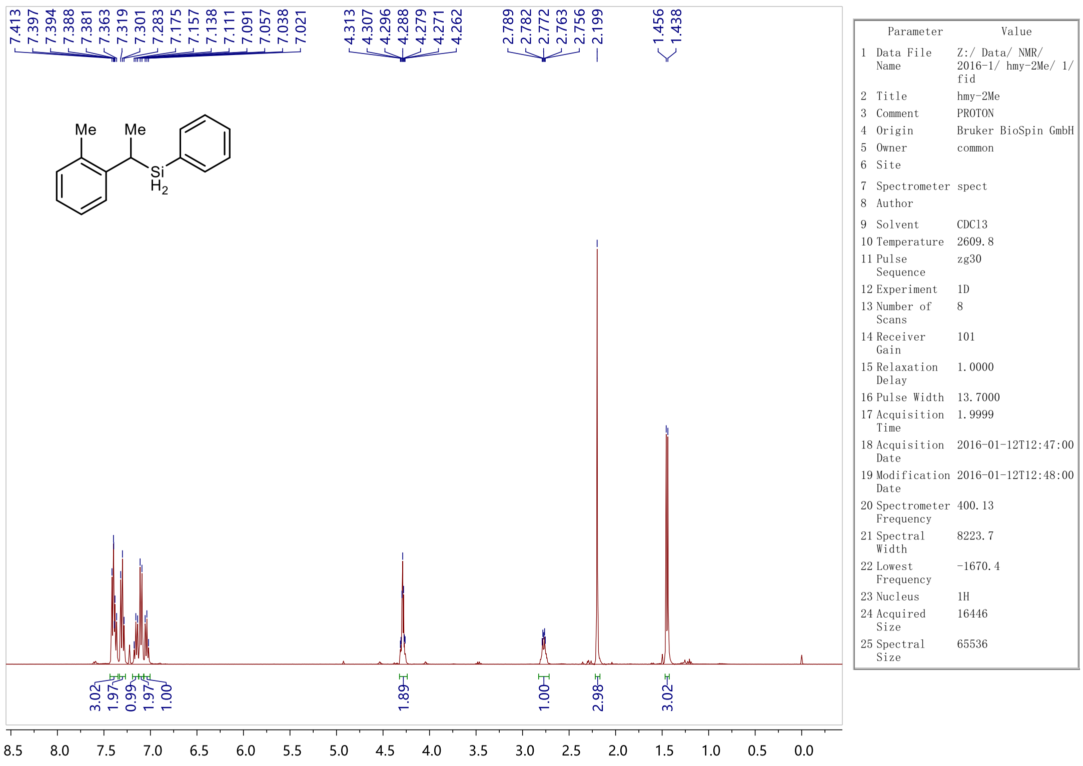

Supplementary Figure 56 | ^{1}H NMR (400 MHz, $CDCl_3$) spectra for compound 10b.

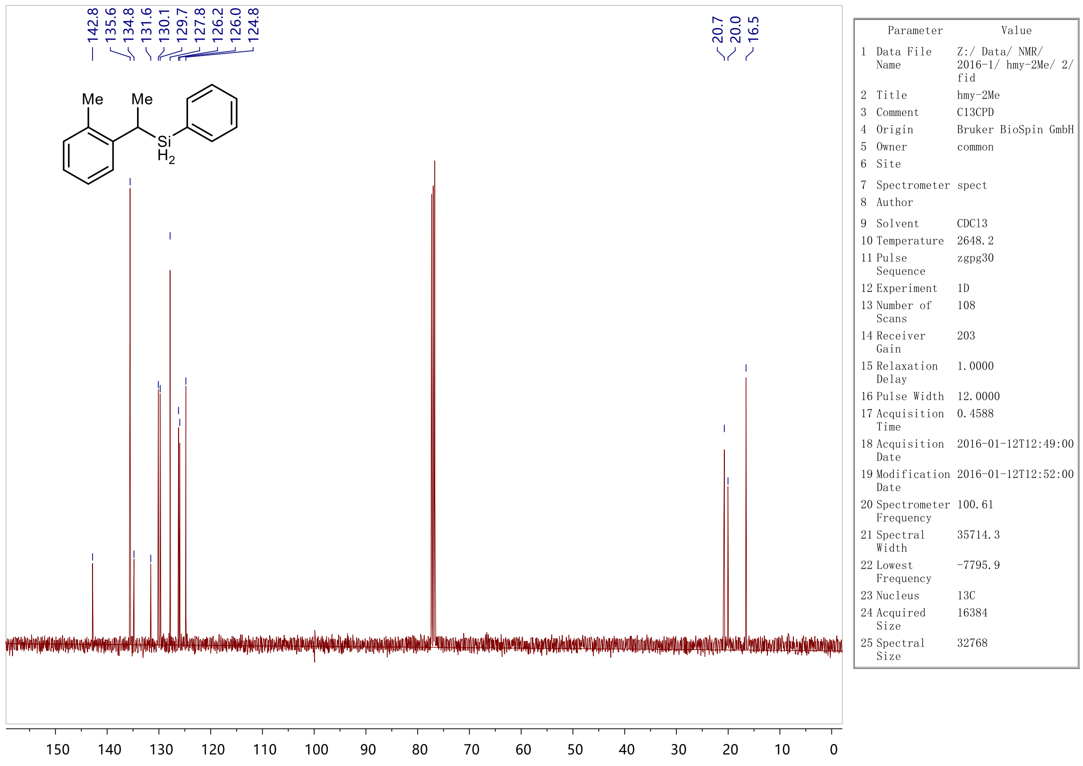

Supplementary Figure 57 | ^{13}C NMR (101 MHz, $CDCl_3$) spectra for compound 10b.

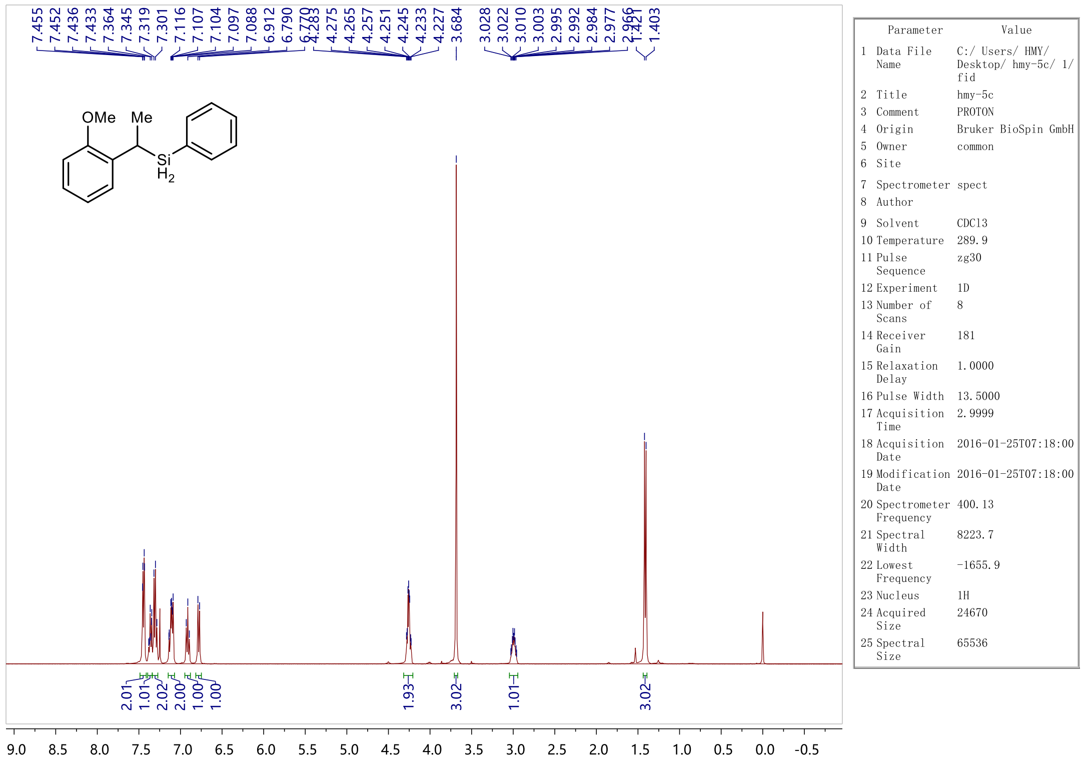

Supplementary Figure 58 | ^{1}H NMR (400 MHz, $CDCl_3$) spectra for compound 10c.

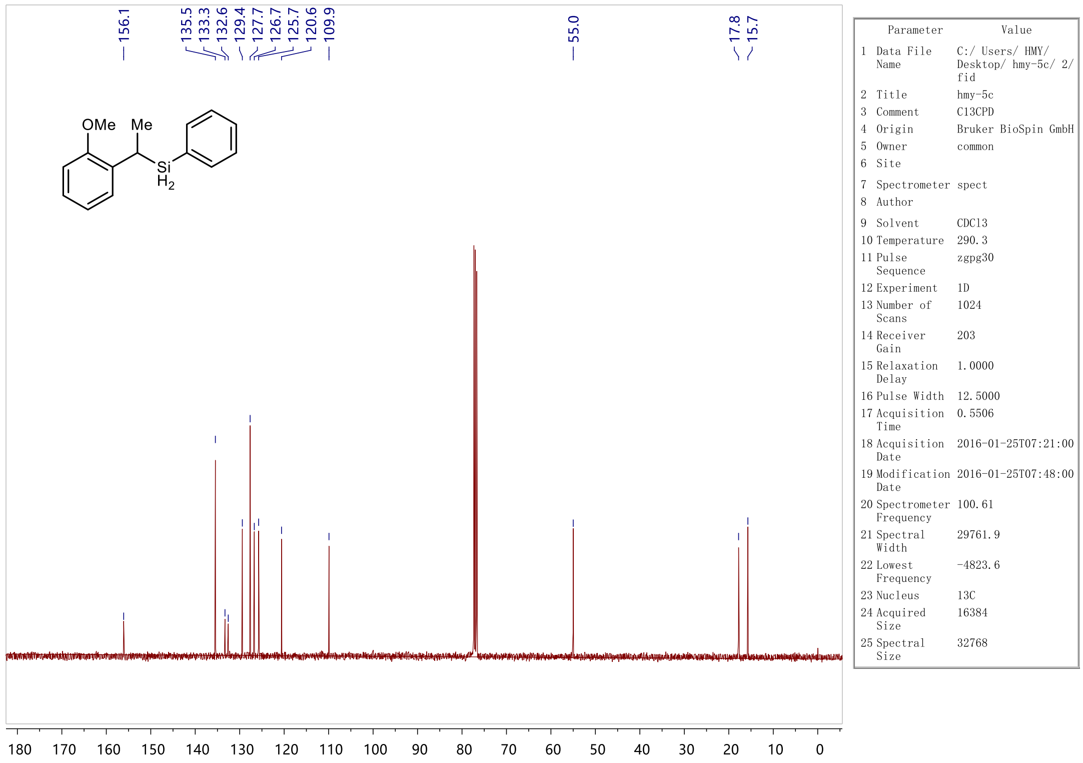

Supplementary Figure 59 | ^{13}C NMR (101 MHz, $CDCl_3$) spectra for compound 10c.

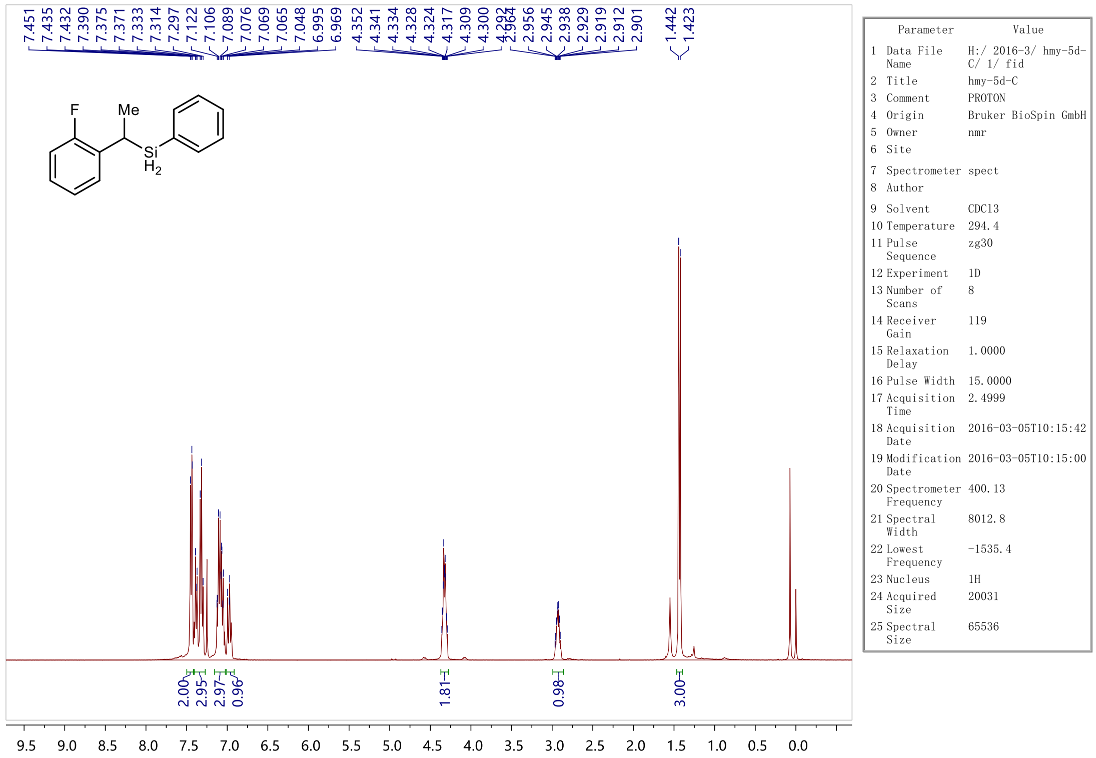

Supplementary Figure 60 | ^{1}H NMR (400 MHz, $CDCl_3$) spectra for compound 10d.

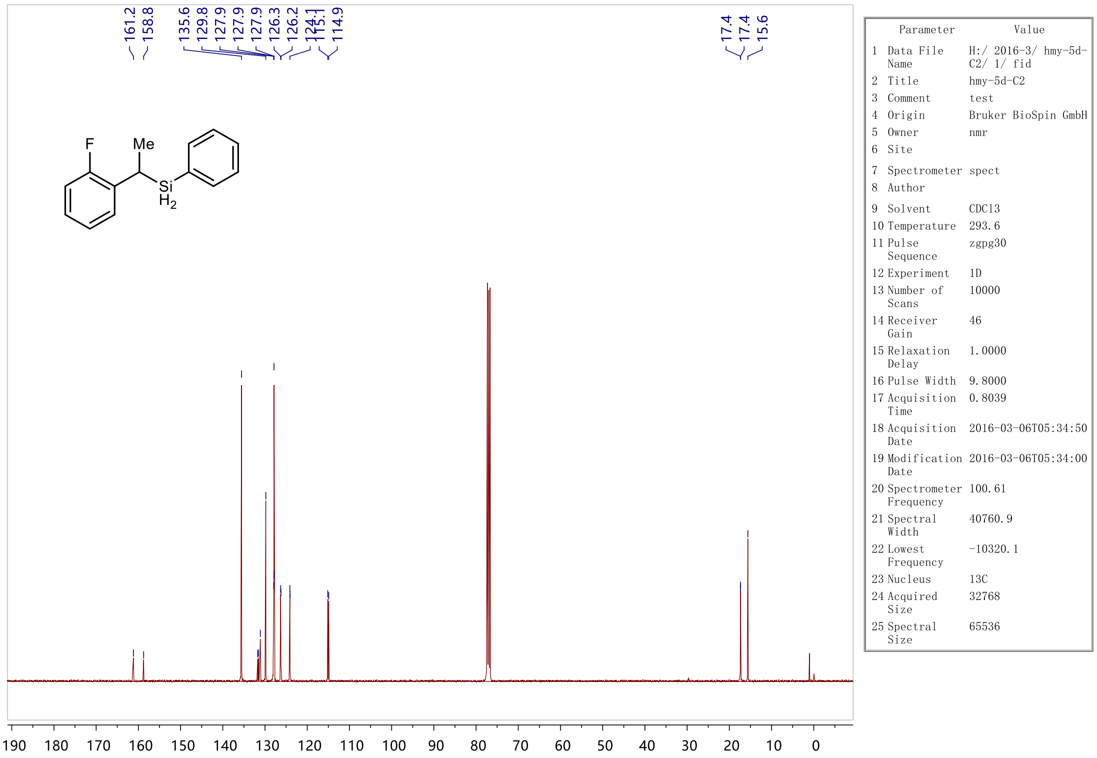

Supplementary Figure 61 | ^{13}C NMR (101 MHz, $CDCl_3$) spectra for compound 10d.

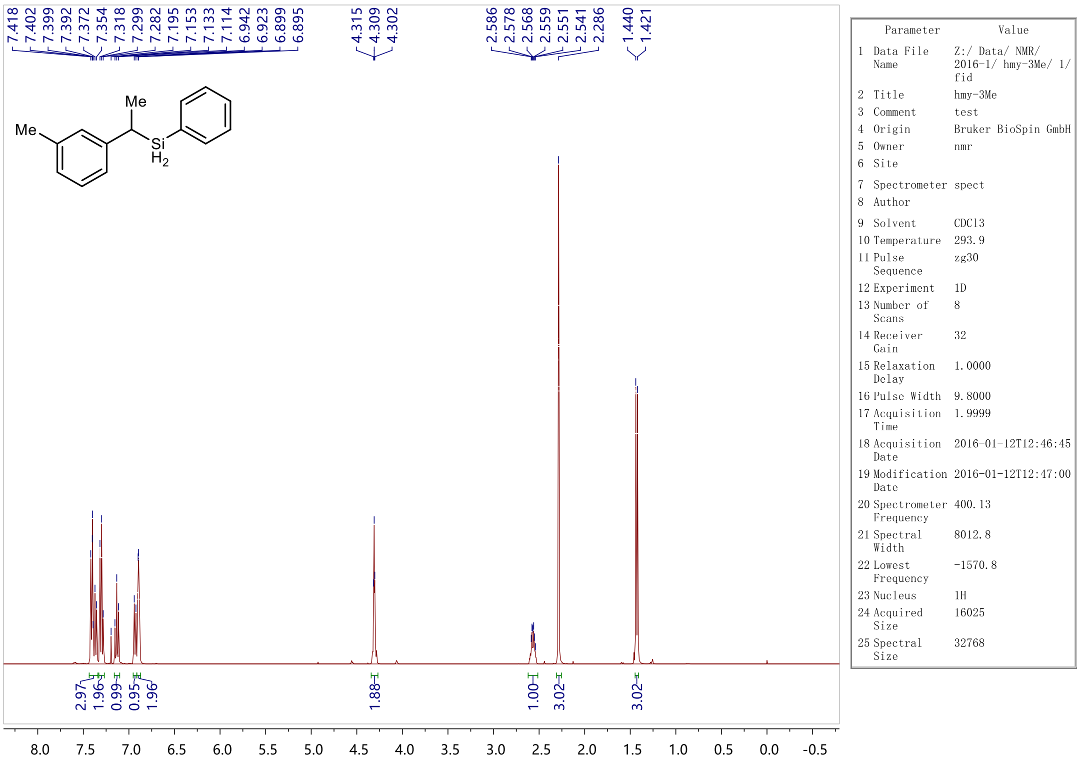

Supplementary Figure 62 | ^{1}H NMR (400 MHz, $CDCl_3$) spectra for compound 10e.

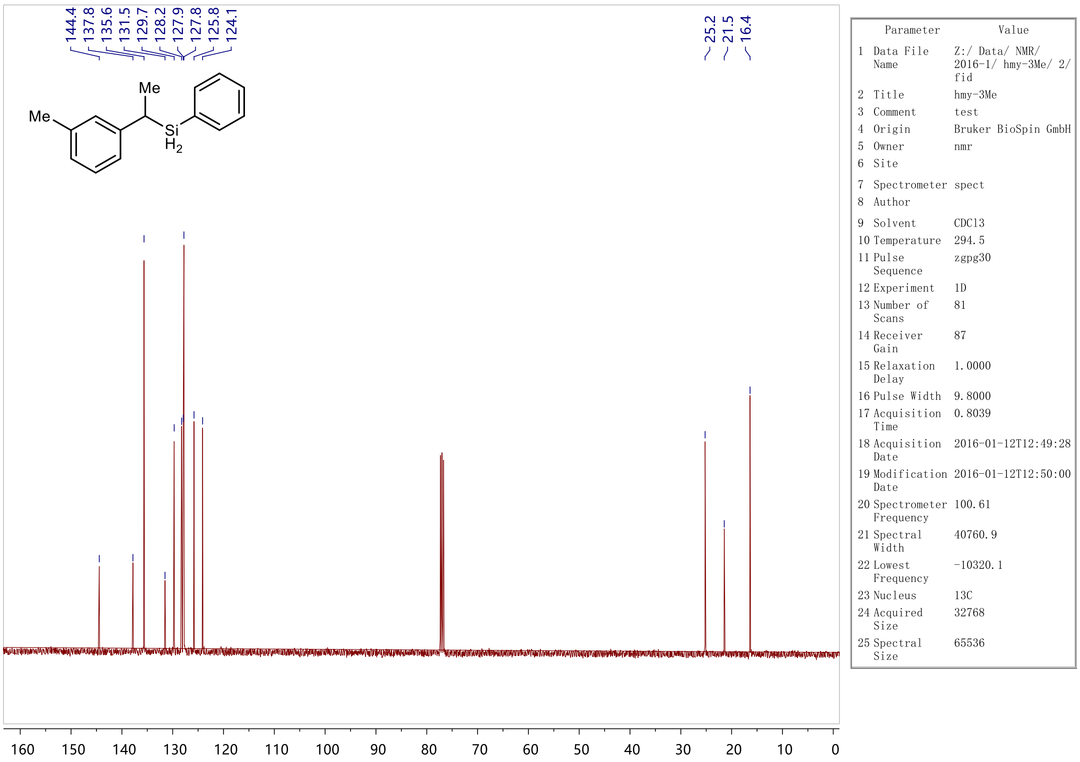

Supplementary Figure 63 | ^{13}C NMR (101 MHz, $CDCl_3$) spectra for compound 10e.

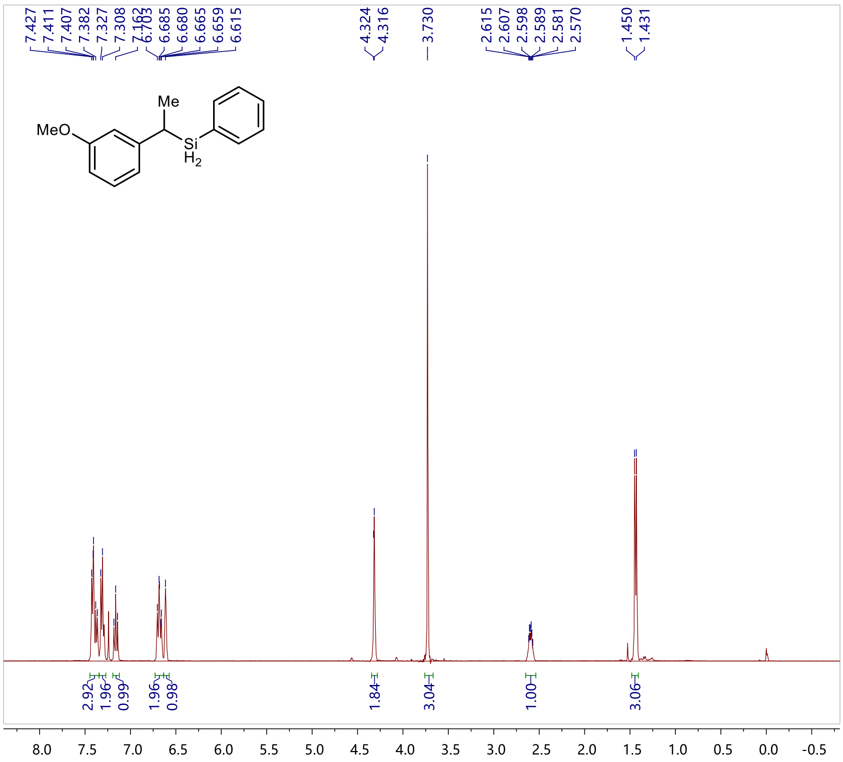

	Parameter	Value
1	Data File Name	Z:/ Data/ NMR/ 2016-1/ hmy-3MeO/ 1/ fid
2	Title	hmy-3MeO
3	Comment	PROTON
4	Origin	Bruker BioSpin GmbH
5	Owner	common
6	Site	
7	Spectrometer	spect
8	Author	
9	Solvent	CDCl3
10	Temperature	1306.8
11	Pulse Sequence	zg30
12	Experiment	1D
13	Number of Scans	8
14	Receiver Gain	203
15	Relaxation Delay	1.0000
16	Pulse Width	13.7000
17	Acquisition Time	1.9999
18	Acquisition Date	2016-01-11T07:57:00
19	Modification Date	2016-01-11T07:57:00
20	Spectrometer Frequency	400.13
21	Spectral Width	8223.7
22	Lowest Frequency	-1640.9
23	Nucleus	1H
24	Acquired Size	16446
25	Spectral Size	65536

Supplementary Figure 64 | ^{1}H NMR (400 MHz, $CDCl_3$) spectra for compound 10f.

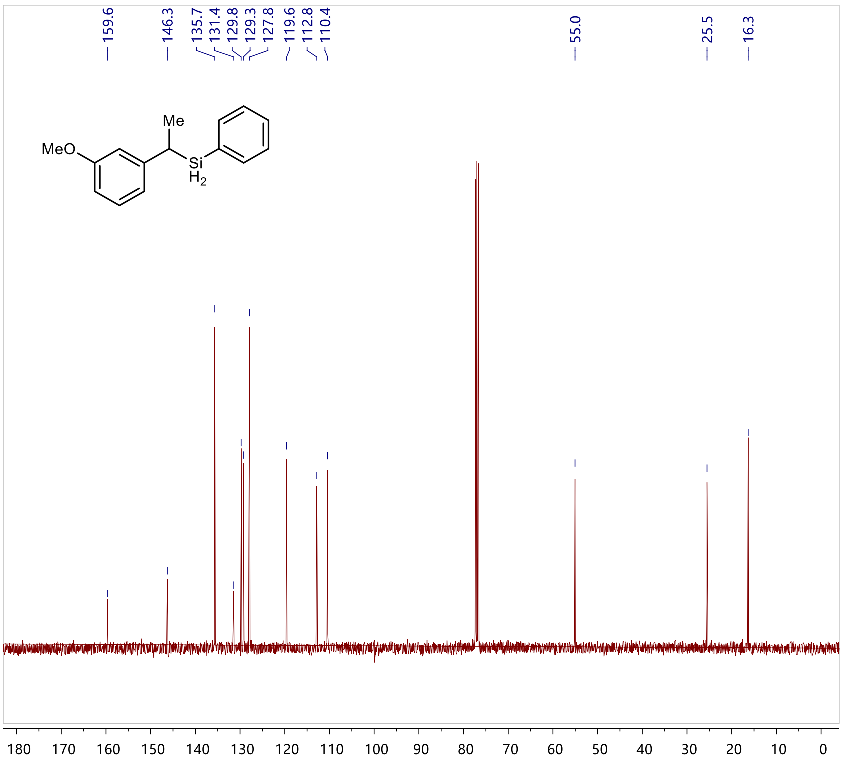

	Parameter	Value
1	Data File Name	Z:/ Data/ NMR/ 2016-1/ hmy-3MeO/ 2/ fid
2	Title	hmy-3MeO
3	Comment	C13CPD
4	Origin	Bruker BioSpin GmbH
5	Owner	common
6	Site	
7	Spectrometer	spect
8	Author	
9	Solvent	CDCl3
10	Temperature	1350.6
11	Pulse Sequence	zgpg30
12	Experiment	1D
13	Number of Scans	715
14	Receiver Gain	203
15	Relaxation Delay	1.0000
16	Pulse Width	12.0000
17	Acquisition Time	0.4588
18	Acquisition Date	2016-01-11T08:00:00
19	Modification Date	2016-01-11T08:17:00
20	Spectrometer Frequency	100.61
21	Spectral Width	35714.3
22	Lowest Frequency	-7795.9
23	Nucleus	13C
24	Acquired Size	16384
25	Spectral Size	32768

Supplementary Figure 65 | ^{13}C NMR (101 MHz, $CDCl_3$) spectra for compound 10f.

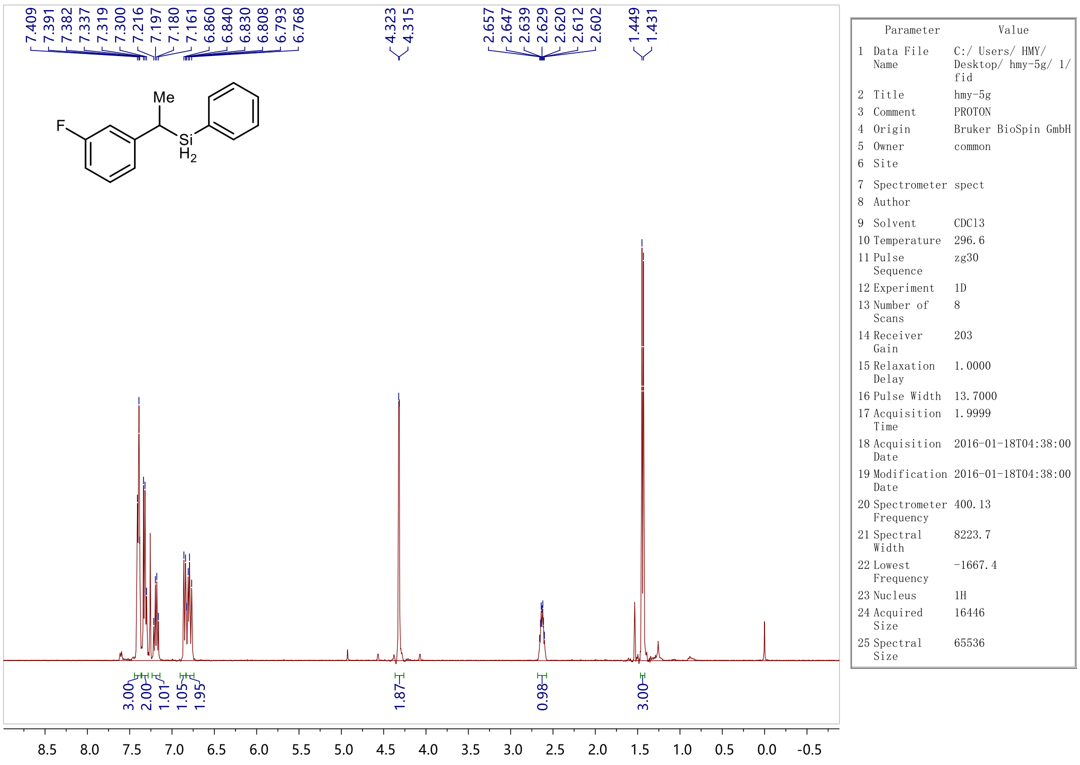

Supplementary Figure 66 | ^{1}H NMR (400 MHz, $CDCl_3$) spectra for compound 10g.

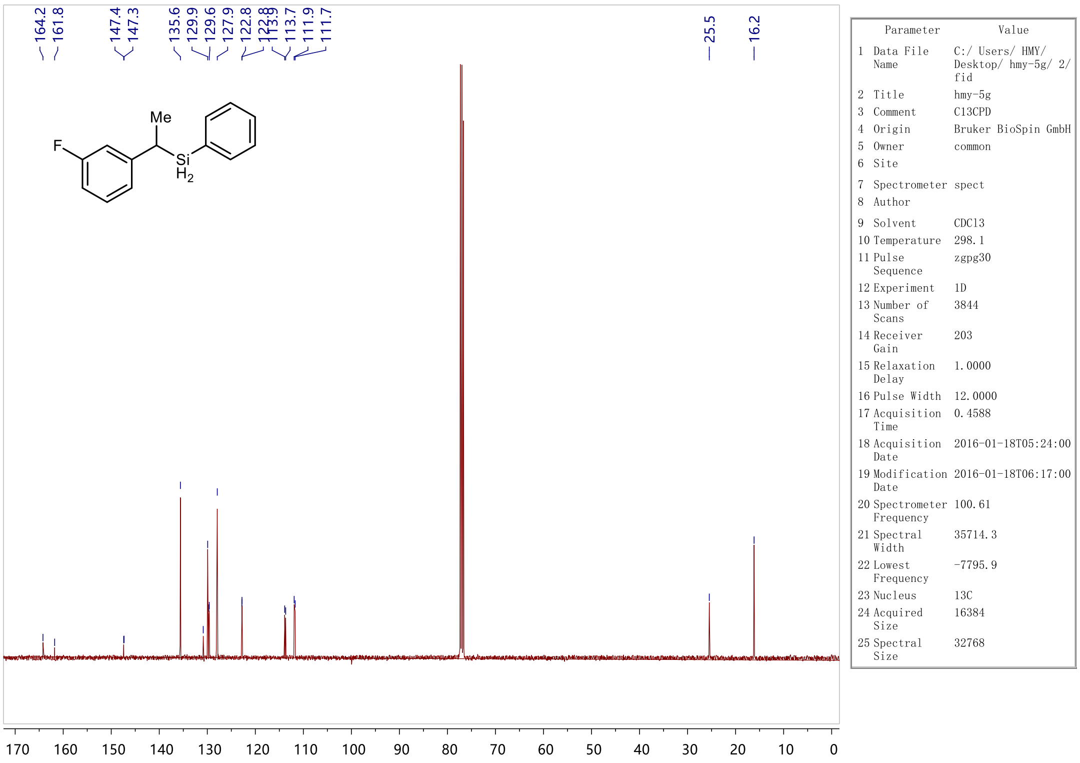

Supplementary Figure 67 | ^{13}C NMR (101 MHz, $CDCl_3$) spectra for compound 10g.

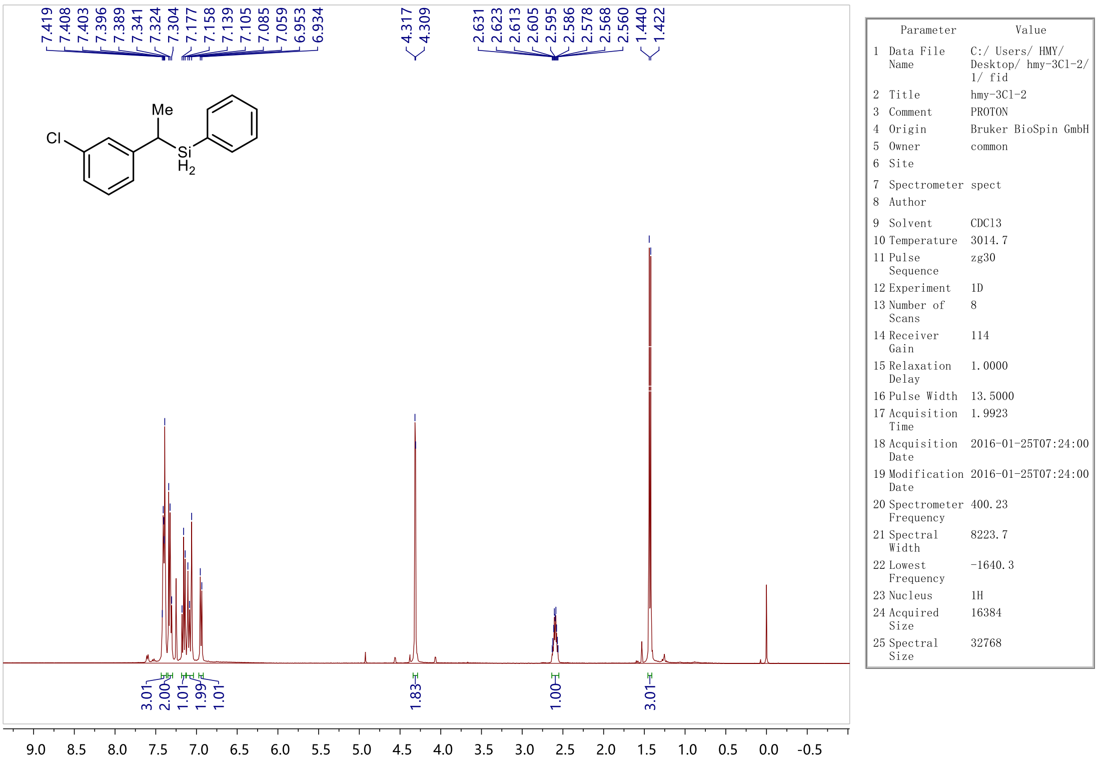

Supplementary Figure 68 | ^{1}H NMR (400 MHz, $CDCl_3$) spectra for compound 10h.

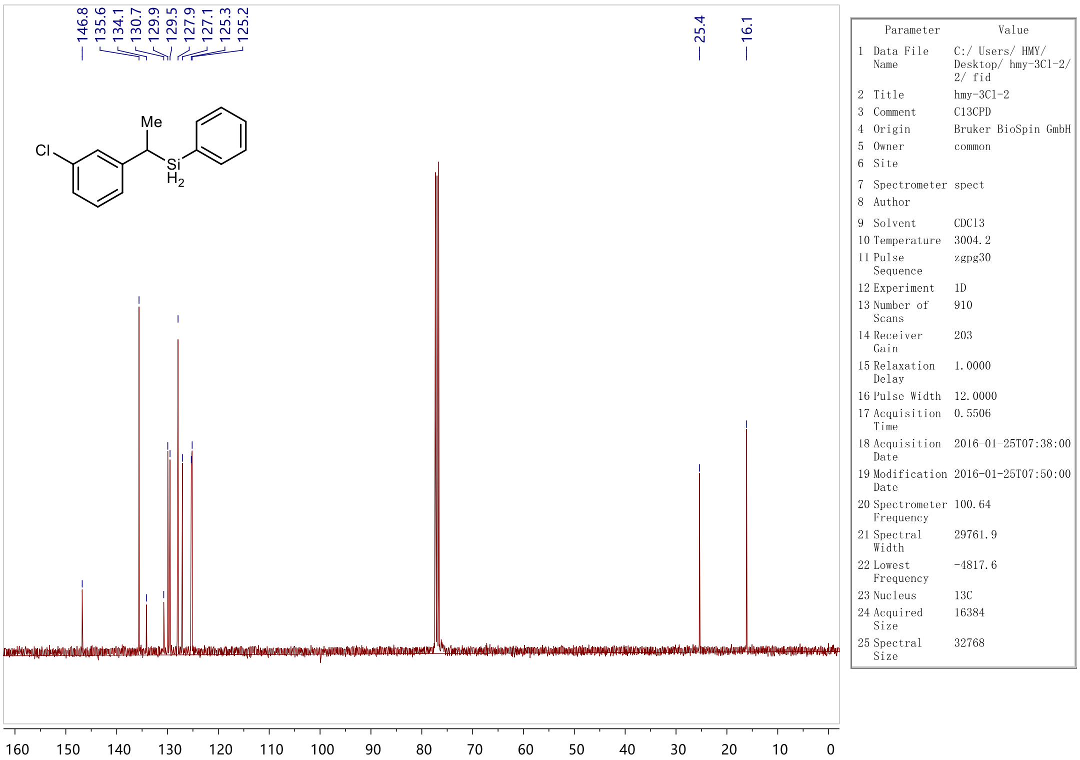

Supplementary Figure 69 | ^{13}C NMR (101 MHz, $CDCl_3$) spectra for compound 10h.

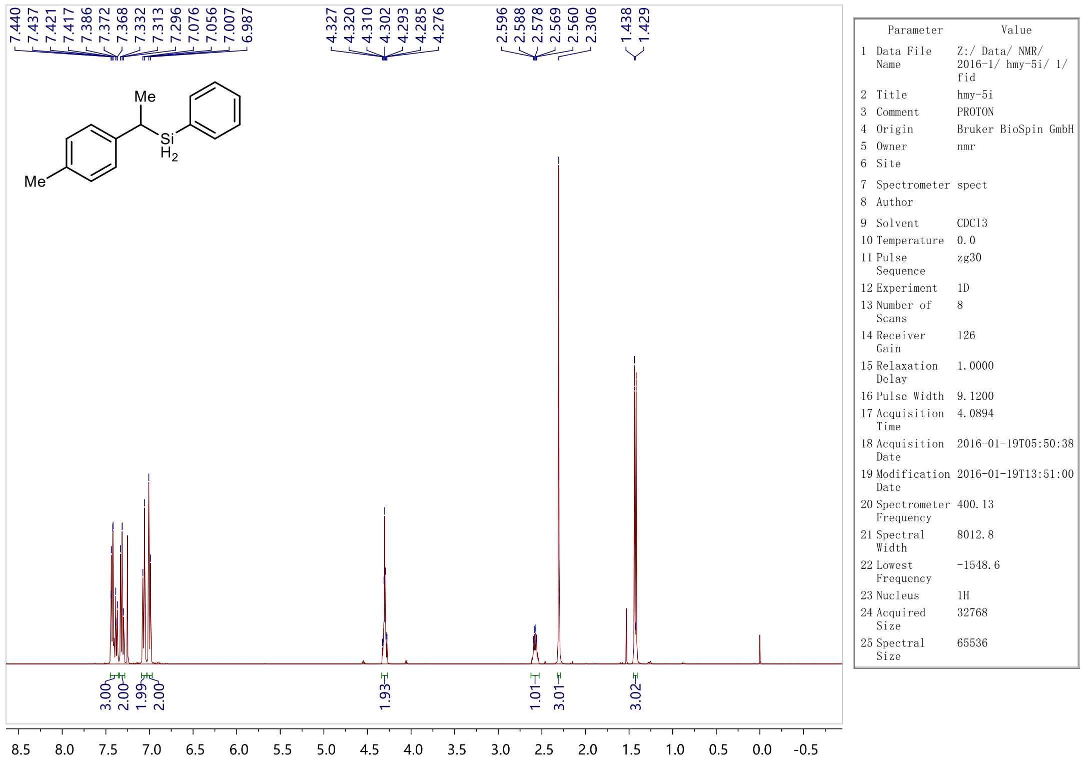

Supplementary Figure 70 | ^{1}H NMR (400 MHz, $CDCl_3$) spectra for compound 10i.

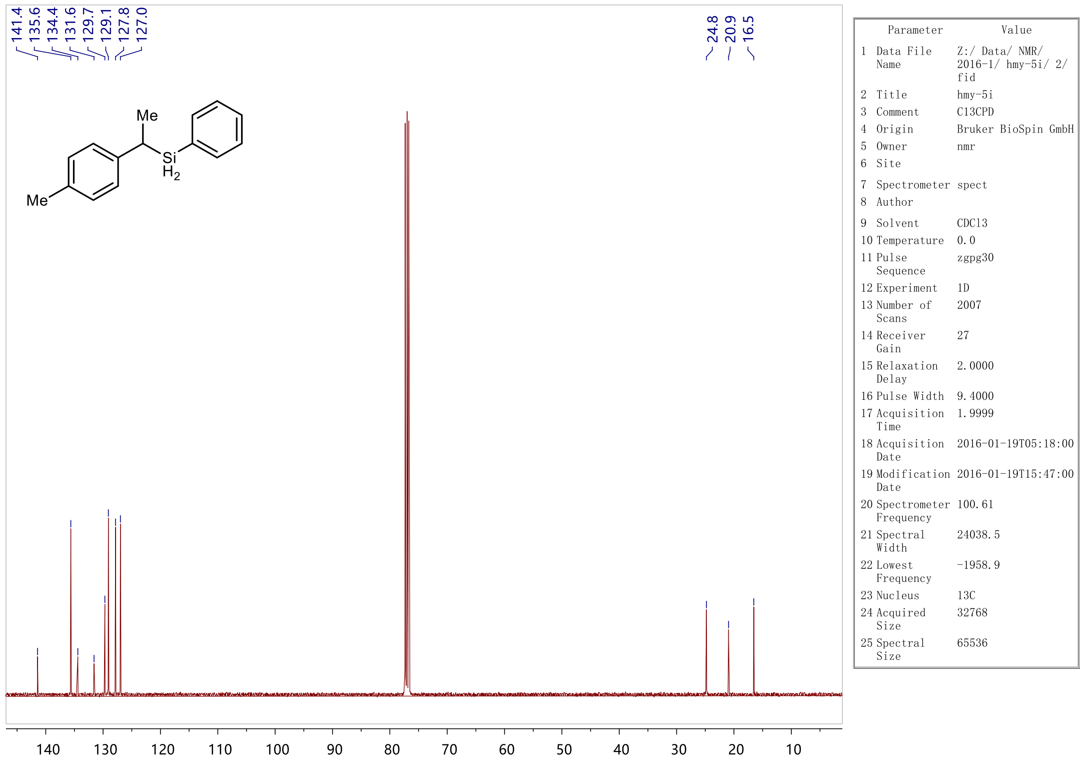

Supplementary Figure 71 | ^{13}C NMR (101 MHz, $CDCl_3$) spectra for compound 10i.

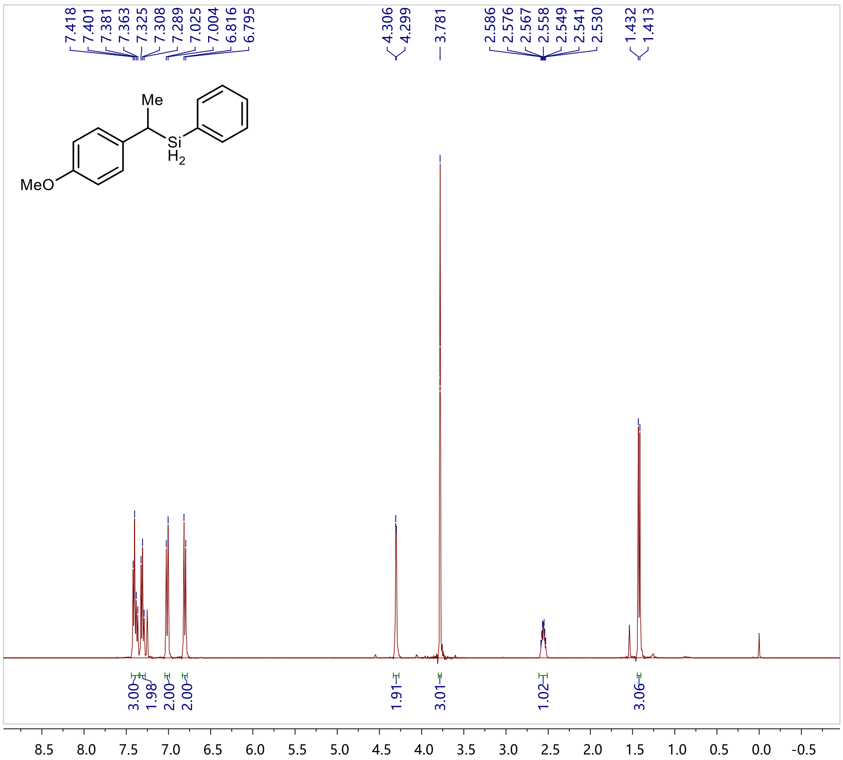

Parameter	Value
1 Data File Name	Z:/ Data/ NMR/ 2016-1/ hmy-4MeO-1/ 1/ fid
2 Title	hmy-4MeO-1
3 Comment	PROTON
4 Origin	Bruker BioSpin GmbH
5 Owner	common
6 Site	
7 Spectrometer	spect
8 Author	
9 Solvent	CDCl3
10 Temperature	296.9
11 Pulse Sequence	zg30
12 Experiment	1D
13 Number of Scans	8
14 Receiver Gain	203
15 Relaxation Delay	1.0000
16 Pulse Width	13.7000
17 Acquisition Time	1.9999
18 Acquisition Date	2016-01-17T22:08:00
19 Modification Date	2016-01-17T22:08:00
20 Spectrometer Frequency	400.13
21 Spectral Width	8223.7
22 Lowest Frequency	-1671.5
23 Nucleus	1H
24 Acquired Size	16446
25 Spectral Size	65536

Supplementary Figure 72 | ^{1}H NMR (400 MHz, $CDCl_3$) spectra for compound 10j.

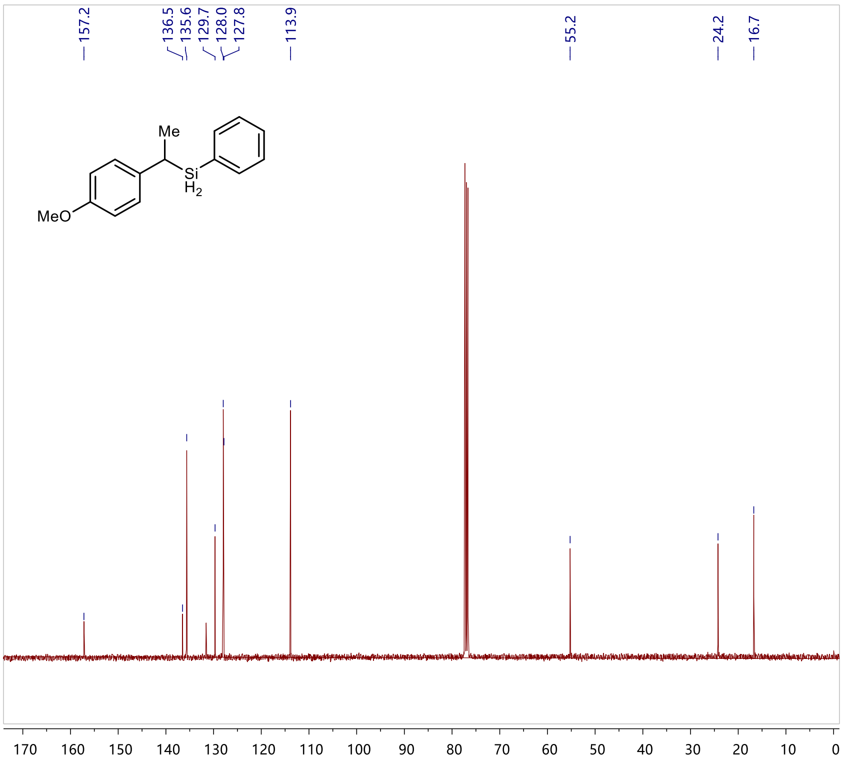

Parameter	Value
1 Data File Name	Z:/ Data/ NMR/ 2016-1/ hmy-4MeO-1/ 2/ fid
2 Title	hmy-4MeO-1
3 Comment	C13CPD
4 Origin	Bruker BioSpin GmbH
5 Owner	common
6 Site	
7 Spectrometer	spect
8 Author	
9 Solvent	CDCl3
10 Temperature	298.2
11 Pulse Sequence	zgpg30
12 Experiment	1D
13 Number of Scans	1489
14 Receiver Gain	203
15 Relaxation Delay	1.0000
16 Pulse Width	12.0000
17 Acquisition Time	0.4588
18 Acquisition Date	2016-01-17T22:18:00
19 Modification Date	2016-01-17T22:47:00
20 Spectrometer Frequency	100.61
21 Spectral Width	35714.3
22 Lowest Frequency	-7795.9
23 Nucleus	13C
24 Acquired Size	16384
25 Spectral Size	32768

Supplementary Figure 73 | ^{13}C NMR (101 MHz, $CDCl_3$) spectra for compound 10j.

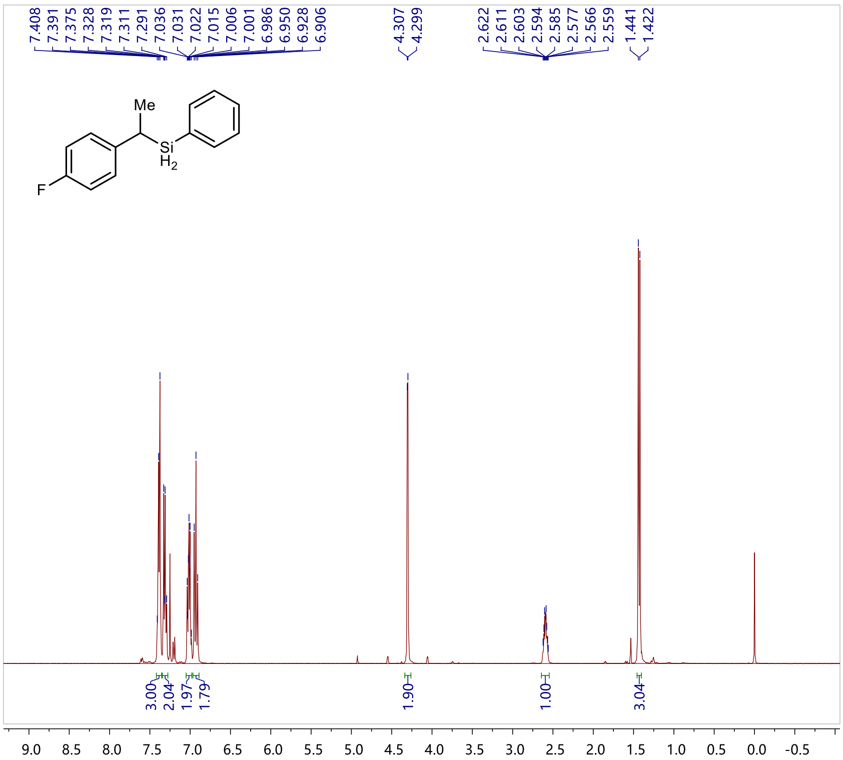

Parameter		Value
1	Data File Name	E:/ date/ 2016-1/ hmy-4F-1/ 4/ fid
2	Title	hmy-4F-1
3	Comment	PROTON
4	Origin	Bruker BioSpin GmbH
5	Owner	common
6	Site	
7	Spectrometer	spect
8	Author	
9	Solvent	CDCl3
10	Temperature	293.6
11	Pulse Sequence	zg30
12	Experiment	1D
13	Number of Scans	8
14	Receiver Gain	203
15	Relaxation Delay	1.0000
16	Pulse Width	13.7000
17	Acquisition Time	1.9999
18	Acquisition Date	2016-01-25T07:24:00
19	Modification Date	2016-01-25T07:24:00
20	Spectrometer Frequency	400.13
21	Spectral Width	8223.7
22	Lowest Frequency	-1640.9
23	Nucleus	1H
24	Acquired Size	16446
25	Spectral Size	65536

Supplementary Figure 74 | ^{1}H NMR (400 MHz, $CDCl_3$) spectra for compound 10k.

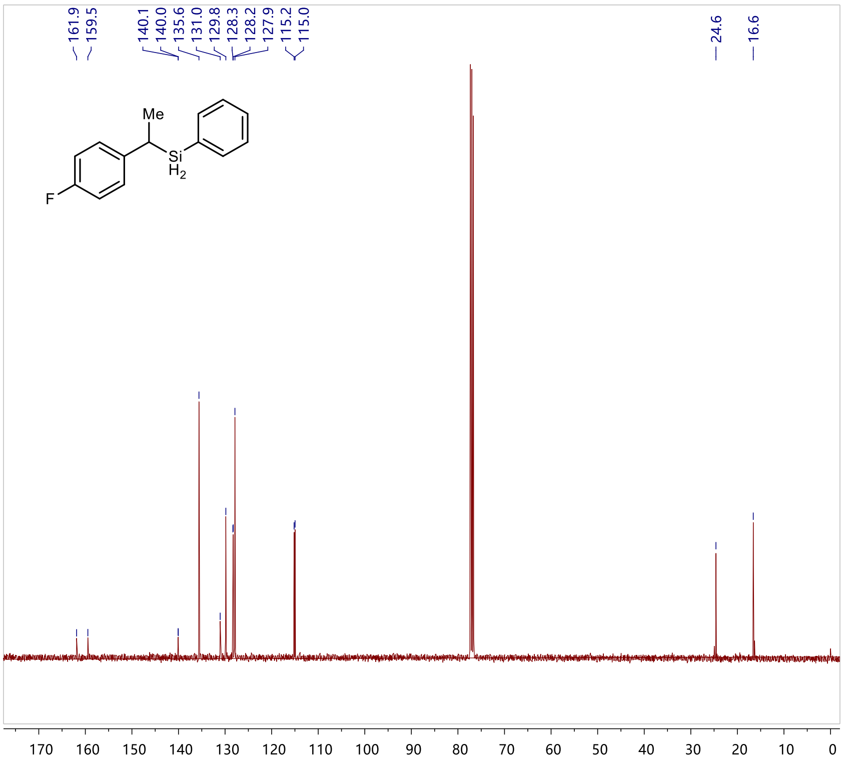

Parameter		Value
1	Data File Name	E:/ date/ 2016-1/ hmy-4F-1/ 5/ fid
2	Title	hmy-4F-1
3	Comment	C13CPD
4	Origin	Bruker BioSpin GmbH
5	Owner	common
6	Site	
7	Spectrometer	spect
8	Author	
9	Solvent	CDCl3
10	Temperature	294.5
11	Pulse Sequence	zgpg30
12	Experiment	1D
13	Number of Scans	1024
14	Receiver Gain	203
15	Relaxation Delay	1.0000
16	Pulse Width	12.0000
17	Acquisition Time	0.4588
18	Acquisition Date	2016-01-25T07:28:00
19	Modification Date	2016-01-25T07:52:00
20	Spectrometer Frequency	100.61
21	Spectral Width	35714.3
22	Lowest Frequency	-7795.9
23	Nucleus	13C
24	Acquired Size	16384
25	Spectral Size	32768

Supplementary Figure 75 | ^{13}C NMR (101 MHz, $CDCl_3$) spectra for compound 10k.

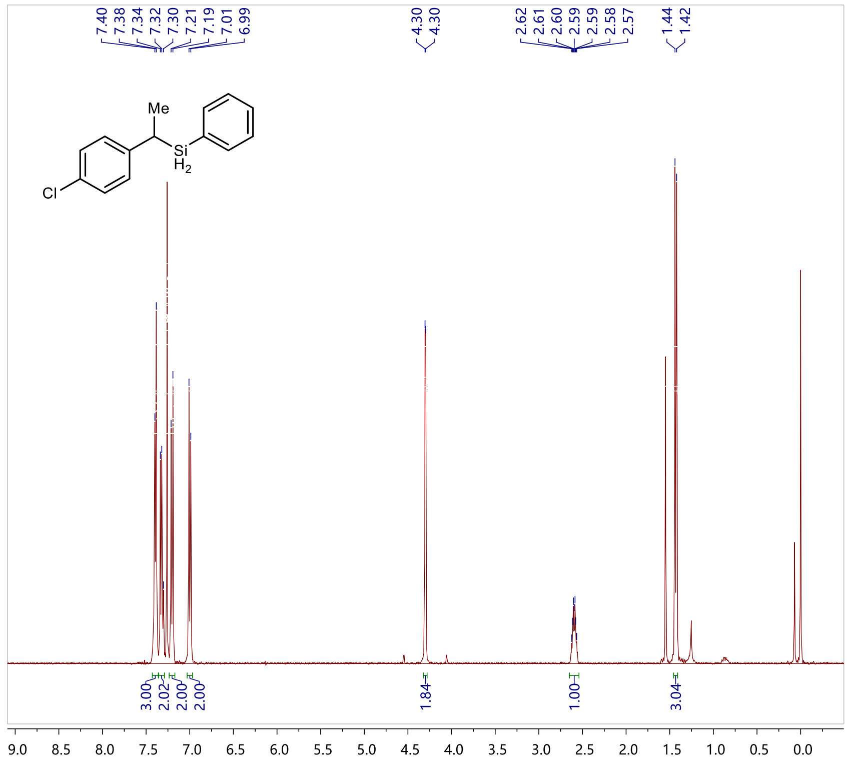

	Parameter	Value
1	Data File Name	Z:/ Data/ NMR/ 2016-1/ hmy-51-3/ 1/ fid
2	Title	hmy-51-3
3	Comment	PROTON
4	Origin	Bruker BioSpin GmbH
5	Owner	common
6	Site	
7	Spectrometer	spect
8	Author	
9	Solvent	CDCl3
10	Temperature	294.5
11	Pulse Sequence	zg30
12	Experiment	1D
13	Number of Scans	8
14	Receiver Gain	203
15	Relaxation Delay	1.0000
16	Pulse Width	13.7000
17	Acquisition Time	1.9999
18	Acquisition Date	2016-01-22T23:01:00
19	Modification Date	2016-01-22T23:01:00
20	Spectrometer Frequency	400.13
21	Spectral Width	8223.7
22	Lowest Frequency	-1659.2
23	Nucleus	1H
24	Acquired Size	16446
25	Spectral Size	65536

Supplementary Figure 76 | ^{1}H NMR (400 MHz, $CDCl_3$) spectra for compound 10l.

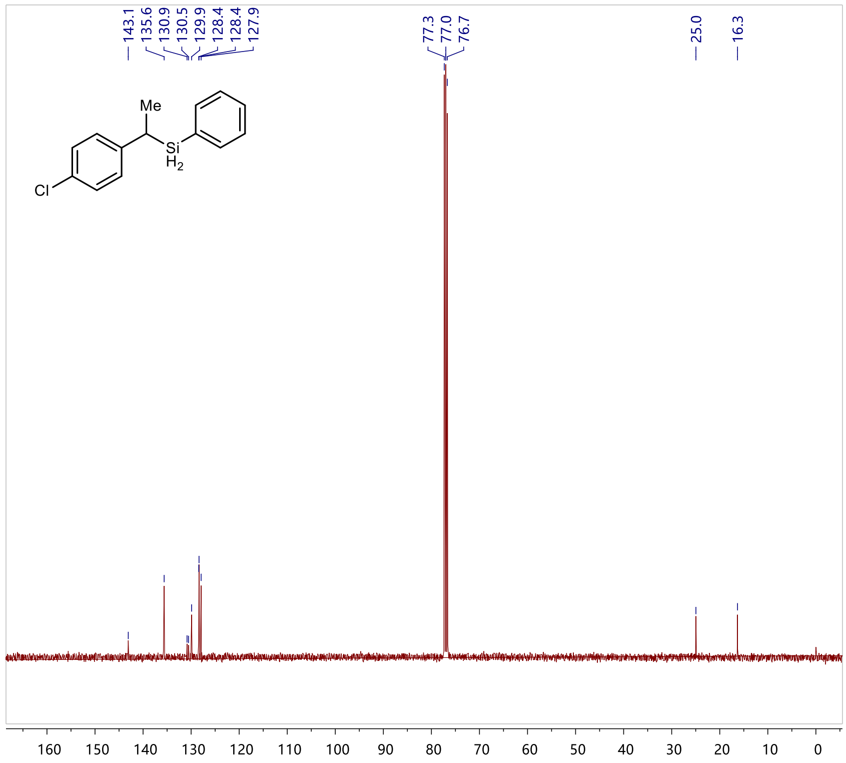

	Parameter	Value
1	Data File Name	Z:/ Data/ NMR/ 2016-1/ hmy-51-3/ 2/ fid
2	Title	hmy-51-3
3	Comment	C13CPD
4	Origin	Bruker BioSpin GmbH
5	Owner	common
6	Site	
7	Spectrometer	spect
8	Author	
9	Solvent	CDCl3
10	Temperature	294.6
11	Pulse Sequence	zgpg30
12	Experiment	1D
13	Number of Scans	1304
14	Receiver Gain	203
15	Relaxation Delay	1.0000
16	Pulse Width	12.0000
17	Acquisition Time	0.4588
18	Acquisition Date	2016-01-22T23:02:00
19	Modification Date	2016-01-22T23:35:00
20	Spectrometer Frequency	100.61
21	Spectral Width	35714.3
22	Lowest Frequency	-7795.9
23	Nucleus	13C
24	Acquired Size	16384
25	Spectral Size	32768

Supplementary Figure 77 | ^{13}C NMR (101 MHz, $CDCl_3$) spectra for compound 10l.

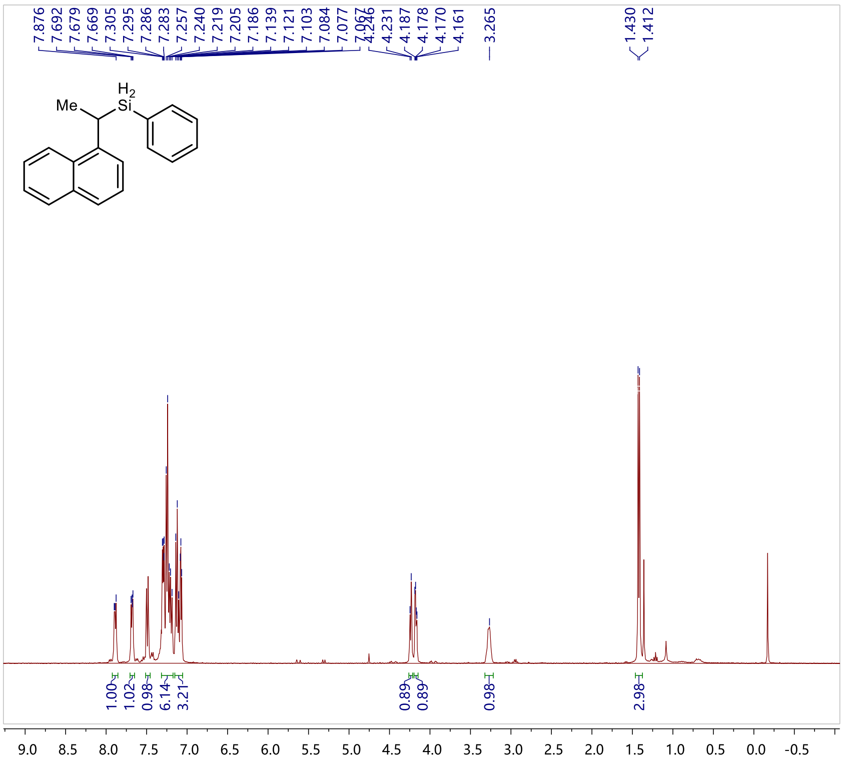

Parameter		Value
1	Data File Name	I:/ 数据整理/ 产物谱图/ hmy-2Na/ 1/ fid
2	Title	hmy-2Na
3	Comment	PROTON
4	Origin	Bruker BioSpin GmbH
5	Owner	nmr
6	Site	
7	Spectrometer	spect
8	Author	
9	Solvent	CDC13
10	Temperature	295.2
11	Pulse Sequence	zg30
12	Experiment	1D
13	Number of Scans	8
14	Receiver Gain	199
15	Relaxation Delay	1.0000
16	Pulse Width	15.0000
17	Acquisition Time	2.4999
18	Acquisition Date	2016-01-01T15:30:10
19	Modification Date	2016-01-01T15:30:00
20	Spectrometer Frequency	400.13
21	Spectral Width	8012.8
22	Lowest Frequency	-1619.6
23	Nucleus	1H
24	Acquired Size	20031
25	Spectral Size	65536

Supplementary Figure 78 | ^{1}H NMR (400 MHz, $CDCl_3$) spectra for compound 10m.

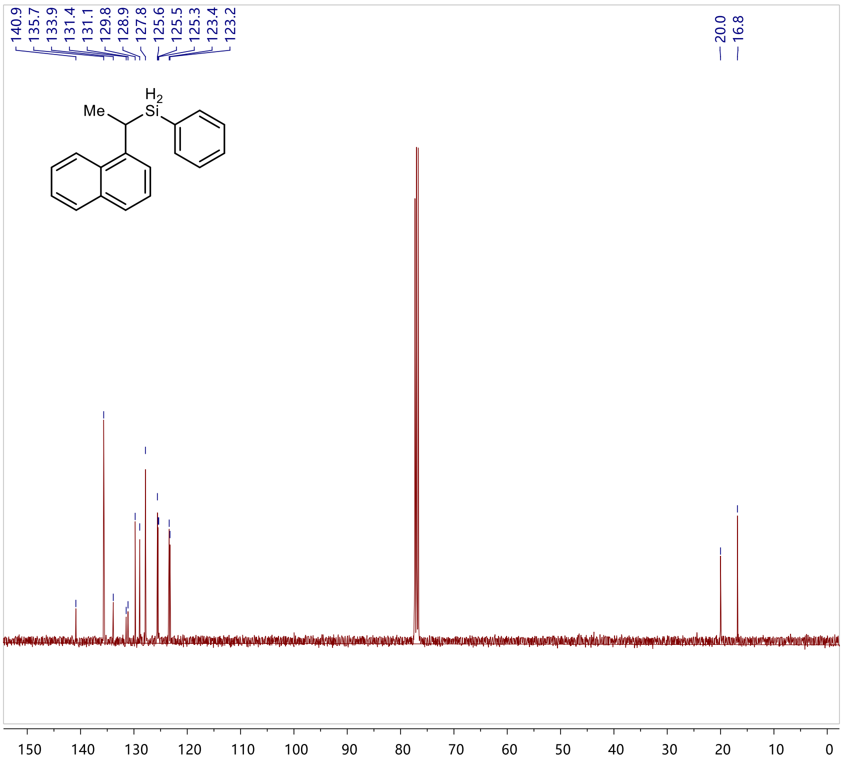

Parameter		Value
1	Data File Name	I:/ 数据整理/ 产物谱图/ hmy-2Na/ 2/ fid
2	Title	hmy-2Na
3	Comment	C13CPD
4	Origin	Bruker BioSpin GmbH
5	Owner	nmr
6	Site	
7	Spectrometer	spect
8	Author	
9	Solvent	CDC13
10	Temperature	295.7
11	Pulse Sequence	zgpg30
12	Experiment	1D
13	Number of Scans	786
14	Receiver Gain	87
15	Relaxation Delay	1.0000
16	Pulse Width	9.8000
17	Acquisition Time	0.4588
18	Acquisition Date	2016-01-01T15:33:57
19	Modification Date	2016-01-01T15:51:00
20	Spectrometer Frequency	100.61
21	Spectral Width	35714.3
22	Lowest Frequency	-7796.8
23	Nucleus	13C
24	Acquired Size	16384
25	Spectral Size	32768

Supplementary Figure 79 | ^{13}C NMR (101 MHz, $CDCl_3$) spectra for compound 10m.

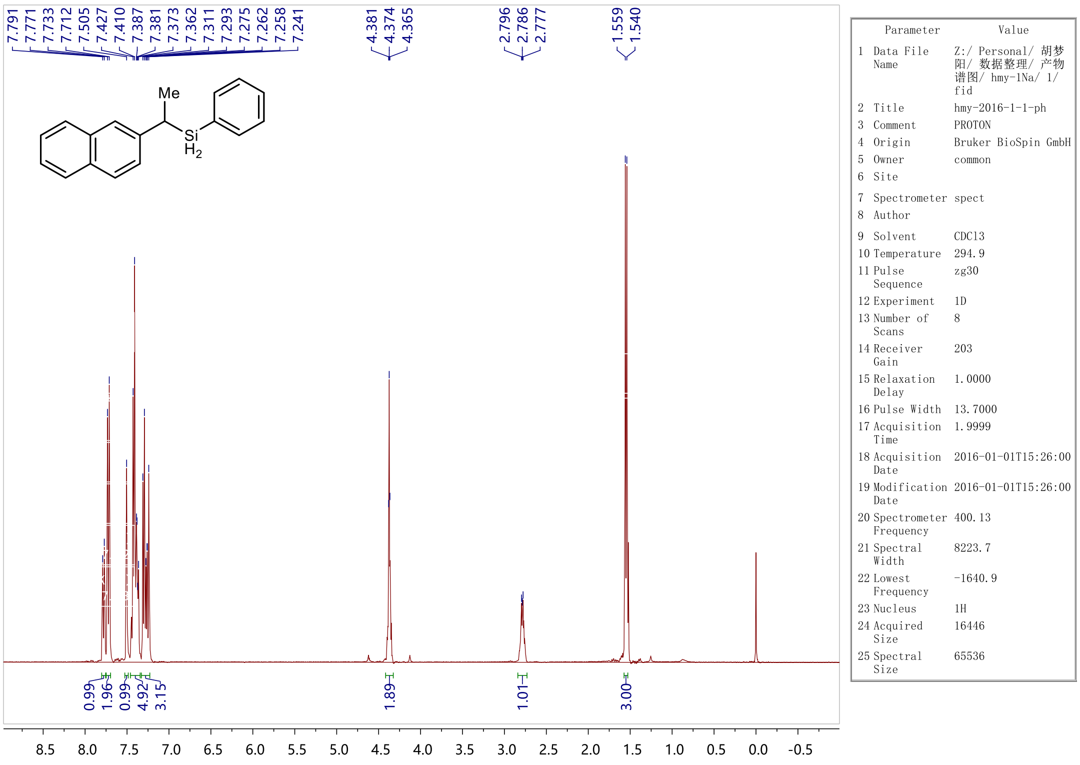

Supplementary Figure 80 | ^{1}H NMR (400 MHz, $CDCl_3$) spectra for compound 10n.

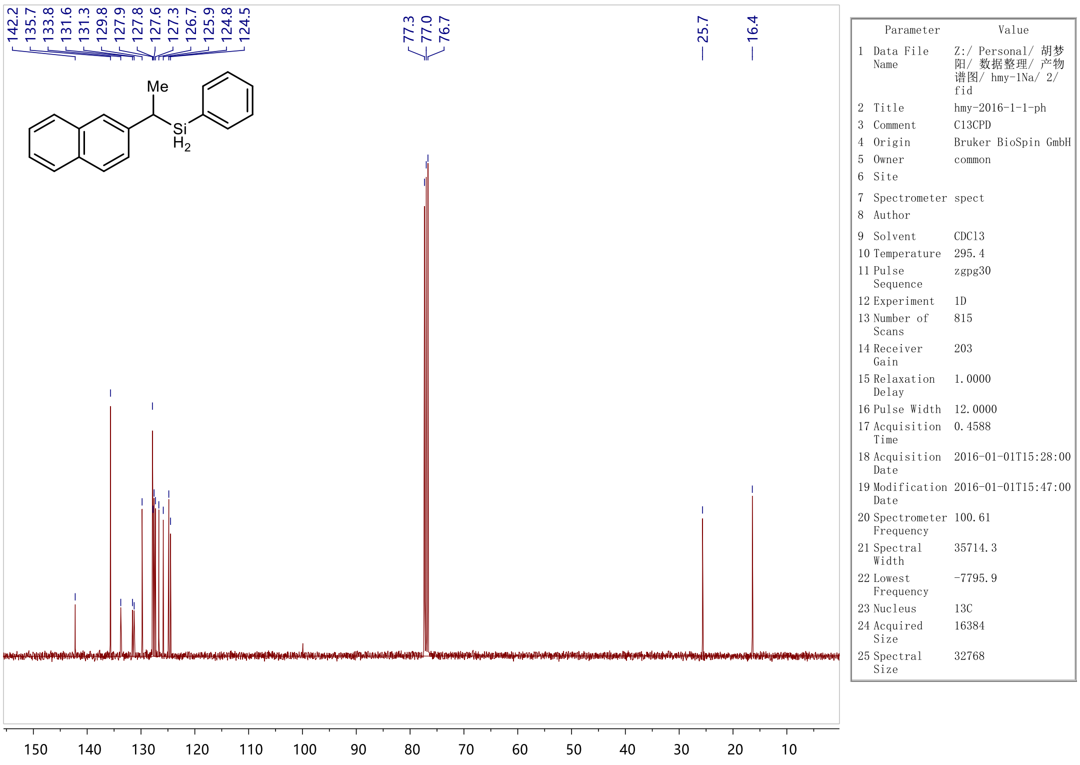

Supplementary Figure 81 | ^{13}C NMR (101 MHz, $CDCl_3$) spectra for compound 10n.

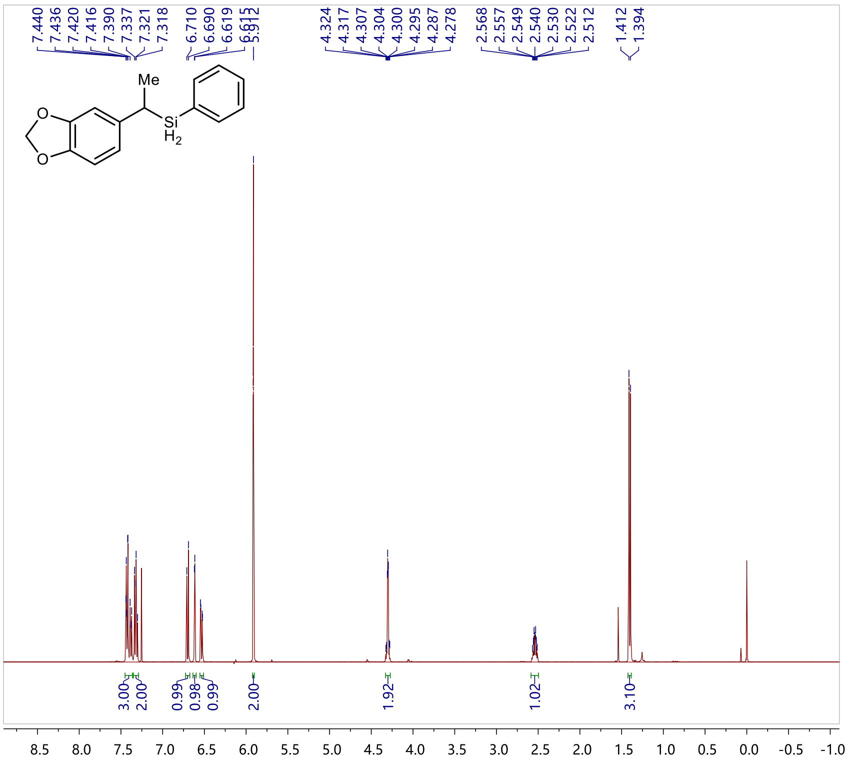

Parameter	Value
1 Data File Name	C:/ Users/ HMY/ Desktop/ hmy-5o-1/ 1/ fid
2 Title	hmy-5o-1
3 Comment	test
4 Origin	Bruker BioSpin GmbH
5 Owner	nmr
6 Site	
7 Spectrometer	spect
8 Author	
9 Solvent	CDCl3
10 Temperature	294.9
11 Pulse Sequence	zg30
12 Experiment	1D
13 Number of Scans	8
14 Receiver Gain	194
15 Relaxation Delay	1.0000
16 Pulse Width	9.8000
17 Acquisition Time	1.9999
18 Acquisition Date	2016-01-18T05:28:07
19 Modification Date	2016-01-18T05:28:00
20 Spectrometer Frequency	400.13
21 Spectral Width	8012.8
22 Lowest Frequency	-1535.4
23 Nucleus	1H
24 Acquired Size	16025
25 Spectral Size	32768

Supplementary Figure 82 | ^{1}H NMR (400 MHz, $CDCl_3$) spectra for compound 10o.

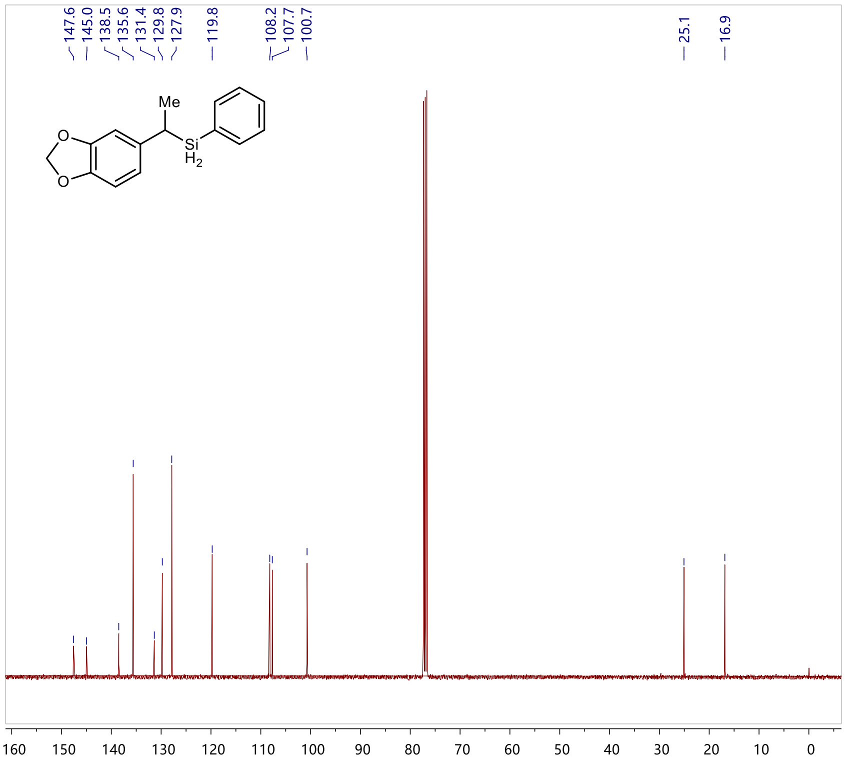

Parameter	Value
1 Data File Name	C:/ Users/ HMY/ Desktop/ hmy-5o-1/ 2/ fid
2 Title	hmy-5o-1
3 Comment	test
4 Origin	Bruker BioSpin GmbH
5 Owner	nmr
6 Site	
7 Spectrometer	spect
8 Author	
9 Solvent	CDCl3
10 Temperature	295.4
11 Pulse Sequence	zgpg30
12 Experiment	1D
13 Number of Scans	1709
14 Receiver Gain	40
15 Relaxation Delay	1.0000
16 Pulse Width	9.8000
17 Acquisition Time	0.8039
18 Acquisition Date	2016-01-18T05:31:50
19 Modification Date	2016-01-18T06:23:00
20 Spectrometer Frequency	100.61
21 Spectral Width	40760.9
22 Lowest Frequency	-10320.1
23 Nucleus	13C
24 Acquired Size	32768
25 Spectral Size	65536

Supplementary Figure 83 | ^{13}C NMR (101 MHz, $CDCl_3$) spectra for compound 10o.

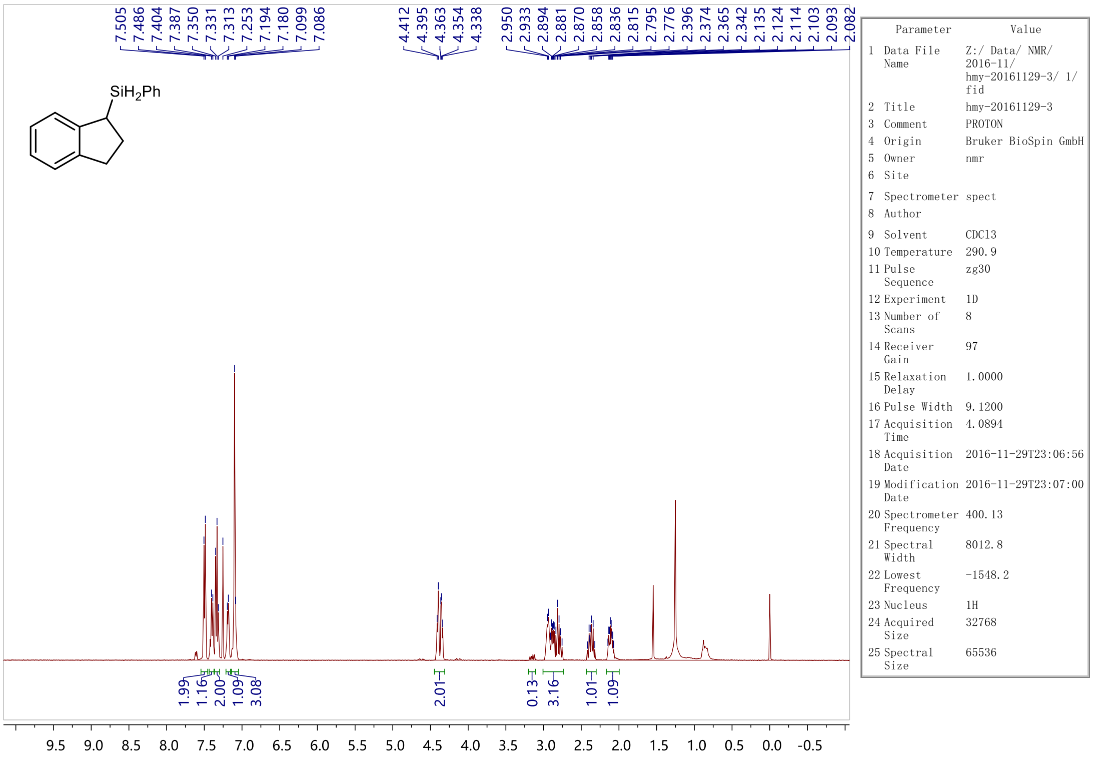

Supplementary Figure 84 | ^{1}H NMR (400 MHz, $CDCl_3$) spectra for compound 12a.

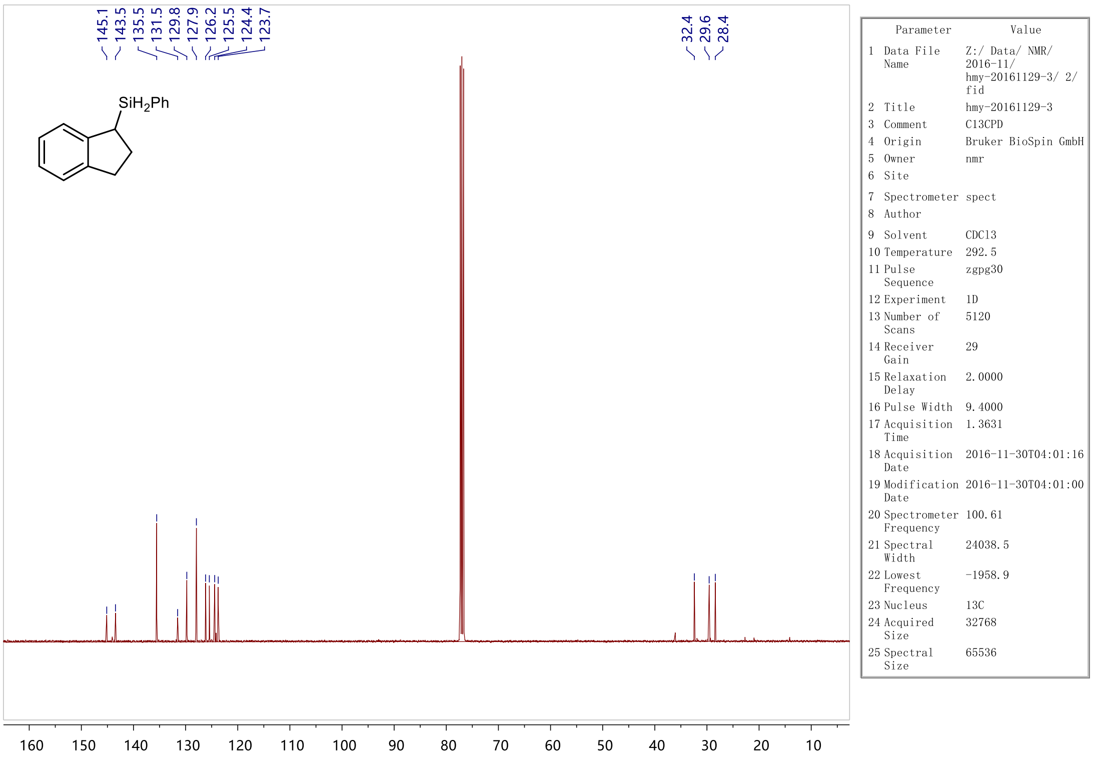

Supplementary Figure 85 | ^{13}C NMR (101 MHz, $CDCl_3$) spectra for compound 12a.

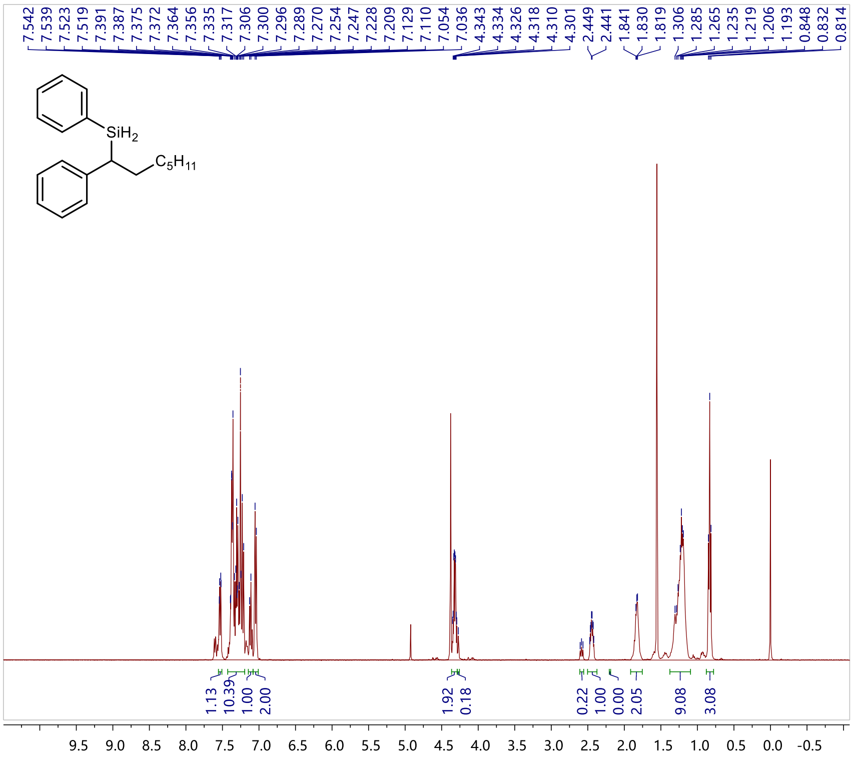

	Parameter	Value
1	Data File Name	Z:/ Data/ NMR/ 2016-09/ hmy-7-57-1/ 1/ fid
2	Title	hmy-7-57-1
3	Comment	PROTON
4	Origin	Bruker BioSpin GmbH
5	Owner	common
6	Site	
7	Spectrometer	spect
8	Author	
9	Solvent	CDCl3
10	Temperature	291.3
11	Pulse Sequence	zg30
12	Experiment	1D
13	Number of Scans	8
14	Receiver Gain	203
15	Relaxation Delay	1.0000
16	Pulse Width	13.5000
17	Acquisition Time	2.9999
18	Acquisition Date	2016-09-03T09:47:00
19	Modification Date	2016-09-03T09:47:00
20	Spectrometer Frequency	400.13
21	Spectral Width	8223.7
22	Lowest Frequency	-1653.6
23	Nucleus	1H
24	Acquired Size	24670
25	Spectral Size	65536

Supplementary Figure 86 | ^{1}H NMR (400 MHz, $CDCl_3$) spectra for compound 12b.

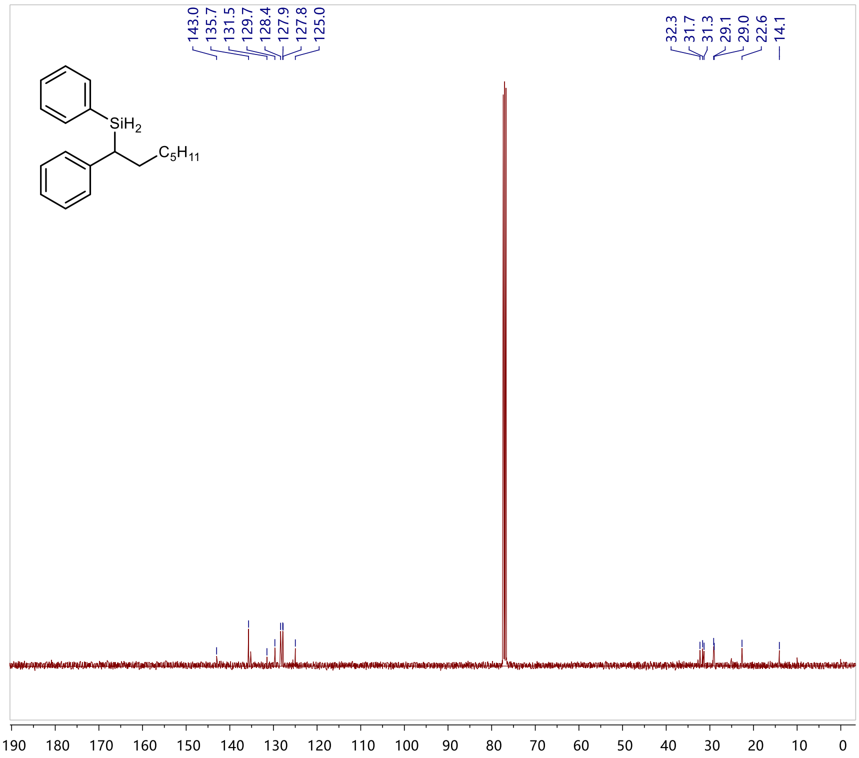

	Parameter	Value
1	Data File Name	Z:/ Data/ NMR/ 2017-03/ hmy-20170319-2/ 2/ fid
2	Title	hmy-20170319-2
3	Comment	C13CPD
4	Origin	Bruker BioSpin GmbH
5	Owner	nmr
6	Site	
7	Spectrometer	spect
8	Author	
9	Solvent	CDCl3
10	Temperature	293.6
11	Pulse Sequence	zgpg30
12	Experiment	1D
13	Number of Scans	512
14	Receiver Gain	36
15	Relaxation Delay	2.0000
16	Pulse Width	9.4000
17	Acquisition Time	1.3631
18	Acquisition Date	2017-03-20T01:04:49
19	Modification Date	2017-03-20T01:04:00
20	Spectrometer Frequency	100.61
21	Spectral Width	24038.5
22	Lowest Frequency	-1958.9
23	Nucleus	13C
24	Acquired Size	32768
25	Spectral Size	65536

Supplementary Figure 87 | ^{13}C NMR (101 MHz, $CDCl_3$) spectra for compound 12b.

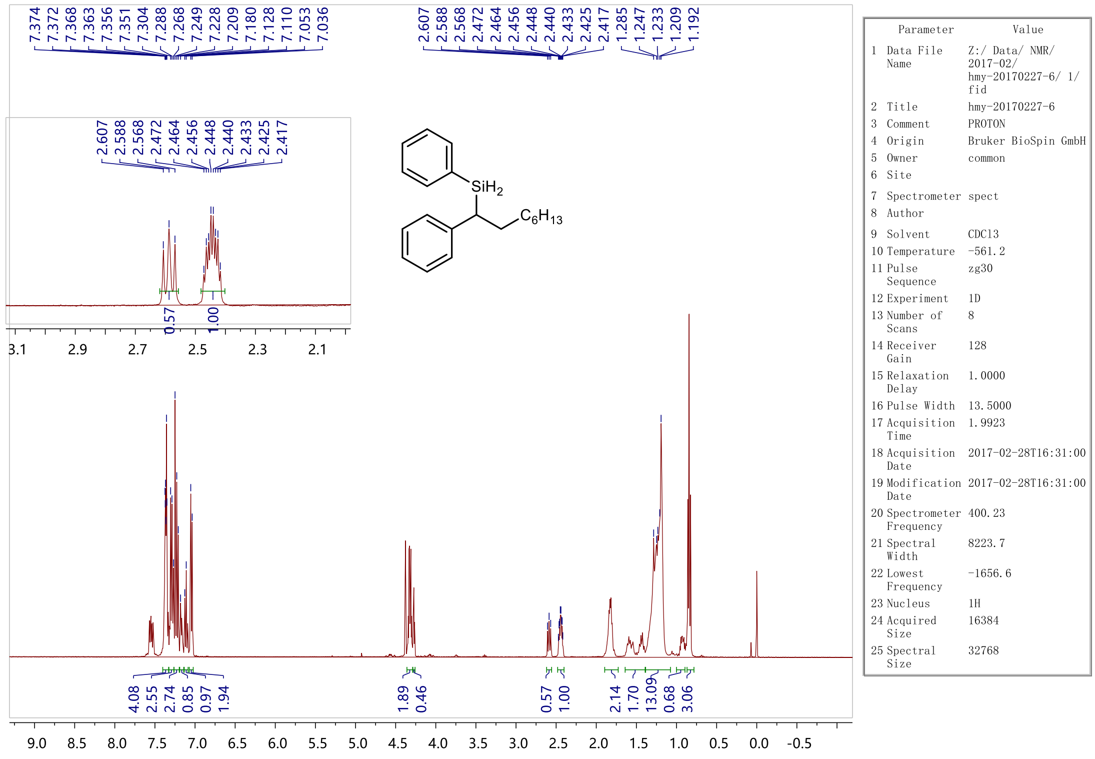

Supplementary Figure 88 | ^{1}H NMR (400 MHz, $CDCl_3$) spectra for compound 12c.

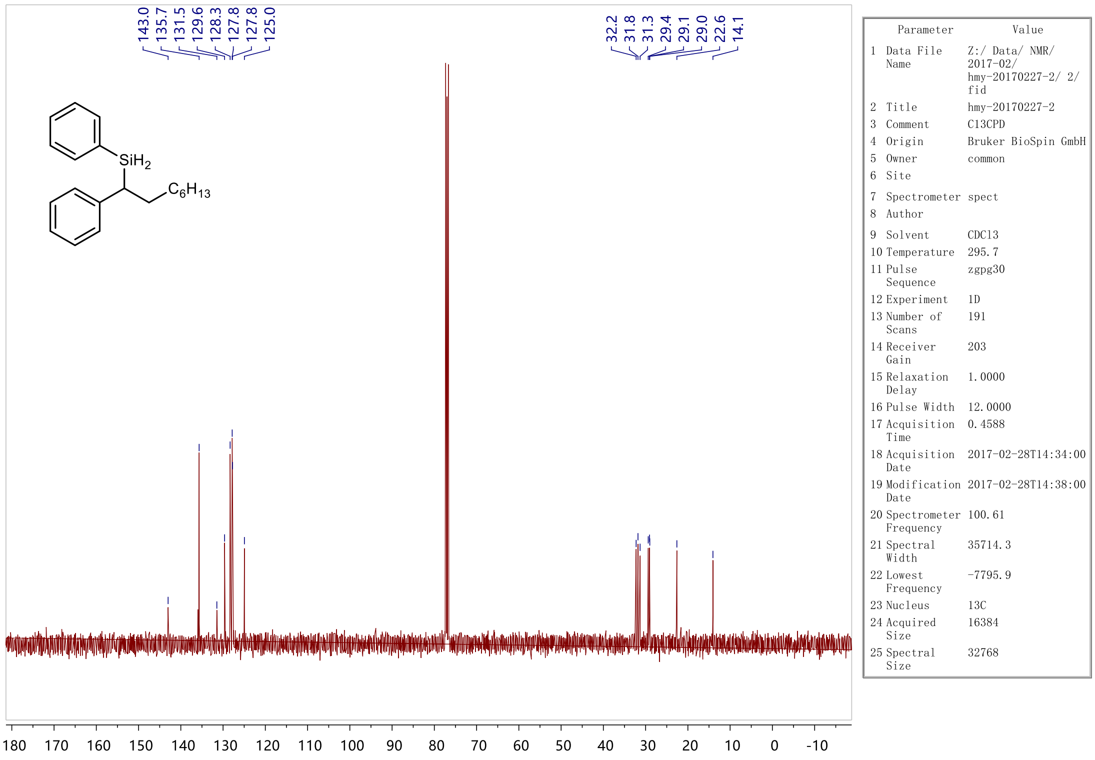

Supplementary Figure 89 | ^{13}C NMR (101 MHz, $CDCl_3$) spectra for compound 12c.

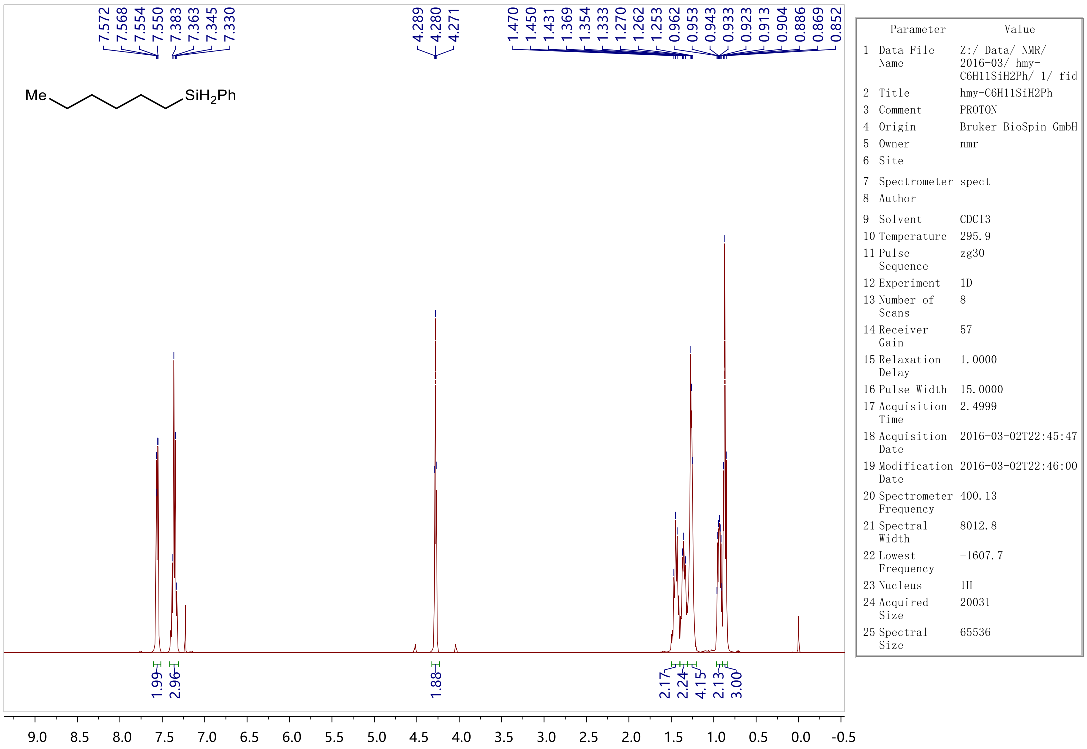

Supplementary Figure 90 | ^{1}H NMR (400 MHz, $CDCl_3$) spectra for compound 12d.

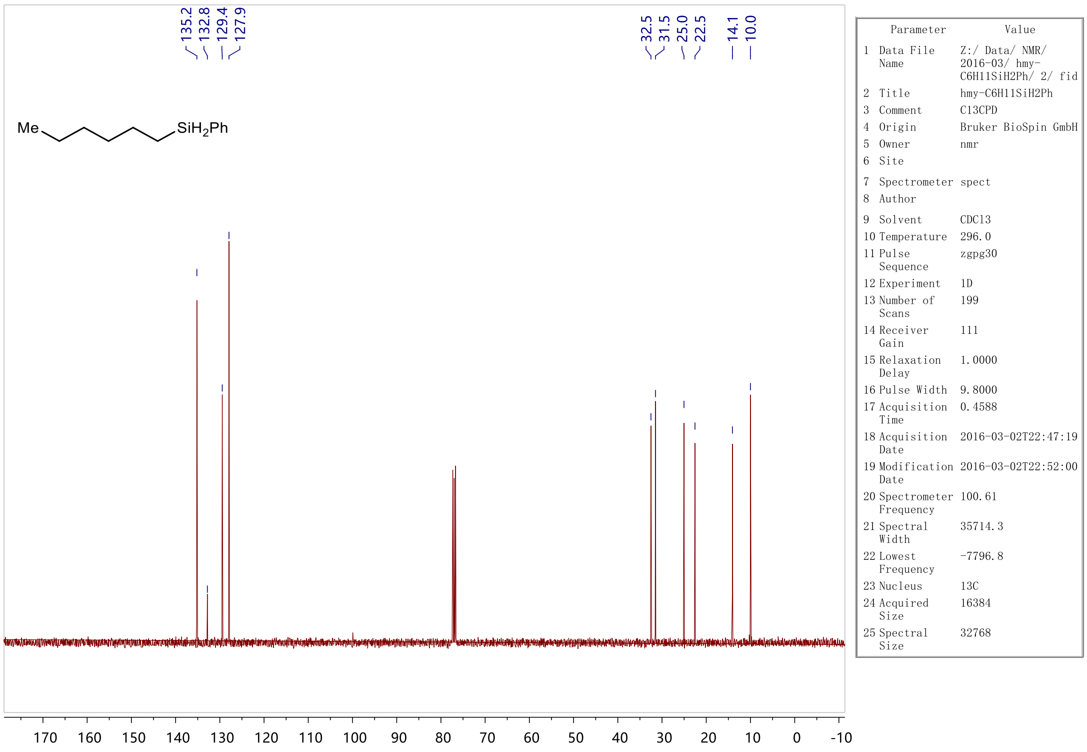

Supplementary Figure 91 | ^{13}C NMR (101 MHz, $CDCl_3$) spectra for compound 12d.

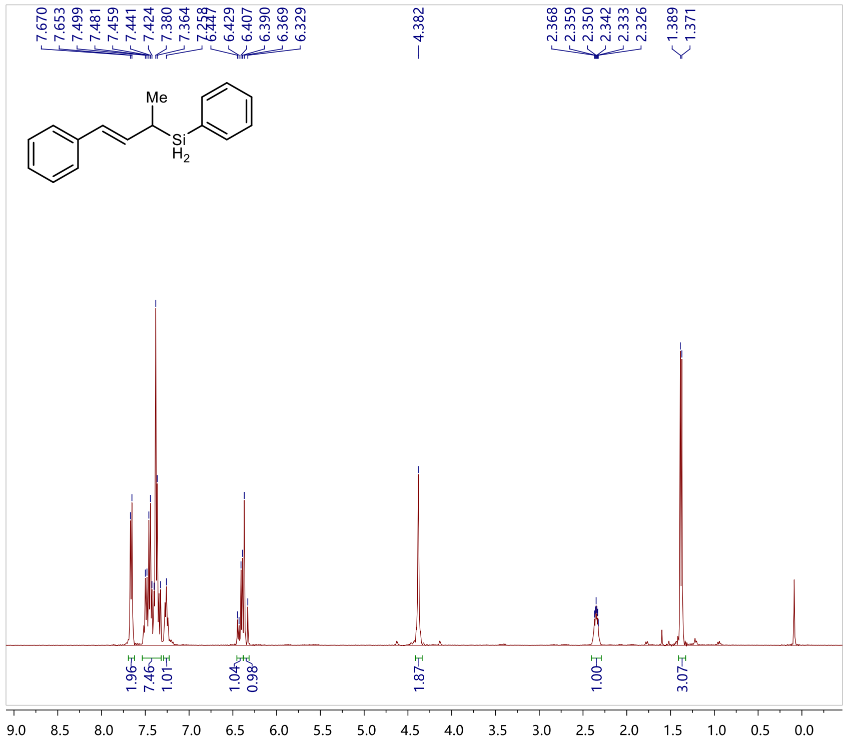

Parameter		Value
1	Data File Name	Z:/ Data/ NMR/ 2016-1/ hmy-diene1/ 1/ fid
2	Title	hmy-diene1
3	Comment	PROTON
4	Origin	Bruker BioSpin GmbH
5	Owner	common
6	Site	
7	Spectrometer	spect
8	Author	
9	Solvent	CDCl3
10	Temperature	2436.7
11	Pulse Sequence	zg30
12	Experiment	1D
13	Number of Scans	8
14	Receiver Gain	128
15	Relaxation Delay	1.0000
16	Pulse Width	13.7000
17	Acquisition Time	1.9999
18	Acquisition Date	2016-01-12T00:19:00
19	Modification Date	2016-01-12T00:19:00
20	Spectrometer Frequency	400.13
21	Spectral Width	8223.7
22	Lowest Frequency	-1640.9
23	Nucleus	1H
24	Acquired Size	16446
25	Spectral Size	65536

Supplementary Figure 92 | ^{1}H NMR (400 MHz, $CDCl_3$) spectra for compound 14a.

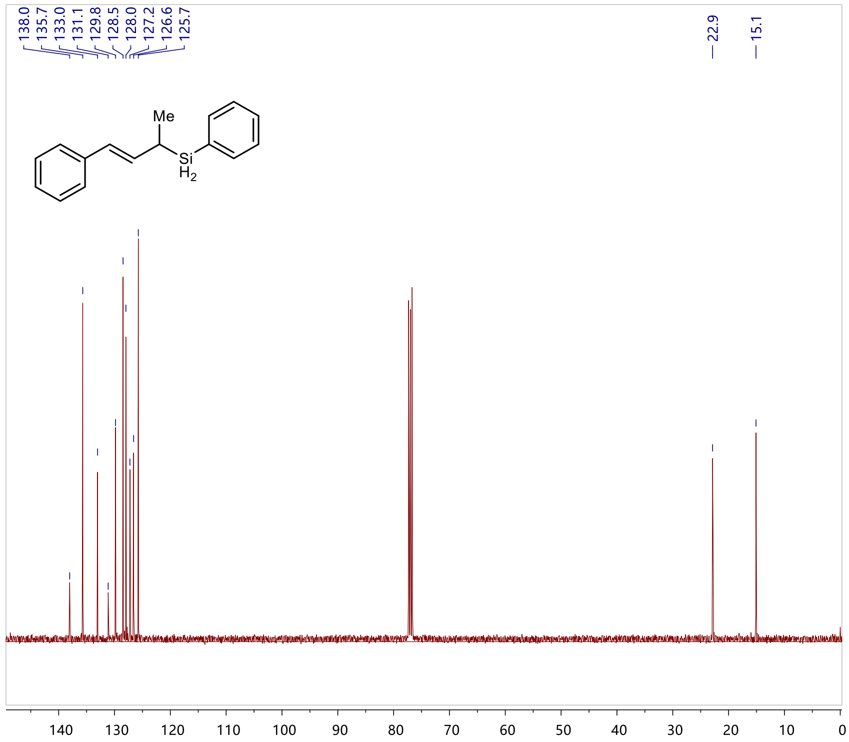

Parameter		Value
1	Data File Name	Z:/ Data/ NMR/ 2016-1/ hmy-diene1/ 2/ fid
2	Title	hmy-diene1
3	Comment	C13CPD
4	Origin	Bruker BioSpin GmbH
5	Owner	common
6	Site	
7	Spectrometer	spect
8	Author	
9	Solvent	CDCl3
10	Temperature	2540.9
11	Pulse Sequence	zgpg30
12	Experiment	1D
13	Number of Scans	933
14	Receiver Gain	203
15	Relaxation Delay	1.0000
16	Pulse Width	12.0000
17	Acquisition Time	0.4588
18	Acquisition Date	2016-01-12T00:44:00
19	Modification Date	2016-01-12T00:44:00
20	Spectrometer Frequency	100.61
21	Spectral Width	35714.3
22	Lowest Frequency	-7795.9
23	Nucleus	13C
24	Acquired Size	16384
25	Spectral Size	32768

Supplementary Figure 93 | ^{13}C NMR (101 MHz, $CDCl_3$) spectra for compound 14a.

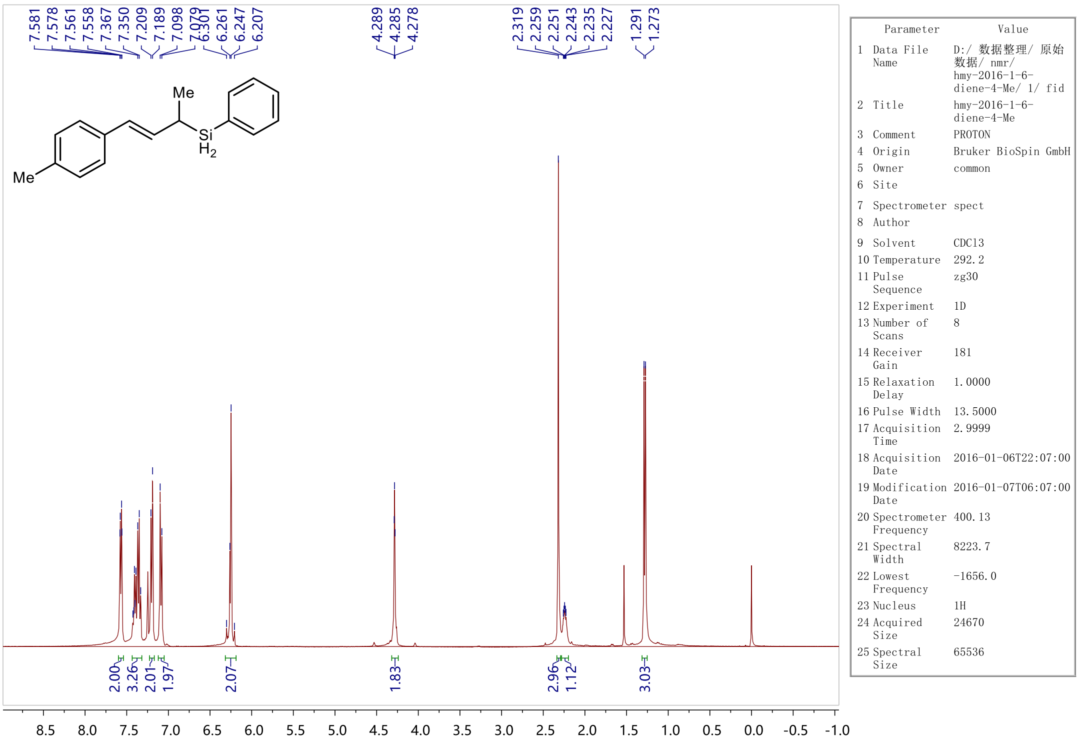

Parameter		Value
1	Data File Name	D:/ 数据整理/ 原始数据/ nmr/ hmy-2016-1-6-diene-4-Me/ 1/ fid
2	Title	hmy-2016-1-6-diene-4-Me
3	Comment	PROTON
4	Origin	Bruker BioSpin GmbH
5	Owner	common
6	Site	
7	Spectrometer	spect
8	Author	
9	Solvent	CDC13
10	Temperature	292.2
11	Pulse Sequence	zg30
12	Experiment	1D
13	Number of Scans	8
14	Receiver Gain	181
15	Relaxation Delay	1.0000
16	Pulse Width	13.5000
17	Acquisition Time	2.9999
18	Acquisition Date	2016-01-06T22:07:00
19	Modification Date	2016-01-07T06:07:00
20	Spectrometer Frequency	400.13
21	Spectral Width	8223.7
22	Lowest Frequency	-1656.0
23	Nucleus	1H
24	Acquired Size	24670
25	Spectral Size	65536

Supplementary Figure 94 | ^{1}H NMR (400 MHz, $CDCl_3$) spectra for compound 14b.

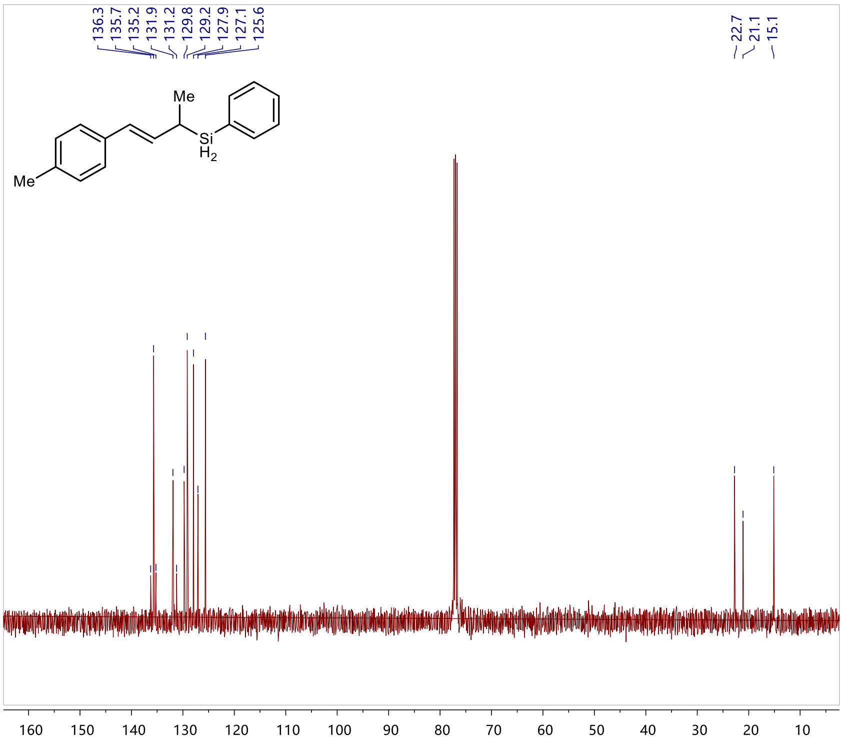

Parameter		Value
1	Data File Name	D:/ 数据整理/ 原始数据/ nmr/ hmy-2016-1-6-diene-4-Me/ 2/ fid
2	Title	hmy-2016-1-6-diene-4-Me
3	Comment	C13CPD
4	Origin	Bruker BioSpin GmbH
5	Owner	common
6	Site	
7	Spectrometer	spect
8	Author	
9	Solvent	CDC13
10	Temperature	292.6
11	Pulse Sequence	zgpg30
12	Experiment	1D
13	Number of Scans	202
14	Receiver Gain	203
15	Relaxation Delay	1.0000
16	Pulse Width	12.5000
17	Acquisition Time	0.5506
18	Acquisition Date	2016-01-06T22:10:00
19	Modification Date	2016-01-07T06:15:00
20	Spectrometer Frequency	100.61
21	Spectral Width	29761.9
22	Lowest Frequency	-4820.2
23	Nucleus	13C
24	Acquired Size	16384
25	Spectral Size	32768

Supplementary Figure 95 | ^{13}C NMR (101 MHz, $CDCl_3$) spectra for compound 14b.

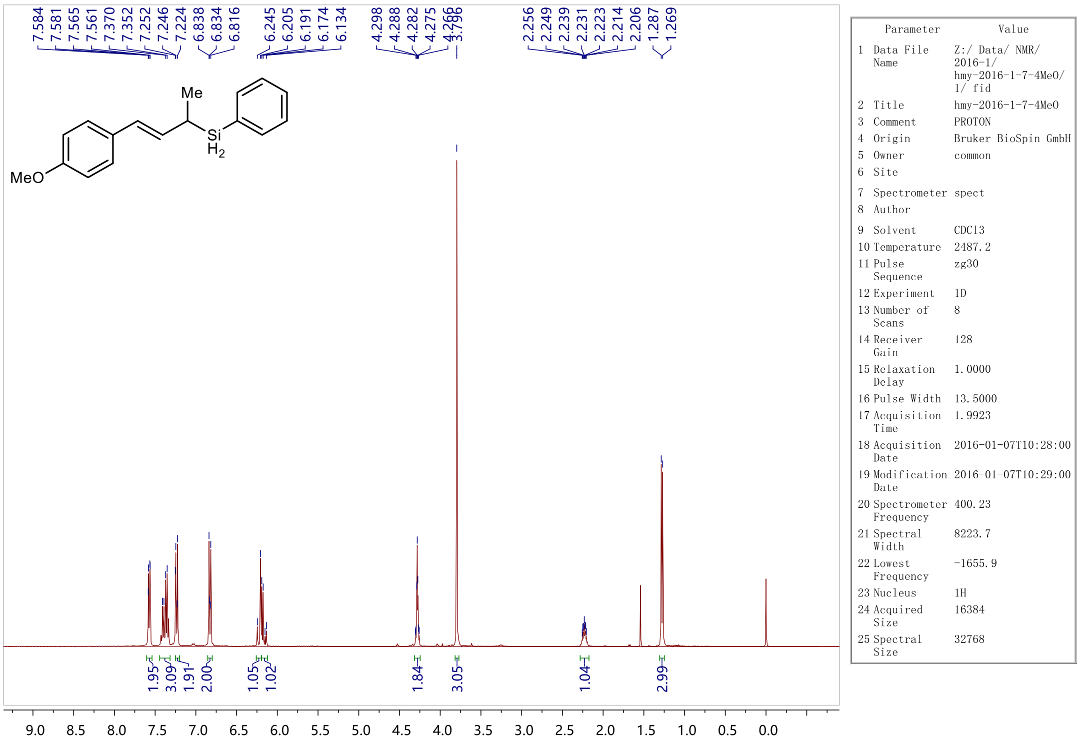

Supplementary Figure 96 | ^{1}H NMR (400 MHz, $CDCl_3$) spectra for compound 14c.

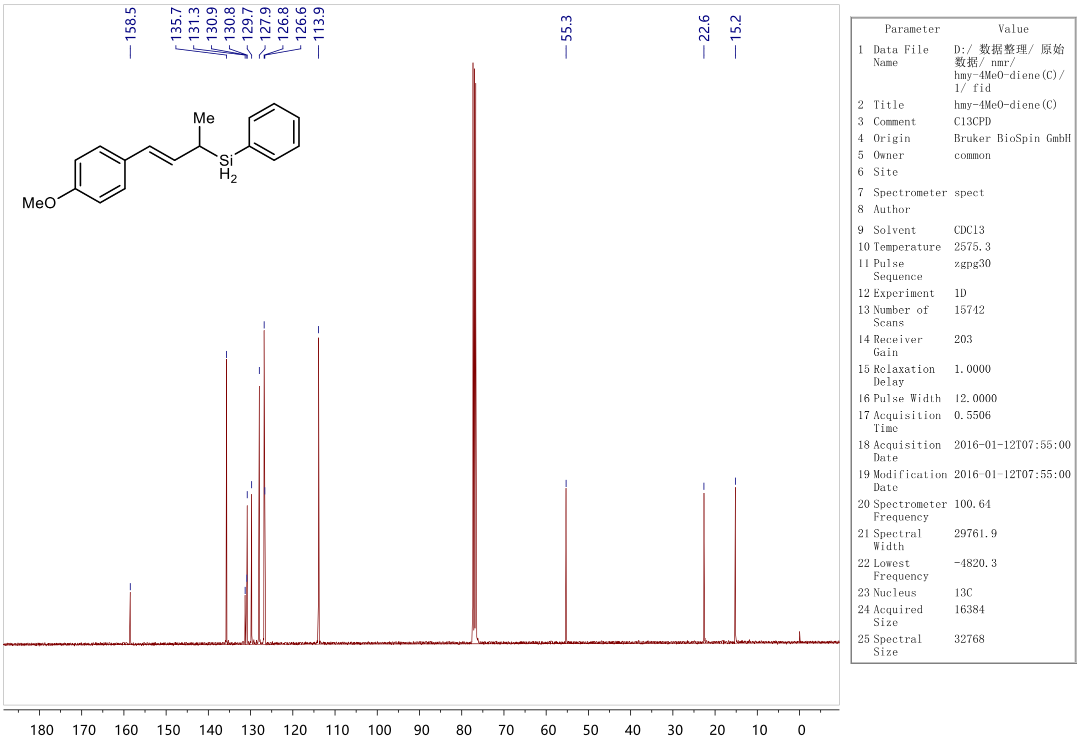

Supplementary Figure 97 | ^{13}C NMR (101 MHz, $CDCl_3$) spectra for compound 14c.

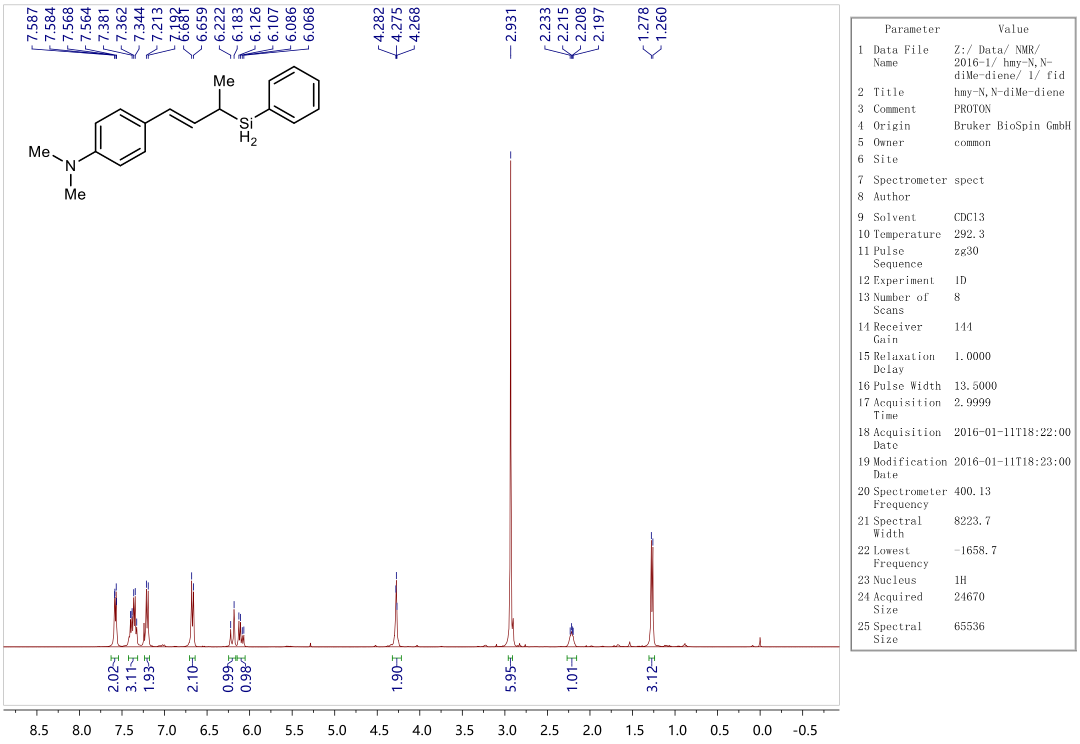

Supplementary Figure 98 | ^{1}H NMR (400 MHz, $CDCl_3$) spectra for compound 14d.

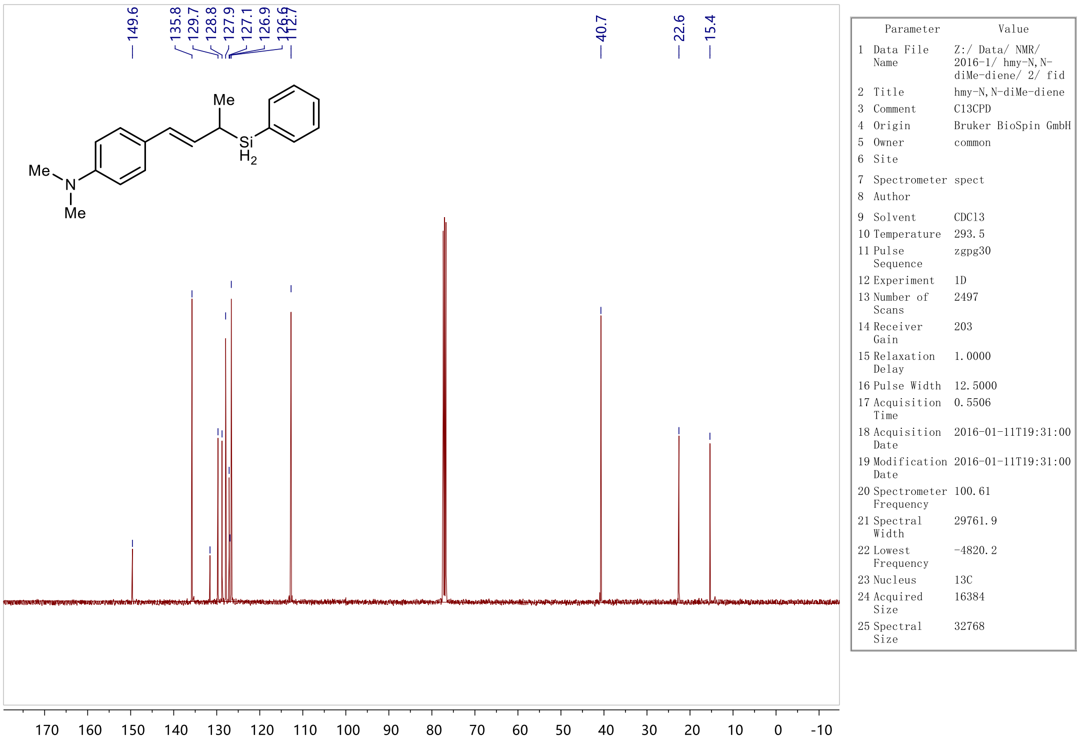

Supplementary Figure 99 | ^{13}C NMR (101 MHz, $CDCl_3$) spectra for compound 14d.

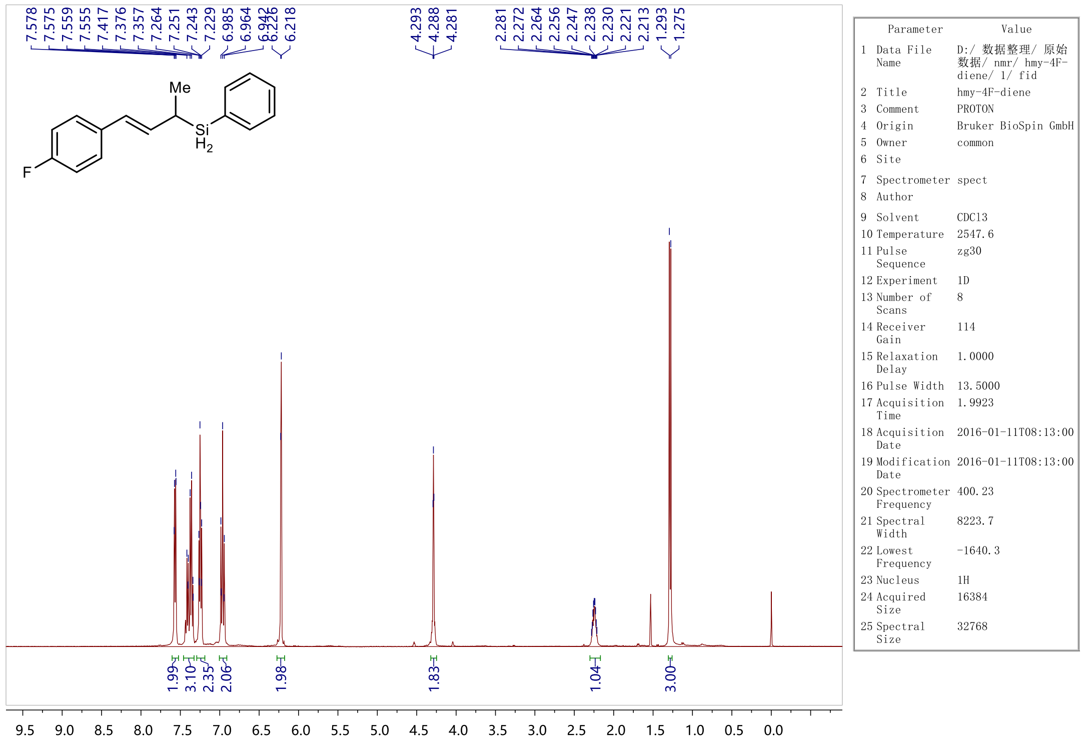

Supplementary Figure 100 | ^{1}H NMR (400 MHz, $CDCl_3$) spectra for compound 14e.

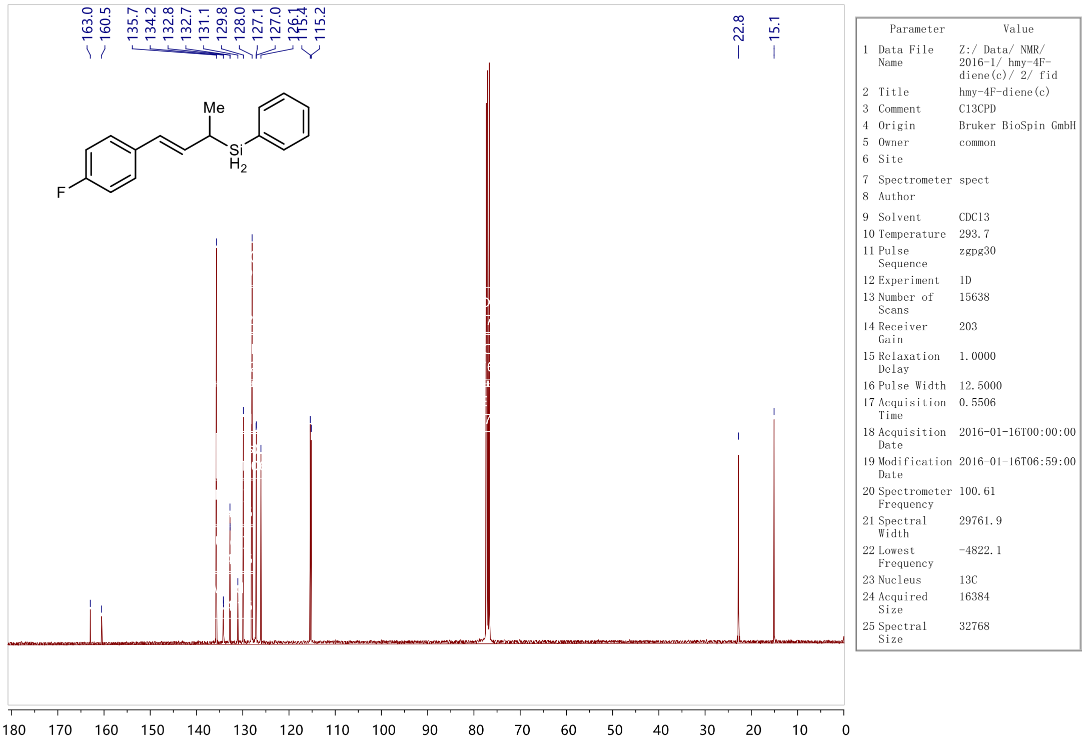

Supplementary Figure 101 | ^{13}C NMR (101 MHz, $CDCl_3$) spectra for compound 14e.

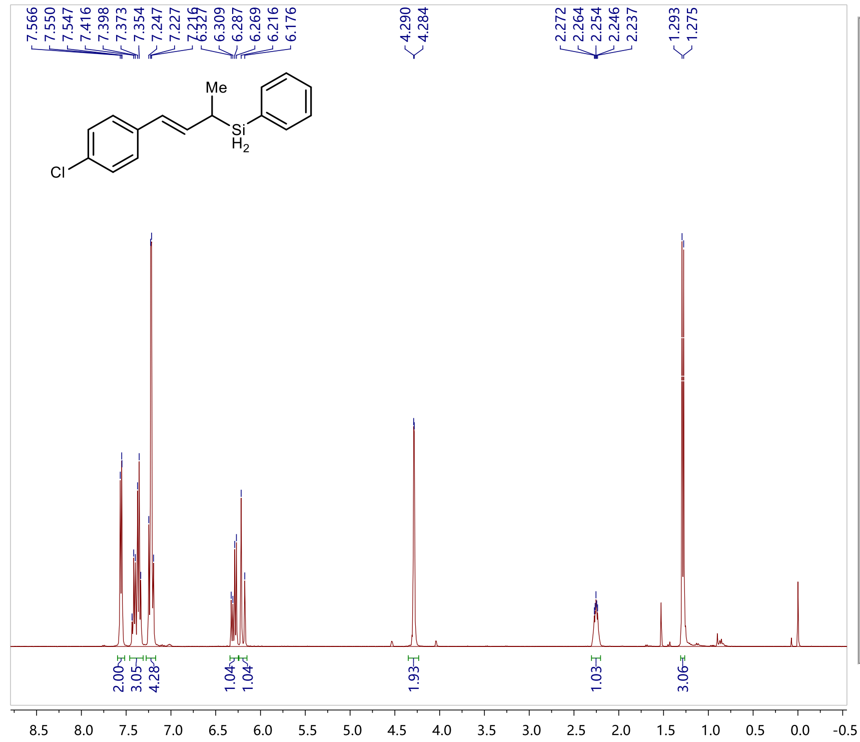

	Parameter	Value
1	Data File Name	Z:/ Data/ NMR/ 2016-1/ hmy-2016-1-8-4Cl/ 1/ fid
2	Title	hmy-2016-1-8-4Cl
3	Comment	PROTON
4	Origin	Bruker BioSpin GmbH
5	Owner	nmr
6	Site	
7	Spectrometer	spect
8	Author	
9	Solvent	CDCl3
10	Temperature	295.3
11	Pulse Sequence	zg30
12	Experiment	1D
13	Number of Scans	8
14	Receiver Gain	133
15	Relaxation Delay	1.0000
16	Pulse Width	15.0000
17	Acquisition Time	2.4999
18	Acquisition Date	2016-01-08T15:16:30
19	Modification Date	2016-01-08T15:16:00
20	Spectrometer Frequency	400.13
21	Spectral Width	8012.8
22	Lowest Frequency	-1541.5
23	Nucleus	1H
24	Acquired Size	20031
25	Spectral Size	65536

Supplementary Figure 102 | ^{1}H NMR (400 MHz, $CDCl_3$) spectra for compound 14f.

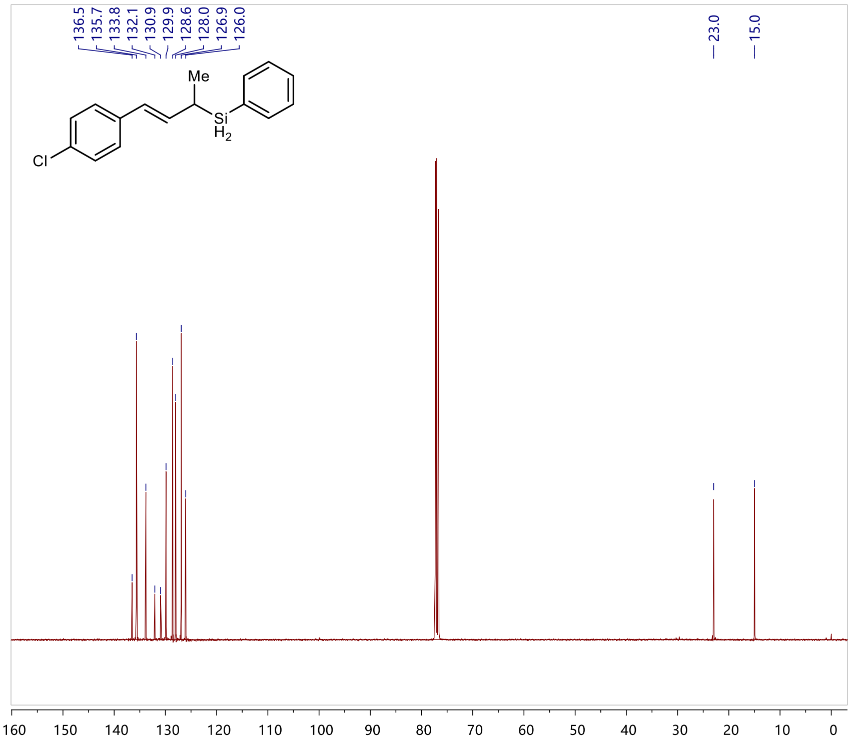

	Parameter	Value
1	Data File Name	Z:/ Data/ NMR/ 2016-1/ hmy-4Cl-diene(C)/ 1/ fid
2	Title	hmy-4Cl-diene(C)
3	Comment	C13CPD
4	Origin	Bruker BioSpin GmbH
5	Owner	nmr
6	Site	
7	Spectrometer	spect
8	Author	
9	Solvent	CDCl3
10	Temperature	296.7
11	Pulse Sequence	zgpg30
12	Experiment	1D
13	Number of Scans	15389
14	Receiver Gain	57
15	Relaxation Delay	1.0000
16	Pulse Width	9.8000
17	Acquisition Time	0.4588
18	Acquisition Date	2016-01-14T01:34:16
19	Modification Date	2016-01-14T07:58:00
20	Spectrometer Frequency	100.61
21	Spectral Width	35714.3
22	Lowest Frequency	-7796.8
23	Nucleus	13C
24	Acquired Size	16384
25	Spectral Size	32768

Supplementary Figure 103 | ^{13}C NMR (101 MHz, $CDCl_3$) spectra for compound 14f.

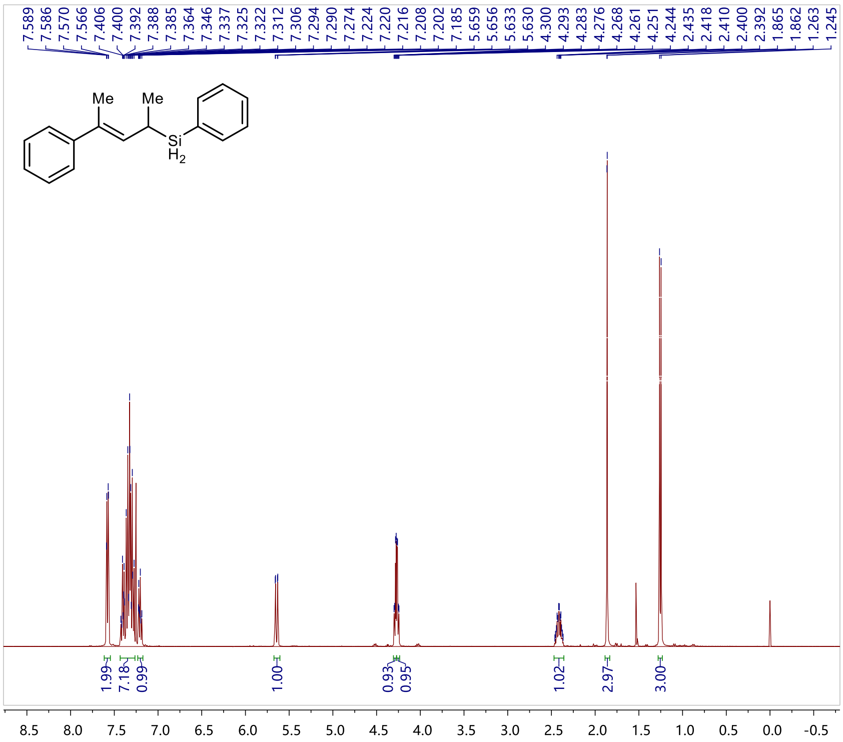

	Parameter	Value
1	Data File Name	Z:/ Data/ NMR/ 2016-1/ hmy-8g-1/ 1/ fid
2	Title	hmy-8g-1
3	Comment	PROTON
4	Origin	Bruker BioSpin GmbH
5	Owner	nmr
6	Site	
7	Spectrometer	spect
8	Author	
9	Solvent	CDCl3
10	Temperature	294.1
11	Pulse Sequence	zg30
12	Experiment	1D
13	Number of Scans	8
14	Receiver Gain	126
15	Relaxation Delay	1.0000
16	Pulse Width	9.1200
17	Acquisition Time	4.0894
18	Acquisition Date	2016-01-26T07:31:42
19	Modification Date	2016-01-26T07:32:00
20	Spectrometer Frequency	400.13
21	Spectral Width	8012.8
22	Lowest Frequency	-1548.4
23	Nucleus	1H
24	Acquired Size	32768
25	Spectral Size	65536

Supplementary Figure 104 | ^{1}H NMR (400 MHz, $CDCl_3$) spectra for compound 14g.

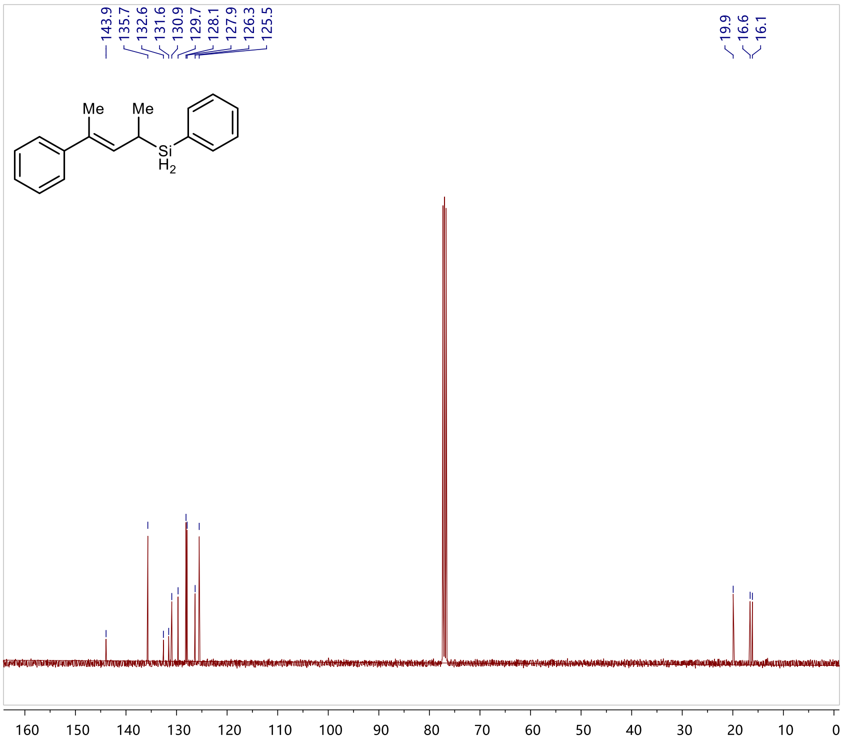

	Parameter	Value
1	Data File Name	Z:/ Data/ NMR/ 2016-1/ hmy-8g-1/ 2/ fid
2	Title	hmy-8g-1
3	Comment	C13CPD
4	Origin	Bruker BioSpin GmbH
5	Owner	nmr
6	Site	
7	Spectrometer	spect
8	Author	
9	Solvent	CDCl3
10	Temperature	294.3
11	Pulse Sequence	zgpg30
12	Experiment	1D
13	Number of Scans	353
14	Receiver Gain	29
15	Relaxation Delay	2.0000
16	Pulse Width	9.4000
17	Acquisition Time	1.3631
18	Acquisition Date	2016-01-26T07:33:16
19	Modification Date	2016-01-26T07:53:00
20	Spectrometer Frequency	100.61
21	Spectral Width	24038.5
22	Lowest Frequency	-1958.9
23	Nucleus	13C
24	Acquired Size	32768
25	Spectral Size	65536

Supplementary Figure 105 | ^{13}C NMR (101 MHz, $CDCl_3$) spectra for compound 14g.

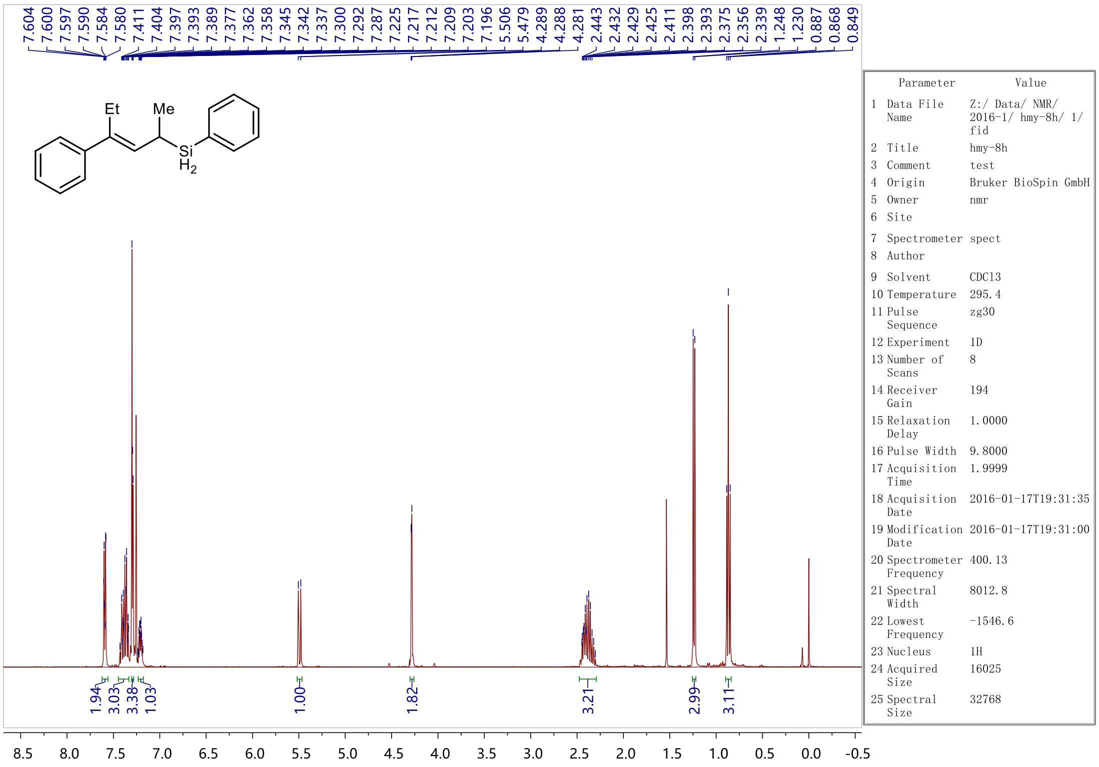

Supplementary Figure 106 | ^{1}H NMR (400 MHz, $CDCl_3$) spectra for compound 14h.

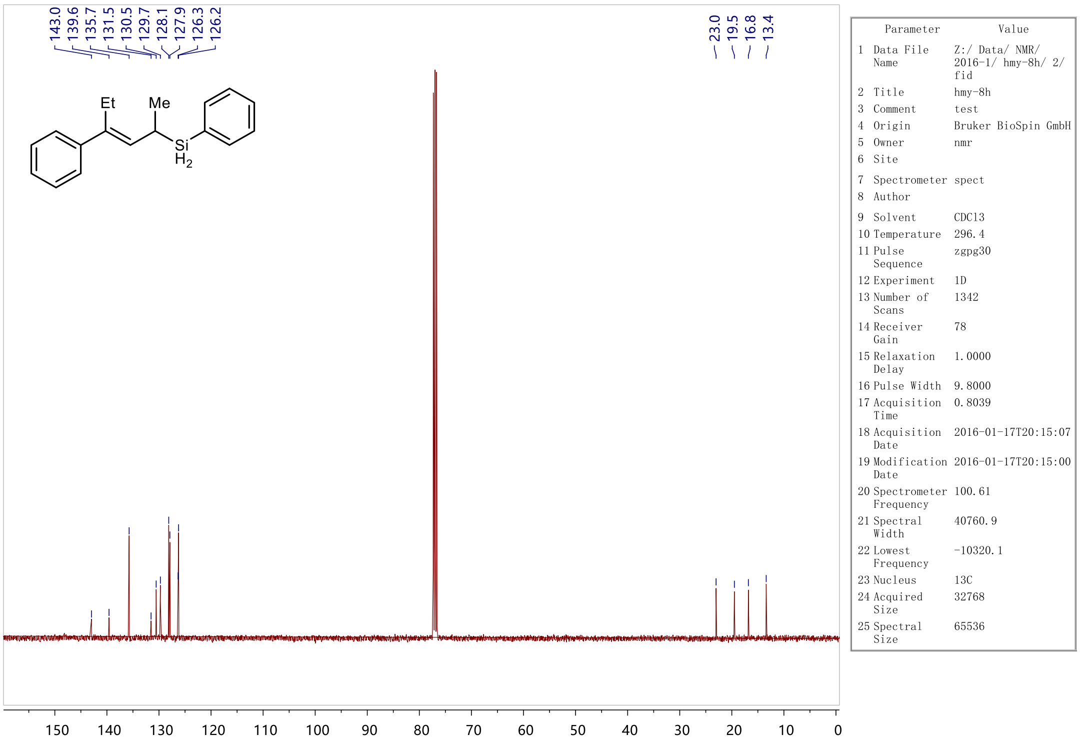

Supplementary Figure 107 | ^{13}C NMR (101 MHz, $CDCl_3$) spectra for compound 14h.

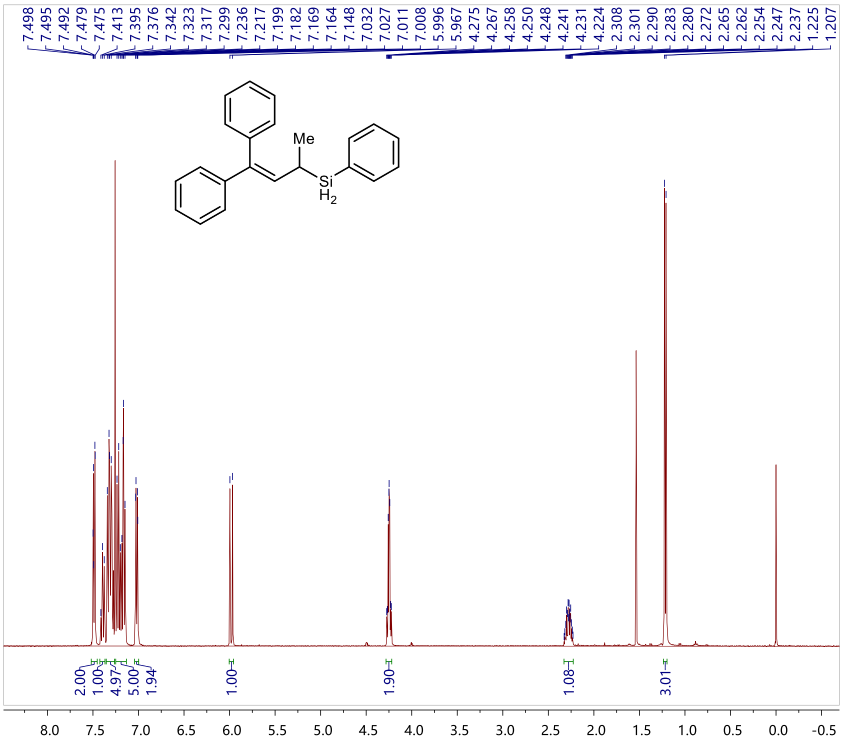

Parameter		Value
1	Data File Name	Z:/ Data/ NMR/ 2016-1/ hmy-8i/ 1/ fid
2	Title	hmy-8i
3	Comment	test
4	Origin	Bruker BioSpin GmbH
5	Owner	nmr
6	Site	
7	Spectrometer	spect
8	Author	
9	Solvent	CDCl3
10	Temperature	295.4
11	Pulse Sequence	zg30
12	Experiment	1D
13	Number of Scans	8
14	Receiver Gain	194
15	Relaxation Delay	1.0000
16	Pulse Width	9.8000
17	Acquisition Time	1.9999
18	Acquisition Date	2016-01-17T20:48:37
19	Modification Date	2016-01-17T20:48:00
20	Spectrometer Frequency	400.13
21	Spectral Width	8012.8
22	Lowest Frequency	-1535.4
23	Nucleus	1H
24	Acquired Size	16025
25	Spectral Size	32768

Supplementary Figure 108 | ^{1}H NMR (400 MHz, $CDCl_3$) spectra for compound 14i.

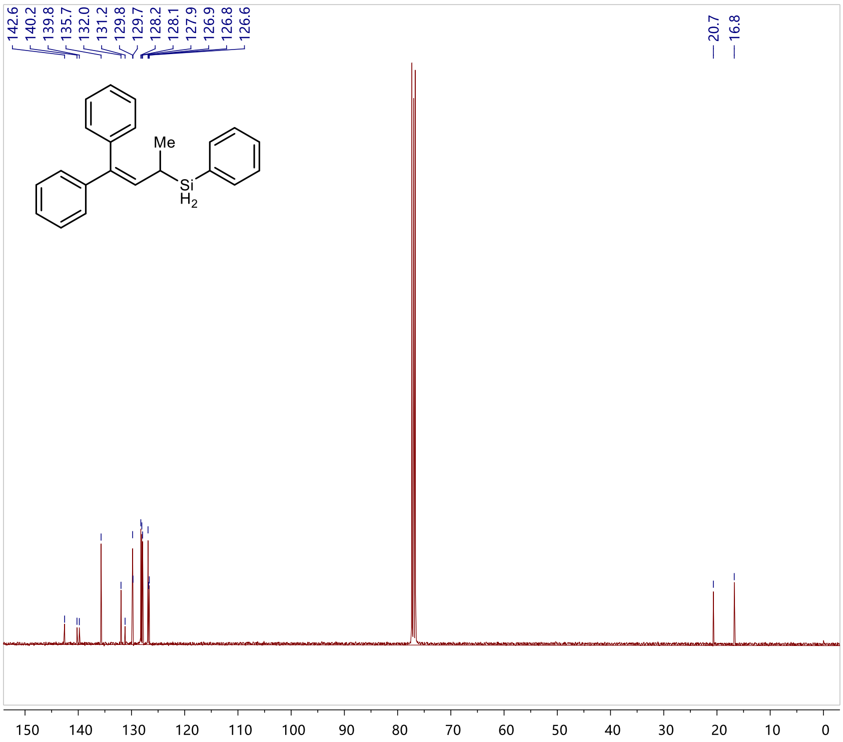

Parameter		Value
1	Data File Name	C:/ Users/ HMY/ Desktop/ hmy-8i/ 1/ fid
2	Title	hmy-8i
3	Comment	C13CPD
4	Origin	Bruker BioSpin GmbH
5	Owner	common
6	Site	
7	Spectrometer	spect
8	Author	
9	Solvent	CDCl3
10	Temperature	298.2
11	Pulse Sequence	zgpg30
12	Experiment	1D
13	Number of Scans	10000
14	Receiver Gain	203
15	Relaxation Delay	1.0000
16	Pulse Width	12.0000
17	Acquisition Time	0.4588
18	Acquisition Date	2016-01-18T04:19:00
19	Modification Date	2016-01-18T04:19:00
20	Spectrometer Frequency	100.61
21	Spectral Width	35714.3
22	Lowest Frequency	-7725.4
23	Nucleus	13C
24	Acquired Size	16384
25	Spectral Size	32768

Supplementary Figure 109 | ^{13}C NMR (101 MHz, $CDCl_3$) spectra for compound 14i.

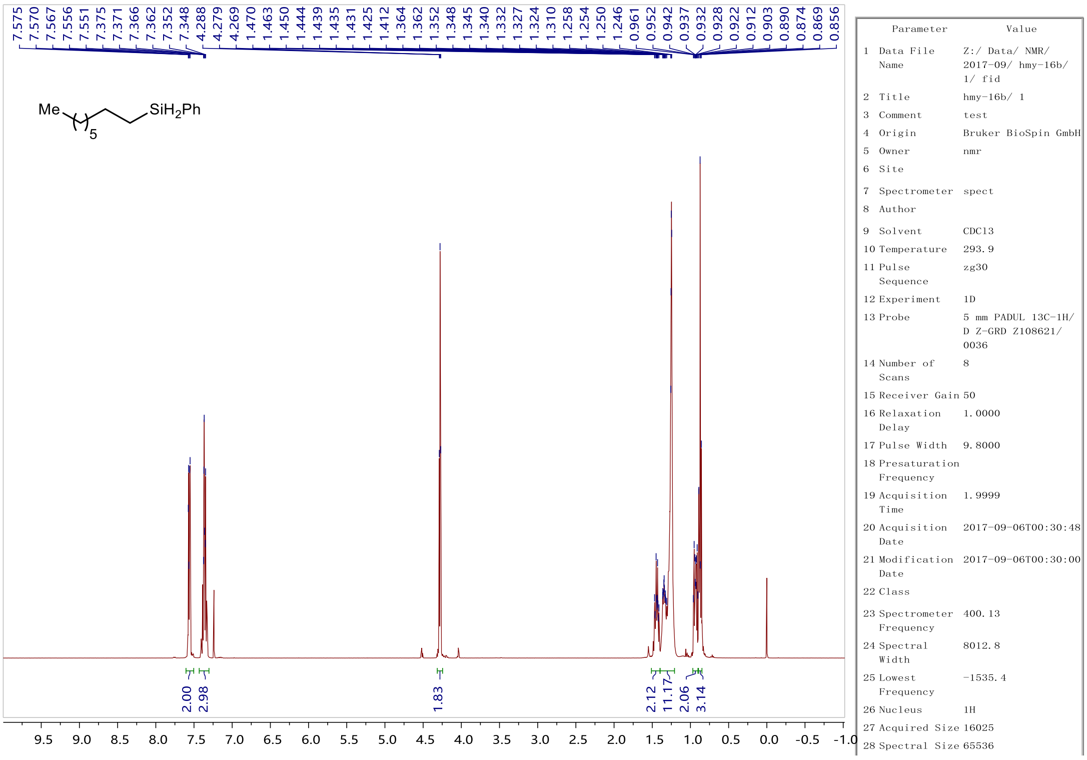

Supplementary Figure 110 | ^{1}H NMR (400 MHz, CDCl$_3$) spectra for compound 16b.

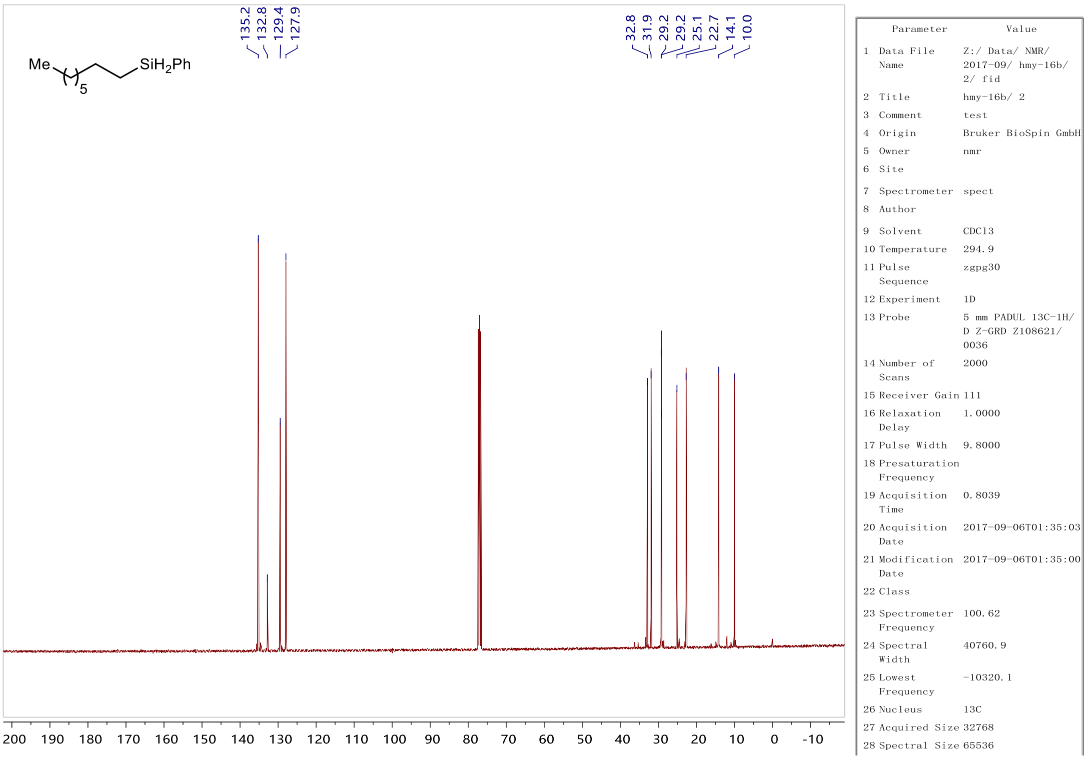

Supplementary Figure 111 | ^{13}C NMR (101 MHz, CDCl$_3$) spectra for compound 16b.

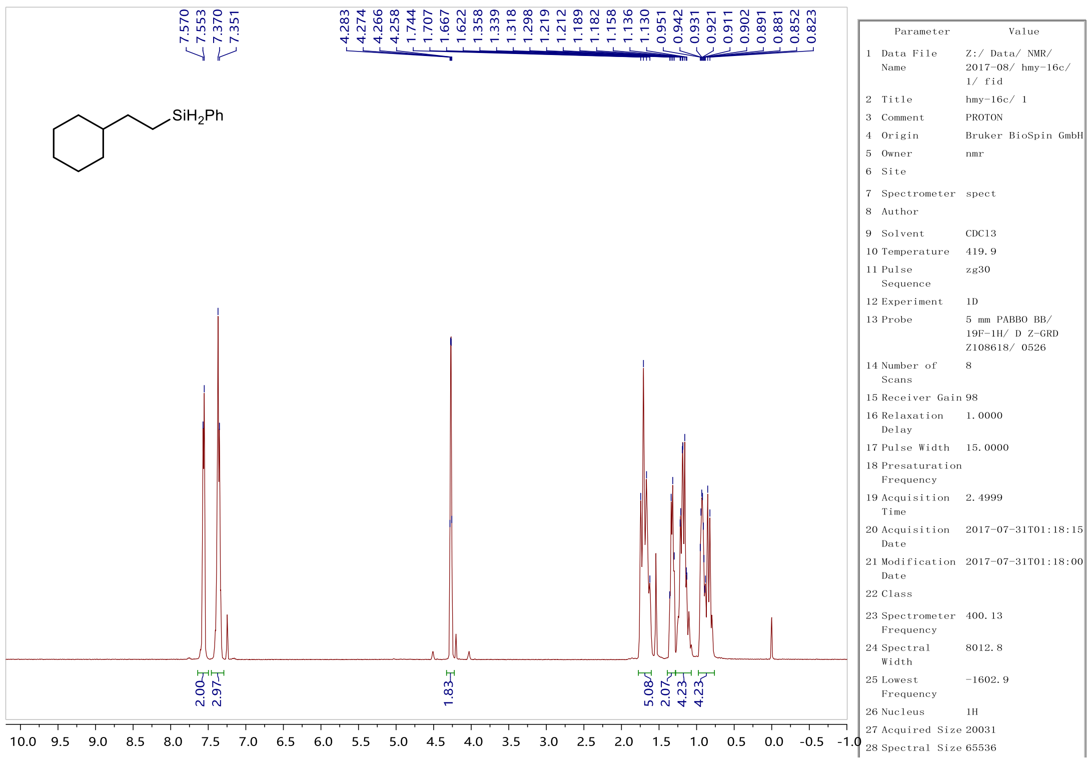

Supplementary Figure 112 | ^{1}H NMR (400 MHz, $CDCl_3$) spectra for compound 16c.

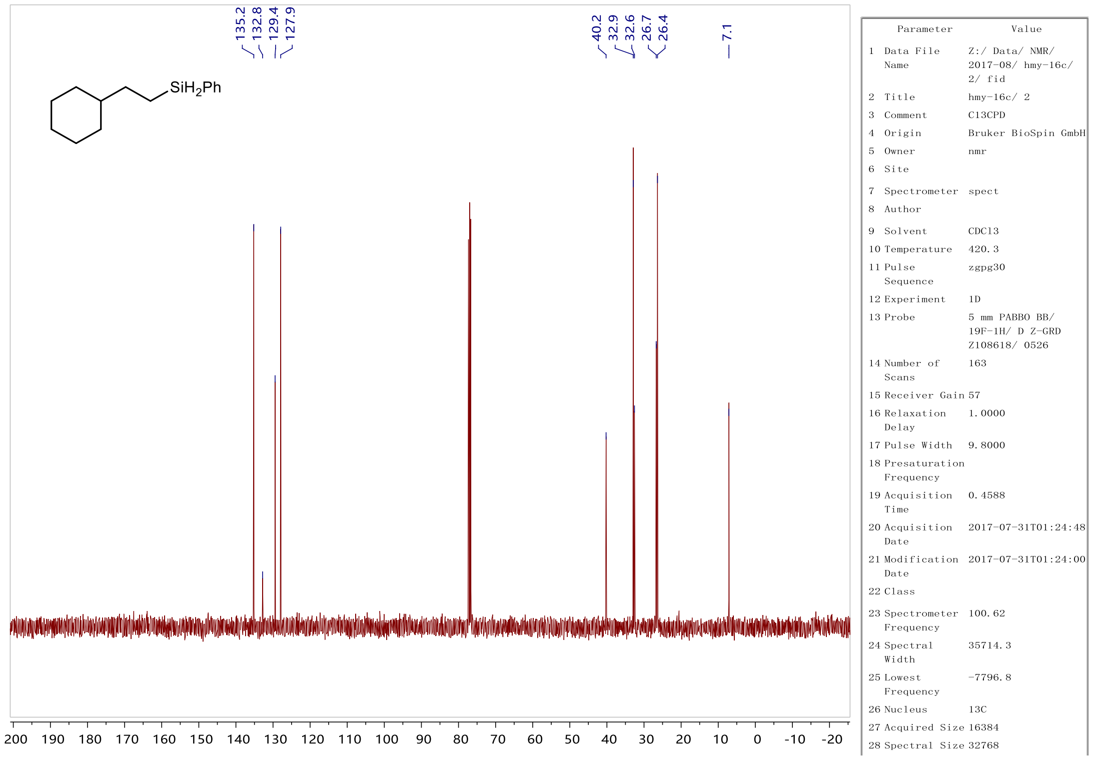

Supplementary Figure 113 | ^{13}C NMR (101 MHz, $CDCl_3$) spectra for compound 16c.

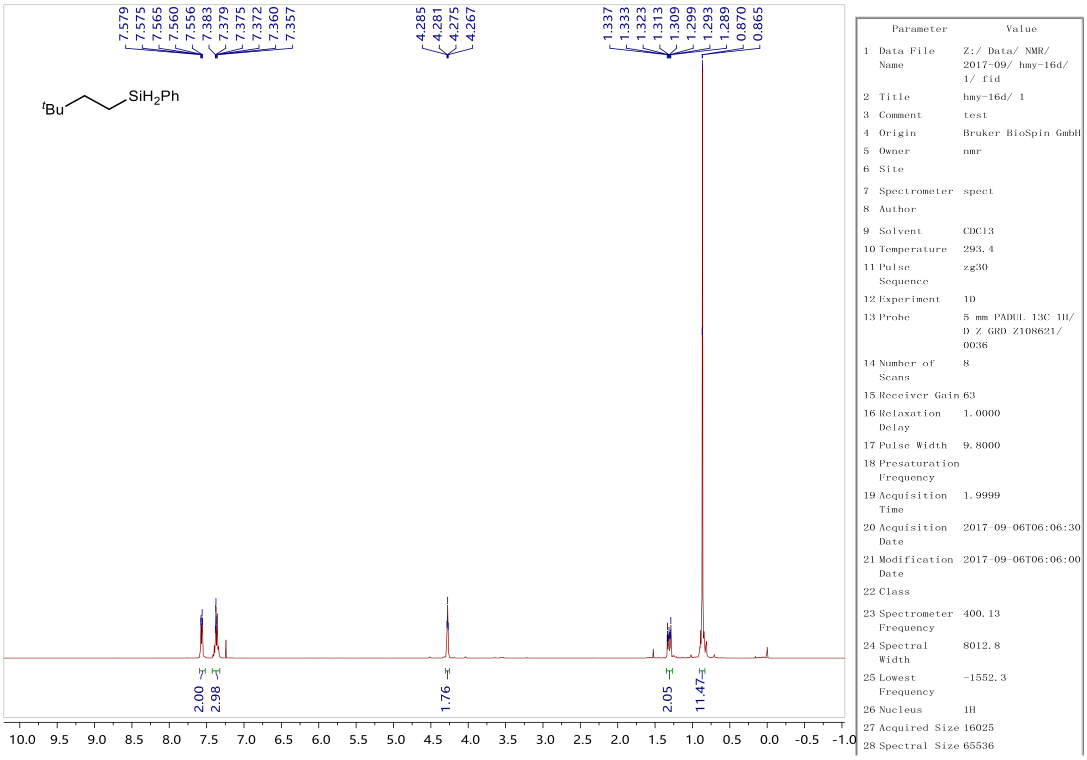

	Parameter	Value
1	Data File Name	Z:/ Data/ NMR/ 2017-09/ hmy-16d/ 1/ fid
2	Title	hmy-16d/ 1
3	Comment	test
4	Origin	Bruker BioSpin GmbH
5	Owner	nmr
6	Site	
7	Spectrometer	spect
8	Author	
9	Solvent	CDCl3
10	Temperature	293.4
11	Pulse Sequence	zg30
12	Experiment	1D
13	Probe	5 mm PADUL 13C-1H/ D Z-GRD Z108621/ 0036
14	Number of Scans	8
15	Receiver Gain	63
16	Relaxation Delay	1.0000
17	Pulse Width	9.8000
18	Presaturation Frequency	
19	Acquisition Time	1.9999
20	Acquisition Date	2017-09-06T06:06:30
21	Modification Date	2017-09-06T06:06:00
22	Class	
23	Spectrometer Frequency	400.13
24	Spectral Width	8012.8
25	Lowest Frequency	-1552.3
26	Nucleus	1H
27	Acquired Size	16025
28	Spectral Size	65536

Supplementary Figure 114 | ^{1}H NMR (400 MHz, $CDCl_3$) spectra for compound 16d.

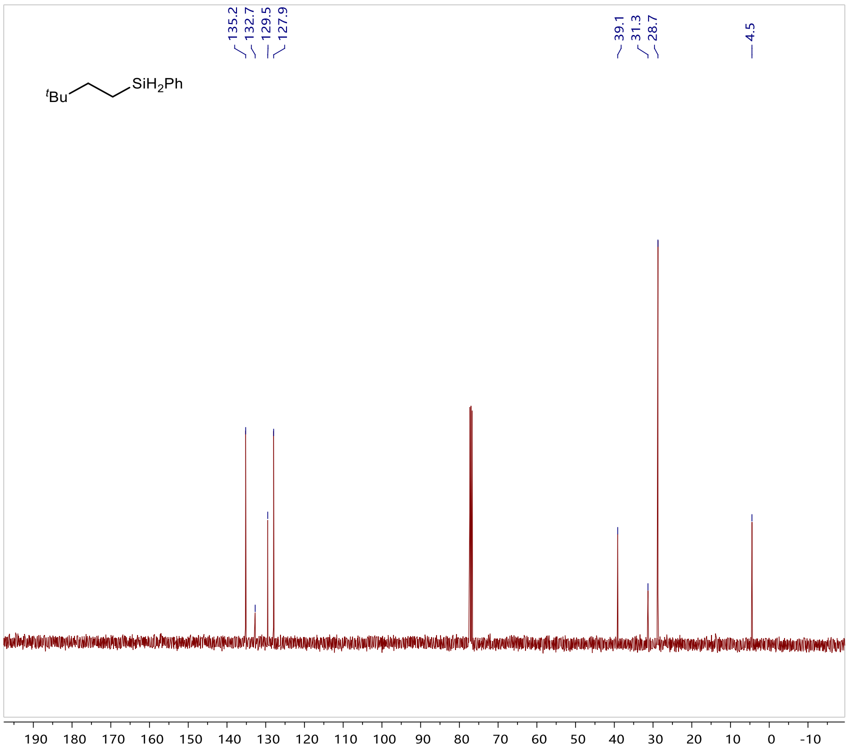

	Parameter	Value
1	Data File Name	Z:/ Data/ NMR/ 2017-09/ hmy-16d/ 2/ fid
2	Title	hmy-16d/ 2
3	Comment	test
4	Origin	Bruker BioSpin GmbH
5	Owner	nmr
6	Site	
7	Spectrometer	spect
8	Author	
9	Solvent	CDCl3
10	Temperature	293.8
11	Pulse Sequence	zgpg30
12	Experiment	1D
13	Probe	5 mm PADUL 13C-1H/ D Z-GRD Z108621/ 0036
14	Number of Scans	31
15	Receiver Gain	87
16	Relaxation Delay	1.0000
17	Pulse Width	9.8000
18	Presaturation Frequency	
19	Acquisition Time	0.8039
20	Acquisition Date	2017-09-06T06:08:08
21	Modification Date	2017-09-06T06:08:00
22	Class	
23	Spectrometer Frequency	100.62
24	Spectral Width	40760.9
25	Lowest Frequency	-10320.1
26	Nucleus	13C
27	Acquired Size	32768
28	Spectral Size	65536

Supplementary Figure 115 | ^{13}C NMR (101 MHz, $CDCl_3$) spectra for compound 16d.

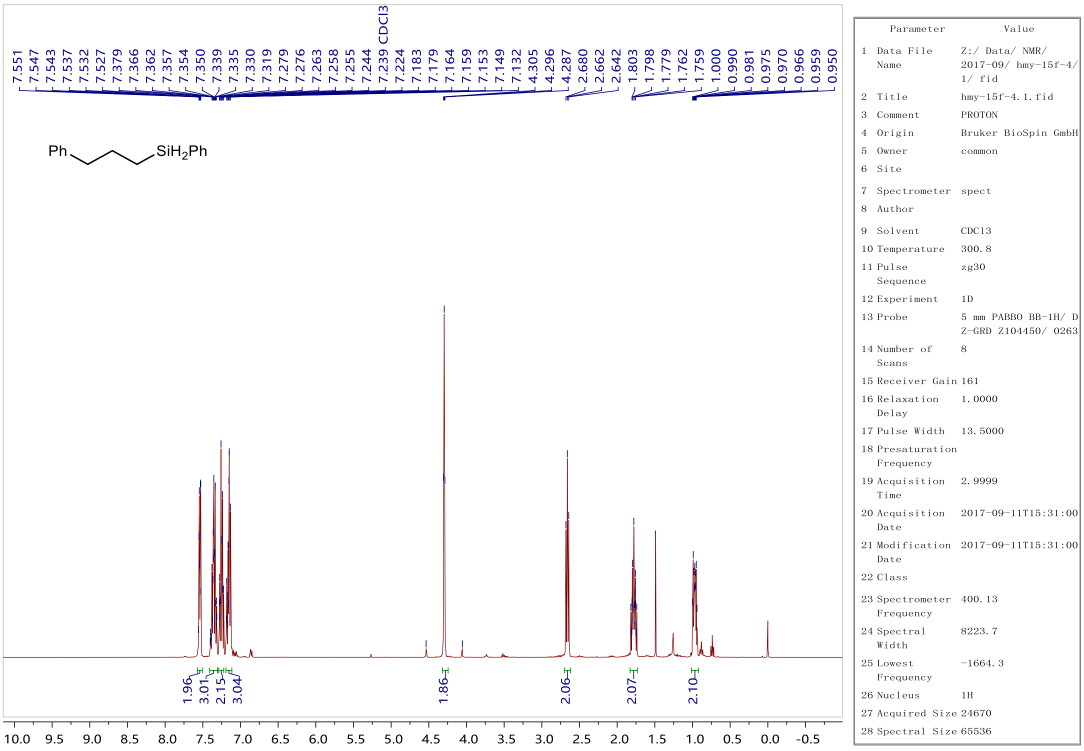

Supplementary Figure 116 | ^{1}H NMR (400 MHz, $CDCl_3$) spectra for compound 16e.

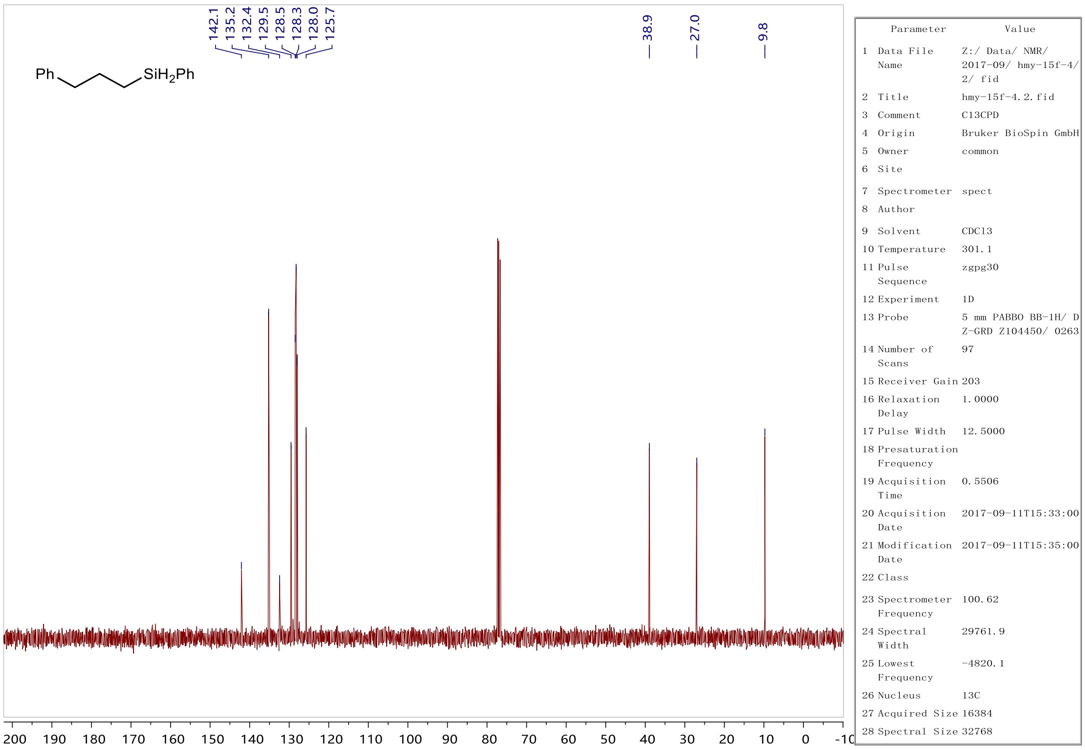

Supplementary Figure 117 | ^{13}C NMR (101 MHz, $CDCl_3$) spectra for compound 16e.

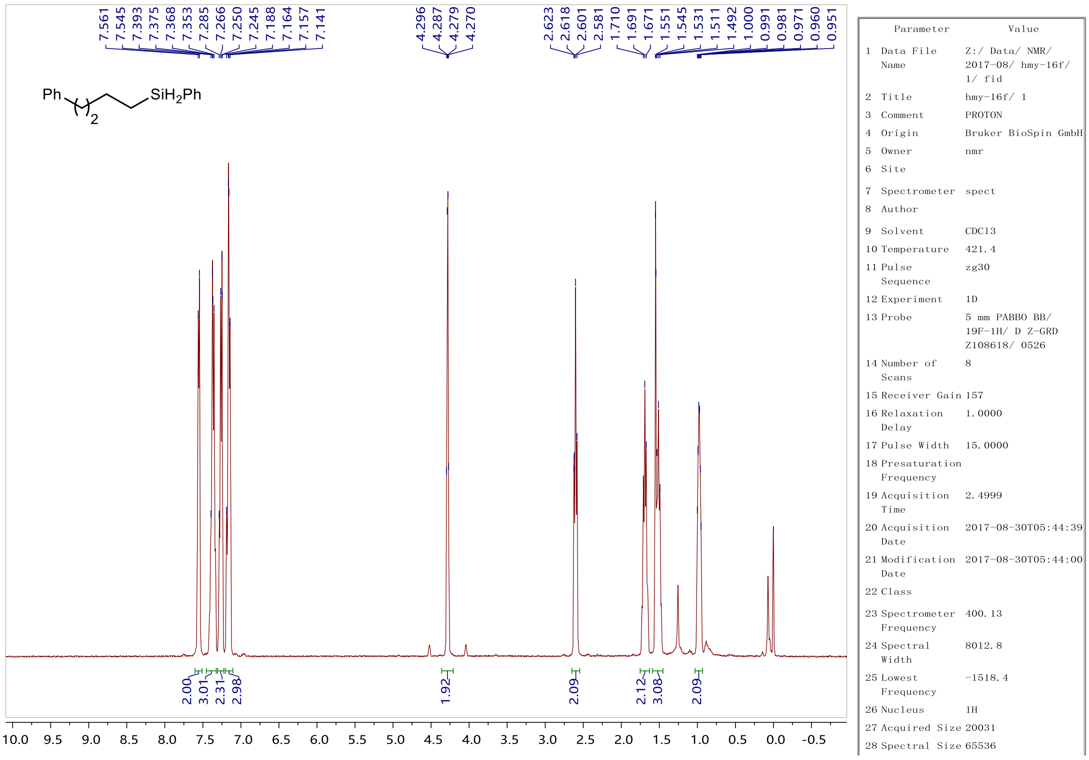

Supplementary Figure 118 | ^{1}H NMR (400 MHz, $CDCl_3$) spectra for compound 16f.

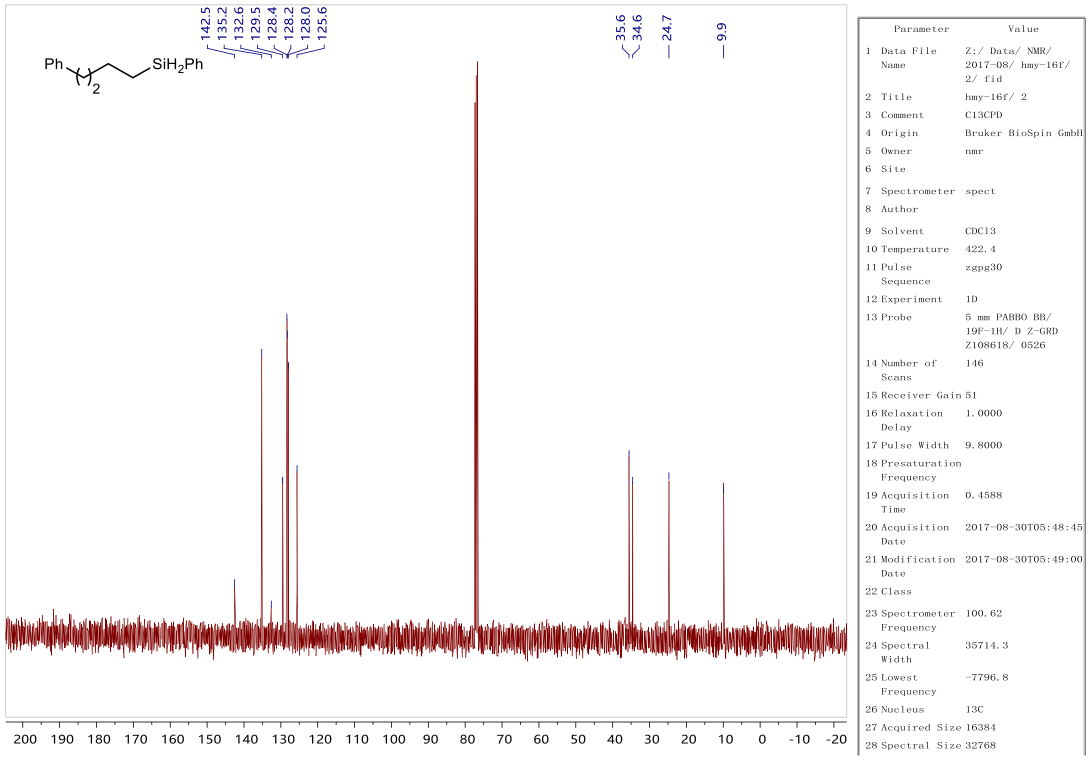

Supplementary Figure 119 | ^{13}C NMR (101 MHz, $CDCl_3$) spectra for compound 16f.

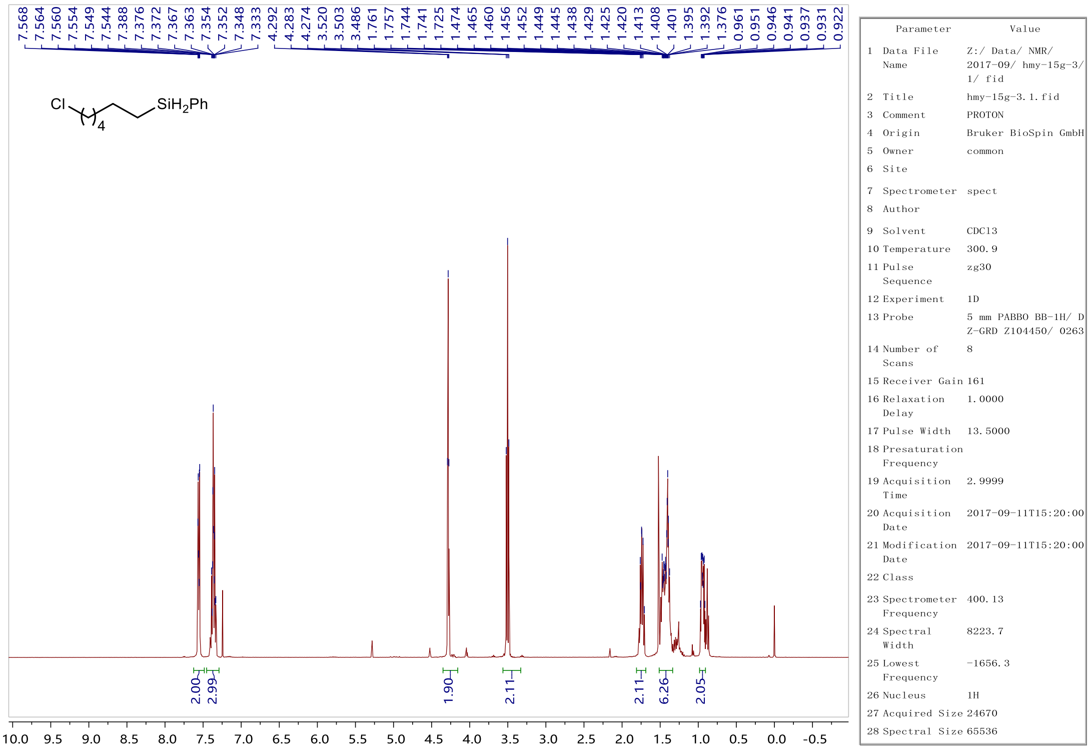

Supplementary Figure 120 | ^{1}H NMR (400 MHz, CDCl$_3$) spectra for compound 16g.

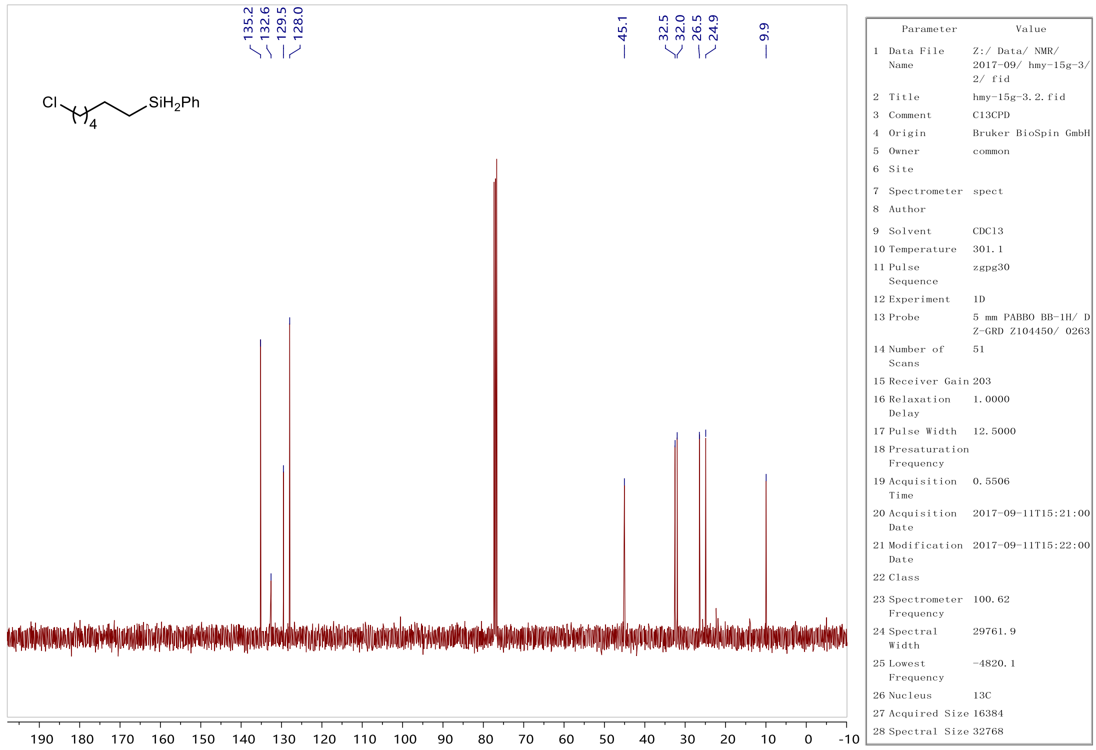

Supplementary Figure 121 | ^{13}C NMR (101 MHz, CDCl$_3$) spectra for compound 16g.

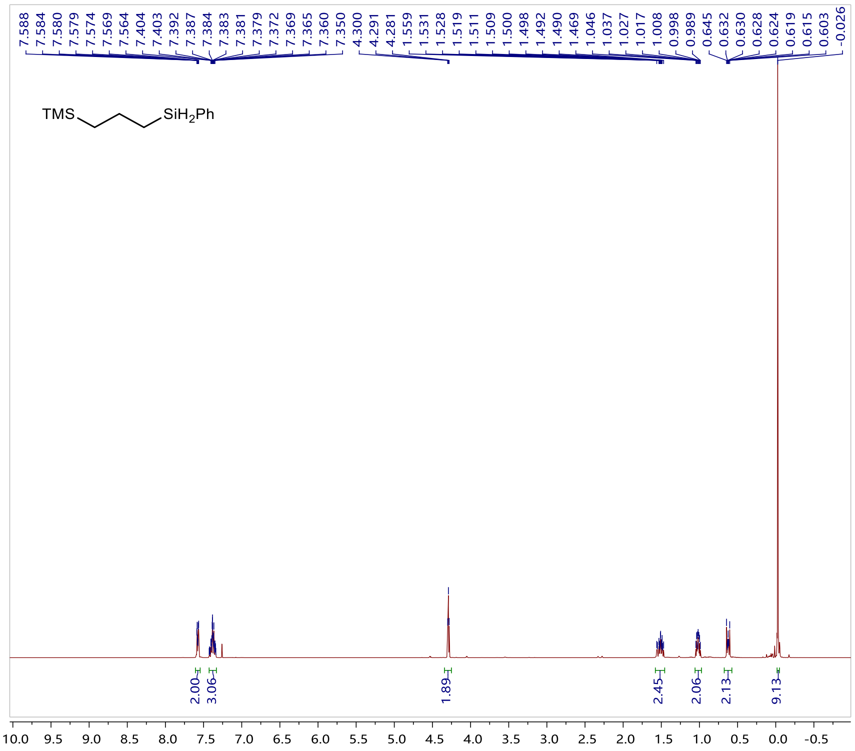

	Parameter	Value
1	Data File Name	Z:/ Data/ NMR/ 2017-08/ hmy-16e/ 1/ fid
2	Title	hmy-16e. 1. fid
3	Comment	PROTON
4	Origin	Bruker BioSpin GmbH
5	Owner	common
6	Site	
7	Spectrometer	spect
8	Author	
9	Solvent	CDCl3
10	Temperature	292. 5
11	Pulse Sequence	zg30
12	Experiment	1D
13	Probe	5 mm PABBO BB-1H/ D Z-GRD Z104450/ 0263
14	Number of Scans	8
15	Receiver Gain	114
16	Relaxation Delay	1. 0000
17	Pulse Width	13. 5000
18	Presaturation Frequency	
19	Acquisition Time	2. 9999
20	Acquisition Date	2017-07-30T07:54:00
21	Modification Date	2017-07-30T07:55:00
22	Class	
23	Spectrometer Frequency	400. 13
24	Spectral Width	8223. 7
25	Lowest Frequency	-1640. 9
26	Nucleus	1H
27	Acquired Size	24670
28	Spectral Size	65536

Supplementary Figure 122 | ^{1}H NMR (400 MHz, $CDCl_3$) spectra for compound 16h.

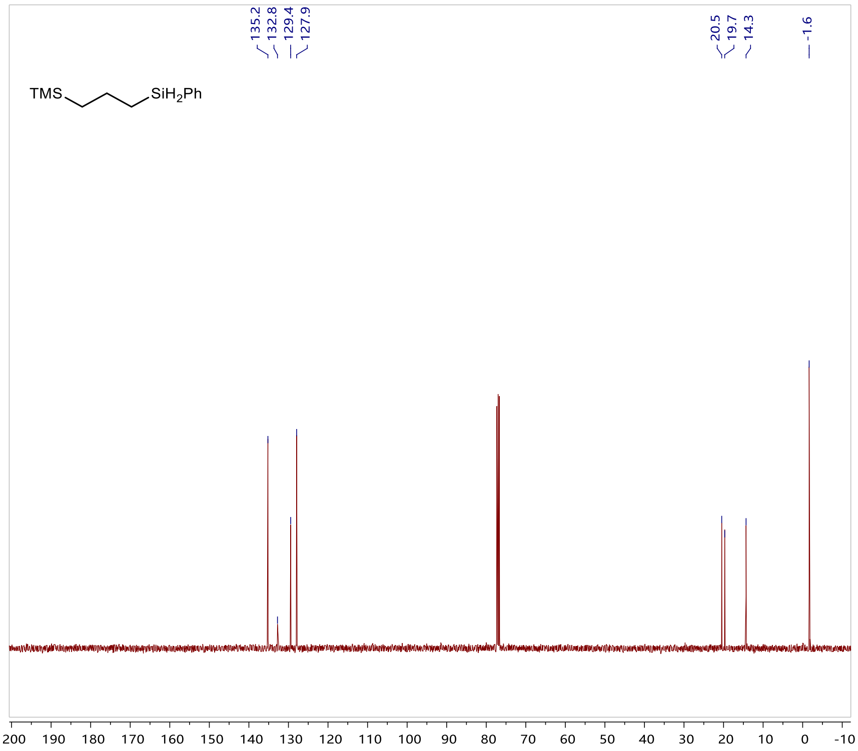

	Parameter	Value
1	Data File Name	Z:/ Data/ NMR/ 2017-08/ hmy-16e/ 2/ fid
2	Title	hmy-16e. 2. fid
3	Comment	C13CPD
4	Origin	Bruker BioSpin GmbH
5	Owner	common
6	Site	
7	Spectrometer	spect
8	Author	
9	Solvent	CDCl3
10	Temperature	292. 4
11	Pulse Sequence	zgpg30
12	Experiment	1D
13	Probe	5 mm PABBO BB-1H/ D Z-GRD Z104450/ 0263
14	Number of Scans	281
15	Receiver Gain	203
16	Relaxation Delay	1. 0000
17	Pulse Width	12. 5000
18	Presaturation Frequency	
19	Acquisition Time	0. 5506
20	Acquisition Date	2017-07-30T07:56:00
21	Modification Date	2017-07-30T08:03:00
22	Class	
23	Spectrometer Frequency	100. 62
24	Spectral Width	29761. 9
25	Lowest Frequency	-4658. 2
26	Nucleus	13C
27	Acquired Size	16384
28	Spectral Size	32768

Supplementary Figure 123 | ^{13}C NMR (101 MHz, $CDCl_3$) spectra for compound 16h.

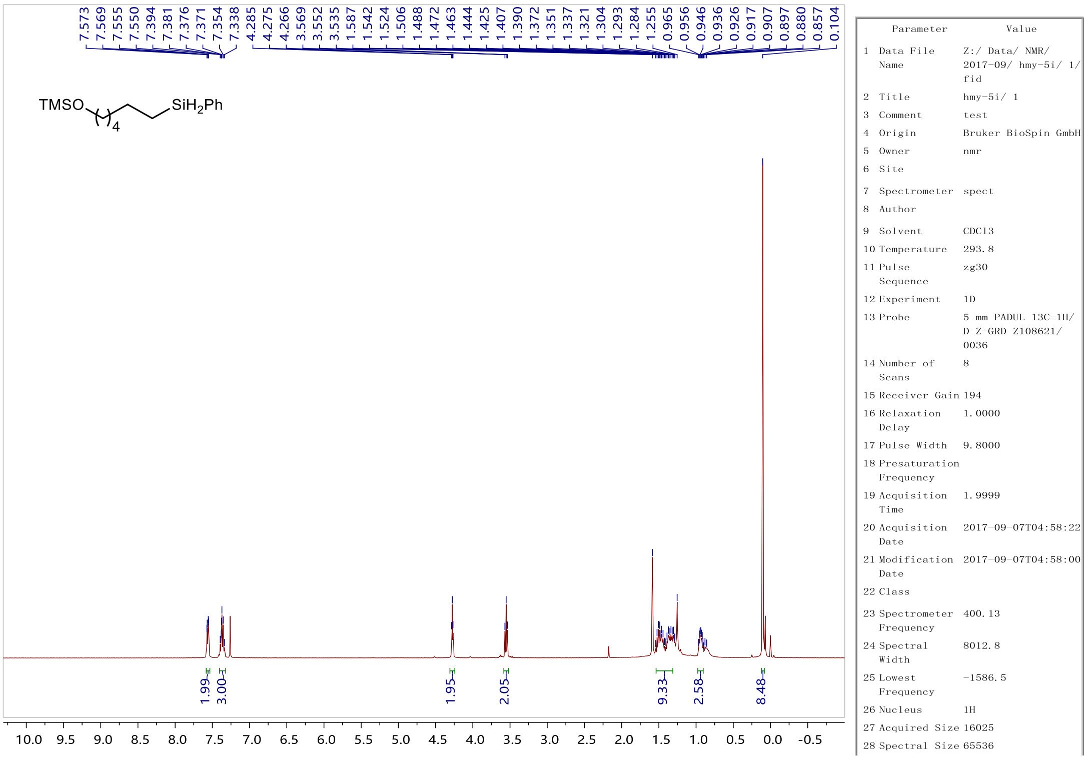

Supplementary Figure 124 | ^{1}H NMR (400 MHz, $CDCl_3$) spectra for compound 16i.

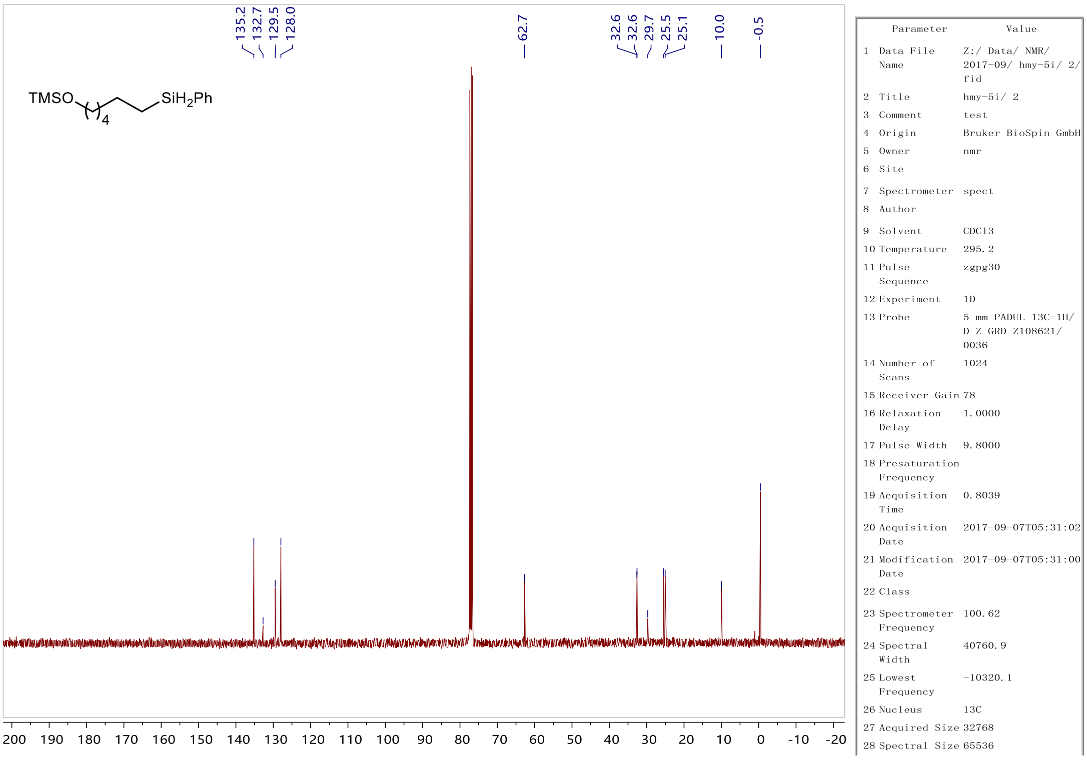

Supplementary Figure 125 | ^{13}C NMR (101 MHz, $CDCl_3$) spectra for compound 16i.

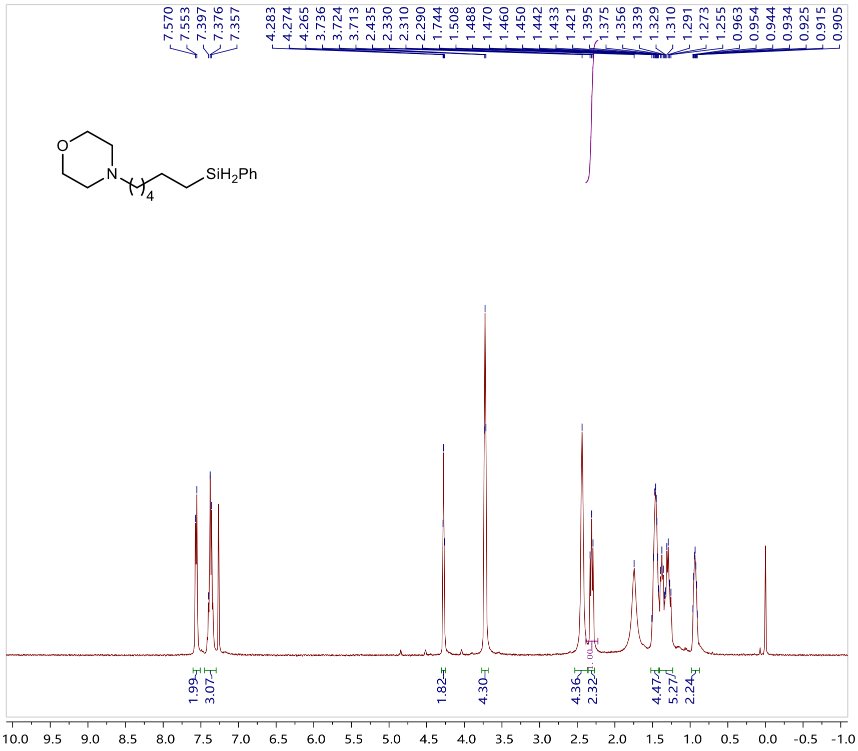

Parameter	Value
1 Data File Name	Z:/ Data/ NMR/ 2017-09/ hmy-16j/ 2/ fid
2 Title	hmy-16j.2.fid
3 Comment	PROTON
4 Origin	Bruker BioSpin GmbH
5 Owner	common
6 Site	
7 Spectrometer	spect
8 Author	
9 Solvent	CDCl3
10 Temperature	1110.2
11 Pulse Sequence	zg30
12 Experiment	1D
13 Probe	5 mm PABBO BB-1H/ D Z-GRD Z104450/ 0277
14 Number of Scans	8
15 Receiver Gain	144
16 Relaxation Delay	1.0000
17 Pulse Width	13.5000
18 Presaturation Frequency	
19 Acquisition Time	1.9923
20 Acquisition Date	2017-09-09T07:52:00
21 Modification Date	2017-09-09T07:52:00
22 Class	
23 Spectrometer Frequency	400.23
24 Spectral Width	8223.7
25 Lowest Frequency	-1640.3
26 Nucleus	1H
27 Acquired Size	16384
28 Spectral Size	65536

Supplementary Figure 126 | ^{1}H NMR (400 MHz, $CDCl_3$) spectra for compound 16j.

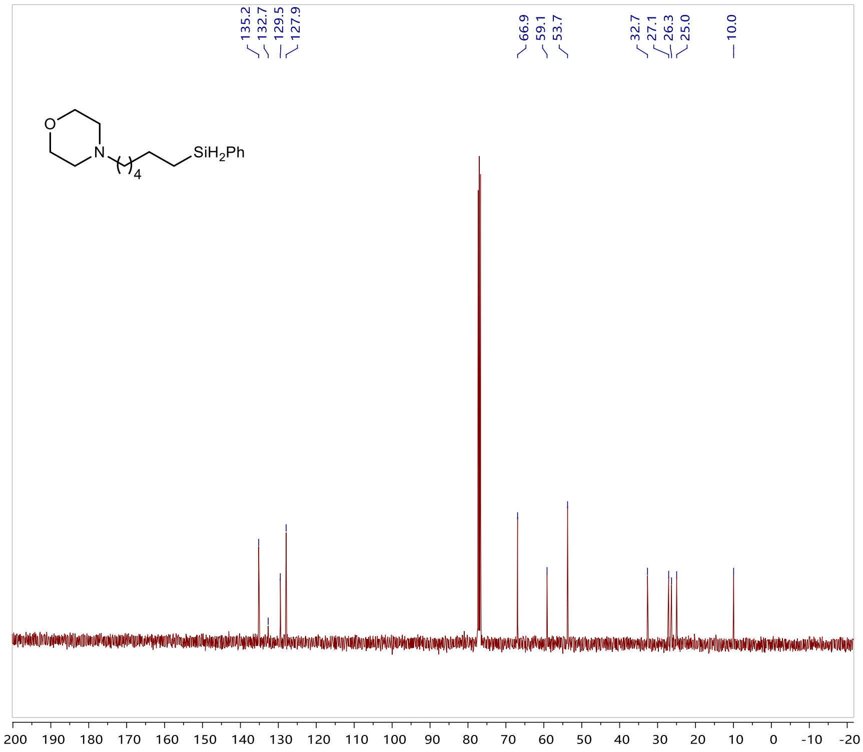

Parameter	Value
1 Data File Name	C:/ Users/ ybb/ Desktop/ hmy-16j-3/ 2/ fid
2 Title	hmy-16j-3/ 2
3 Comment	C13CPD
4 Origin	Bruker BioSpin GmbH
5 Owner	nmr
6 Site	
7 Spectrometer	spect
8 Author	
9 Solvent	CDCl3
10 Temperature	297.5
11 Pulse Sequence	zgpg30
12 Experiment	1D
13 Probe	5 mm PABBO BB/ 19F-1H/ D Z-GRD Z108618/ 0526
14 Number of Scans	722
15 Receiver Gain	119
16 Relaxation Delay	1.0000
17 Pulse Width	9.8000
18 Presaturation Frequency	
19 Acquisition Time	0.4588
20 Acquisition Date	2017-09-09T16:47:04
21 Modification Date	2017-09-09T16:47:00
22 Class	
23 Spectrometer Frequency	100.62
24 Spectral Width	35714.3
25 Lowest Frequency	-7796.8
26 Nucleus	13C
27 Acquired Size	16384
28 Spectral Size	32768

Supplementary Figure 127 | ^{13}C NMR (101 MHz, $CDCl_3$) spectra for compound 16j.

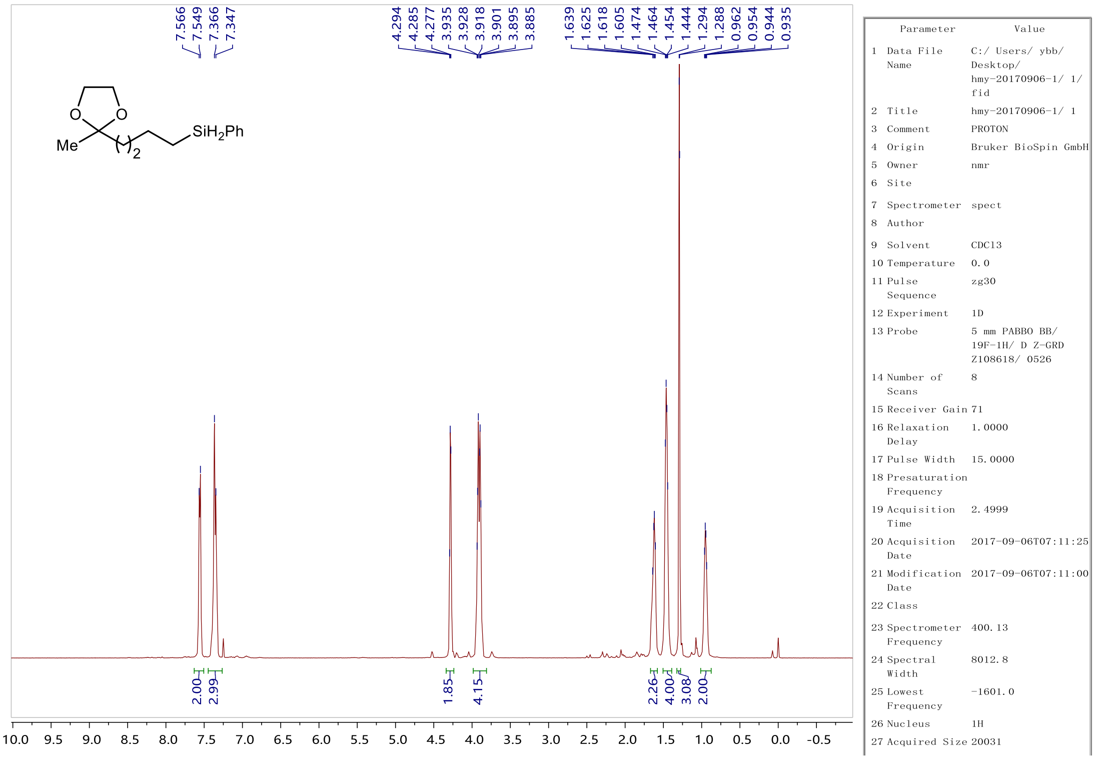

Supplementary Figure 128 | ^{1}H NMR (400 MHz, $CDCl_3$) spectra for compound 16k.

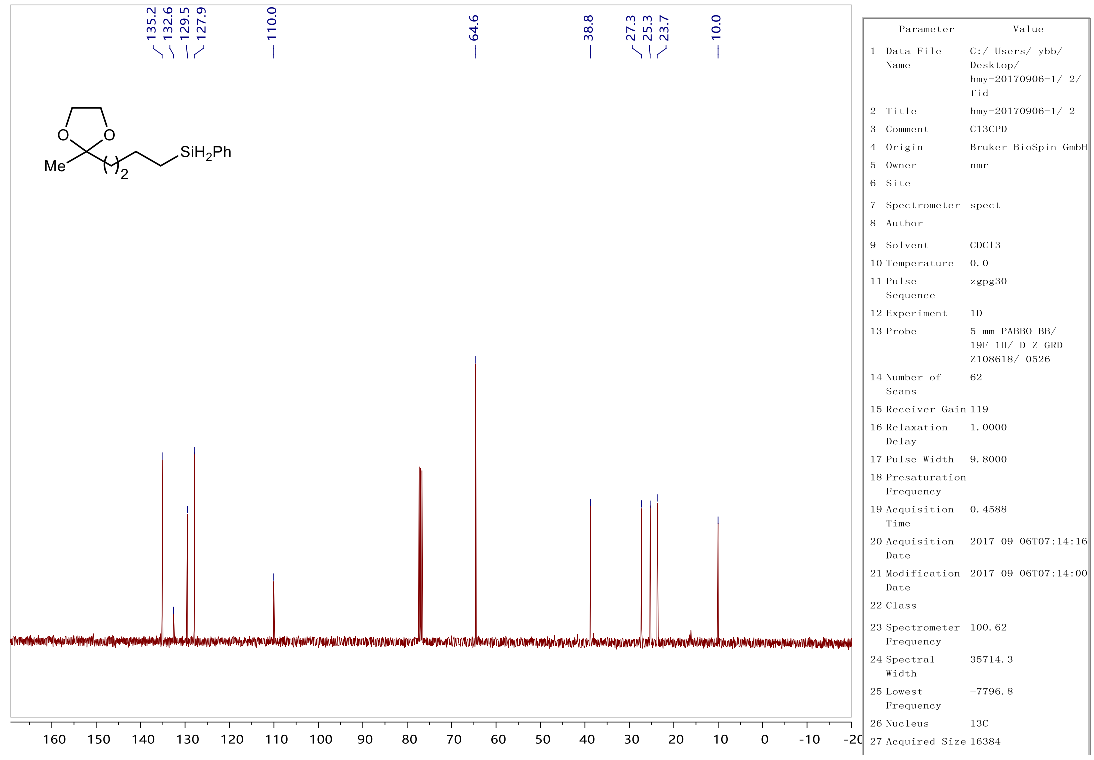

Supplementary Figure 129 | ^{13}C NMR (101 MHz, $CDCl_3$) spectra for compound 16k.

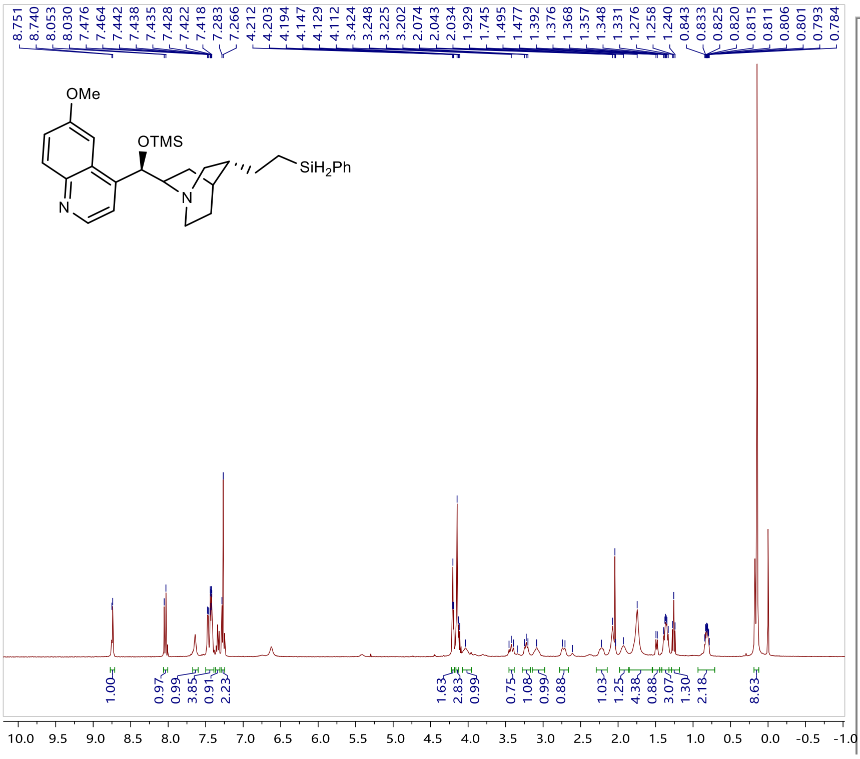

Parameter		Value
1	Data File Name	C:/ Users/ ybb/ Desktop/ hmy-161-11/ 1/ fid
2	Title	hmy-161-11/ 1
3	Comment	PROTON
4	Origin	Bruker BioSpin GmbH
5	Owner	nmr
6	Site	
7	Spectrometer	spect
8	Author	
9	Solvent	CDCl3
10	Temperature	300.5
11	Pulse Sequence	zg30
12	Experiment	1D
13	Probe	5 mm PABBO BB/ 19F-1H/ D Z-GRD Z108618/ 0526
14	Number of Scans	8
15	Receiver Gain	174
16	Relaxation Delay	1.0000
17	Pulse Width	15.0000
18	Presaturation Frequency	
19	Acquisition Time	2.4999
20	Acquisition Date	2017-09-09T20:54:06
21	Modification Date	2017-09-09T20:54:00
22	Class	
23	Spectrometer Frequency	400.13
24	Spectral Width	8012.8
25	Lowest Frequency	-1553.6
26	Nucleus	1H
27	Acquired Size	20031
28	Spectral Size	65536

Supplementary Figure 130 | ^{1}H NMR (400 MHz, $CDCl_3$) spectra for compound 16l.

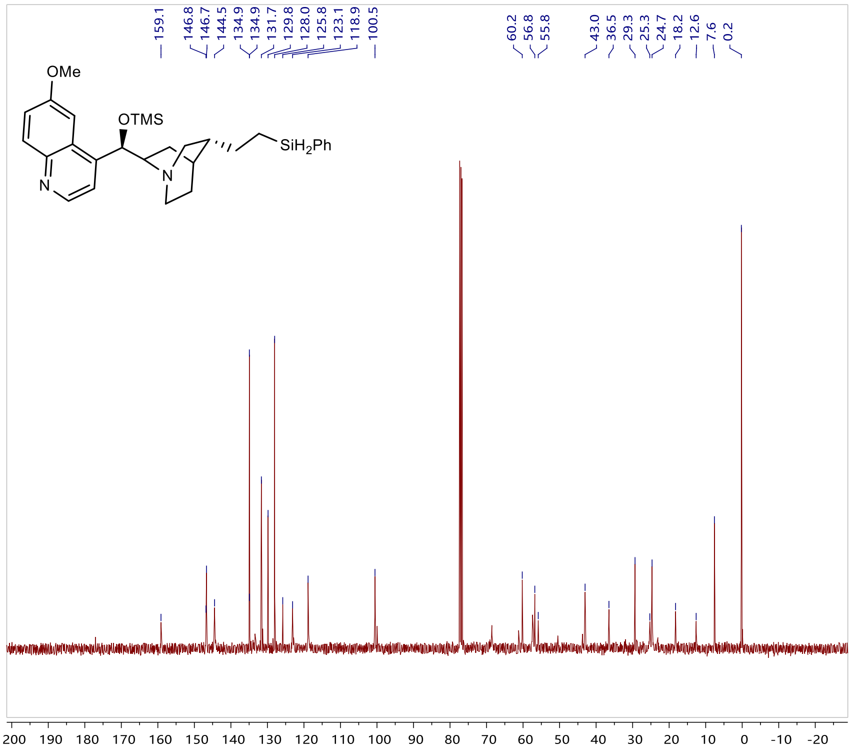

Parameter		Value
1	Data File Name	C:/ Users/ ybb/ Desktop/ hmy-161-1/ 2/ fid
2	Title	hmy-161-1.2.fid
3	Comment	C13CPD
4	Origin	Bruker BioSpin GmbH
5	Owner	common
6	Site	
7	Spectrometer	spect
8	Author	
9	Solvent	CDCl3
10	Temperature	314.1
11	Pulse Sequence	zgpg30
12	Experiment	1D
13	Probe	5 mm PABBO BB-1H/ D Z-GRD Z108618/ 0258
14	Number of Scans	763
15	Receiver Gain	203
16	Relaxation Delay	1.0000
17	Pulse Width	12.0000
18	Presaturation Frequency	
19	Acquisition Time	0.4588
20	Acquisition Date	2017-09-09T20:11:00
21	Modification Date	2017-09-09T20:30:00
22	Class	
23	Spectrometer Frequency	100.62
24	Spectral Width	35714.3
25	Lowest Frequency	-7823.5
26	Nucleus	13C
27	Acquired Size	16384
28	Spectral Size	32768

Supplementary Figure 131 | ^{13}C NMR (101 MHz, $CDCl_3$) spectra for compound 16l.

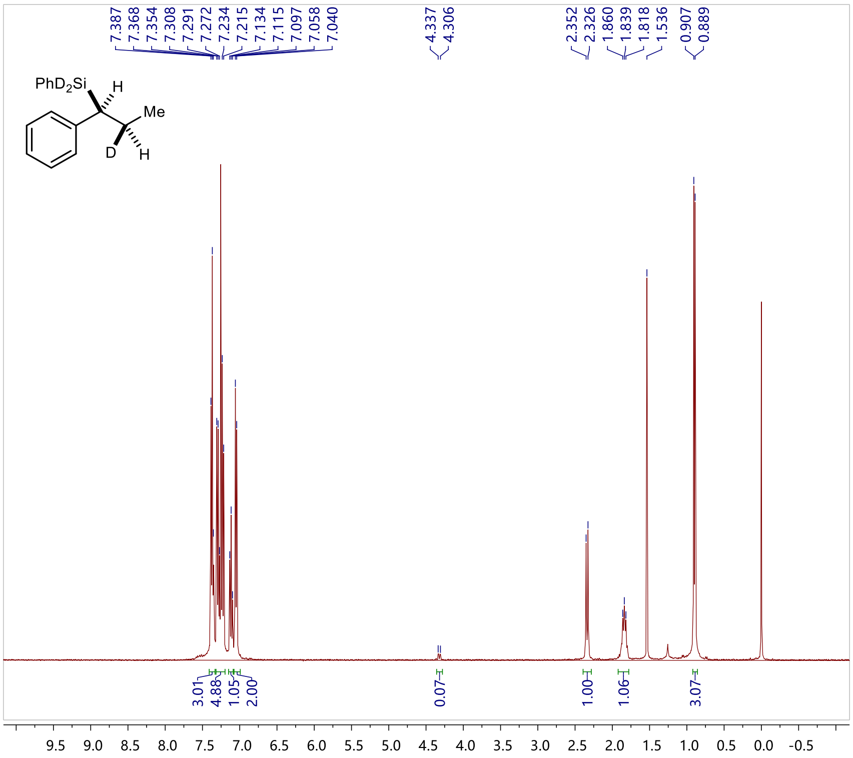

Parameter	Value
1 Data File Name	Z:/ Data/ NMR/ 2016-07/ hmy-2016-7-16-1-D/ 1/ fid
2 Title	hmy-2016-7-16-1-D
3 Comment	PROTON
4 Origin	Bruker BioSpin GmbH
5 Owner	common
6 Site	
7 Spectrometer	spect
8 Author	
9 Solvent	CDCl3
10 Temperature	-1432.1
11 Pulse Sequence	zg30
12 Experiment	1D
13 Number of Scans	8
14 Receiver Gain	203
15 Relaxation Delay	1.0000
16 Pulse Width	13.7000
17 Acquisition Time	1.9999
18 Acquisition Date	2016-07-16T12:13:00
19 Modification Date	2016-07-16T12:13:00
20 Spectrometer Frequency	400.13
21 Spectral Width	8223.7
22 Lowest Frequency	-1652.6
23 Nucleus	1H
24 Acquired Size	16446
25 Spectral Size	65536

Supplementary Figure 132 | ^{1}H NMR (400 MHz, $CDCl_3$) spectra for compound 8a-*d*.

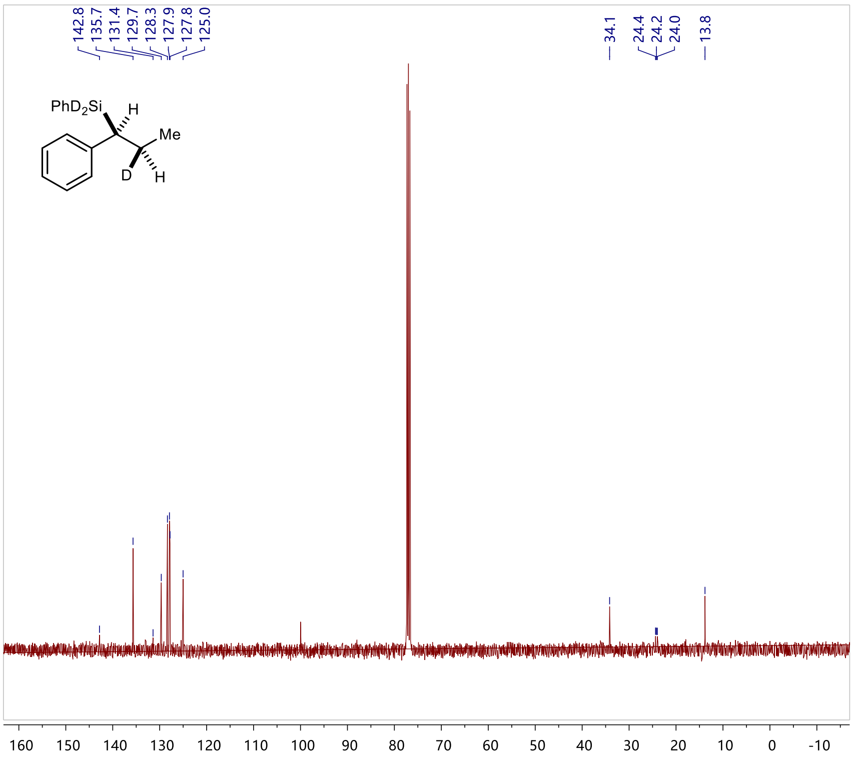

Parameter	Value
1 Data File Name	Z:/ Data/ NMR/ 2016-07/ hmy-2016-7-16-1-D/ 2/ fid
2 Title	hmy-2016-7-16-1-D
3 Comment	C13CPD
4 Origin	Bruker BioSpin GmbH
5 Owner	common
6 Site	
7 Spectrometer	spect
8 Author	
9 Solvent	CDCl3
10 Temperature	-65.7
11 Pulse Sequence	zgpg30
12 Experiment	1D
13 Number of Scans	432
14 Receiver Gain	203
15 Relaxation Delay	1.0000
16 Pulse Width	12.0000
17 Acquisition Time	0.4588
18 Acquisition Date	2016-07-16T12:24:00
19 Modification Date	2016-07-16T12:25:00
20 Spectrometer Frequency	100.61
21 Spectral Width	35714.3
22 Lowest Frequency	-7795.9
23 Nucleus	13C
24 Acquired Size	16384
25 Spectral Size	32768

Supplementary Figure 133 | ^{13}C NMR (101 MHz, $CDCl_3$) spectra for compound 8a-*d*.

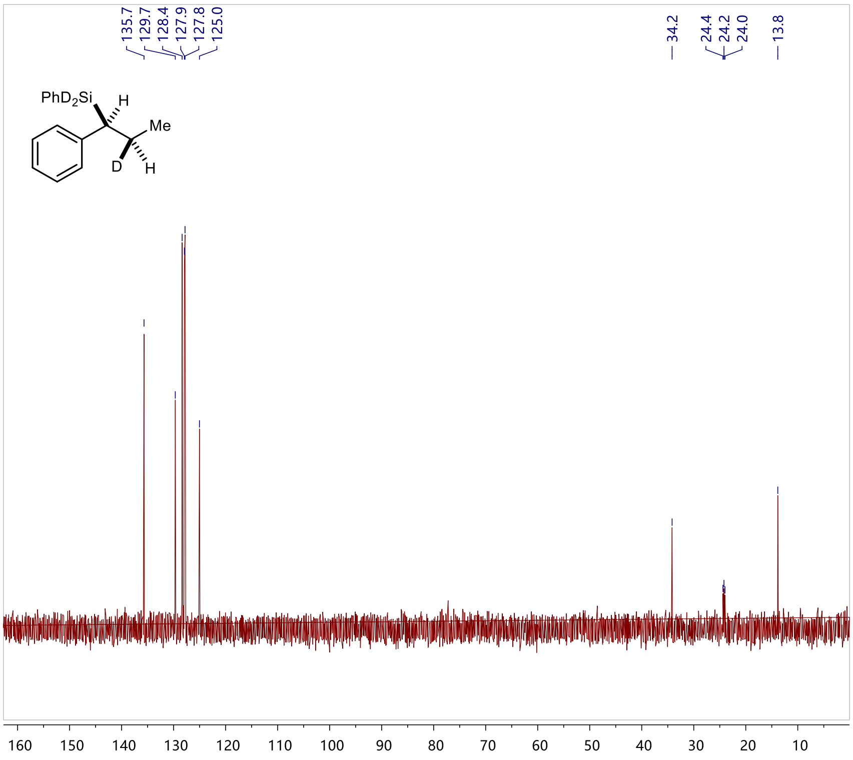

	Parameter	Value
1	Data File Name	Z:/ Data/ NMR/ 2016-07/ hmy-2016-7-16-1-D/ 3/ fid
2	Title	hmy-2016-7-16-1-D
3	Comment	C13DEPT135. sxc
4	Origin	Bruker BioSpin GmbH
5	Owner	common
6	Site	
7	Spectrometer	spect
8	Author	
9	Solvent	CDCl3
10	Temperature	-173.2
11	Pulse Sequence	dept135
12	Experiment	1D-DEPT-135
13	Number of Scans	698
14	Receiver Gain	203
15	Relaxation Delay	1.0000
16	Pulse Width	12.0000
17	Acquisition Time	1.3632
18	Acquisition Date	2016-07-16T12:54:00
19	Modification Date	2016-07-16T12:54:00
20	Spectrometer Frequency	100.61
21	Spectral Width	24038.5
22	Lowest Frequency	-1958.4
23	Nucleus	13C
24	Acquired Size	32768
25	Spectral Size	65536

Supplementary Figure 134 | ^{13}C-DEPT135 NMR (101 MHz, $CDCl_3$) spectra for compound 8a-*d*

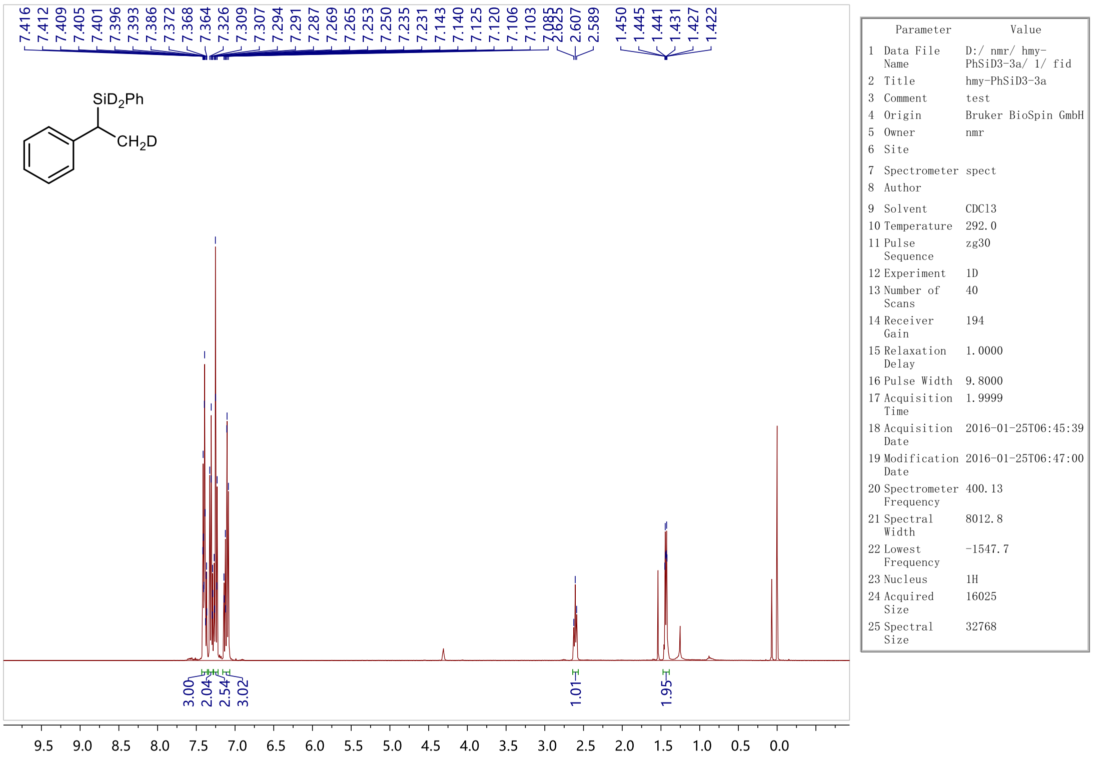

Supplementary Figure 135 | ^{1}H NMR (400 MHz, $CDCl_3$) spectra for compound 12a-*d*.

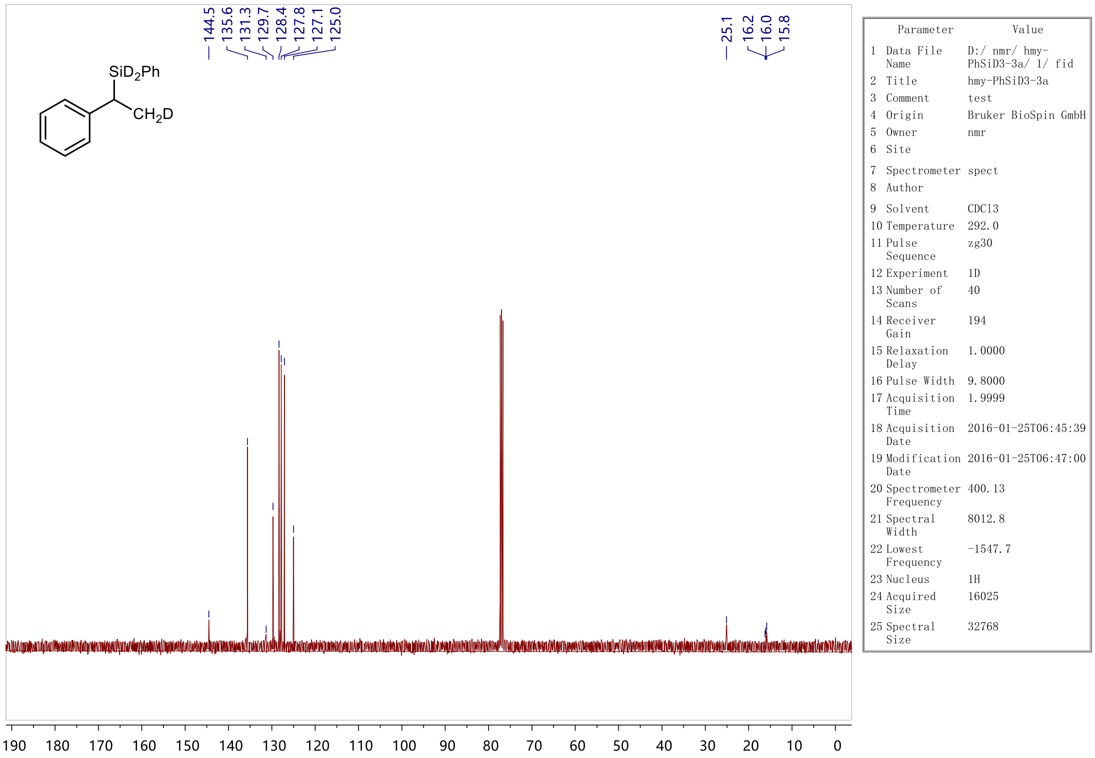

Supplementary Figure 136 | ^{13}C NMR (101 MHz, $CDCl_3$) spectra for compound 12a-*d*.

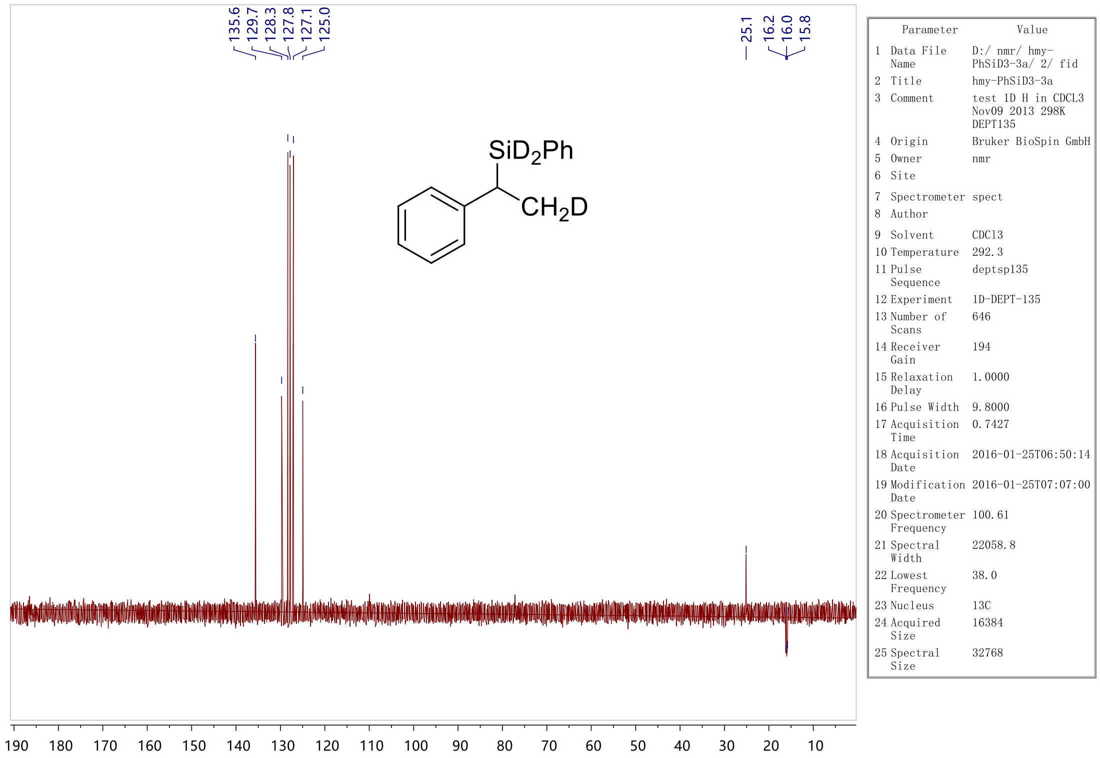

Supplementary Figure 137 | ^{13}C-DEPT135 NMR (101 MHz, $CDCl_3$) spectra for compound 12a-*d*.

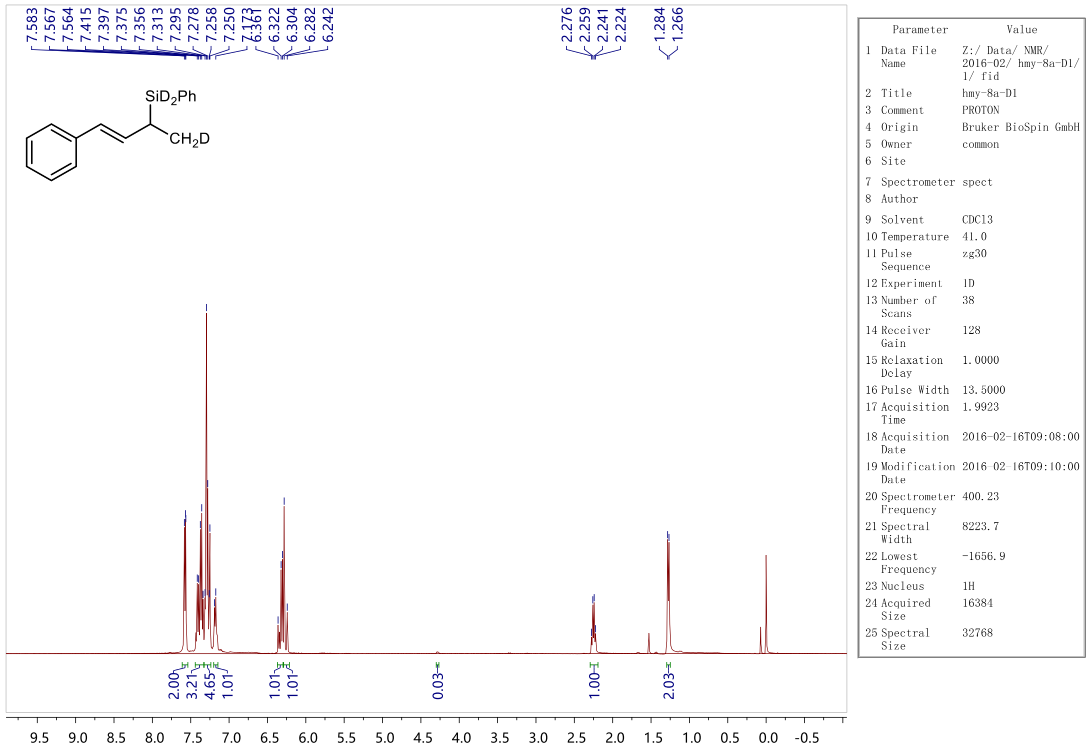

Supplementary Figure 138 | ^{1}H NMR (400 MHz, $CDCl_3$) spectra for compound 14a-*d*.

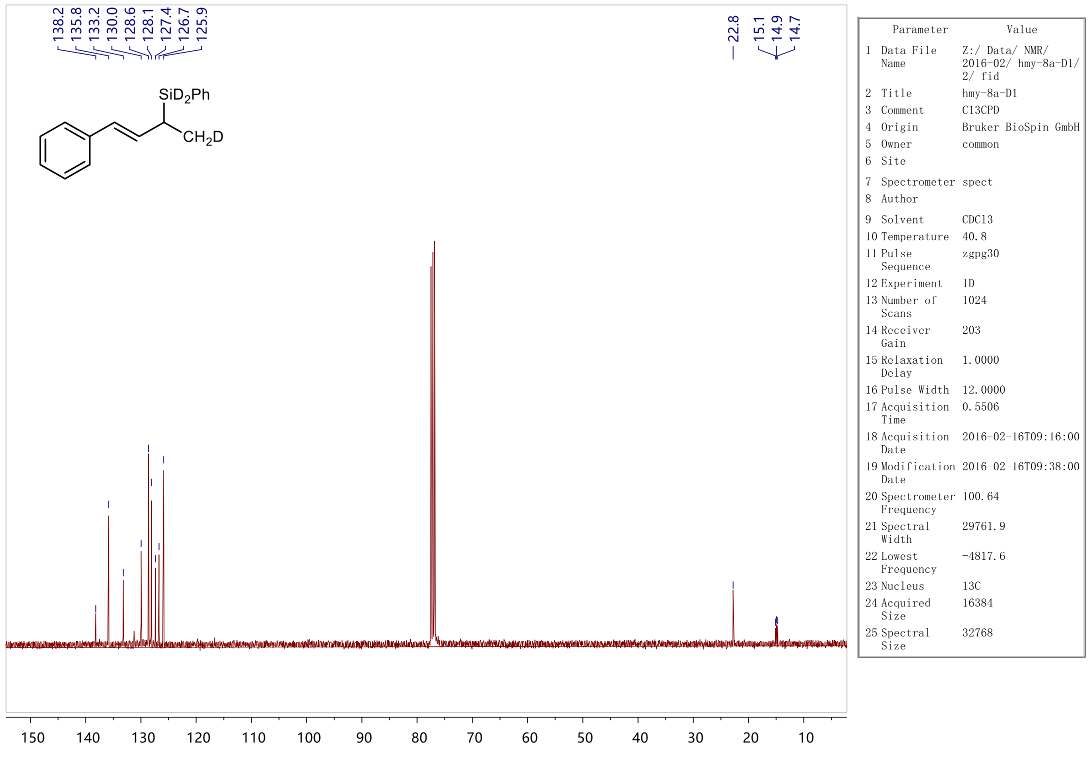

Supplementary Figure 139 | ^{13}C NMR (101 MHz, $CDCl_3$) spectra for compound 14a-*d*.

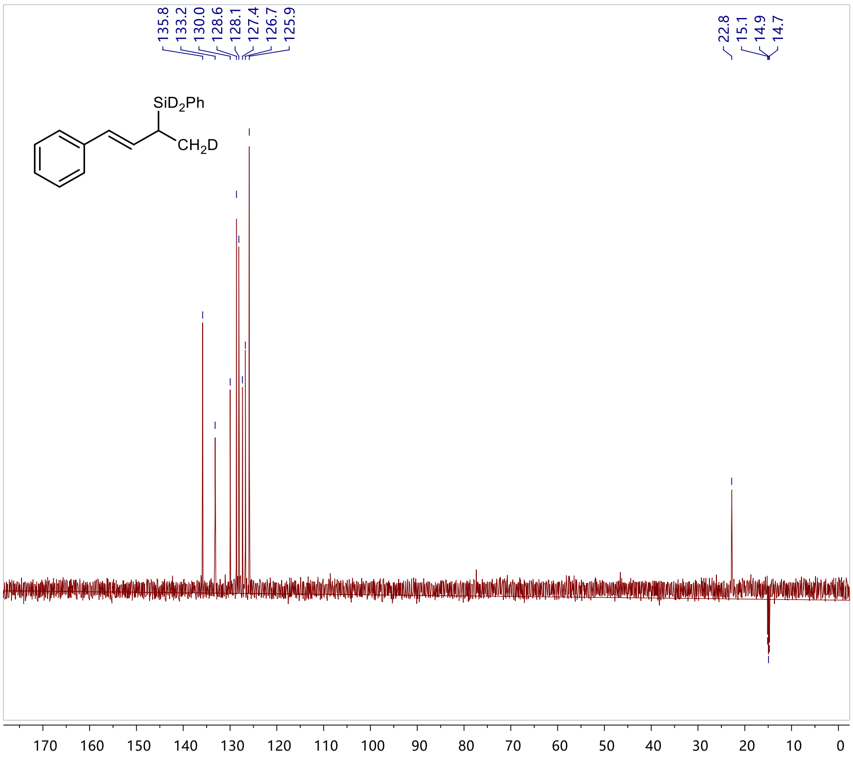

	Parameter	Value
1	Data File Name	Z:/ Data/ NMR/ 2016-02/ hmy-8a-D1/ 3/ fid
2	Title	hmy-8a-D1
3	Comment	C13DEPT135.sxc
4	Origin	Bruker BioSpin GmbH
5	Owner	common
6	Site	
7	Spectrometer	spect
8	Author	
9	Solvent	CDCl3
10	Temperature	40.6
11	Pulse Sequence	dept135
12	Experiment	1D-DEPT-135
13	Number of Scans	899
14	Receiver Gain	203
15	Relaxation Delay	1.0000
16	Pulse Width	12.0000
17	Acquisition Time	0.5506
18	Acquisition Date	2016-02-16T09:47:00
19	Modification Date	2016-02-16T10:09:00
20	Spectrometer Frequency	100.64
21	Spectral Width	29761.9
22	Lowest Frequency	-4817.6
23	Nucleus	13C
24	Acquired Size	16384
25	Spectral Size	32768

Supplementary Figure 140 | ^{13}C-DEPT135 NMR (101 MHz, $CDCl_3$) spectra for compound 14a-*d*.

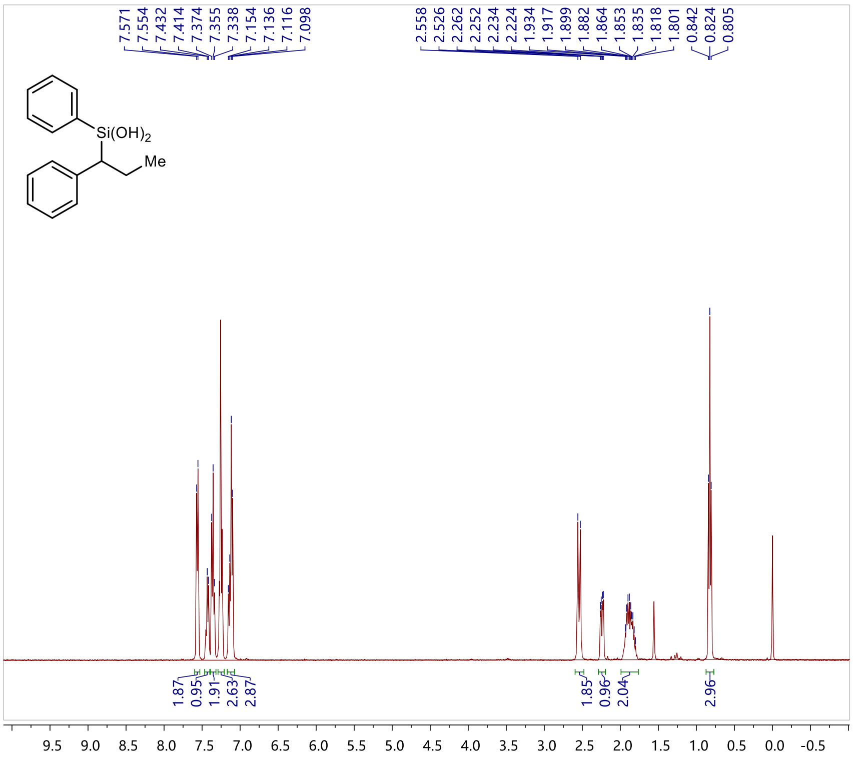

	Parameter	Value
1	Data File Name	Z:/ Data/ NMR/ 2017-02/ hmy-20170216-1/ 1/ fid
2	Title	hmy-20170216-1
3	Comment	PROTON
4	Origin	Bruker BioSpin GmbH
5	Owner	common
6	Site	
7	Spectrometer	spect
8	Author	
9	Solvent	CDCl3
10	Temperature	295.9
11	Pulse Sequence	zg30
12	Experiment	1D
13	Number of Scans	8
14	Receiver Gain	203
15	Relaxation Delay	1.0000
16	Pulse Width	13.7000
17	Acquisition Time	1.9999
18	Acquisition Date	2017-02-16T09:18:00
19	Modification Date	2017-02-16T09:18:00
20	Spectrometer Frequency	400.13
21	Spectral Width	8223.7
22	Lowest Frequency	-1651.1
23	Nucleus	1H
24	Acquired Size	16446
25	Spectral Size	65536

Supplementary Figure 141 | ^{1}H NMR (400 MHz, $CDCl_3$) spectra for compound 15.

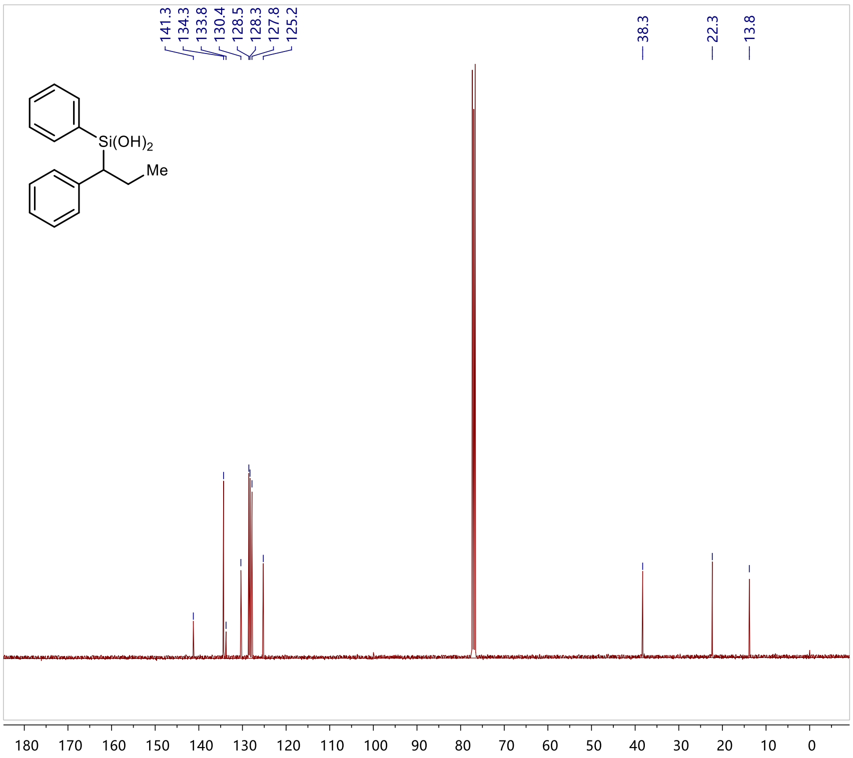

	Parameter	Value
1	Data File Name	Z:/ Data/ NMR/ 2017-02/ hmy-20170220-diOH/ 1/ fid
2	Title	hmy-20170220-diOH
3	Comment	C13CPD
4	Origin	Bruker BioSpin GmbH
5	Owner	common
6	Site	
7	Spectrometer	spect
8	Author	
9	Solvent	CDCl3
10	Temperature	297.1
11	Pulse Sequence	zgpg30
12	Experiment	1D
13	Number of Scans	5120
14	Receiver Gain	203
15	Relaxation Delay	1.0000
16	Pulse Width	12.0000
17	Acquisition Time	0.4588
18	Acquisition Date	2017-02-20T06:33:00
19	Modification Date	2017-02-20T08:43:00
20	Spectrometer Frequency	100.61
21	Spectral Width	35714.3
22	Lowest Frequency	-7795.9
23	Nucleus	13C
24	Acquired Size	16384
25	Spectral Size	32768

Supplementary Figure 142 | ^{13}C NMR (101 MHz, $CDCl_3$) spectra for compound 15.

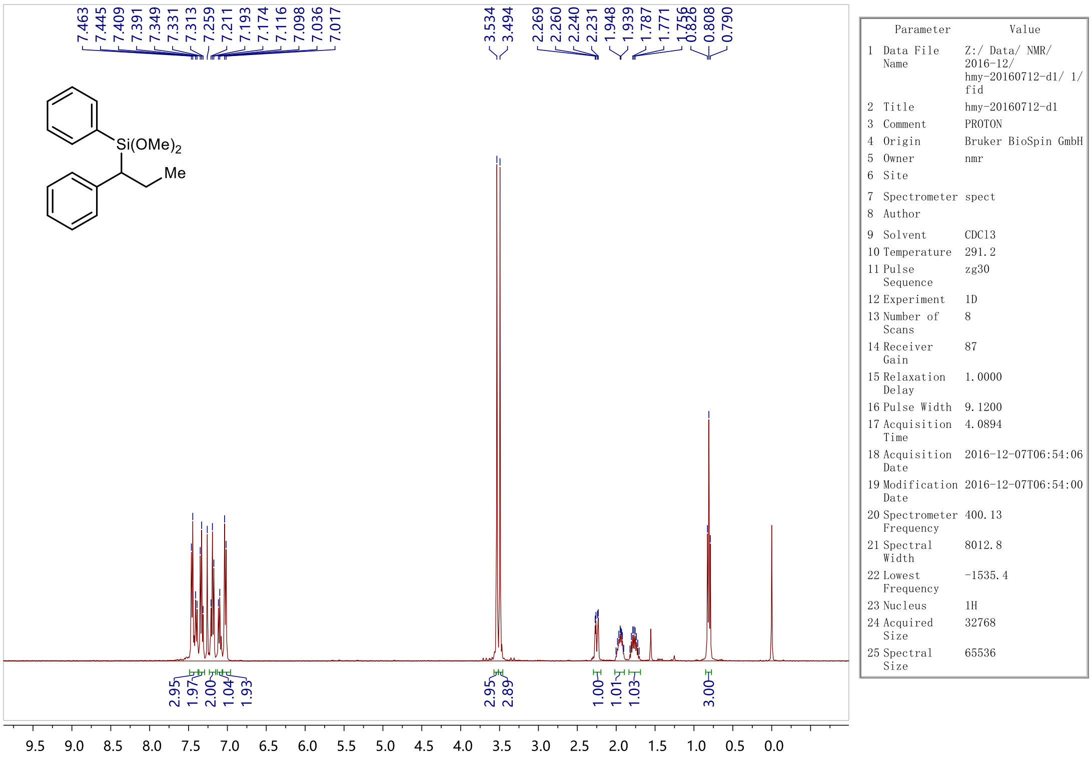

Supplementary Figure 143 | ^{1}H NMR (400 MHz, $CDCl_3$) spectra for compound 16.

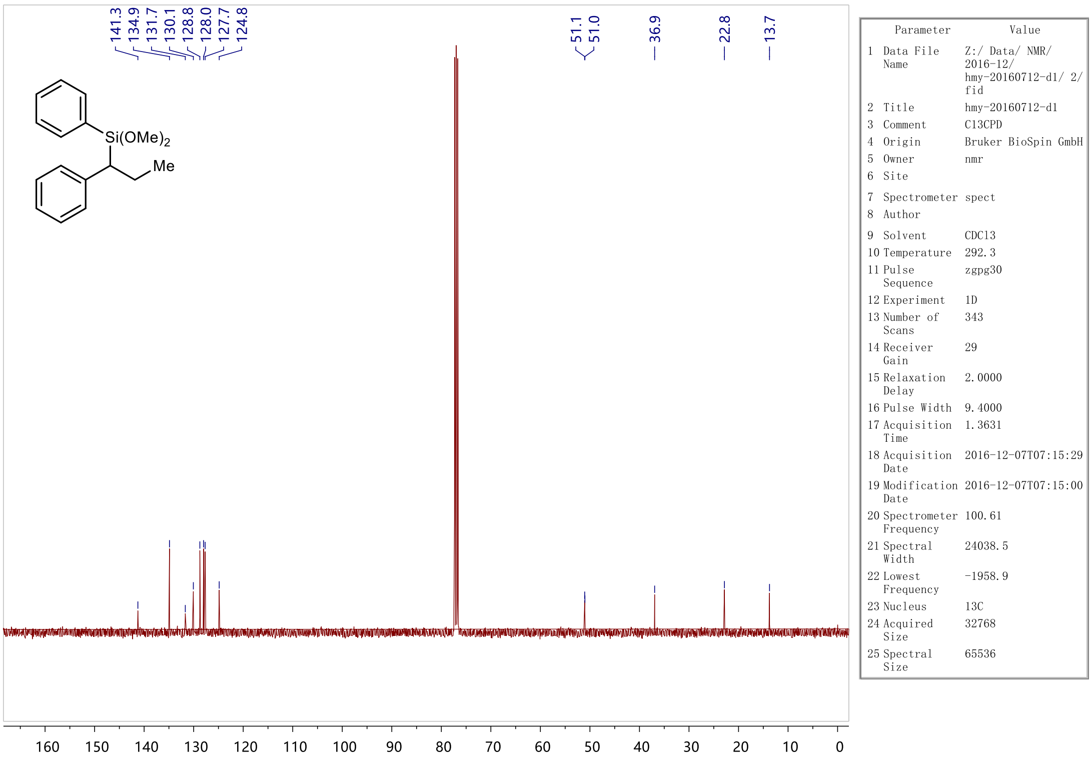

Supplementary Figure 144 | ^{13}C NMR (101 MHz, $CDCl_3$) spectra for compound 16.

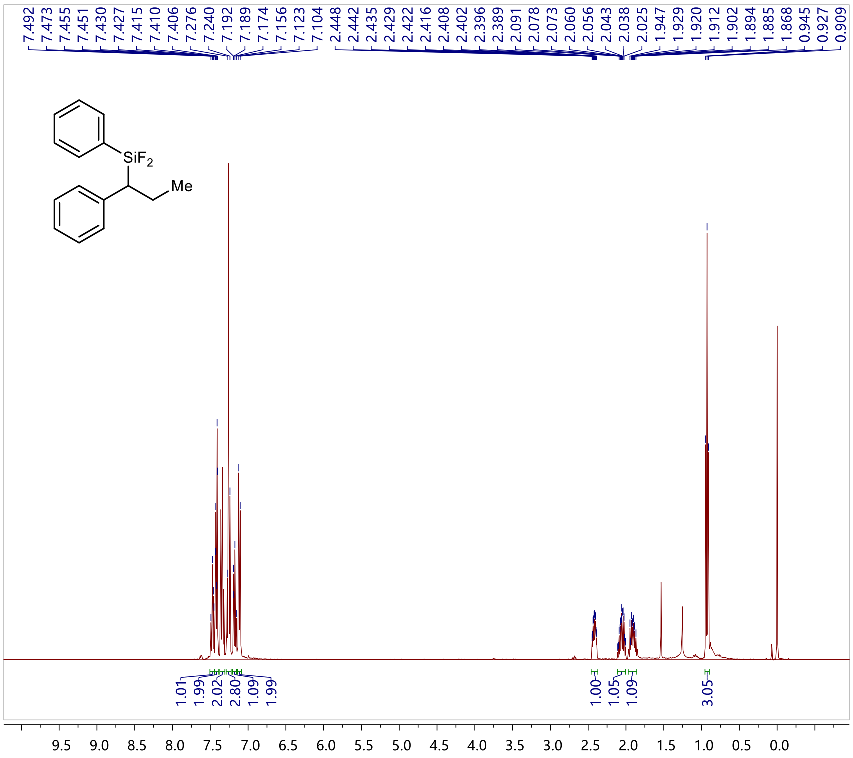

Parameter	Value
1 Data File Name	Z:/ Data/ NMR/ 2017-02/ hmy-20170228-3/ 1/ fid
2 Title	hmy-20170228-3
3 Comment	PROTON
4 Origin	Bruker BioSpin GmbH
5 Owner	common
6 Site	
7 Spectrometer	spect
8 Author	
9 Solvent	CDCl3
10 Temperature	292.0
11 Pulse Sequence	zg30
12 Experiment	1D
13 Number of Scans	8
14 Receiver Gain	203
15 Relaxation Delay	1.0000
16 Pulse Width	13.5000
17 Acquisition Time	2.9999
18 Acquisition Date	2017-02-28T14:53:00
19 Modification Date	2017-02-28T14:53:00
20 Spectrometer Frequency	400.13
21 Spectral Width	8223.7
22 Lowest Frequency	-1652.6
23 Nucleus	1H
24 Acquired Size	24670
25 Spectral Size	65536

Supplementary Figure 145 | ^{1}H NMR (400 MHz, $CDCl_3$) spectra for compound 17.

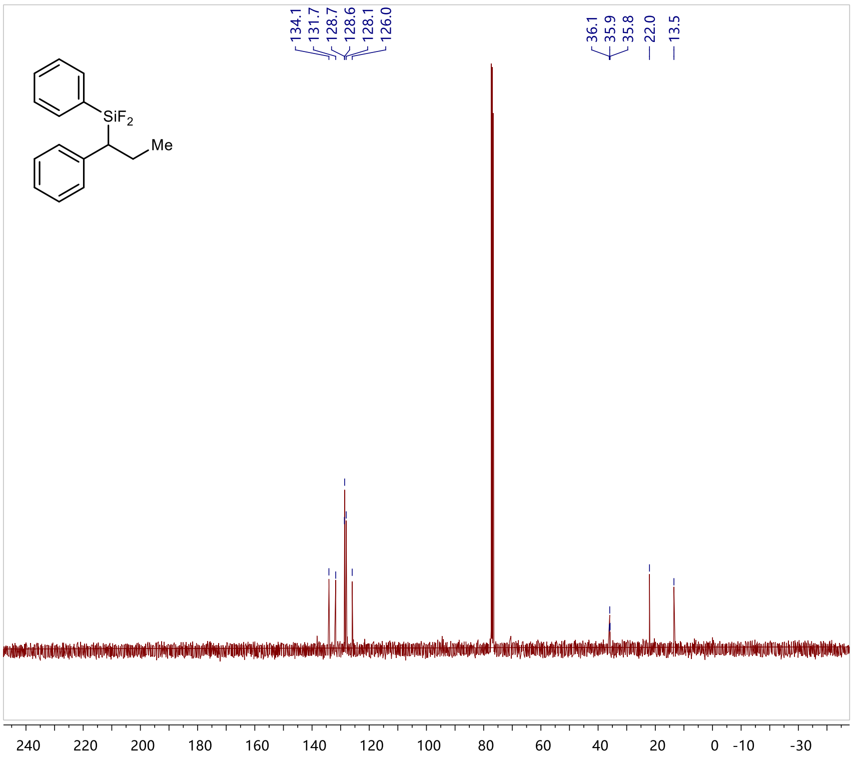

Parameter	Value
1 Data File Name	Z:/ Data/ NMR/ 2017-02/ hmy-20170228-3/ 2/ fid
2 Title	hmy-20170228-3
3 Comment	C13CPD
4 Origin	Bruker BioSpin GmbH
5 Owner	common
6 Site	
7 Spectrometer	spect
8 Author	
9 Solvent	CDCl3
10 Temperature	292.9
11 Pulse Sequence	zgpg30
12 Experiment	1D
13 Number of Scans	388
14 Receiver Gain	203
15 Relaxation Delay	1.0000
16 Pulse Width	12.5000
17 Acquisition Time	0.5506
18 Acquisition Date	2017-02-28T14:57:00
19 Modification Date	2017-02-28T15:05:00
20 Spectrometer Frequency	100.61
21 Spectral Width	29761.9
22 Lowest Frequency	-4820.2
23 Nucleus	13C
24 Acquired Size	16384
25 Spectral Size	32768

Supplementary Figure 146 | ^{13}C NMR (101 MHz, $CDCl_3$) spectra for compound 17.

Supplementary References

1. Frisch, M. J., Trucks, G. W., Schlegel, H. B., Scuseria, G. E., Robb, M. A., Cheeseman, J. R., Scalmani, G., Barone, V., Mennucci, B., Petersson, G. A., Nakatsuji, H., Caricato, M., Li, X., Hratchian, H. P., Izmaylov, A. F., Bloino, J., Zheng, G., Sonnenberg, J. L., Hada, M., Ehara, M., Toyota, K., Fukuda, R., Hasegawa, J., Ishida, M., Nakajima, T., Honda, Y., Kitao, O., Nakai, H., Vreven, T., Montgomery J. A., Jr., Peralta, J. E., Ogliaro, F., Bearpark, M., Heyd, J. J., Brothers, E., Kudin, K. N., Staroverov, V. N., Kobayashi, R., Normand, J., Raghavachari, K., Rendell, A., Burant, J. C., Iyengar, S. S., Tomasi, J., Cossi, M., Rega, N., Millam, J. M., Klene, M., Knox, J. E., Cross, J. B., Bakken, V., Adamo, C., Jaramillo, J., Gomperts, R., Stratmann, R. E., Yazyev, O., Austin, A. J., Cammi, R., Pomelli, C., Ochterski, J. W., Martin, R. L., Morokuma, K., Zakrzewski, V. G., Voth, G. A., Salvador, P., Dannenberg, J. J., Dapprich, S., Daniels, A. D., Farkas, Ö., Foresman, J. B., Ortiz, J. V., Cioslowski, J. & Fox, D. J. *Gaussian 09, Revision D.01*, Gaussian, Inc., Wallingford CT, (2009).

2. Chai, J. D. & Headgordon, M. Long-range corrected hybrid density functionals with damped atom-atom dispersion corrections. *Phys. Chem. Chem. Phys.* **10**, 6615–6620 (2008).

3. Schäfer, A., Horn, H. & Ahlrichs, R. Fully optimized contracted Gaussian basis sets for atoms Li to Kr. *J. Chem. Phys.* **97**, 2571–2577 (1992).

4. Schäfer, A., Huber, C. & Ahlrichs, R. Fully optimized contracted Gaussian basis sets of triple zeta valence quality for atoms Li to Kr. *J. Chem. Phys.* **100**, 5829–5835 (1994).

5. Weigend, F. & Ahlrichs, R. Balanced basis sets of split valence, triple zeta valence and quadruple zeta valence quality for H to Rn: Design and assessment of accuracy. *Phys. Chem. Chem. Phys.* **7**, 3297–3305 (2005).

6. Weigend, F. Accurate Coulomb-fitting basis sets for H to Rn. *Phys. Chem. Chem. Phys.* **8**, 1057–1065 (2006).

7. Cossi, M., Rega, N., Scalmani, G. & Barone, V. Energies, structures, and electronic properties of molecules in solution with the C-PCM solvation model. *J. Comp. Chem.* **24**, 669–681 (2003).

8. And, V. B. & Cossi, M. Quantum calculation of molecular energies and energy gradients in solution by a conductor solvent model. *J. Phys. Chem. A* **102**, 1995–2001 (1998).
9. Zhao, Y. & Truhlar, D. G. The M06 suite of density functionals for main group thermochemistry, thermochemical kinetics, noncovalent interactions, excited states, and transition elements: two new functionals and systematic testing of four M06-class functionals and 12 other functiona. *Theor. Chem. Acc.* **120**, 215–241 (2008).
10. Marenich, A. V., Cramer, C. J. & Truhlar, D. G. Universal solvation model based on solute electron density and on a continuum model of the solvent defined by the bulk dielectric constant and atomic surface tensions. *J. Phys. Chem. B* **113**, 6378–6396 (2009).
11. Hratchian, H. P. & Schlegel, H. B. Accurate reaction paths using a Hessian based predictor–corrector integrator. *J. Chem. Phys.* **120**, 9918–9924 (2004).
12. Hratchian, H. P. & Schlegel, H. B. Using hessian updating to increase the efficiency of a hessian based predictor corrector reaction path following method. *J. Chem. Theory Comput.* **1**, 61–69 (2005).
13. CYLview, 1.0b; Legault, C. Y. Université de Sherbrooke, 2009 (http://www.cylview.org).
14. Kuritani, M., Tashiro, S. & Shionoya, M. Heterodinuclear metal arrangement in a flat macrocycle with two chemically-equivalent metal chelating sites. *Inorg. Chem.* **51**, 1508–1515 (2012).
15. Wang, T.-L., Chen, F., Qin, J., He, Y.-M. & Fan, Q.-H. Asymmetric ruthenium-catalyzed hydrogenation of 2- and 2,9-substituted 1,10-phenanthrolines. *Angew. Chem. Int. Ed.* **52**, 7172–7176 (2013).
16. Saha, M. L., Mittal, N., Bats, J. W. & Schmittel, M. A six-component metallosupramolecular pentagon via self-sorting. *Chem. Commun.* **50**, 12189–12192 (2014).
17. Mundal, D. A., Lutz, K. E. & Thomson R. J. Stereoselective synthesis of dienes from *N*-allylhydrazones. *Org. Lett.* **11**, 465–468 (2009).

18. Barluenga, J., Tomas-Gamasa, M., Aznar, F. & Valdés, C. Synthesis of dienes by palladium-catalyzed couplings of tosylhydrazones with aryl and alkenyl halides. *Adv. Synth. Catal.* **352**, 3235–3240 (2010).

19. Miller, D. J., Yu, F., Young, N. J. & Allemann, R. K. Competitive inhibition of aristolochene synthase by phenyl-substituted farnesyl diphosphates: evidence of active site plasticity. *Org. Biomol. Chem.* **5**, 3287–3298 (2007).

20. Zhou, L., Ye, F., Zhang, Y. & Wang, J.-B. Cyclopropylmethyl palladium species from carbene migratory insertion: new routes to 1,3-butadienes. *Org. Lett.* **14**, 922–925 (2012).

21. Shirakawa, E., Ikeda, D., Yamaguchi, S. & Hayashi, T. Fe–Cu cooperative catalysis in the isomerization of alkyl Grignard reagents. *Chem. Commun.* **10**, 1214–1216 (2008).

22. Fu, P.-F., Brard, L., Li, Y. & Marks, T. J. Regioselection and enantioselection in organolanthanide-catalyzed olefin hydrosilylation. A kinetic and mechanistic study. *J. Am. Chem. Soc.* **117**, 7157–7168 (1995).

23. Hyder, I., Jiménez-Tenorio, M., Puerta, M. C. & Valerga, P. Oligomerization and regioselective hydrosilylation of styrenes catalyzed by cationic allyl nickel complexes bearing allylphosphine ligands. *Dalton Trans.* **28**, 3000–3009 (2007).

24. Greenhalgh, M. D., Frank, D. J. & Thomas, S. P. Iron-catalysed chemo-, regio-, and stereoselective hydrosilylation of alkenes and alkynes using a bench-stable iron(II) pre-catalyst. *Adv. Synth. Catal.* **356**, 584–590 (2014).

25. Peng, D. *et al.* Phosphinite-iminopyridine iron catalysts for chemoselective alkene hydrosilylation. *J. Am. Chem. Soc.* **135**, 19154–19166 (2013).

26. Barnes, G. H., Jr. & Daughenbaugh, N. E. The preparation of organosilanols via the metal-catalyzed reaction of organosilicon hydrides with water. *J. Org. Chem.* **31**, 885–887 (1966).

27. Ojima, Y., Yamaguchi, K. & Mizuno, N. An efficient solvent-free route to silyl esters and silyl ethers. *Adv. Synth. Catal.* **351**, 1405–1411 (2009).

28. Battilocchio, C., Hawkins, J. M. & Ley, S. V. A mild and efficient flow procedure for the transfer hydrogenation of ketones and aldehydes using hydrous zirconia. *Org. Lett.* **15**, 2278–2281 (2013).

29. Gulia, N., Pigulski, B., Charewicz, M. & Szafert, S. A versatile and highly efficient method for 1-chlorination of terminal and trialkylsilyl-protected alkynes. *Chem. Eur. J.* **20**, 2746–2749 (2014).

30. Scholz, R. *et al*. Enantioselective synthesis, configurational stability, and reactivity of lithium α-*tert*-butylsulfonyl carbanion salts. *Eur. J. Org. Chem.* **24**, 4588–4616 (2010).

31. Fronza, G., Fuganti, C., Grasselli, P. & Mele, A. On the mode of Bakers' yeast transformation of 3-chloropropiophenone and related ketones. Synthesis of (2*S*)-[2-2H]propiophenone, (*R*)-fluoxetine, and (*R*)- and (*S*)-fenfluramine. *J. Org. Chem.* **56**, 6019–6023 (1991).
